# *N*-Heterocyclic carbene-catalyzed enantioselective hetero-[10 + 2] annulation

**DOI:** 10.1038/s42004-020-00425-7

**Published:** 2020-11-27

**Authors:** Qiupeng Peng, Shi-Jun Li, Bei Zhang, Donghui Guo, Yu Lan, Jian Wang

**Affiliations:** 1 School of Pharmaceutical Sciences, Collaborative Innovation Center for Diagnosis and Treatment of Infectious Diseases Key Laboratory of Bioorganic Phosphorous Chemistry and Chemical Biology (Ministry of Education), Tsinghua University, Beijing, 100084 China; 2 College of Chemistry, and Institute of Green Catalysis, Zhengzhou University, Zhengzhou, 450001 China; 3 School of Chemistry and Chemical Engineering, Chongqing Key Laboratory of Theoretical and Computational Chemistry, Chongqing University, Chongqing, 400030 China

**Keywords:** Asymmetric catalysis, Synthetic chemistry methodology

## Abstract

Higher-order cycloadditions are a powerful strategy for the construction of polycycles in one step. However, an efficient and concise version for the induction of asymmetry is lacking. *N*-heterocyclic carbenes are widely used organocatalysts for asymmetric synthesis and could be an ideal choice for enantioselective higher-order cycloadditions. Here, we report an enantioselective [10 + 2] annulation between catalytically formed aza-benzofulvene intermediates and trifluoromethyl ketone derivatives. This protocol exhibits a wide scope, high yields, and good ee values, reflecting a robust and efficient higher-order cycloaddition. Density functional theory calculations provide an accurate prediction of the reaction enantioselectivity, and in-depth insight to the origins of stereocontrol.

## Abstract (web summary)

High-order cycloaddition reactions are useful for the construction of polycycles in a single step, but versions that induce asymmetry are limited. Here the authors report the construction of asymmetric polycycles via *N*-heterocyclic carbene-catalyzed hetero-[10 + 2] cycloaddition of indole-2-carbaldehydes with trifluoromethyl ketone derivatives.

## Introduction

**Fig. 1:**
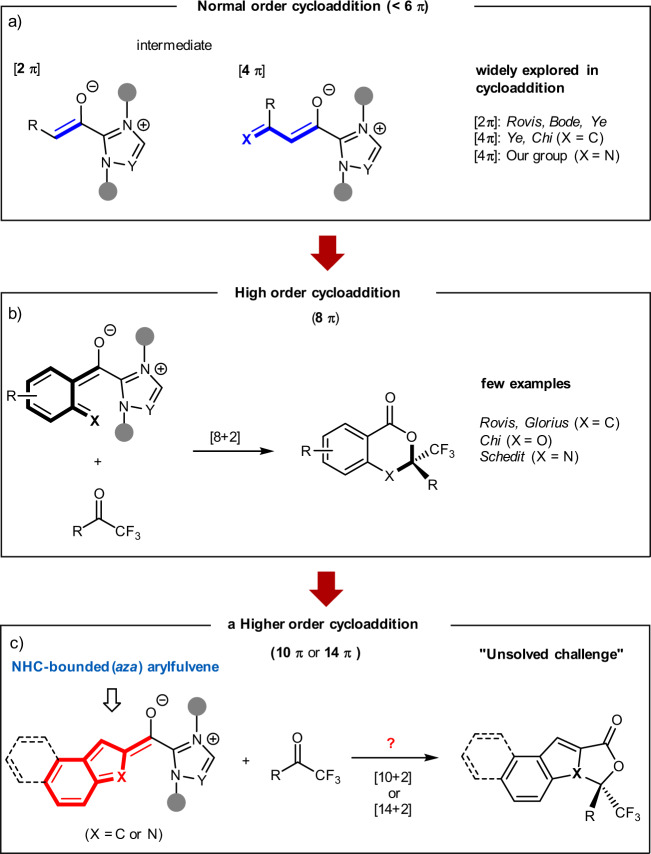
NHC-catalyzed normal and higher-order cycloaddition. **a** NHC-catalyzed normal order cycloaddition. **b** NHC-catalyzed high order cycloaddition. **c** NHC-catalyzed higher-order cycloaddition (this work).

In the past few decades, the use of chiral *N*-heterocyclic carbenes (NHCs) as asymmetric organocatalysts^1–10^, with the associated advantages of their easy operation and of carrying out enantioselective transformations in a benign environment and under mild reaction conditions, has led to impressive and continuous growth in their use. Specifically, the NHC-catalyzed asymmetric cycloaddition for the assembly of chiral mono- or polycyclic molecules has received broad attention, driven by the predominance of these chiral cyclic structures in natural products and pharmaceuticals^11,12^. In this context, normal order cycloadditions (cycloaddition that involves <6*π*-electron components) have been investigated in NHC catalysis in terms of in situ generated active enolate^13,14^ or dienolate intermediates^15^ (Fig. 1a). These pioneer works include [2 + 2]^16–20^, [2 + 3]^21–23^, [2 + 4]^24–29^, [4 + 2]^30–35^, etc.^36,37^. In 2008, Zhang et al.^18^ and Duguet et al.^16^ simultaneously realized an NHC-catalyzed [2 + 2] cycloaddition of enolates with imines, yielding versatile chiral *β*-lactams. The enantioselective [2 + 3] cycloaddition of enolates with oxaziridines or nitrovinylindoles has been reported by Shao et al.^21^ and Ni et al.^22^ groups, using NHC organocatalysis, independently. Asymmetric carbene-catalyzed [2 + 4] reaction of enolates with azadienes was also disclosed by He et al.^24^ to furnish chiral dihydropyridinones. In addition to enolates, NHC-bounded dienolates have also been successfully studied in [4 + 2]^30^ or [4 + 3]^38^ cycloadditions to generate six- or seven-membered heterocycles, respectively.

Although the above-mentioned normal order cycloaddition reactions are widely explored, the higher-order cycloaddition (≥6π-electrons) has received a high level of attention and is somehow used to concisely construct polycycles in one step fashion. Significant progress of highly stereoselective higher-order cycloadditions has been made in recent years^39–41^. Elegant relevant works in this direction involve Feng*’*s Ni-catalyzed [8 + 2] cycloaddition of azaheptafulvenes with alkylidene malonates^42,43^. The Jørgensen group enriched this field by developing a series of highly enantioselective cycloaddition reactions (e.g., [8 + 2]^44,45^, [6 + 4]^46^, and [10 + 4]^47^) promoted via enamine catalysis. However, to a certain extent^48–51^, this class of higher-order cycloadditions suffers from some drawbacks (i.e., difficult stereocontrol and lack of periselectivity), thus resulting in slower growth than normal order cycloaddition. Despite the difficulties ahead, some encouraging progress was still achieved in the direction of NHC catalysis^52–55^. Janssen-Muller^56^ and Chen and Rovis^57^ reported a [8 + 2] cycloaddition of NHC-bounded o-quinodimethane intermediates (8*π*-electron) with ketones, respectively (Fig. 1b)^58,59^. A recent Chen et al.^60^ work indicated that salicylaldehydes could be oxidized to generate NHC-bounded o-quinone methide intermediates (8*π*-electron), which participated in a [8 + 2] cycloaddition with electrophile trifluoromethyl ketones. Besides the above accomplishments, Lee et al.^61^ then successfully found that the NHC-bounded aza-*o*-quinone methide intermediates (8*π*-electron), first generated from decarboxylation of *N*-methylisatoic anhydrides, could react with trifluoromethyl ketones to deliver enantioenriched dihydrobenzoxazin-4-ones via a [8 + 2] cycloaddition strategy.

In brief, NHC-catalyzed cycloadditions ranged from [2 + 2] to [8 + 2] have been extensively investigated over the past few years, but there is a remarkable lack of higher-order cycloadditions (e.g., [10 + 2]^62,63^ and [14 + 2]). Although the intricately competitive pathways make the reaction-control difficult, these higher-order cycloadditions can provide a direct way to efficiently build polycyclic scaffolds.

Herein, we report a hetero-[10 + 2] higher-order cycloaddition of indole-2-carbaldehydes with trifluoromethyl ketone derivatives, proceeding via an NHC-bounded aza-benzofulvene intermediate (Fig. 1c). This discovery represents the initial use of NHC-bounded aza-arylfulvene intermediates in catalytic and enantioselective [10 + 2] or [14 + 2] reaction. In addition, in medicinal chemistry, the incorporation of “F”-containing fragments normally provides an effective route to enhance the metabolic stability, as well as other chemical or physical properties, of target molecules^64–66^. Based on the importance of polycyclic structures and incorporated “F”-containing fragments, the potential of these synthesized molecules in drug discovery is worth our expectation.

## Results

### Reaction optimization

We commenced our studies by investigating the reaction of indole-2-carbaldehyde **1a** and 2,2,2-trifluoroacetophenone **2a** as the model substrates, K_2_CO_3_ as the base, DQ as the oxidant, tetrahydrofuran as the solvent, and the results are briefly summarized in Table 1. When *L*-phenylalanine-derived triazolium NHC precatalyst **A** was exploited, the expected cycloadduct **3a** was not observed. Replacing the mesitylene group with pentafluorophenyl group triazolium NHC precatalyst **B** gave desired product **3a** in 40% yield and 0% ee, whereas the use of precatalyst **C** and **D** resulted in almost no reaction. To our delight, when indanol-derived triazolium catalyst **E** was tested, the [10 + 2] cycloadduct **3a** was successfully formed in 61% yield with 35% ee and implies that this highly enantioselective [10 + 2] annulation can be achieved in the presence of ideal conditions. The catalytic performance could be further improved by changing the X group of precatalyst **E** from H to NO_2_ (entry 6). After evaluating bases and solvents, we found that a combination of PhCO_2_Na as the base and hexane as the solvent gave the product **3a** in 80% yield and 88% ee (entry 10). Improvements in yield and enantioselectivity were found when thiourea was used as the additive to form **3a** (entry 12, 85% yield, 91% ee).

**Table 1:** Optimization of the reaction conditions^a^.



Entry	NHC cat.	Solvent	Base	Additive	Yield (%)^b^	ee (%)^c^
1	**A**	THF	K_2_CO_3_	/	trace	–
2	**B**	THF	K_2_CO_3_	/	40	0
3	**C**	THF	K_2_CO_3_	/	trace	–
4	**D**	THF	K_2_CO_3_	/	trace	–
5	**E**	THF	K_2_CO_3_	/	61	35
6	**F**	THF	K_2_CO_3_	/	70	51
7	**F**	DCM	K_2_CO_3_	/	53	59
8	**F**	Toluene	K_2_CO_3_	/	40	42
9	**F**	Hexane	Et_3_N	/	42	68
10	**F**	Hexane	PhCO_2_Na	/	80	88
11	**F**	Hexane	PhCO_2_Na	G	93	88
12	**F**	Hexane	PhCO_2_Na	H	85	91
13^d^	**F**	Hexane	PhCO_2_Na	H	74	91

^a^Conditions: **1a** (0.1 mmol), **2a** (0.12 mmol), catalyst (15 mol%), base (0.10 mmol) and DQ (0.11 mmol), solvent (1.0 mL), room temperature, 4 Å MS (30 mg), Ar, 48 h.

^b^Isolated yield after flash column chromatography.

^c^Enantiomeric excess (ee) determined via chiral-phase HPLC analysis.

^d^cat. **F** (10 mol%) was used, 72 h.

### Substrate scope

**Fig. 2:**
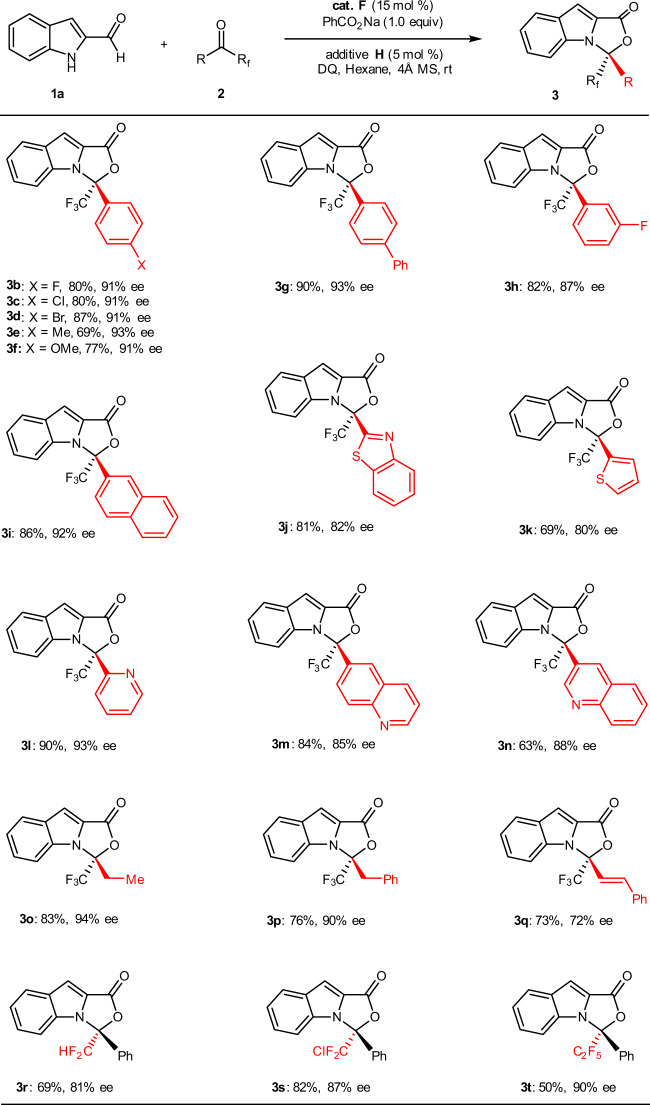
Scope of ketones. Reaction conditions: **1a** (0.2 mmol), **2** (0.24 mmol), cat. **F** (15 mol%), additive **H** (5 mol%), PhCO_2_Na (0.20 mmol) and DQ (0.22 mmol), hexane (2.0 mL), room temperature, 4 Å MS (60 mg), Ar, 36–96 h.

With the optimal catalytic system in hand, we moved our attention to exploring the generality of this asymmetric higher order [10 + 2] annulation. As illustrated in Fig. 2, by reacting with indole-2-carbaldehyde **1a**, an array of aryl trifluoromethyl ketones **2** was examined first. In the reactions to generate the [10 + 2] cycloadducts **3**, yields and enantioselectivities were found to be independent of the electronic properties of the substituents on the aryl group in **2** (**3b−i**). When the heteroaryl trifluoromethyl ketones were reacted with indole-2-carbaldehyde **1a** under optimal conditions, an [10 + 2] annulation was efficiently realized in all cases (**3j−n**). Reactions attempted using the alkyl trifluoromethyl ketones gave their corresponding [10 + 2] cycloadducts in good yields with high ee values (**3o** and **3p**). Whereas the alkenyl trifluoromethyl ketone **2q** was reacted with **1a**, product **3q** was also obtained in a good yield (73%) but with a slightly diminished enantioselectivity (72% ee). Switching the fluorinated substituent from CF_3_ to CF_2_H, ClCF_2_, or C_2_F_5_ in ketones, synthetic useful yields, and high to excellent enantioselectivities were still obtained under current conditions (**3r−t**).

**Fig. 3:**
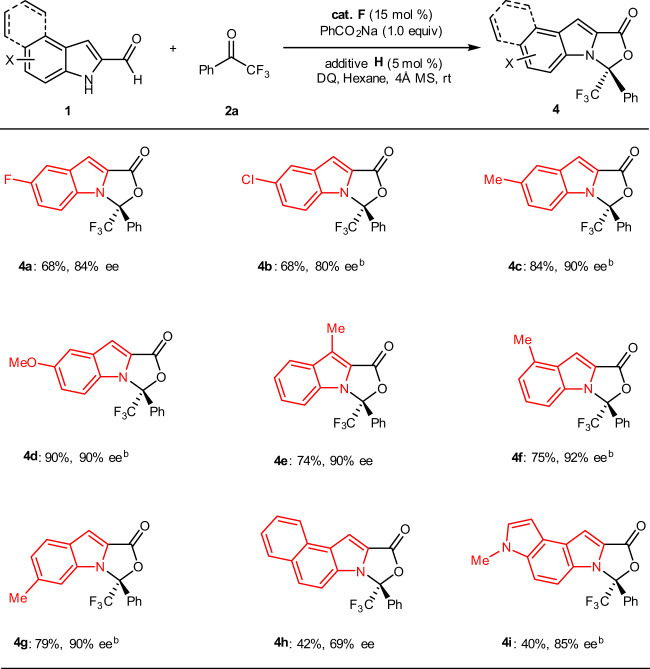
Scope of indole-2-carbaldehydes **1**. Reaction conditions: **1** (0.2 mmol), **2a** (0.24 mmol), cat. **F** (15 mol%), additive **H** (5 mol%), PhCO_2_Na (0.20 mmol) and DQ (0.22 mmol), hexane (2.0 mL), room temperature, 4 Å MS (60 mg), Ar, 36–96 h. ^b^DCM-Hexane (1:5) was used.

Next, we turned our focus to investigate the scope of substrate **1** (Fig. 3). Different substituents and substitution patterns on the indole skeleton were examined comprehensively. Electron-withdrawing substituents such as halo (**4a** and **4b**) units on the phenyl ring of the aldehyde substrates were well tolerated. Electron-releasing groups such as methyl (**4c**, **4e**, **4f**, and **4g**) and methoxyl unit (**4d**) could also be installed on the indole scaffold of the aldehyde substrates. It is worth to note that this [10 + 2] protocol could be extended to a higher-order [14 + 2] cycloaddition, affording their corresponding cycloadducts (**4h** and **4i**) in good enentioselectivities albeit with acceptable but dropped yields under the current standard conditions. The absolute configuration of **3e** (CCDC 1961662) was determined by single-crystal X-ray analysis and other products were assigned by analogy.

### Postulated mechanism

**Fig. 4:**
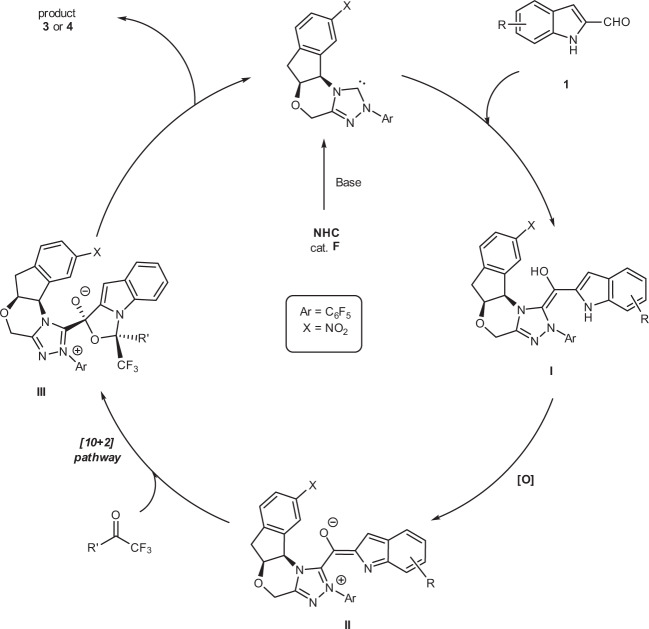
Postulated mechanistic pathways. Postulated catalytic mechanism of [10 + 2] annulation.

**Fig. 5:**
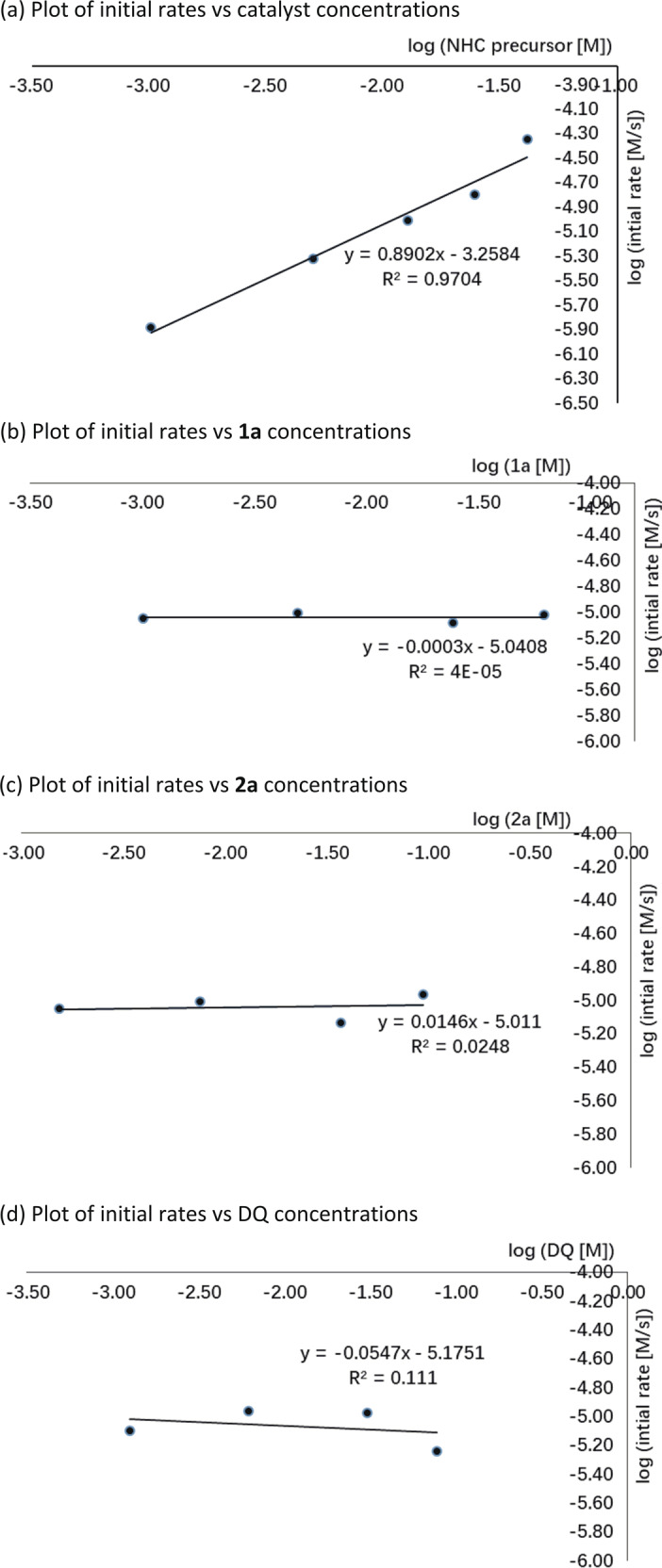
Plot of initial rates vs catalyst and substrates. **a** Plot of initial rates vs. catalyst concentrations. **b** Plot of initial rates vs. **1a** concentrations. **c** Plot of initial rates vs. **2a** concentrations. **d** Plot of initial rates vs. DQ concentrations.

A postulated catalytic mechanism of [10 + 2] annulation is summarized in Fig. 4. Deprotonation of NHC precatalyst **F** gives the corresponding NHC, which adds to aldehyde **1** to give the corresponding tetrahedral intermediate,^67,68^ with further deprotonation giving the Breslow intermediate **I**. Intermediate **I** is subsequently oxidized to the key NHC-bounded aza-benzofulvene intermediate **II**. A mass correlating to intermediate **II** was observed via high-resolution mass spectrometry (See Supplementary information (SI) Supplementary Table 1 for details). This critical intermediate **II** can promote a concerted [10 + 2] pathway or a stepwise Michael addition–acylation to form intermediate **III**, which undergoes N-acylation to release the NHC catalyst **F** for the next catalytic cycle. Kinetic experiments were conducted to gain a better insight into the mechanistic details. The initial rate constants of the reaction were determined in situ ^1^H-nuclear magnetic resonance (NMR) and ^19^F-NMR spectroscopy. The results show that the reaction appeared to have a nearly first-order dependence on NHC catalyst **F** (Fig. 5a), and zero-order dependence on substrates **1a** (Figs. 5b), **2a** (Fig. 5c), and DQ (Fig. 5d).

**Fig. 6:**
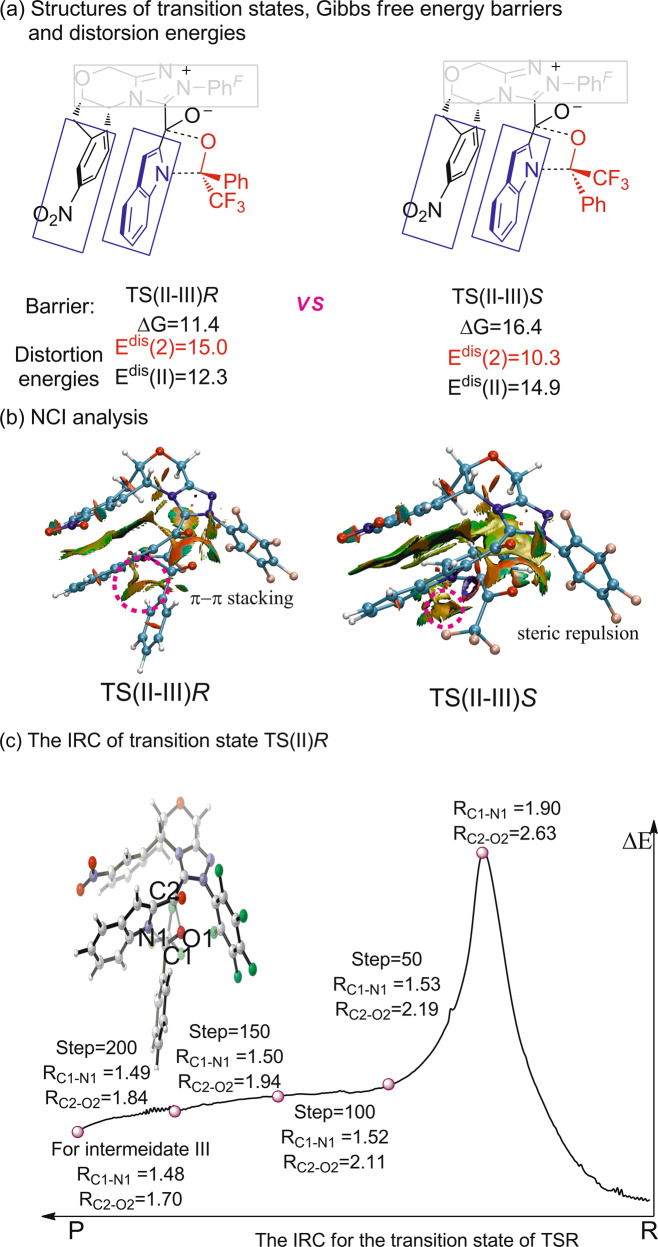
The DFT investigation on the enantioselectivity of the [10 + 2] annulation. **a** The two transition states of TS(II–III)*R* and TS(II–III)*S* Gibbs free energy barriers and distortion energies comparing. **b** NCI analysis of the TS(II–III)*R* and TS(II–III)*S*. **c** The IRC of transition state TS(II)*R*.

To further reveal the enantioselectivity of this [10 + 2] annulation, density functional theory (DFT) calculation was performed to study the key step of nucleophilic attack of intermediate **II** onto trifluoroacetophenone. As shown in Fig. 6a, two transition states named TS(II–III)*R* and TS(II–III)*S* was located, where the *re*- or *si*-face of trifluoroacetophenone was attacked, respectively. The calculated relative free energy of transition state TS(III–IV)*R* is 5.0 kcal/mol lower than that of TS(II–III)*S*, which predicts that the generation of *R*-configuration product **4a** is favorable. The calculated results overestimate the level of enantioinduction in this reaction process but are consistent with predicting the observed experimental product configuration. The geometry of those two transition states is also given in Fig. 6b. After the absorption of indole reactant onto the NHC catalyst, a strong π–π stacking between indolyl moiety and the aryl in the NHC catalyst can significantly stabilize the deprotonated indolyl moiety. The π–π attraction is clearly shown in calculated noncovalent interaction (NCI) maps. In addition, kinetic experiments revealed that electron-rich indoles or electron-deficient aryl ketones reacted more quickly, which partially elucidated the potential π−π interaction. When the nucleophilic attack occurs, trifluoromethyl of trifluoroacetophenone appears at the more bulky inner side in transition state TS(II–III)*R*. It is more favorable than the case in transition state TS(II–III)*S* that the phenyl group is set to the inner side. The NCI map of transition state TS(II–III)*R* clearly reveals that the repulsion between phenyl group of trifluoroacetophenone and the NHC catalyst leads to instability of transition state TS(III–IV)*S*, while this repulsion is absent in transition state TS(II–III)*R*.

In order to figure out whether the process from **II** to **III** would be concerted or stepwise, the intrinsic reaction coordinate calculation (IRC) of transition state TS(II)*R* has been performed (Fig. 6c). The result clearly shows the C1 of trifluoroacetophenone and N1 of indole would form the covalent bond firstly. Along with the decreasing distance of C1–N1, the bond of oxygen atom O1–C2 gradually formed until the intermediate **III** generate. Hence, we speculate that the process tends to be a concerted asynchronous process^59,69^.

### Synthetic transformations and applications

**Fig. 7:**
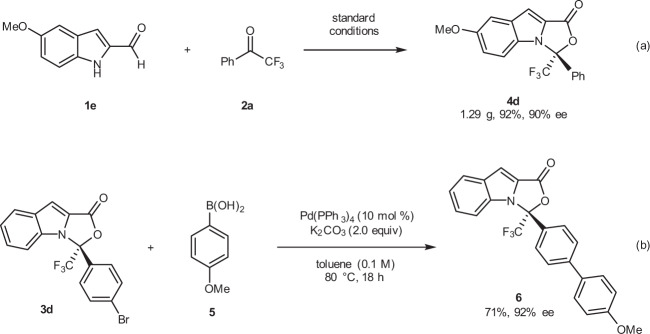
Gram scale synthesis and transformation. **a** Reaction conditions: **1e** (4.0 mmol), **2a** (4.8 mmol), **F** (0.60 mmol), DQ (4.4 mmol), additive **H** (0.20 mmol), PhCO_2_Na (4.0 mmol), Hexane (33.4 mL) and DCM (6.6 mL), room temperature, 4 Å M.S. (600 mg), Ar, 40 h. **b** Reaction conditions: **3d** (0.05 mmol), **5** (0.10 mmol), K_2_CO_3_ (0.10 mmol) and Pd(PPh_3_)_4_ (0.005 mmol), toluene (0.5 mL), 80 °C, 18 h.

Our protocol is amenable to large-scale preparation. For example, the use of standard conditions was sufficient to produce **4d** (1.29 g) in 92% yield and with 90% ee (Fig. 7a). A facile Pd-catalyzed Suzuki coupling of **3d** with 4-methoxyphenylboronic acid **5** led to product **6** in a 72% yield and with a remained enantioselectivity (Fig. 7b).

In summary, a unique NHC-catalyzed enantioselective hetero-[10 + 2] annulation of indole-2-carbaldehydes with trifluoromethyl ketone derivatives has been developed. This process generates a new NHC-bounded aza-benzofulvene as a key intermediate. This new protocol allows the rapid assembly of enantioenriched polycycles from readily available starting materials under mild conditions. DFT calculations elucidated the origins of the [10 + 2] process. Further investigations on new NHC-bounded aza-arylfulvene as an active intermediate in asymmetric synthesis are currently ongoing in our laboratory.

## Methods

### Synthesis of 3/4

To a flame-dried Schlenk reaction tube equipped with a magnetic stir bar, was added the precatalyst **F** (15.4 mg, 0.03 mmol), DQ (90.0 mg, 0.22 mmol), additive **H** (5.0 mg, 0.01 mmol), PhCO_2_Na (28.8 mg, 0.20 mmol), **1** (0.20 mmol) and 4 Å MS (60 mg). The Schlenk tube was closed with a septum, evacuated, and refilled with an argon atmosphere. Hexane (2.0 mL) and **2** (0.24 mmol) was added. The mixture was then stirred at 25 °C and monitored by TLC until **1** was consumed. The mixture was concentrated under reduced pressure and purified by column chromatography on silica gel (hexane/EtOAc = 100:1) to afford the desired product **3** or **4**. Full experimental details can be found in the Supplementary Methods.

## Supplementary information

Supplementary Information

Description of Additional Supplementary Files

Supplementary Data 1

Supplementary Data 2

## Data Availability

For ^1^H NMR, ^13^C NMR, and ^19^F NMR spectra see Supplementary Figs. 1–93 and high-performance liquid chromatography spectra see Supplementary Figs. 94–153. The supplementary crystallographic data (Supplementary Data 1) for this paper could be obtained free of charge from The Cambridge Crystallographic Data Centre (**3e:** CCDC 1961662) via www.ccdc.cam.ac.uk/data_request/cif. The coordinates for the corresponding structures and IRC of transition state TS(II)R in Supplementary Data 2.
