# Supplementary material for: N-Heterocyclic carbene-catalyzed enantioselective hetero-[10 + 2] annulation
Source: Commun Chem. 2020 Nov 27;3:177. doi: 10.1038/s42004-020-00425-7 (PMC9814252; doi:10.1038/s42004-020-00425-7)
Supplement: Supplementary file 1 — Supplementary Information [file 42004_2020_425_MOESM1_ESM.pdf]

Supporting Information

N-Heterocyclic carbene-catalyzed enantioselective hetero-[10+2] annulation

Qiupeng Peng, Shi-Jun Li, Bei Zhang, Donghui Guo, Yu Lan* and Jian Wang*

Contents

NMR Spectra 2

HPLC Spectra 64

General Information 94

Procedure for Synthesis of Catalyst **F** 94

General Procedure for Catalytic Reaction of Indole-2-carbaldehydes with Ketones 95

Characterization Data 95

Gram Scale Synthesis Procedure 110

Synthetic Transformation 111

HRMS of **II** 111

Kinetic Experiment 112

Control Experiment 116

Crystal Structure of **3e** 118

Computational Details 119

Supplementary References 121

NMR Spectra

3a (*R*)-3-phenyl-3-(trifluoromethyl)-1*H*,3*H*-oxazolo[3,4-a]indol-1-one

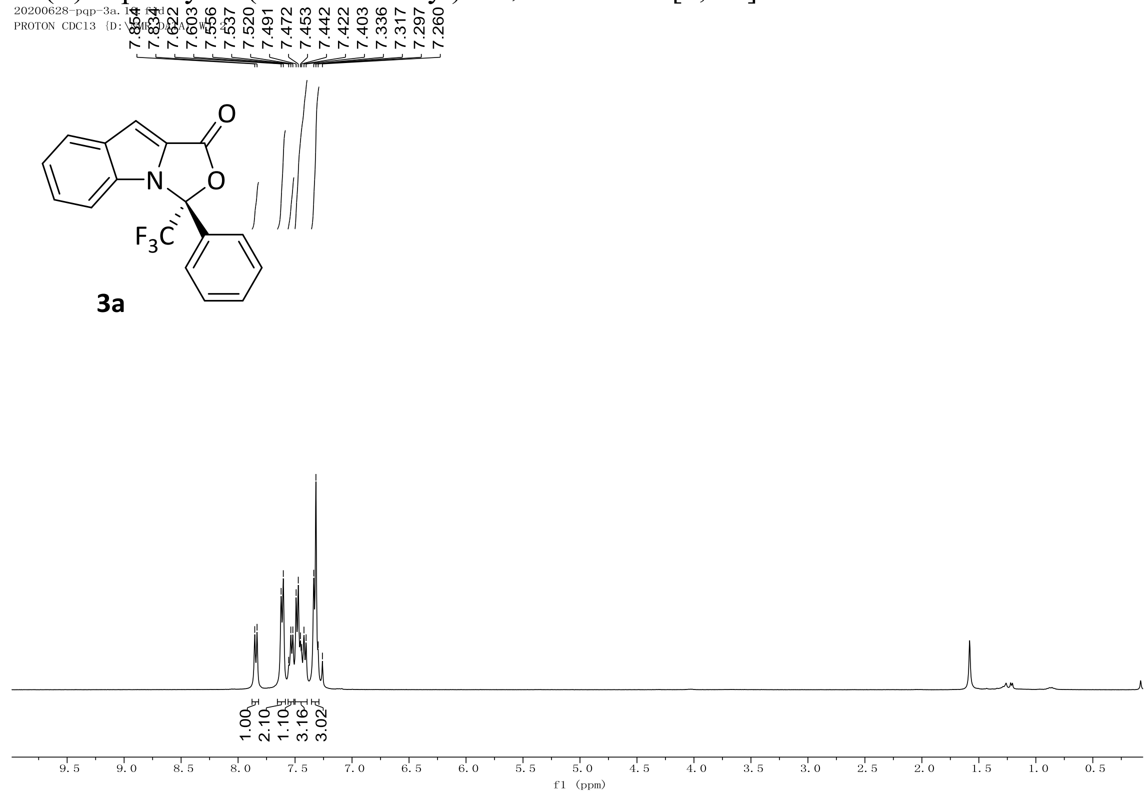

Supplementary Figure 1. ^{1}H NMR Spectra of **3a**

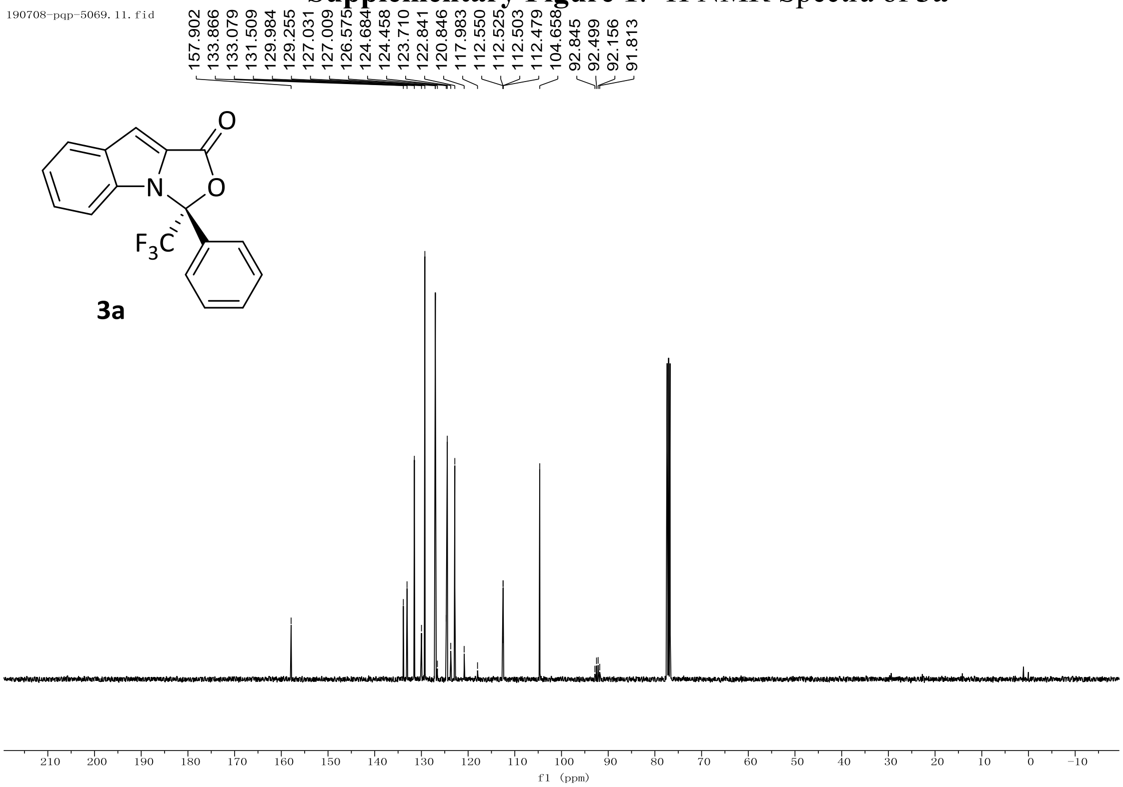

Supplementary Figure 2. ^{13}C NMR Spectra of **3a**

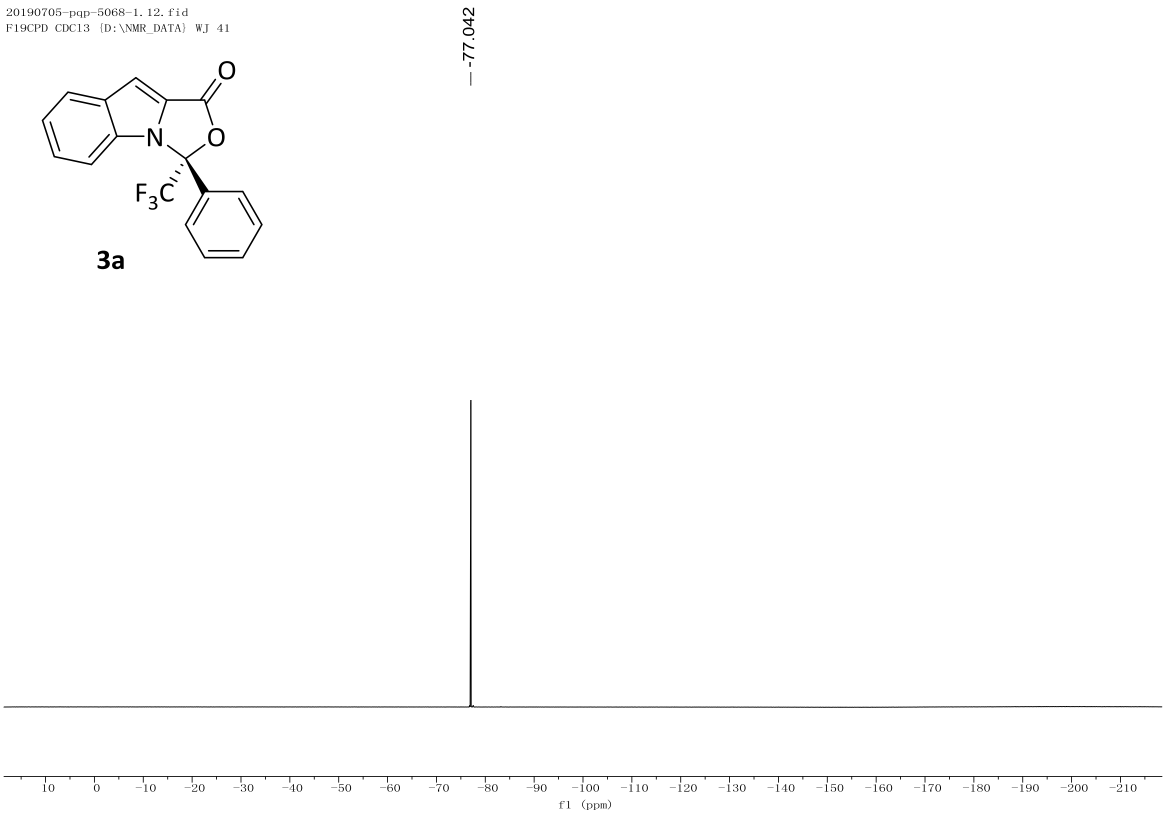

Supplementary Figure 3. ^{19}F NMR Spectra of **3a**

3b (*R*)-3-(4-fluorophenyl)-3-(trifluoromethyl)-1*H*,3*H*-oxazolo[3,4-a]indol-1-one

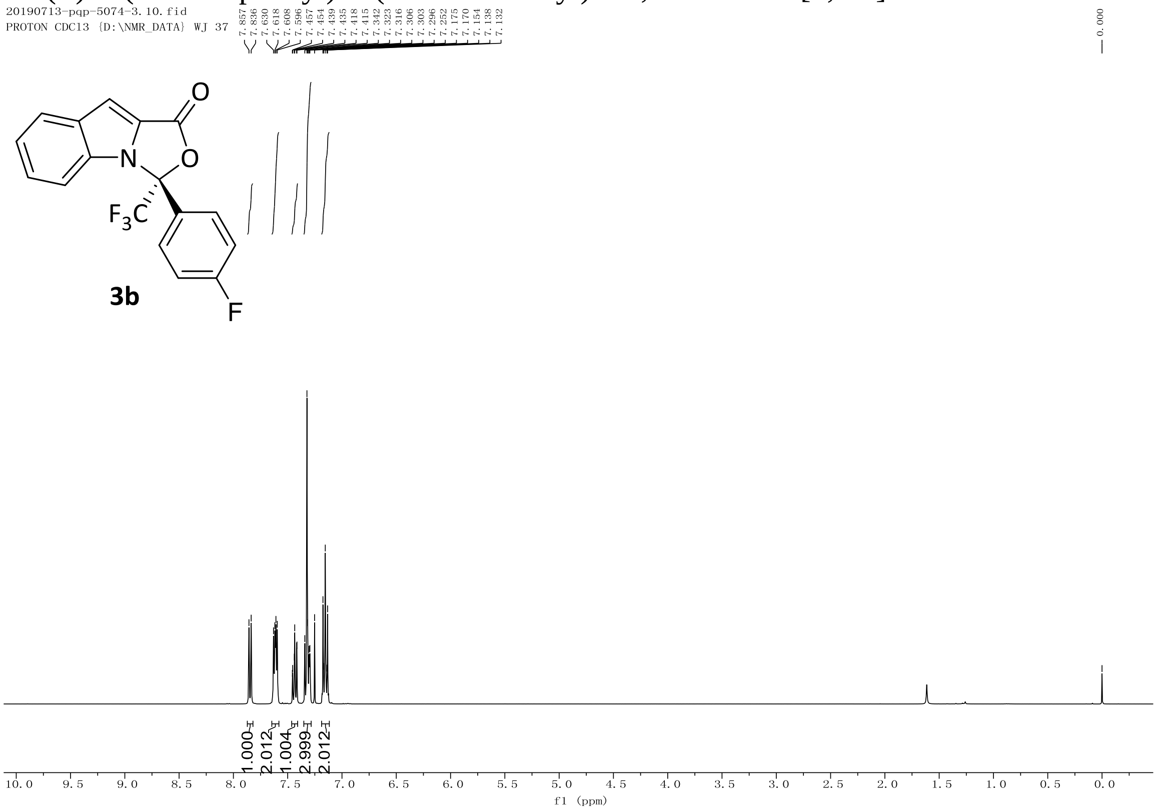

Supplementary Figure 4. ^{1}H NMR Spectra of **3b**

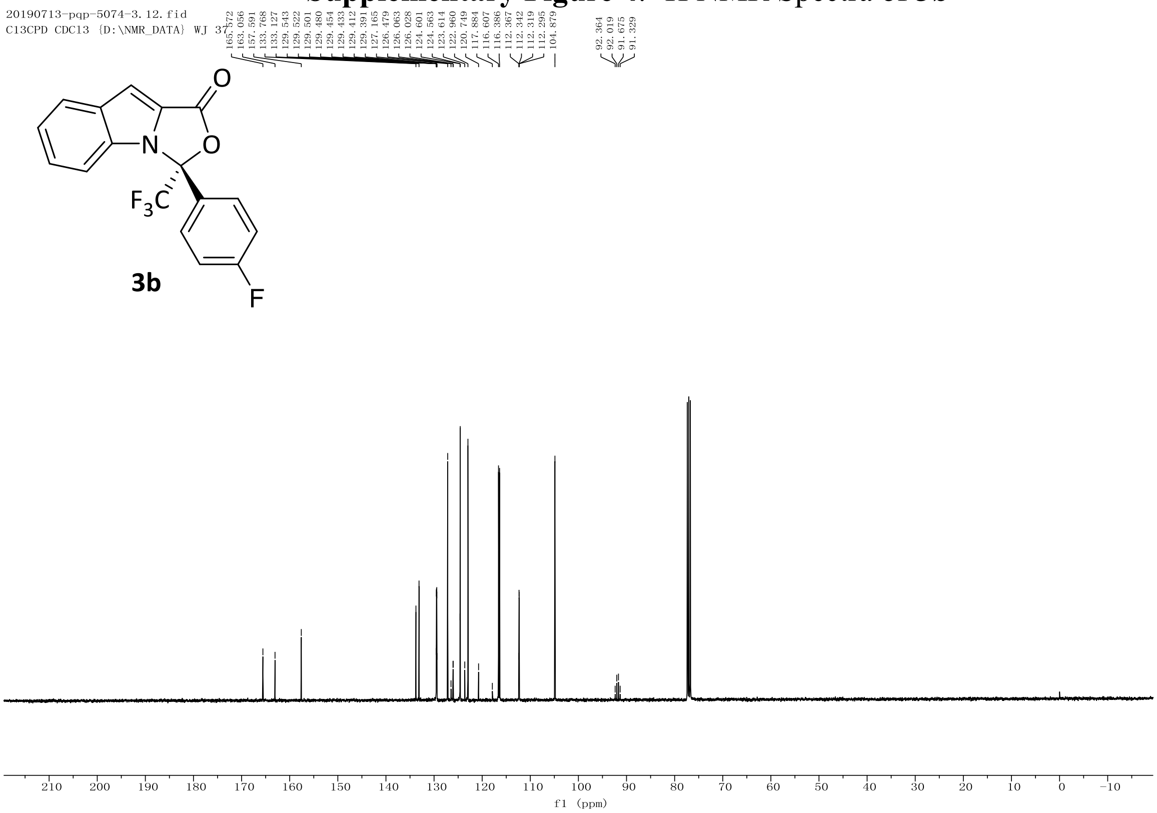

Supplementary Figure 5. ^{13}C NMR Spectra of **3b**

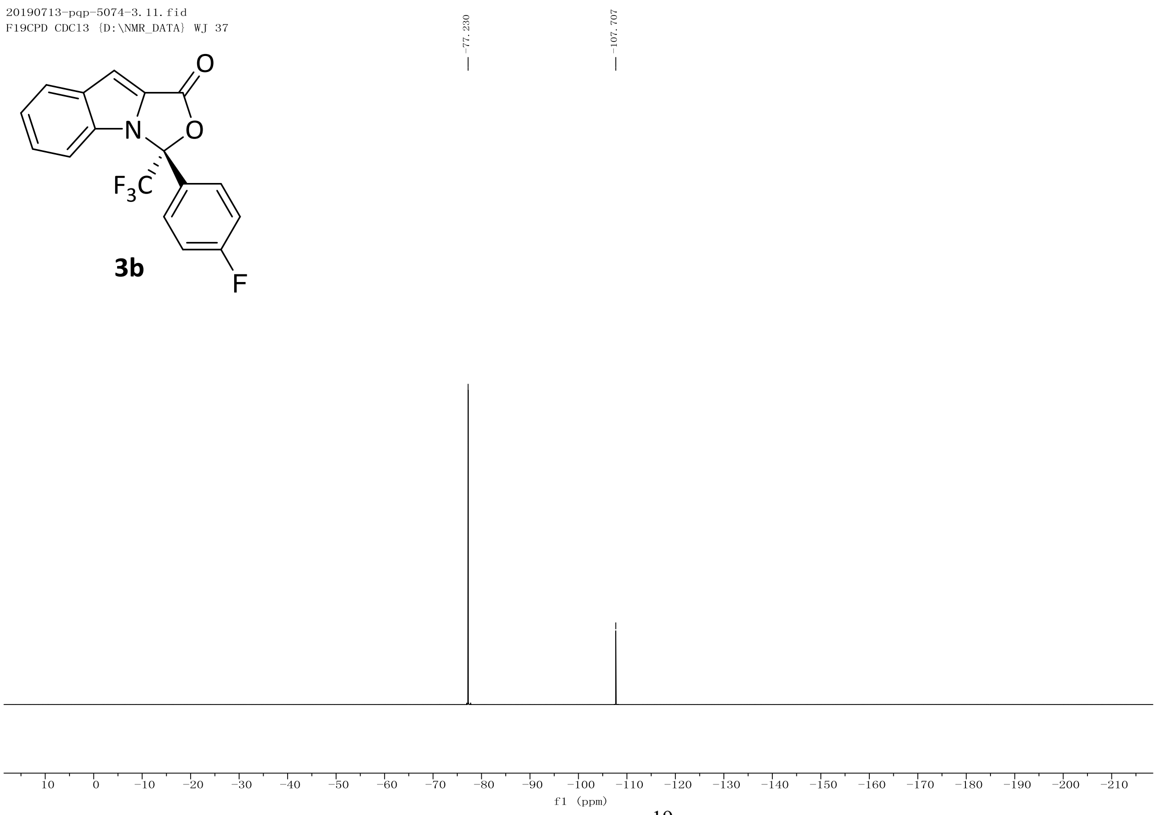

Supplementary Figure 6. ^{19}F NMR Spectra of **3b**

3c (*R*)-3-(4-chlorophenyl)-3-(trifluoromethyl)-1*H*,3*H*-oxazolo[3,4-a]indol-1-one

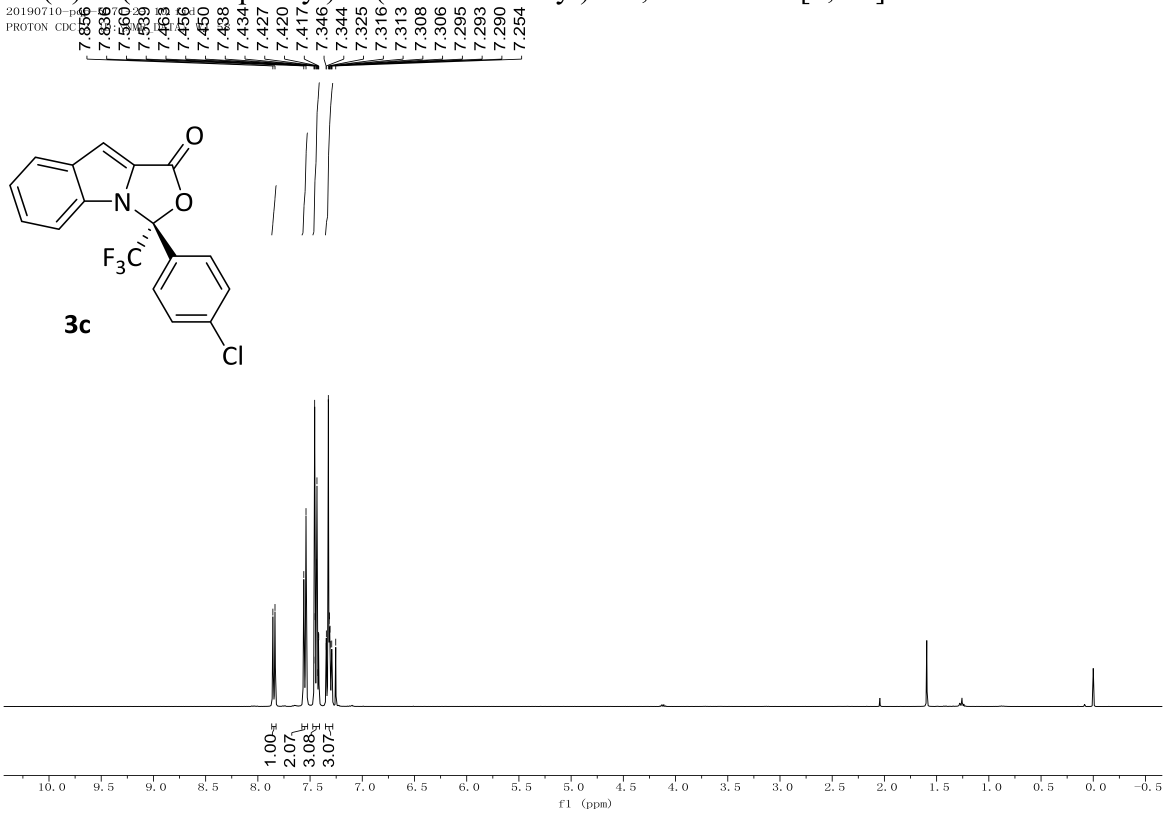

Supplementary Figure 7. ^{1}H NMR Spectra of **3c**

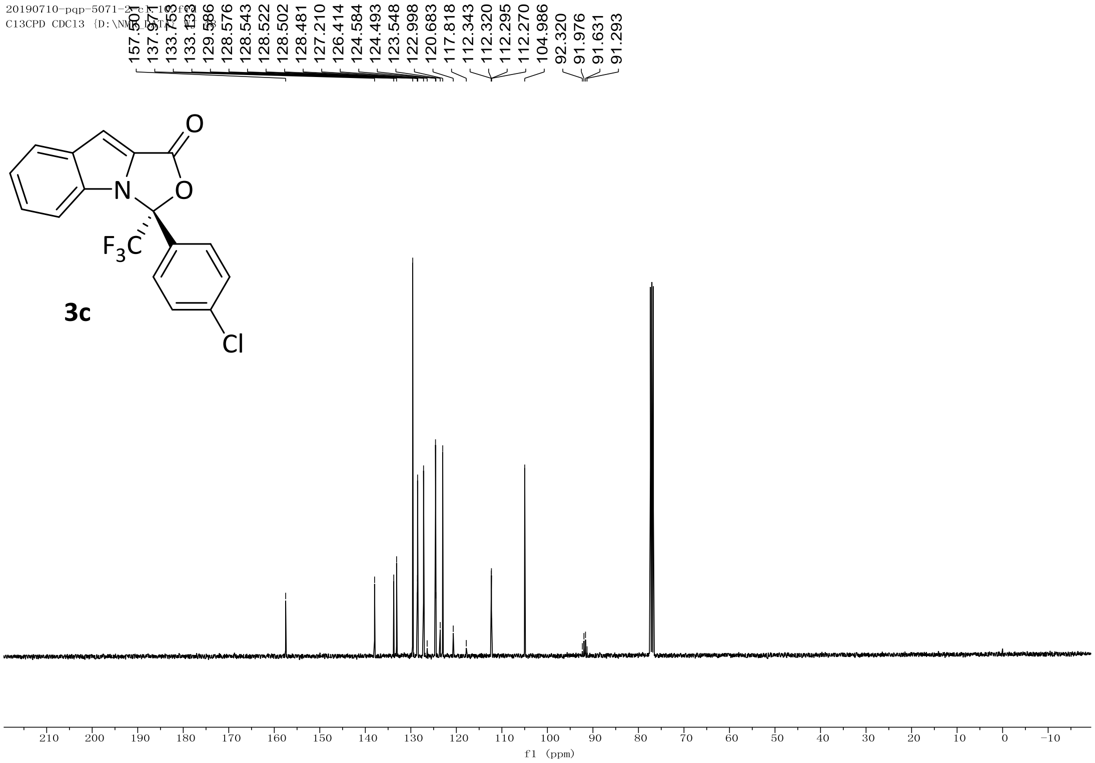

Supplementary Figure 8. ^{13}C NMR Spectra of **3c**

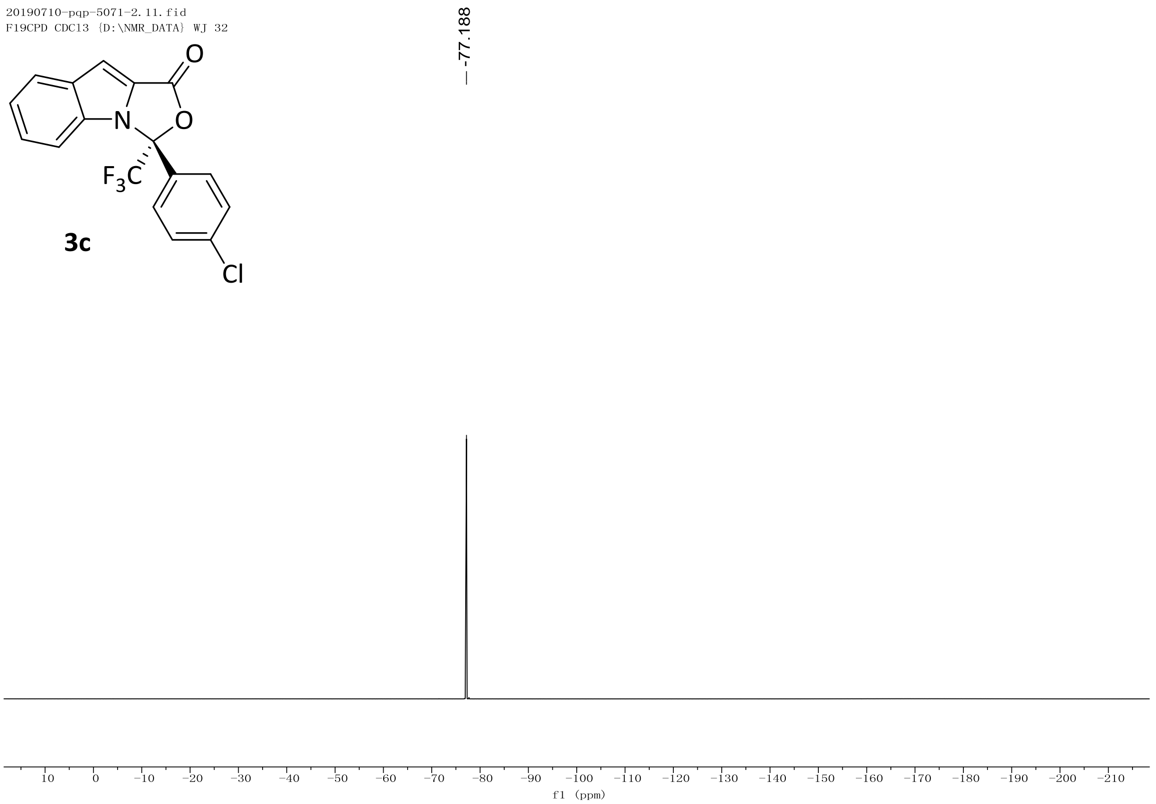

Supplementary Figure 9. ^{19}F NMR Spectra of **3c**

3d (*R*)-3-(4-bromophenyl)-3-(trifluoromethyl)-1*H*,3*H*-oxazolo[3,4-a]indol-1-one

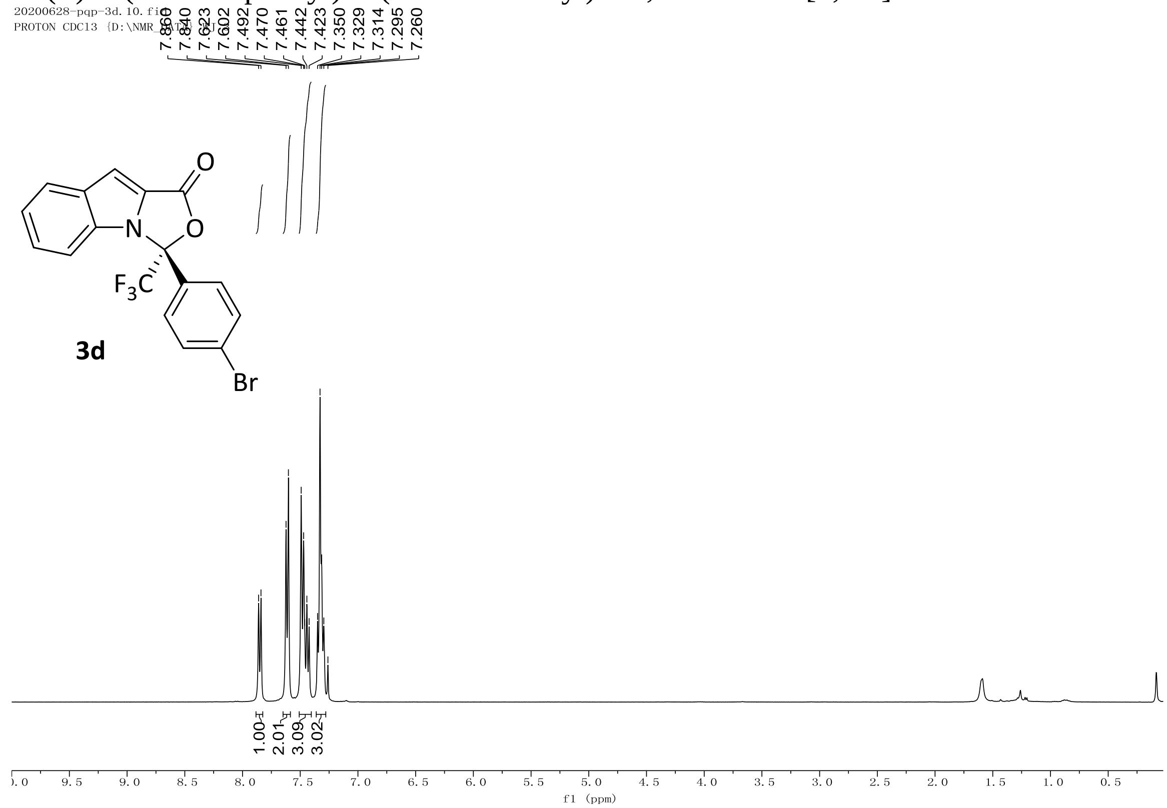

Supplementary Figure 10. ^{1}H NMR Spectra of **3d**

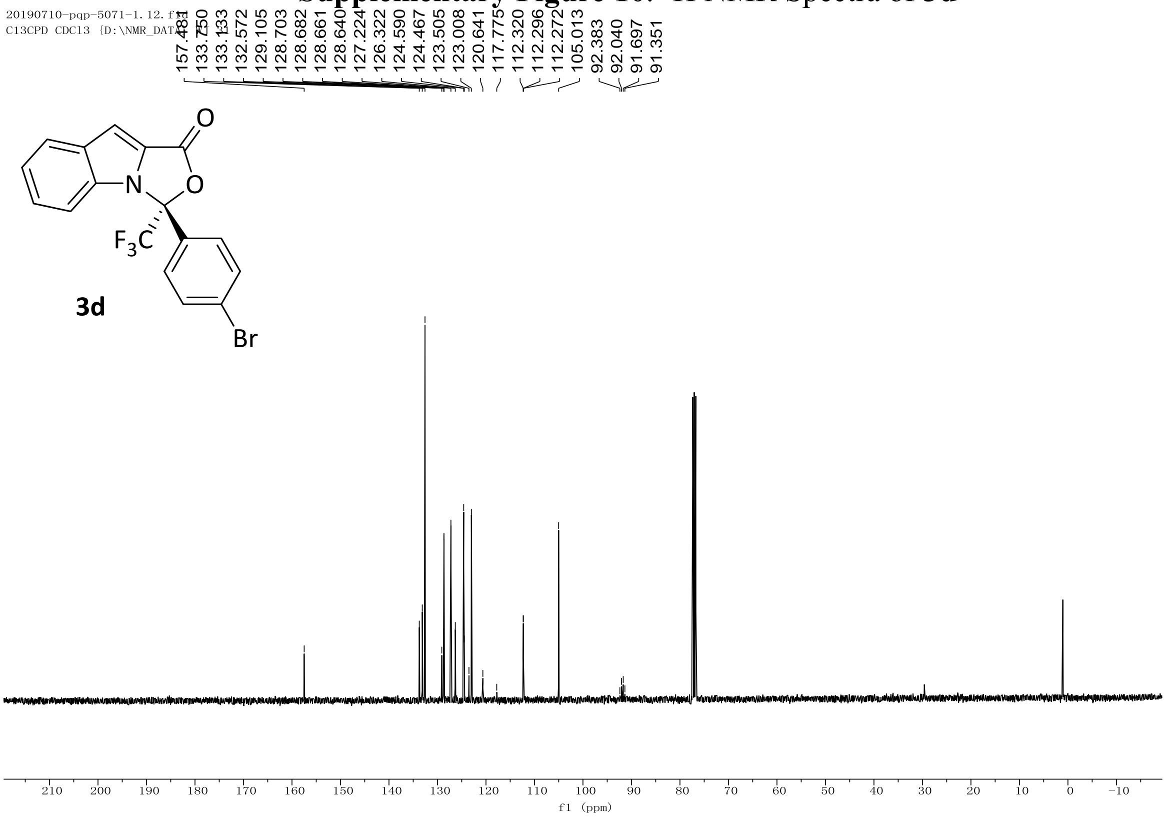

Supplementary Figure 11. ^{13}C NMR Spectra of **3d**

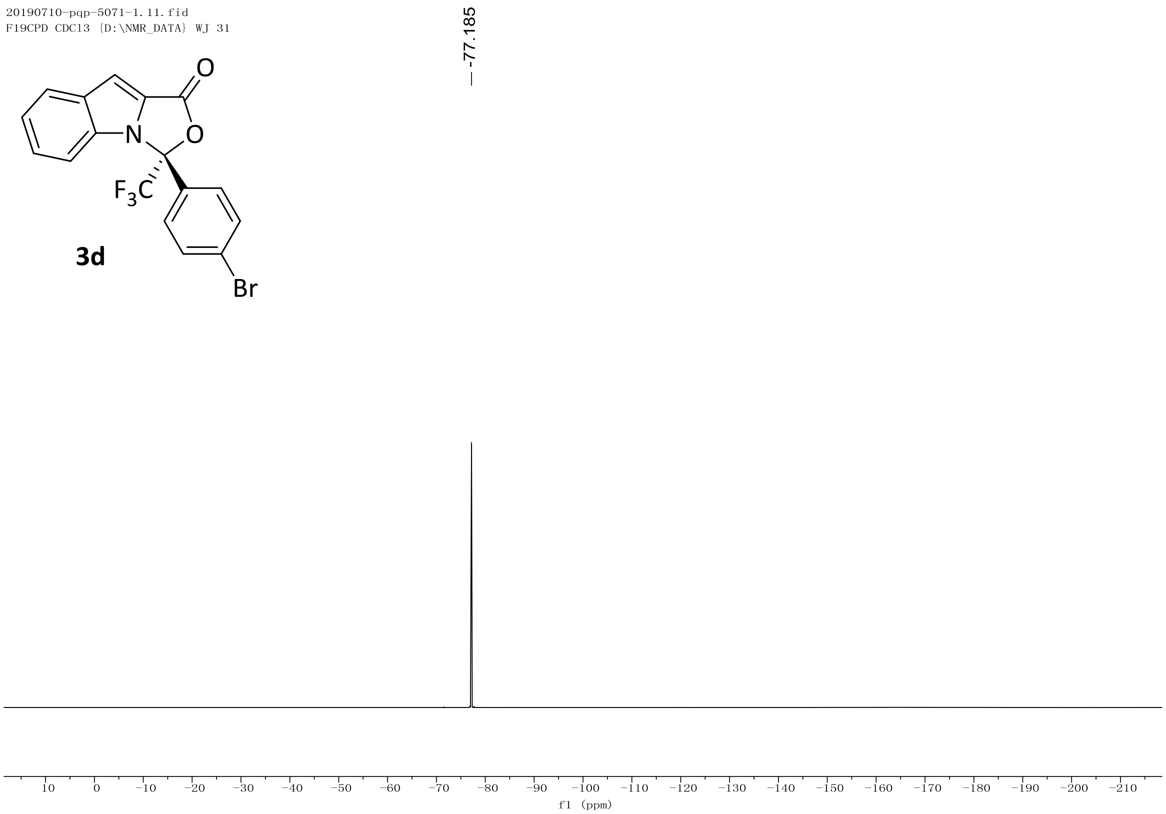

Supplementary Figure 12. ^{19}F NMR Spectra of **3d**

3e (*R*)-3-(p-tolyl)-3-(trifluoromethyl)-1*H*,3*H*-oxazolo[3,4-a]indol-1-one

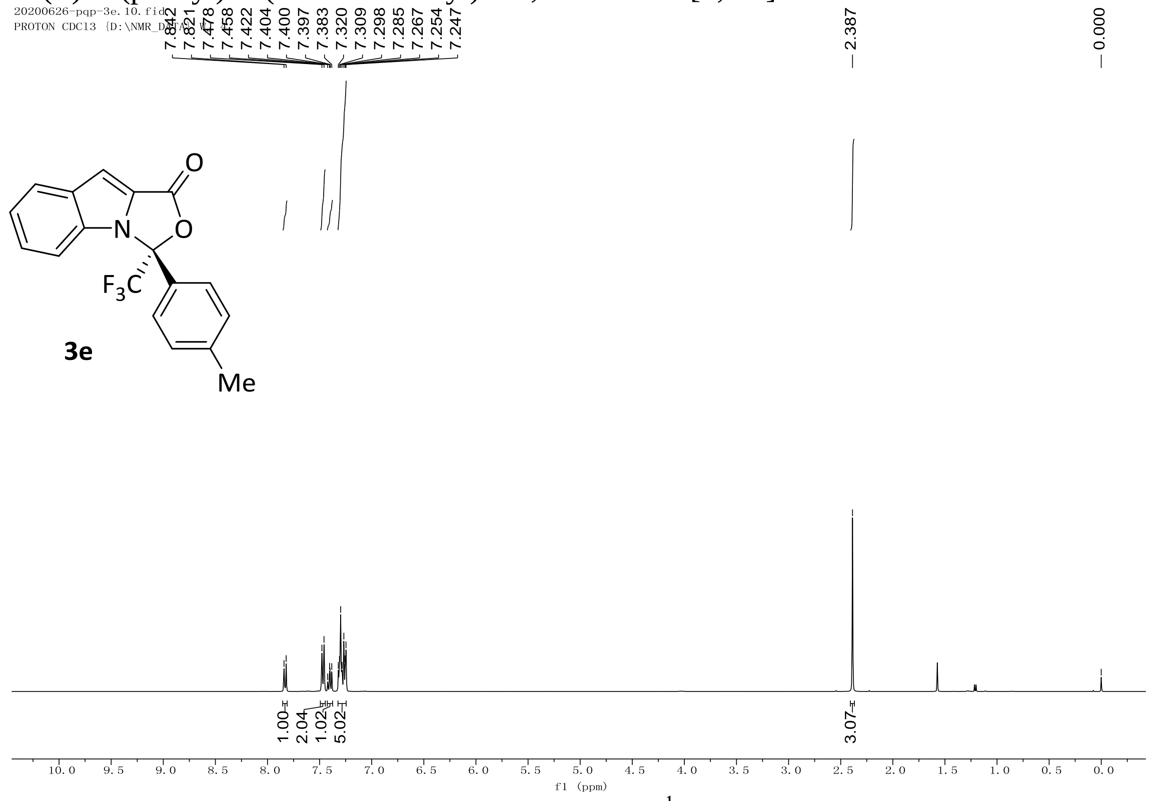

Supplementary Figure 13. ^{1}H NMR Spectra of **3e**

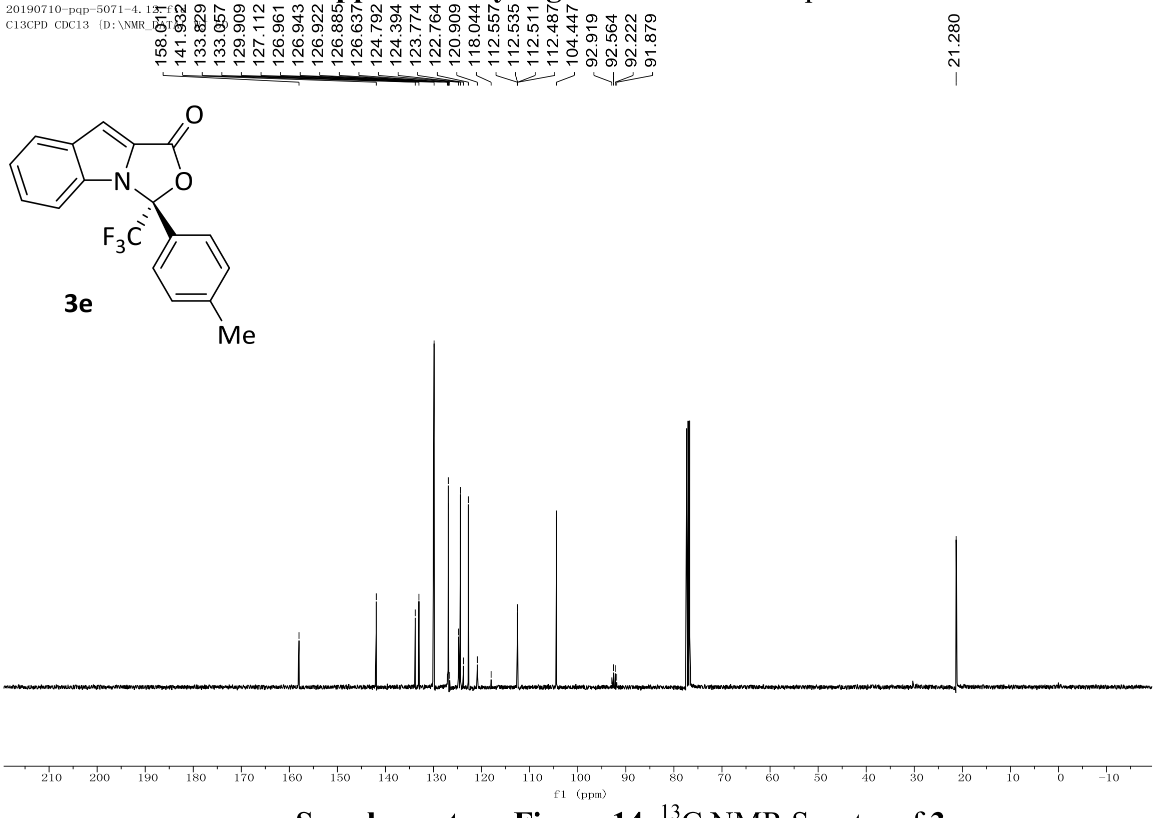

Supplementary Figure 14. ^{13}C NMR Spectra of **3e**

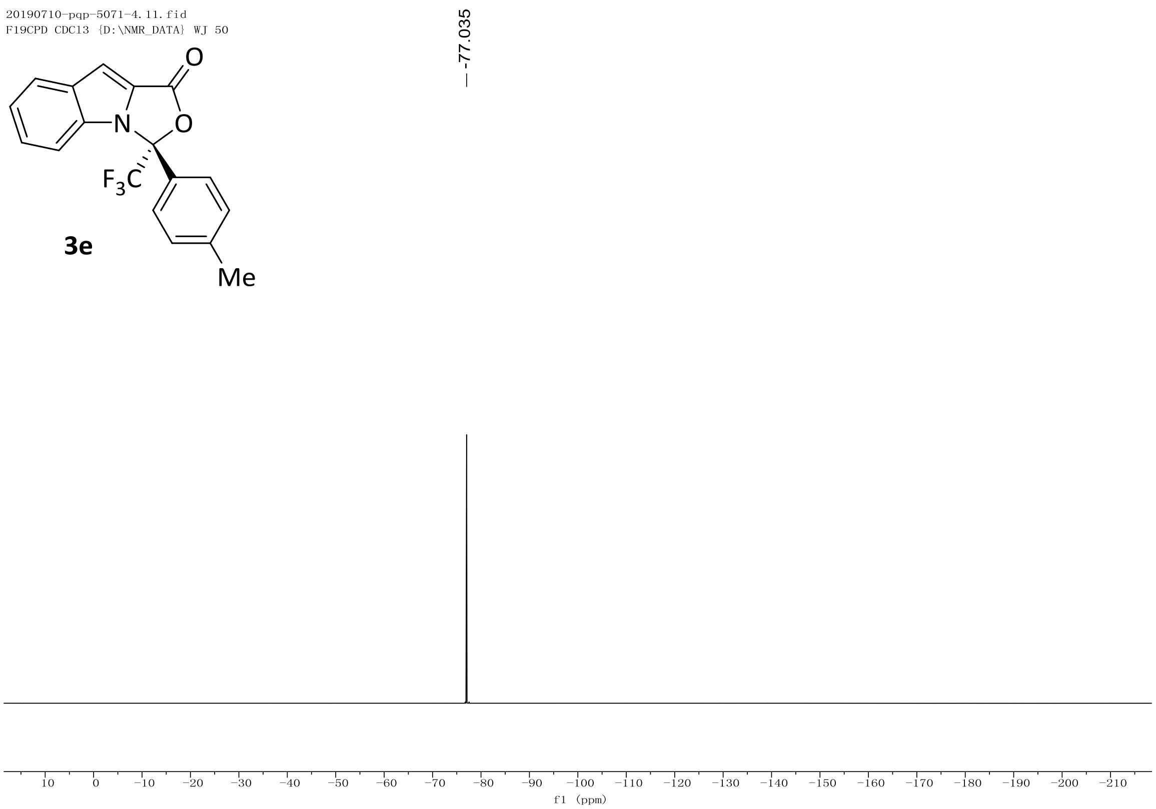

Supplementary Figure 15. ^{19}F NMR Spectra of **3e**

3f (*R*)-3-(4-methoxyphenyl)-3-(trifluoromethyl)-1*H*,3*H*-oxazolo[3,4-a]indol-1-one

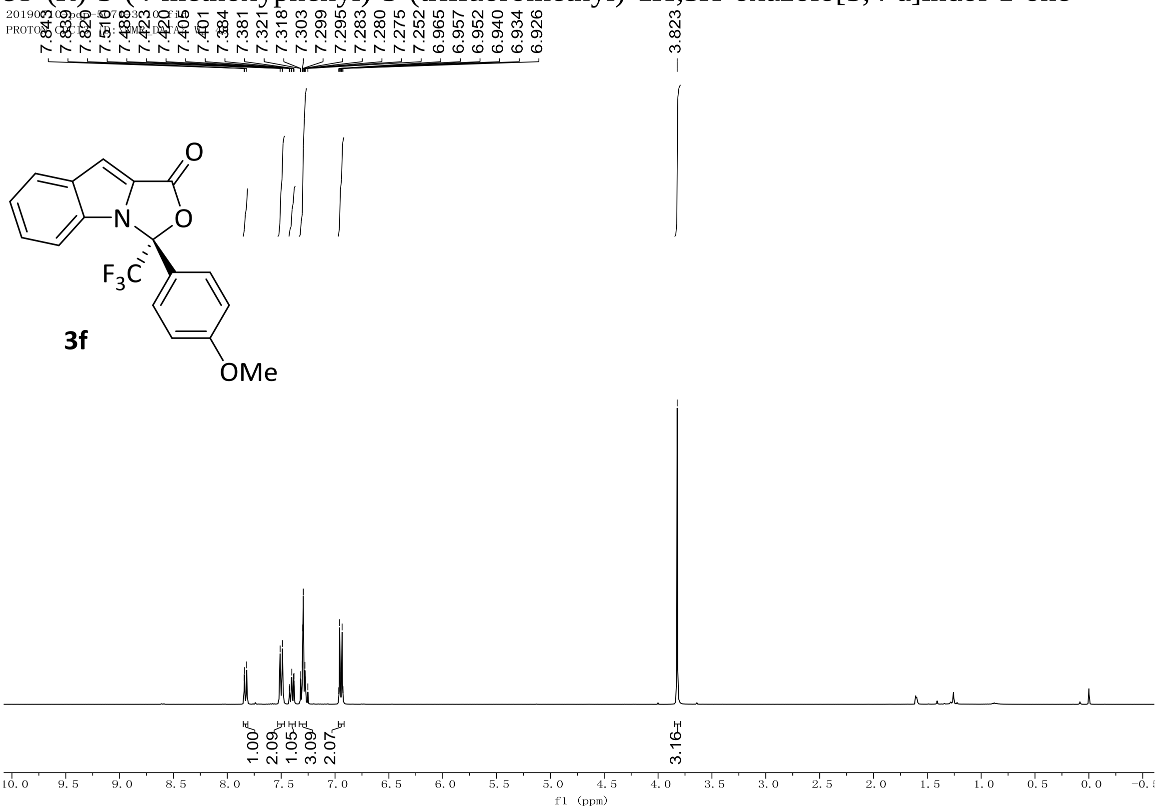

Supplementary Figure 16. ^{1}H NMR Spectra of **3f**

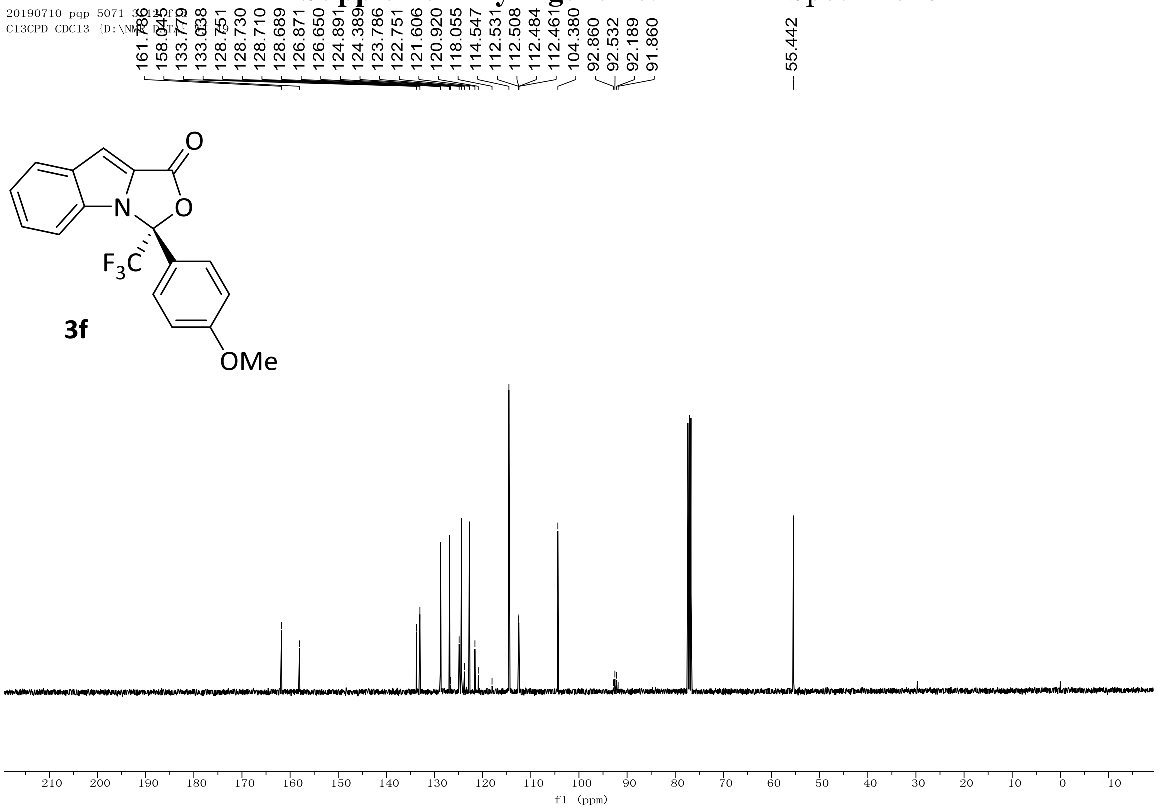

Supplementary Figure 17. ^{13}C NMR Spectra of **3f**

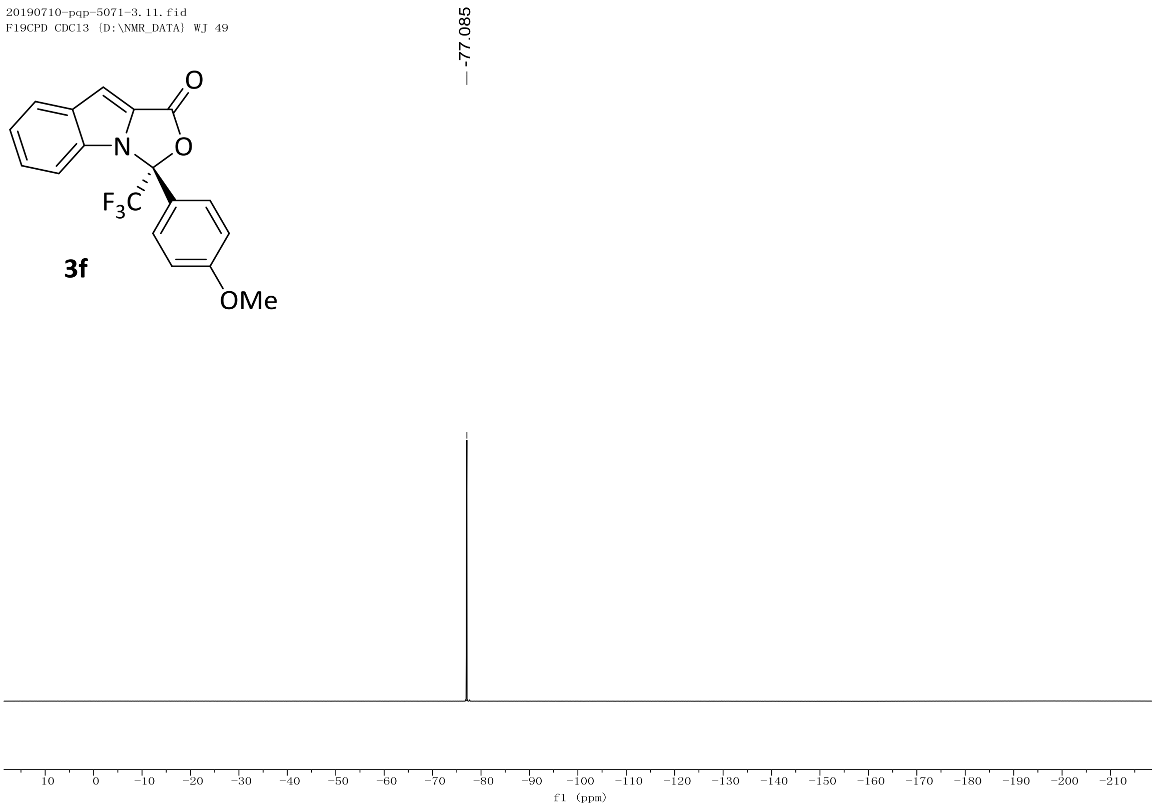

Supplementary Figure 18. ^{19}F NMR Spectra of **3f**

3g (*R*)-3-([1,1'-biphenyl]-4-yl)-3-(trifluoromethyl)-1*H*,3*H*-oxazolo[3,4-a]indol-1-one

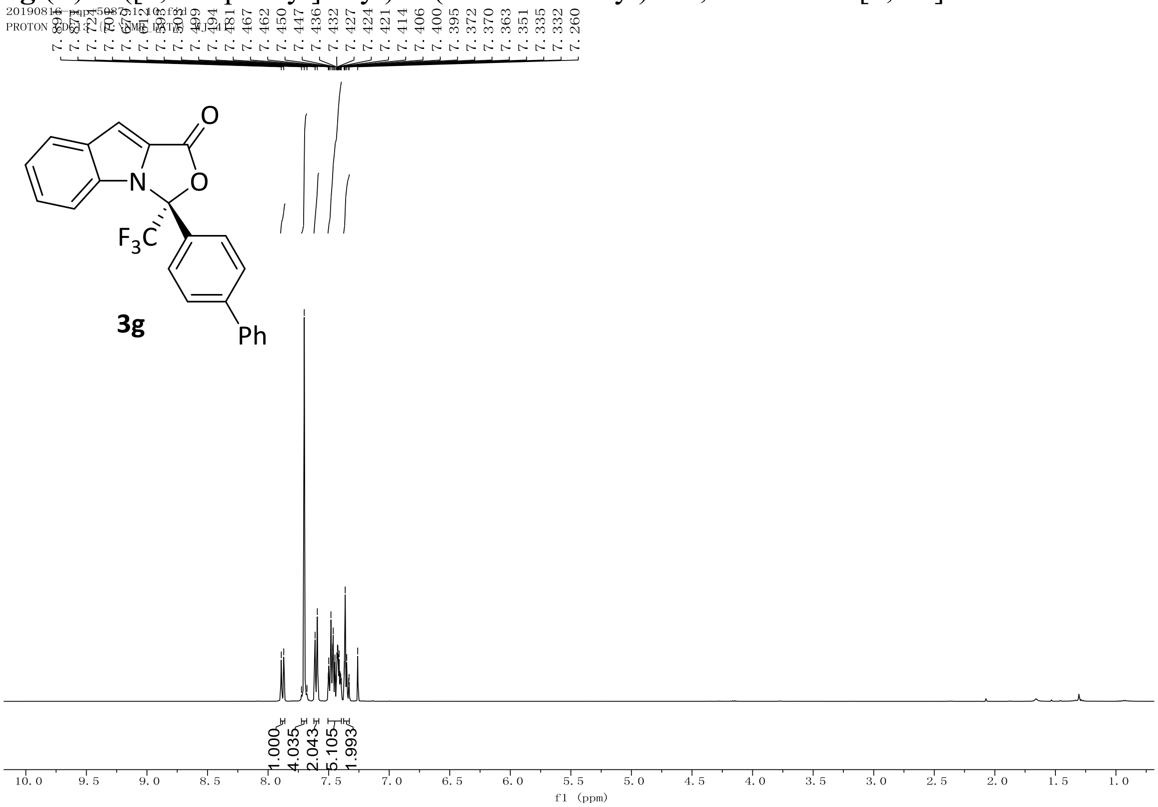

Supplementary Figure 19. ^{1}H NMR Spectra of **3g**

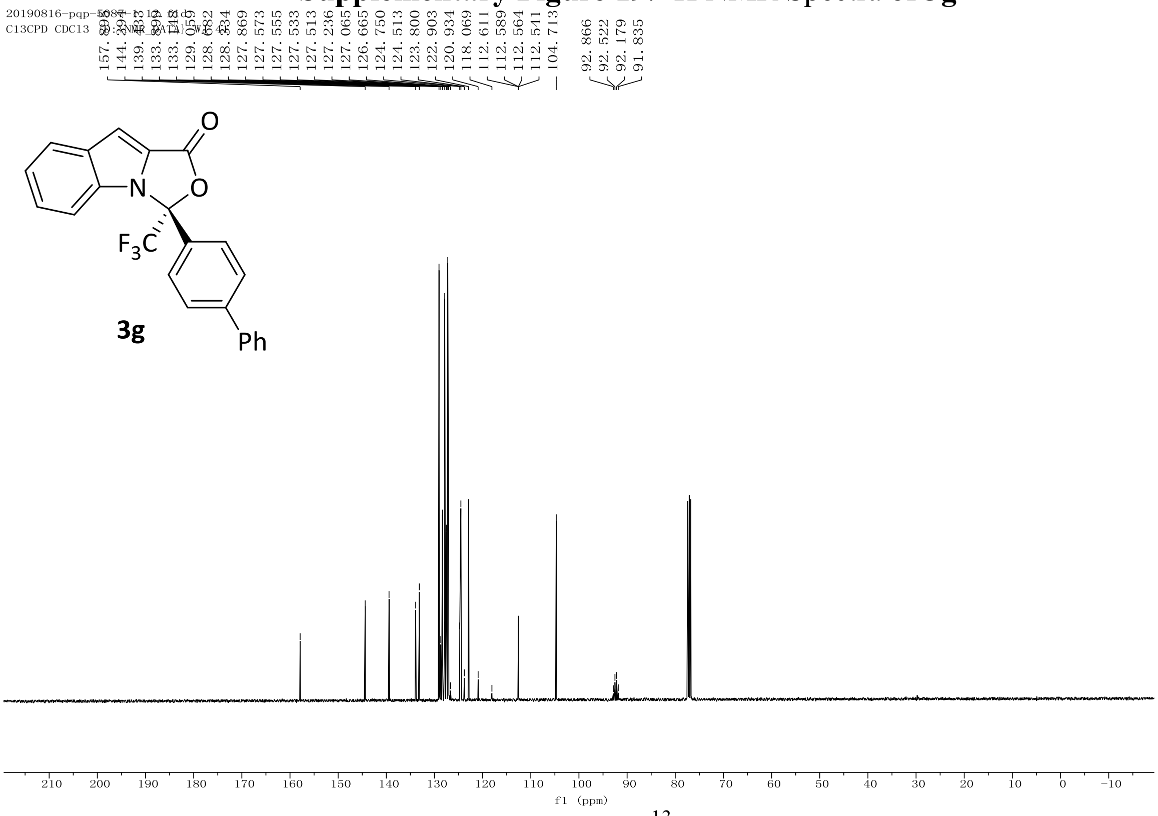

Supplementary Figure 20. ^{13}C NMR Spectra of **3g**

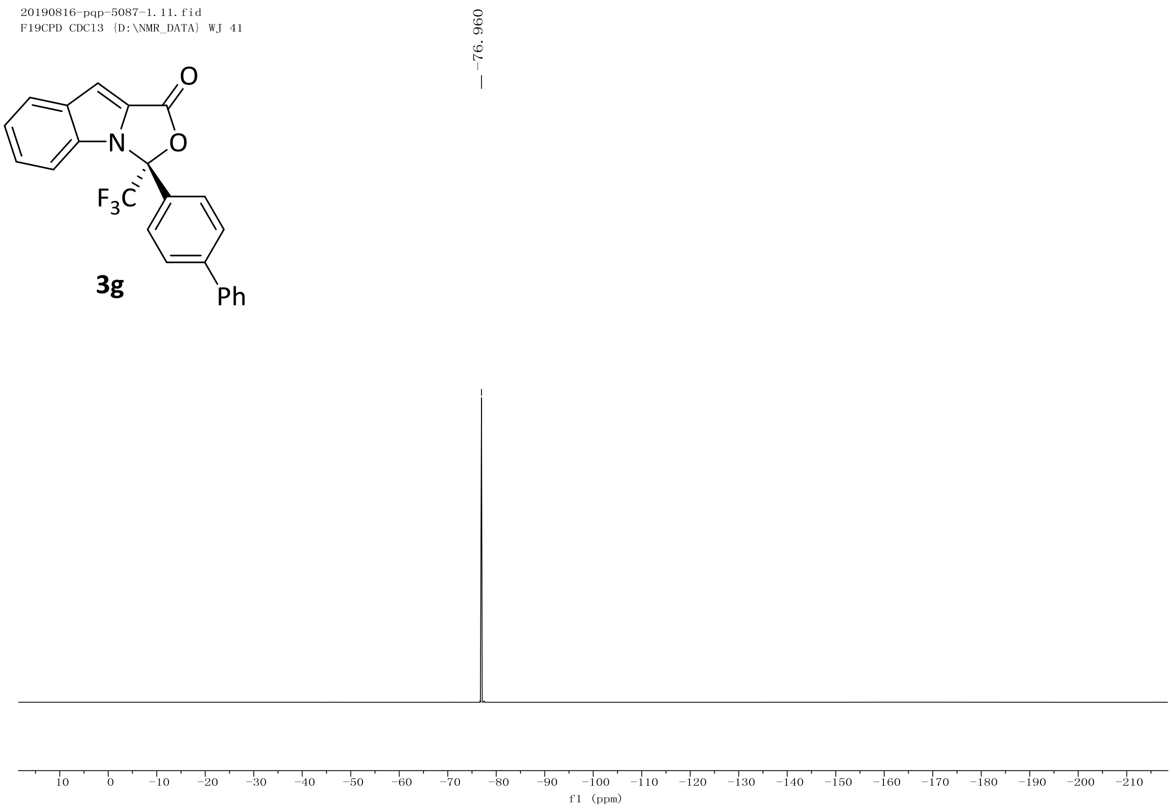

Supplementary Figure 21. ^{19}F NMR Spectra of **3g**

3h (*R*)-3-(3-fluorophenyl)-3-(trifluoromethyl)-1*H*,3*H*-oxazolo[3,4-a]indol-1-one

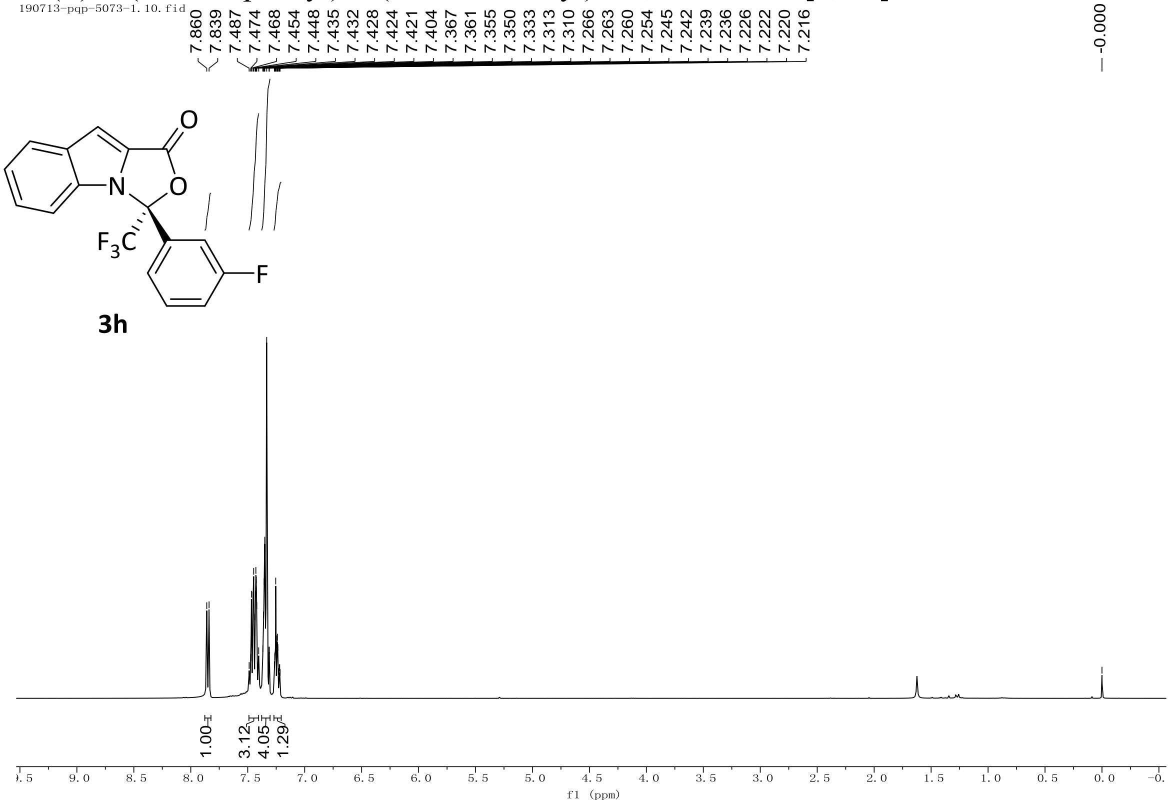

Supplementary Figure 22. ^{1}H NMR Spectra of **3h**

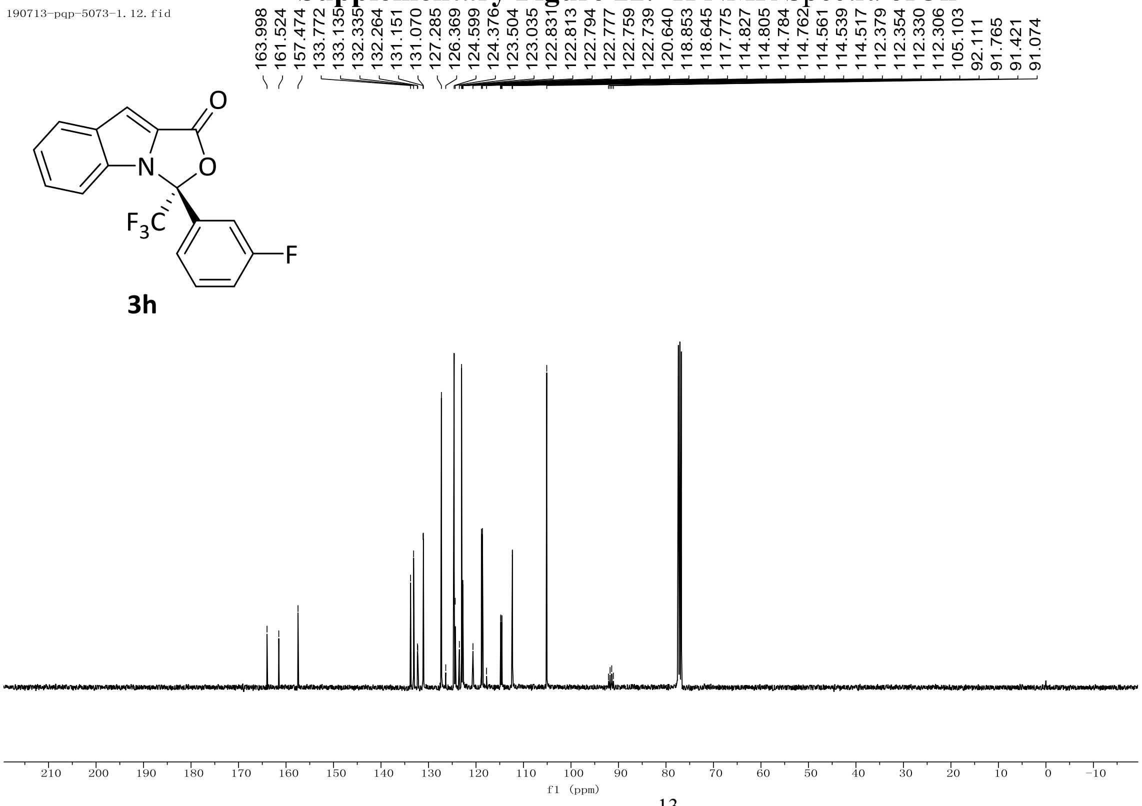

Supplementary Figure 23. ^{13}C NMR Spectra of **3h**

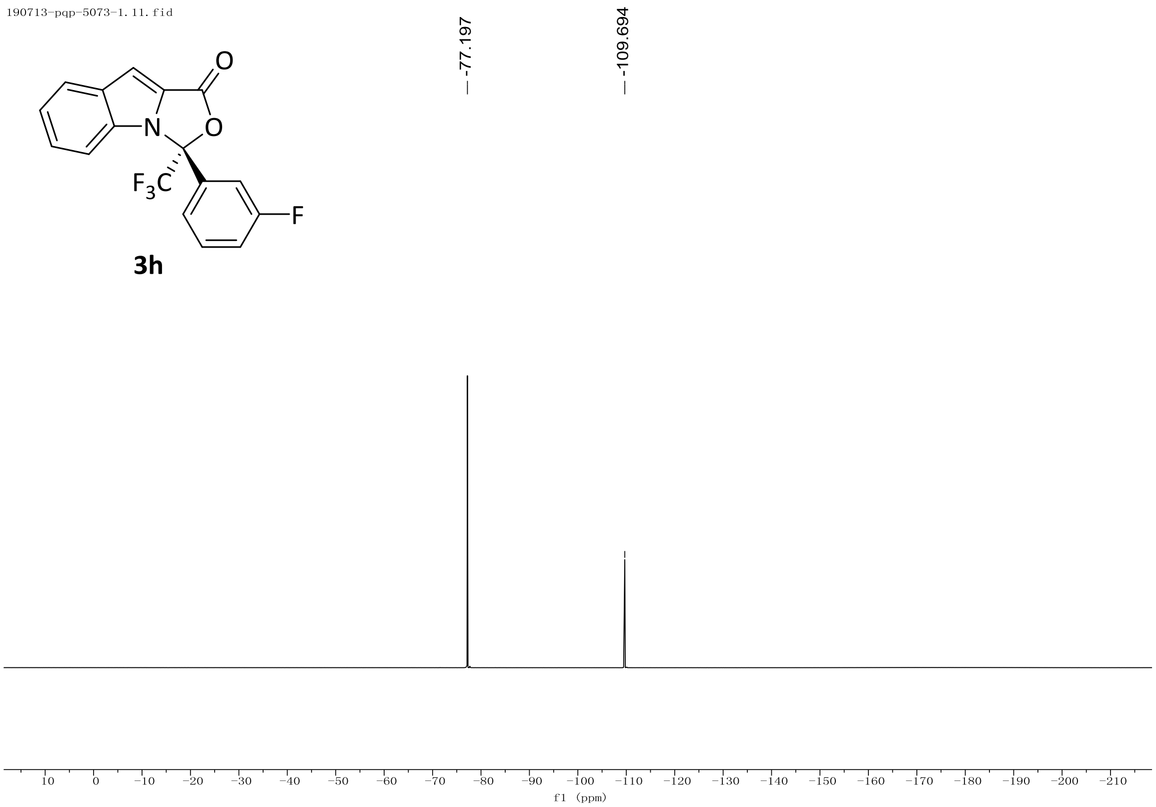

Supplementary Figure 24. ^{19}F NMR Spectra of **3h**

3i (*R*)-3-(naphthalen-2-yl)-3-(trifluoromethyl)-1*H*,3*H*-oxazolo[3,4-a]indol-1-one

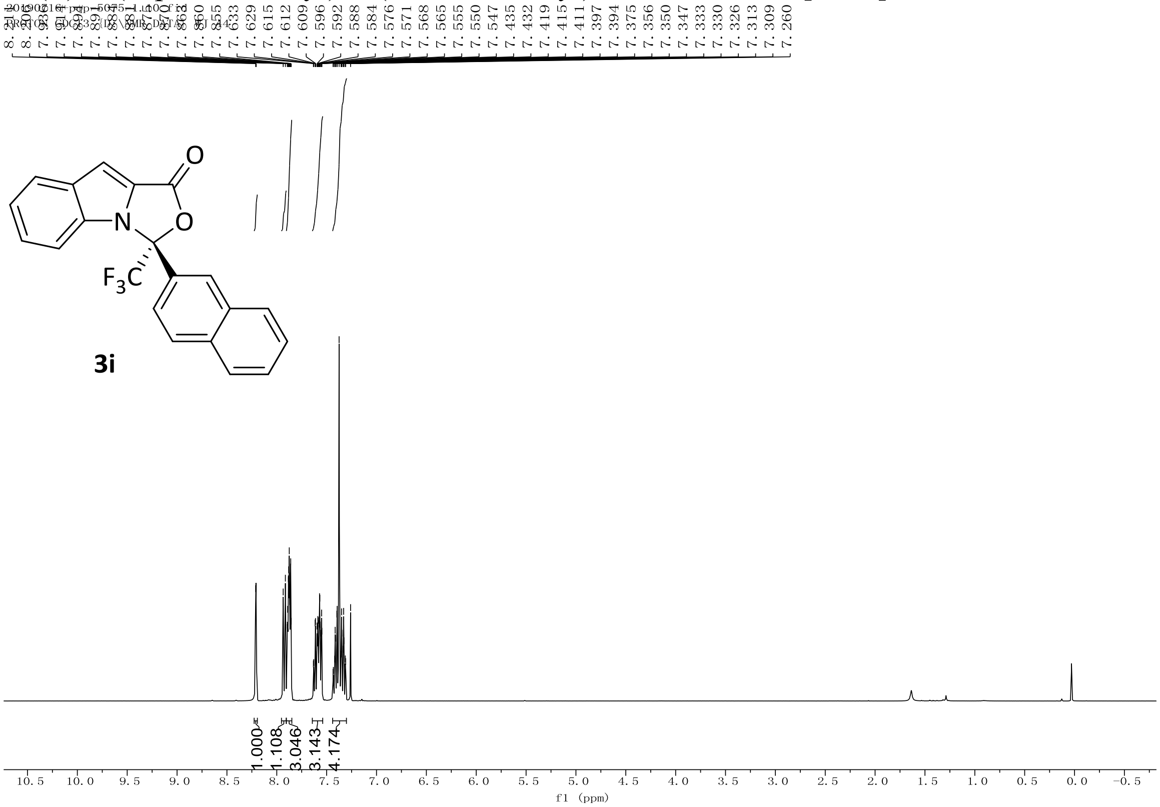

Supplementary Figure 25. ^{1}H NMR Spectra of **3i**

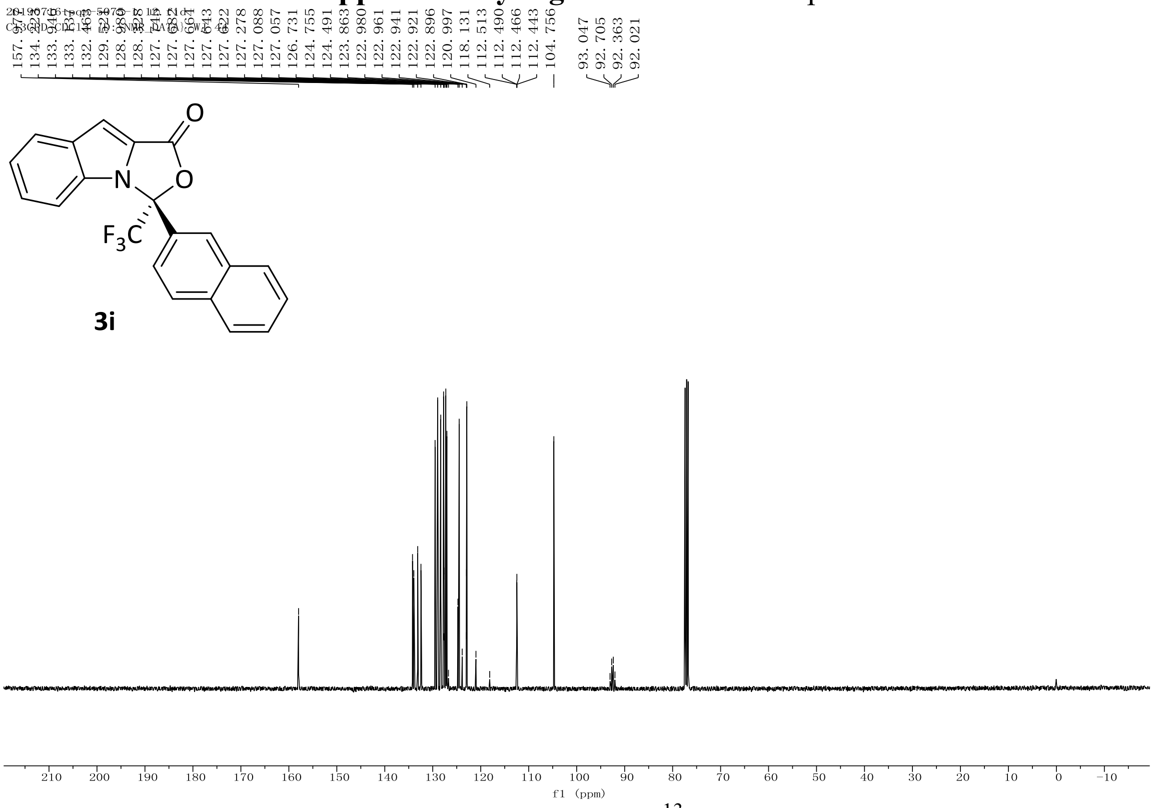

Supplementary Figure 26. ^{13}C NMR Spectra of **3i**

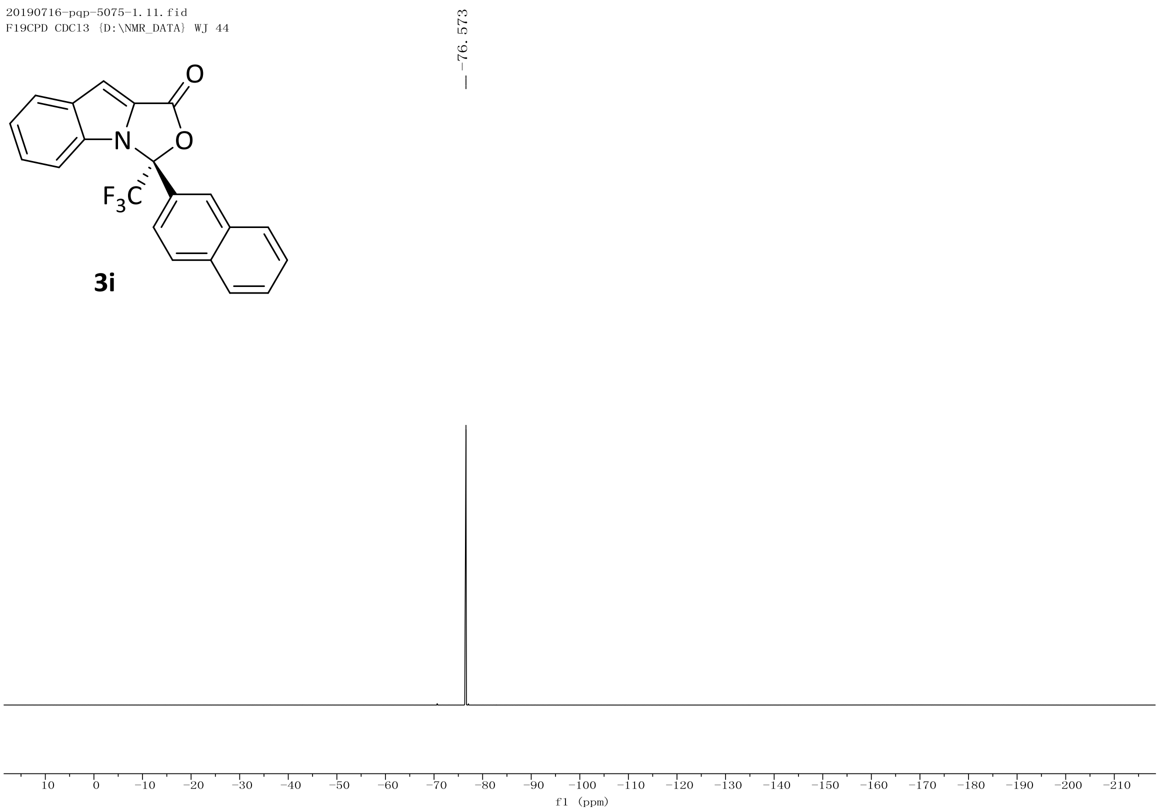

Supplementary Figure 27. ^{19}F NMR Spectra of **3i**

3j (*S*)-3-(benzo[d]thiazol-2-yl)-3-(trifluoromethyl)-1*H*,3*H*-oxazolo[3,4-a]indol-1-one

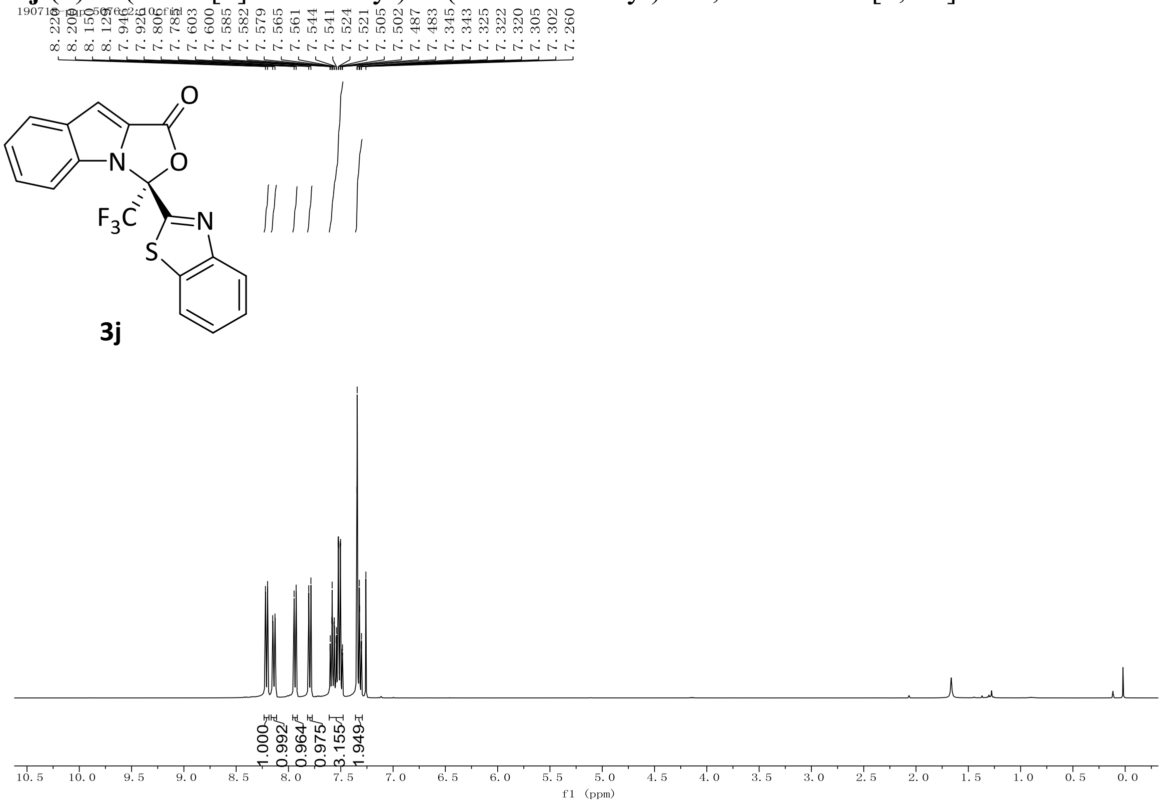

Supplementary Figure 28. ^{1}H NMR Spectra of **3j**

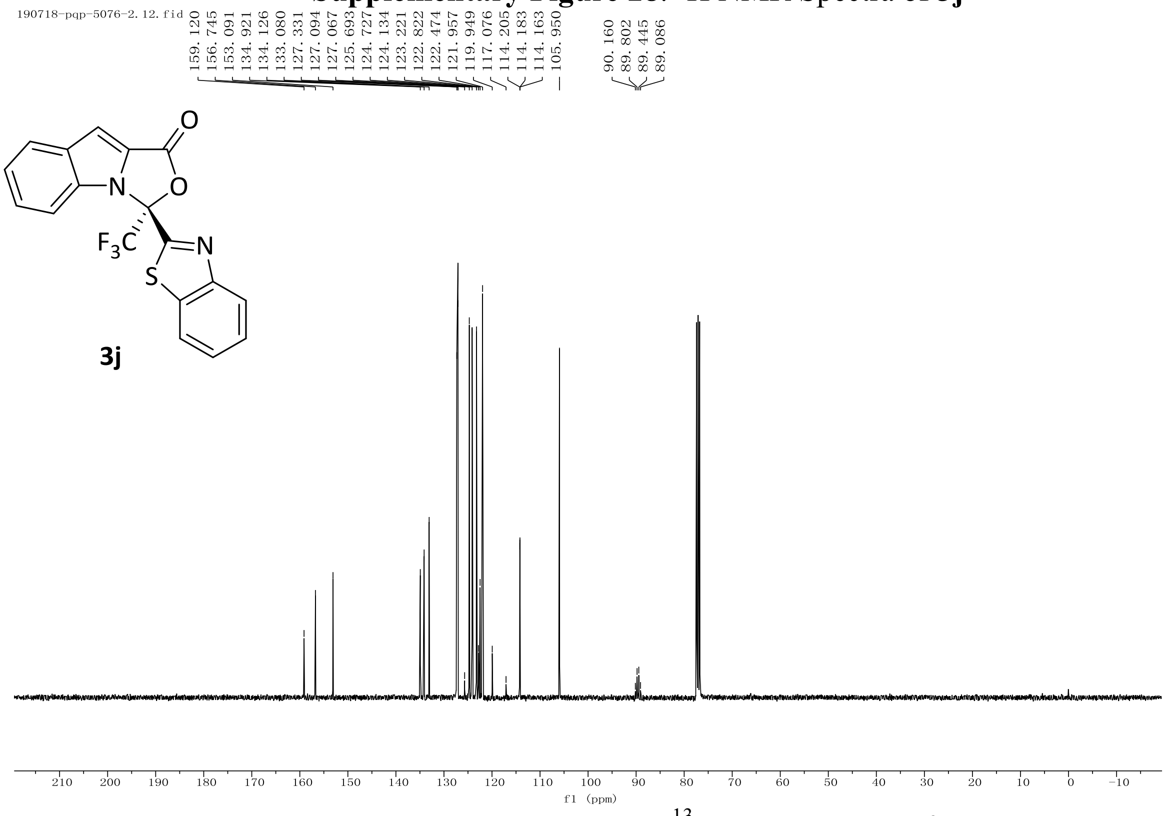

Supplementary Figure 29. ^{13}C NMR Spectra of **3j**

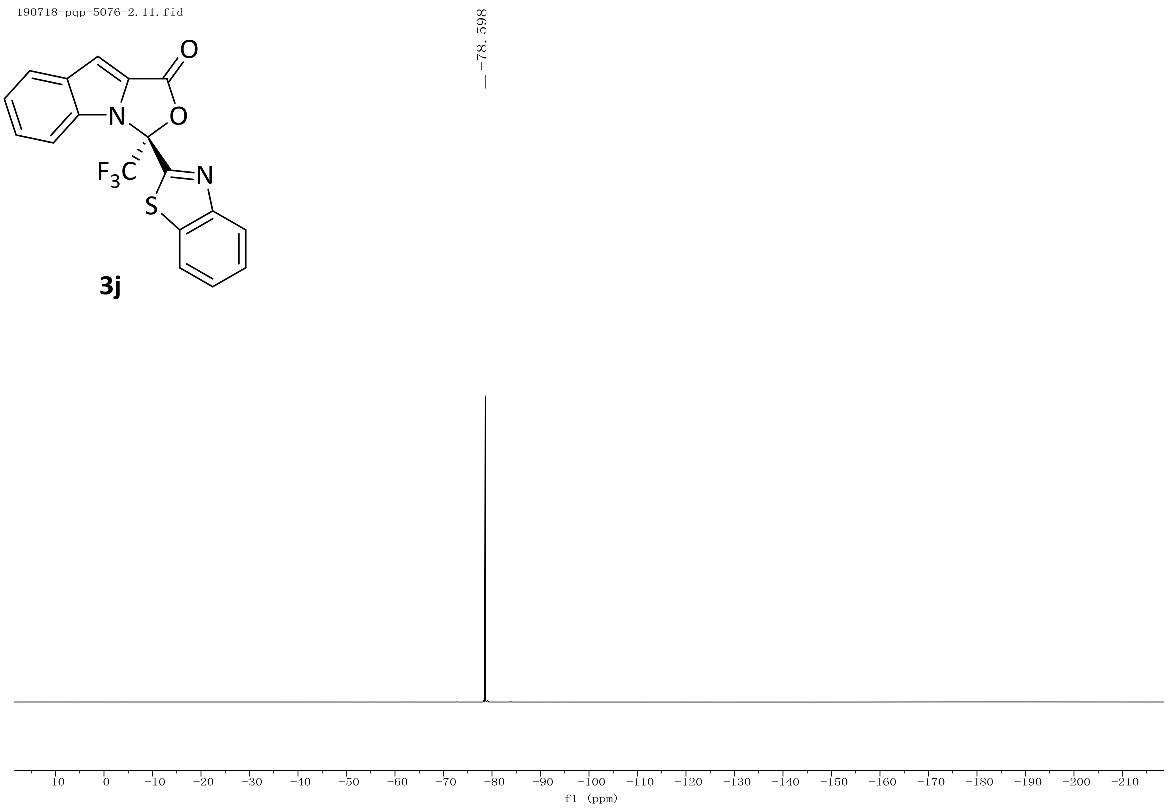

Supplementary Figure 30. ^{19}F NMR Spectra of **3j**

3k (*S*)-3-(thiophen-2-yl)-3-(trifluoromethyl)-1*H*,3*H*-oxazolo[3,4-a]indol-1-one

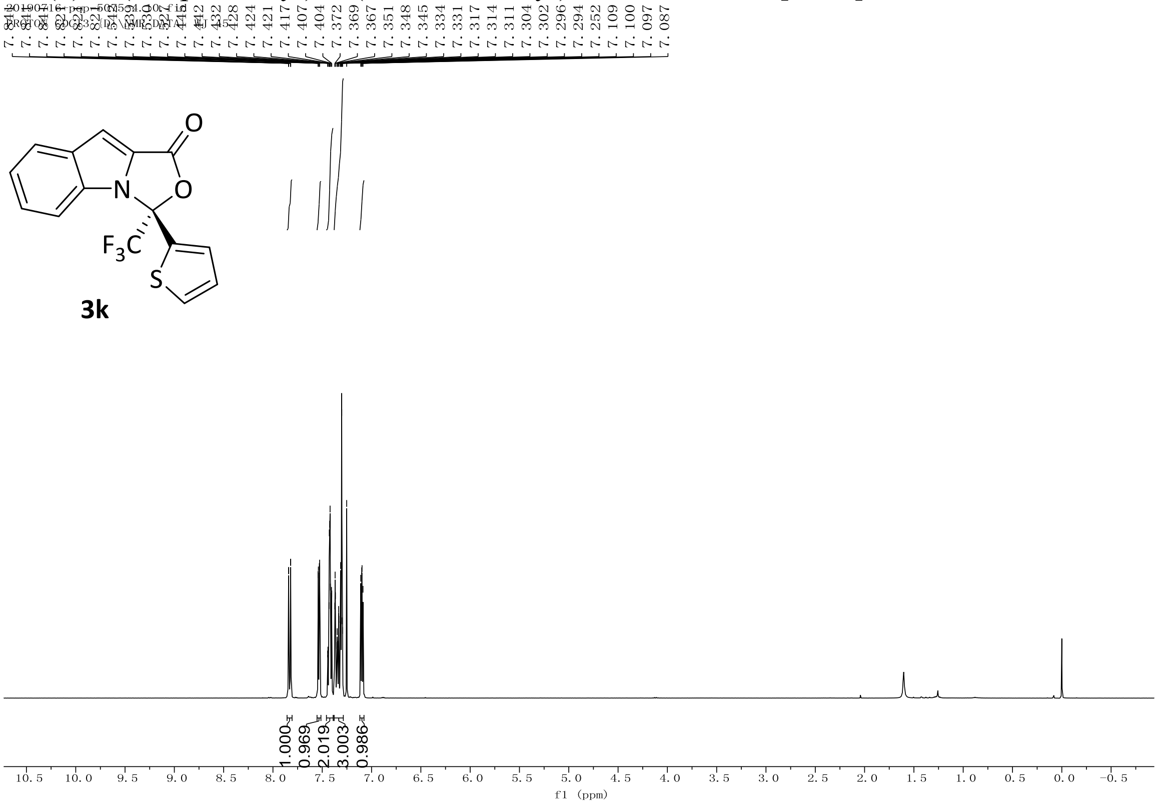

Supplementary Figure 31. ^{1}H NMR Spectra of **3k**

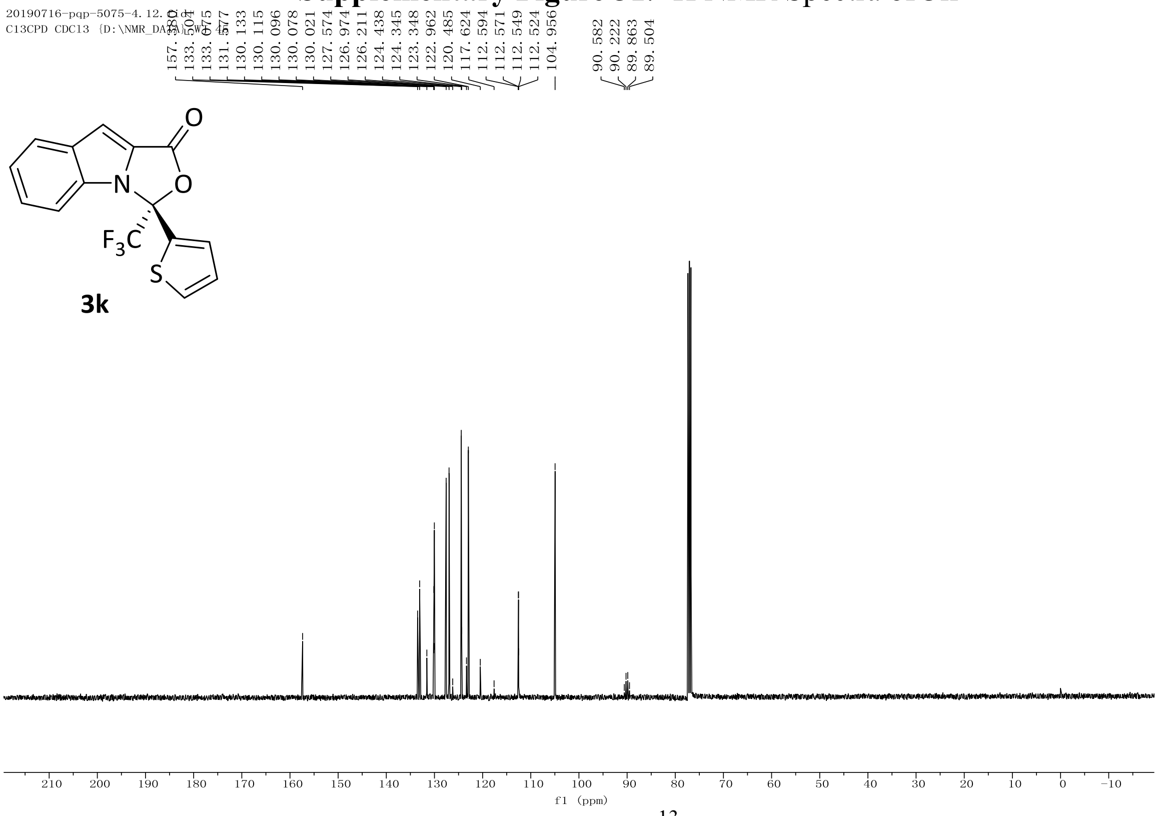

Supplementary Figure 32. ^{13}C NMR Spectra of **3k**

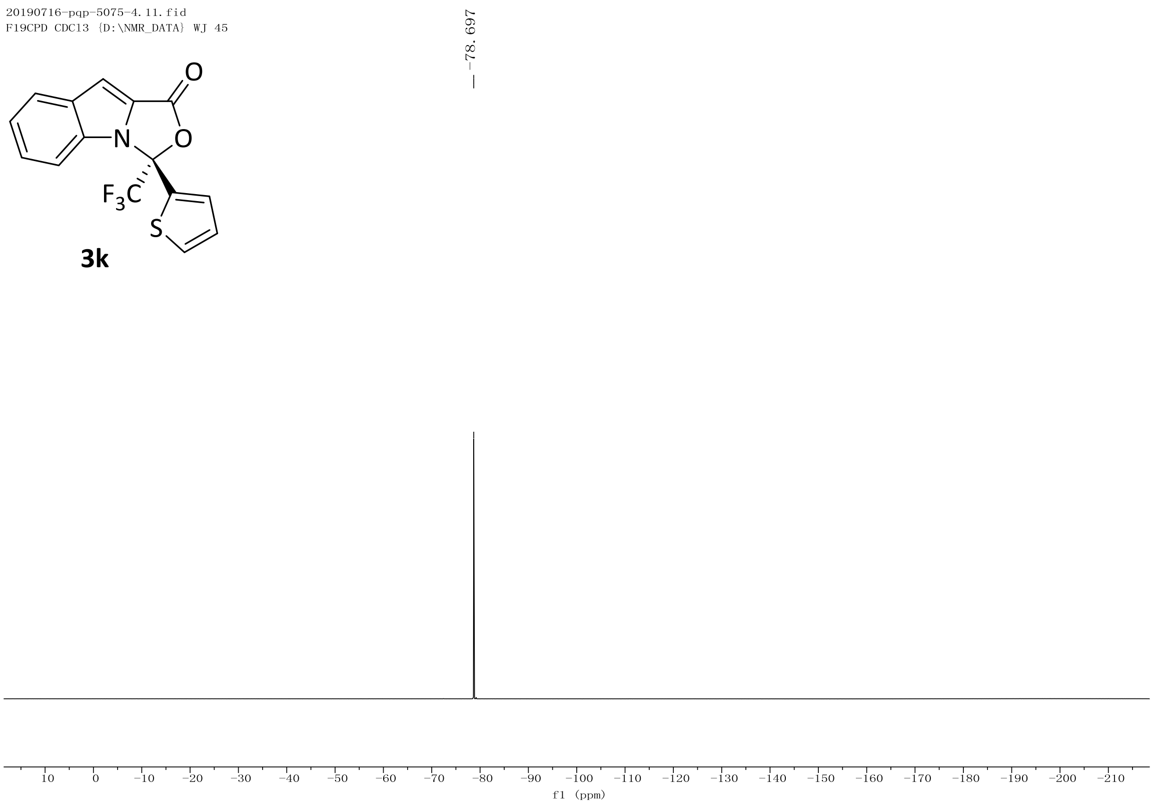

Supplementary Figure 33. ^{13}C NMR Spectra of **3k**

3l (*R*)-3-(pyridin-2-yl)-3-(trifluoromethyl)-1*H*,3*H*-oxazolo[3,4-a]indol-1-one

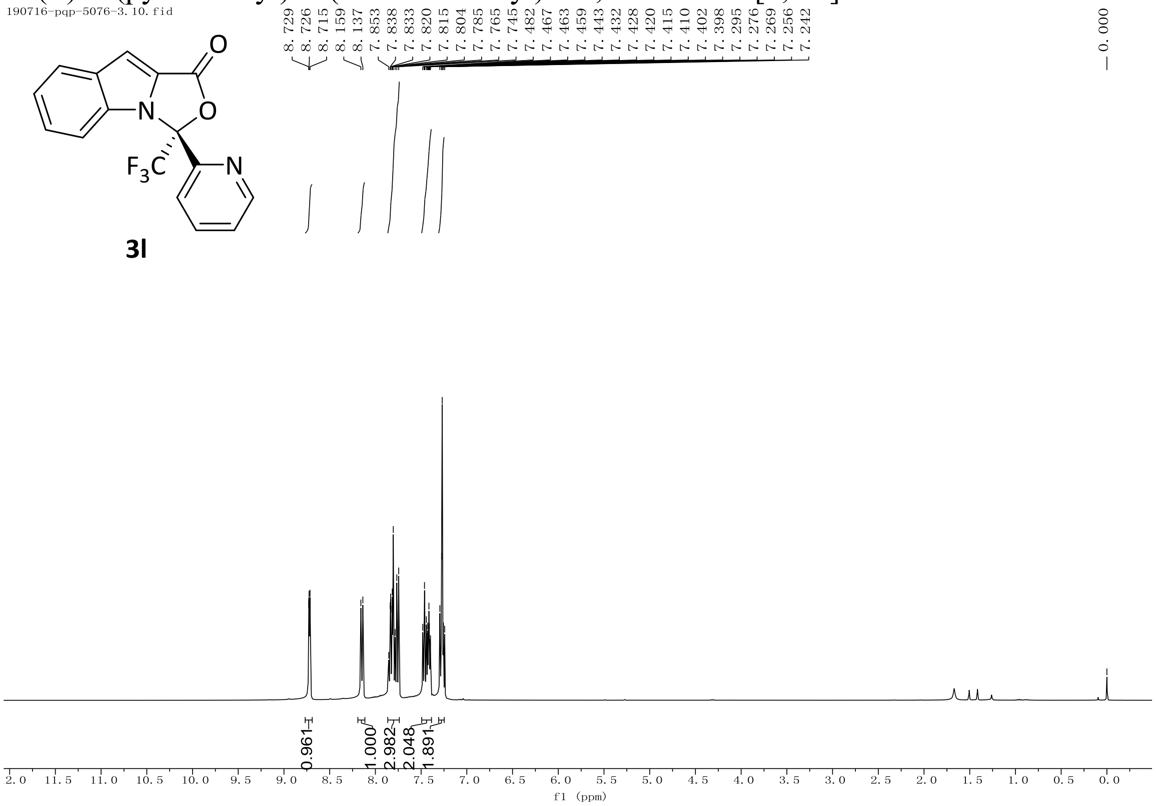

Supplementary Figure 34. ^{1}H NMR Spectra of **3l**

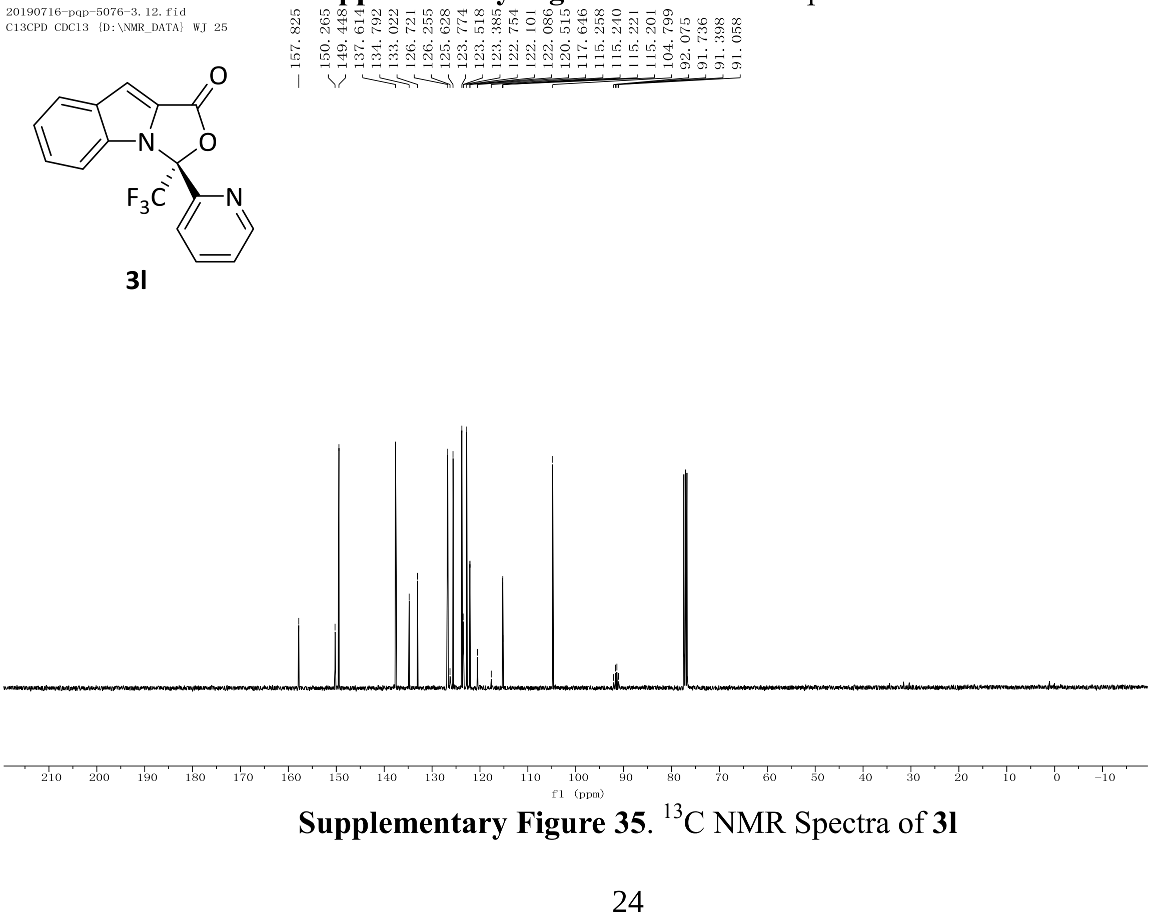

Supplementary Figure 35. ^{13}C NMR Spectra of **3l**

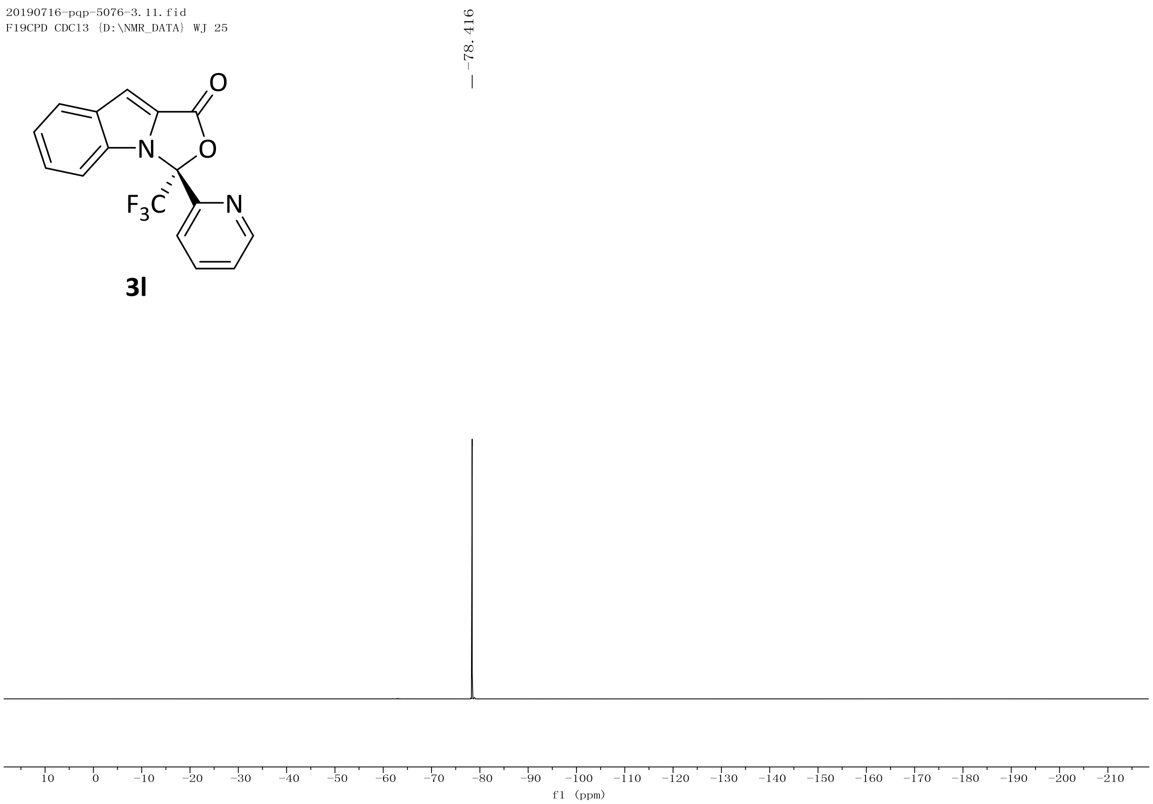

Supplementary Figure 36. ^{19}F NMR Spectra of **3l**

3m (*R*)-3-(quinolin-6-yl)-3-(trifluoromethyl)-1*H*,3*H*-oxazolo[3,4-a]indol-1-one

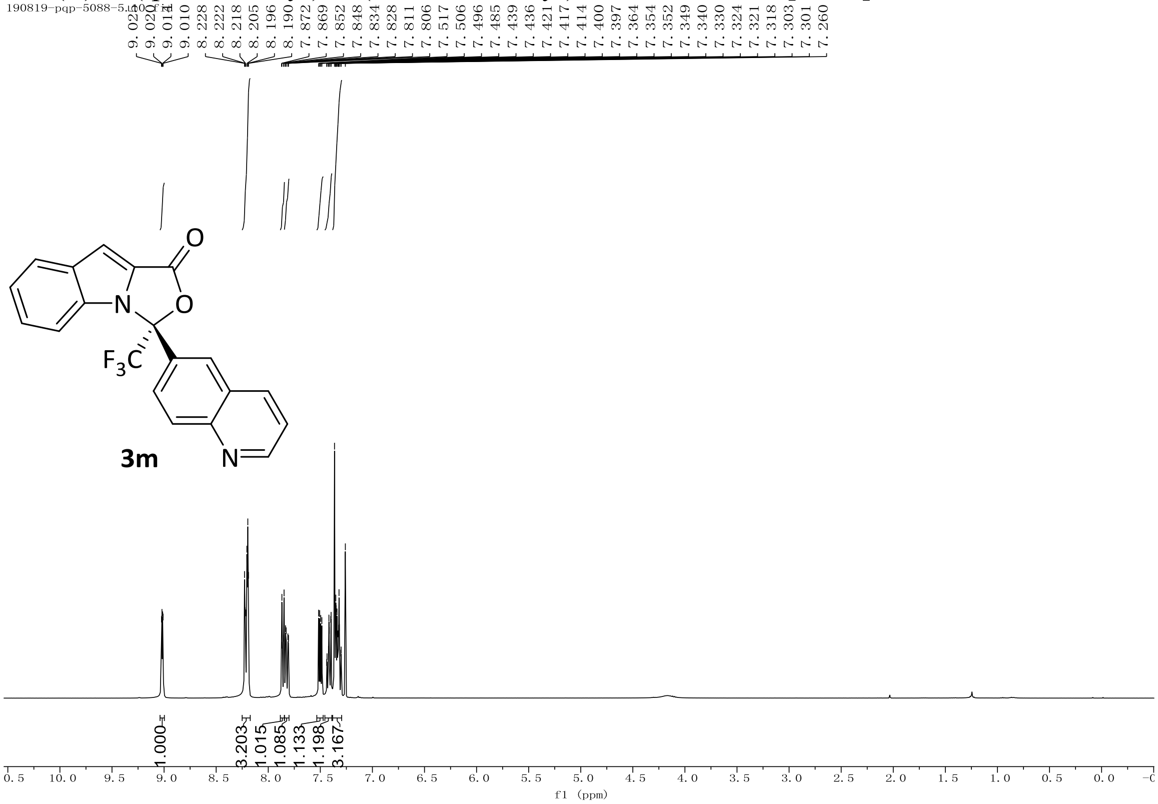

Supplementary Figure 37. ^{1}H NMR Spectra of **3m**

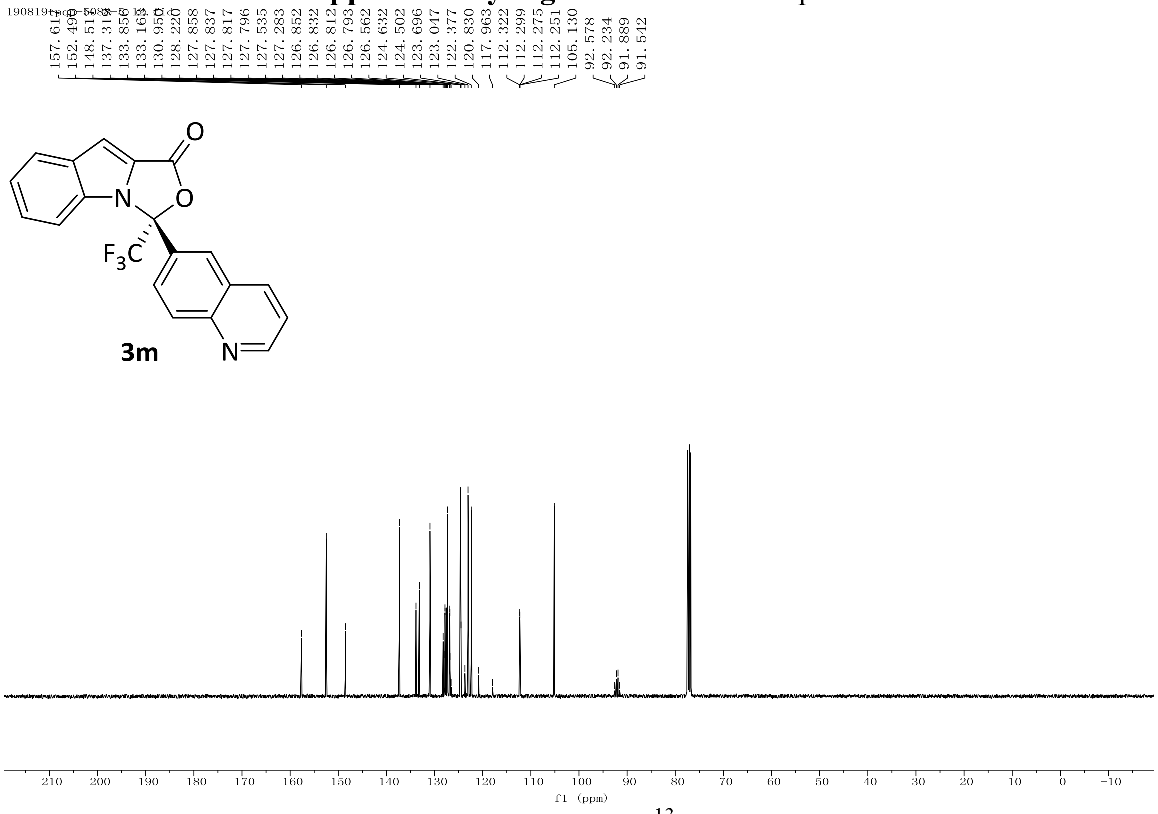

Supplementary Figure 38. ^{13}C NMR Spectra of **3m**

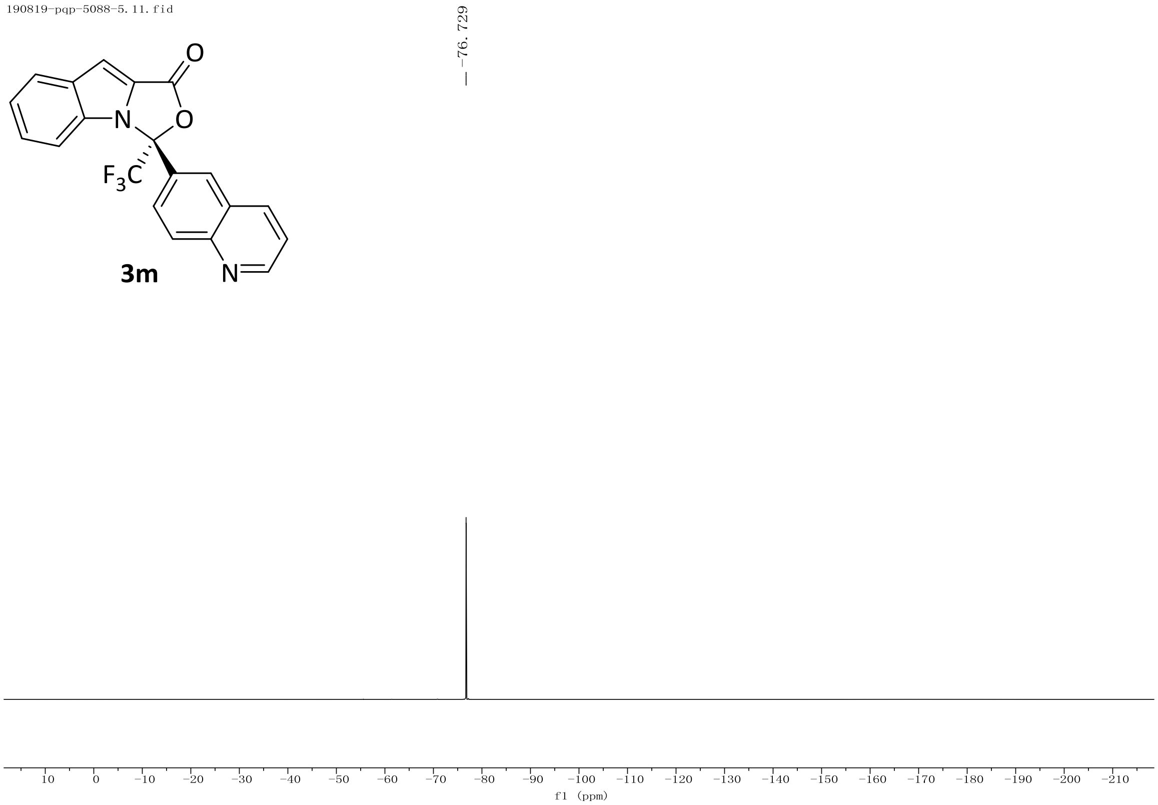

Supplementary Figure 39. ^{19}F NMR Spectra of **3m**

3n (*R*)-3-(quinolin-3-yl)-3-(trifluoromethyl)-1*H*,3*H*-oxazolo[3,4-a]indol-1-one

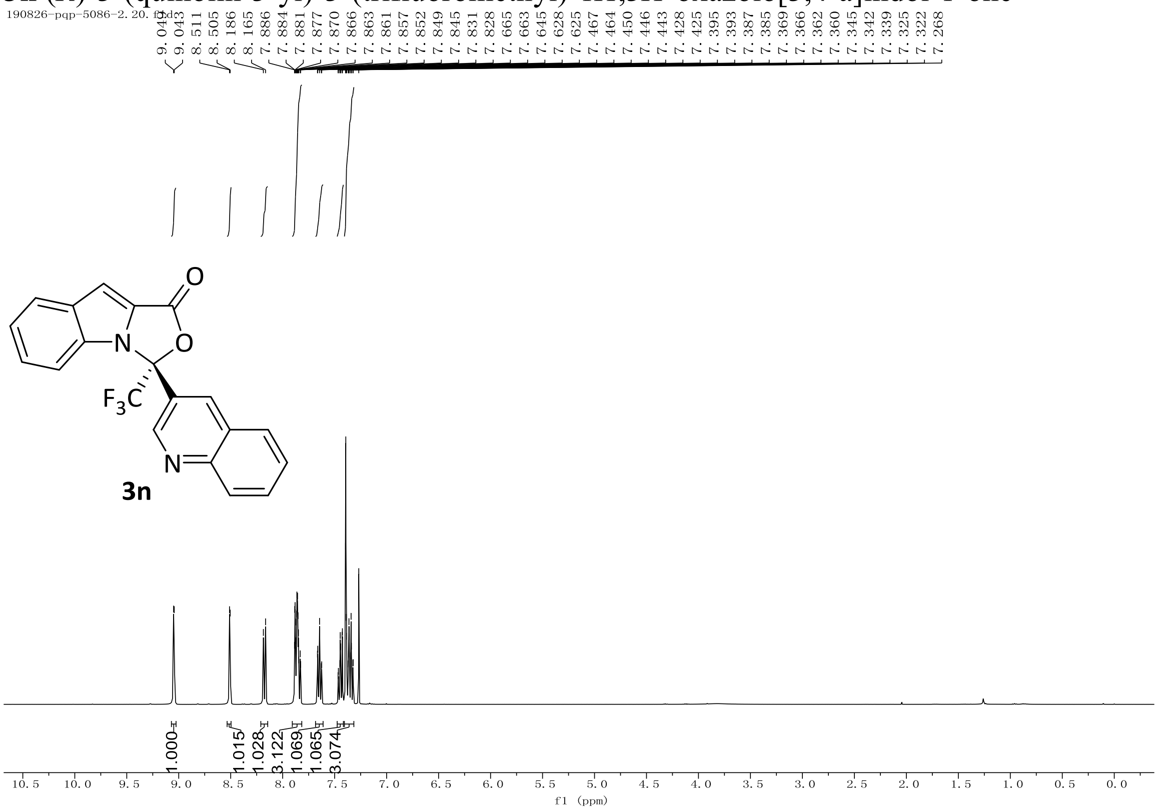

Supplementary Figure 40. ^{1}H NMR Spectra of **3n**

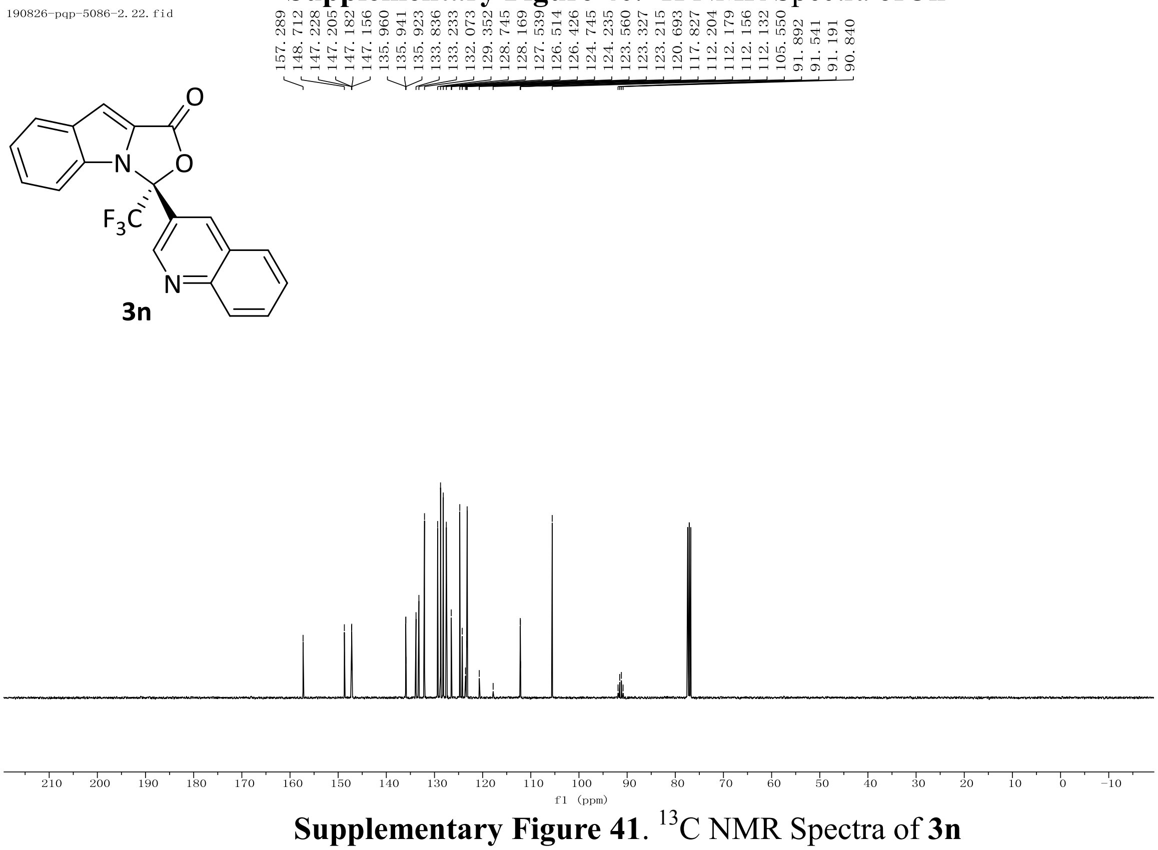

Supplementary Figure 41. ^{13}C NMR Spectra of **3n**

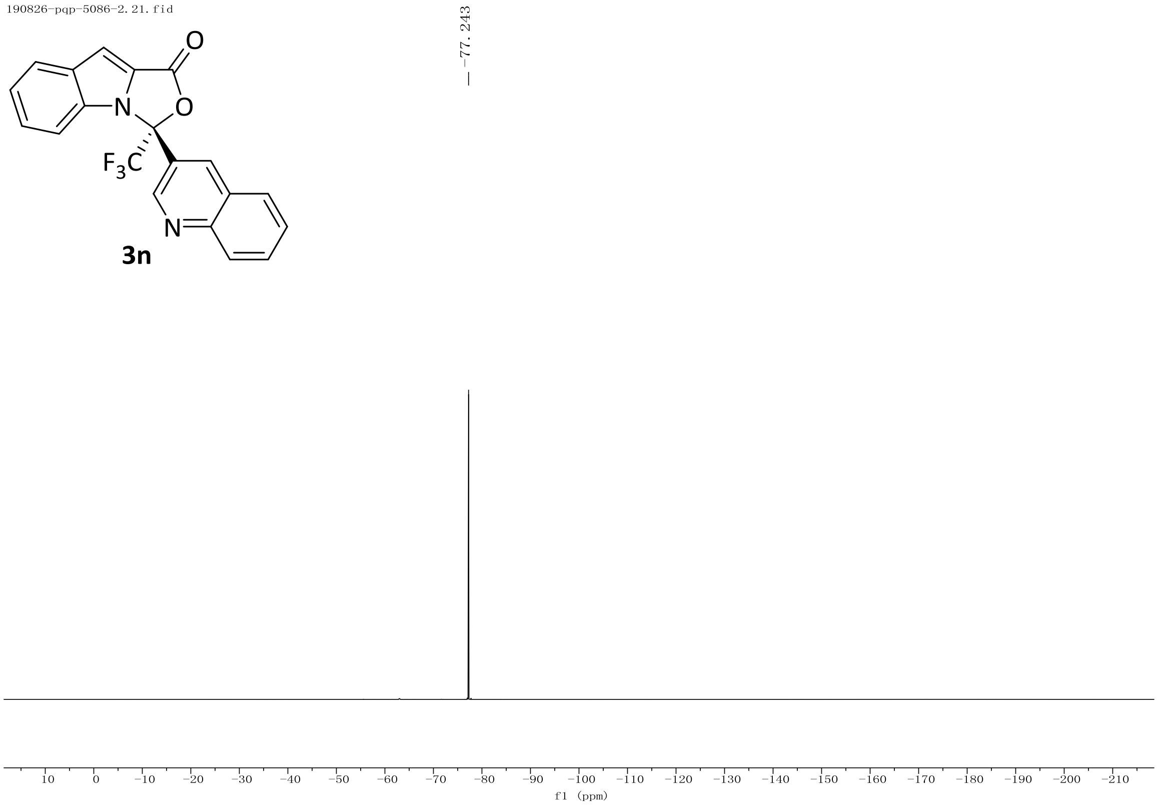

Supplementary Figure 42. ^{19}F NMR Spectra of **3n**

3o (*R*)-3-ethyl-3-(trifluoromethyl)-1*H*,3*H*-oxazolo[3,4-a]indol-1-one

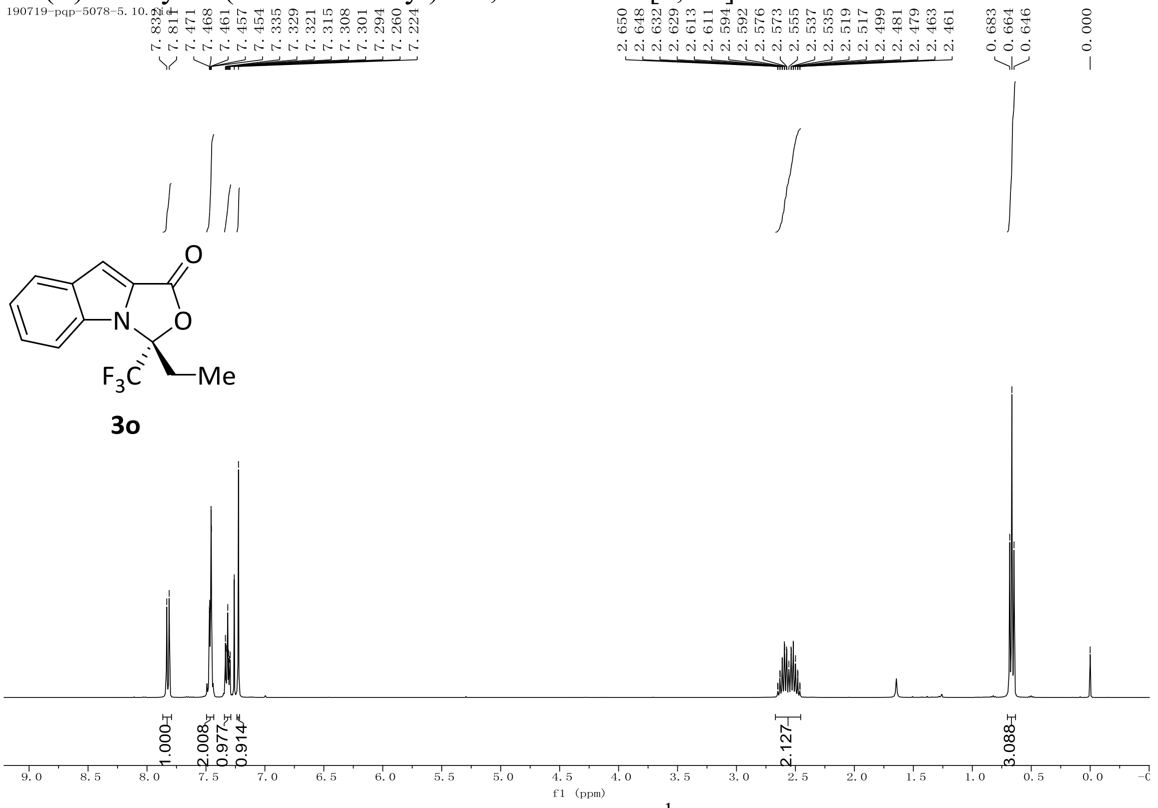

Supplementary Figure 43. ^{1}H NMR Spectra of **3o**

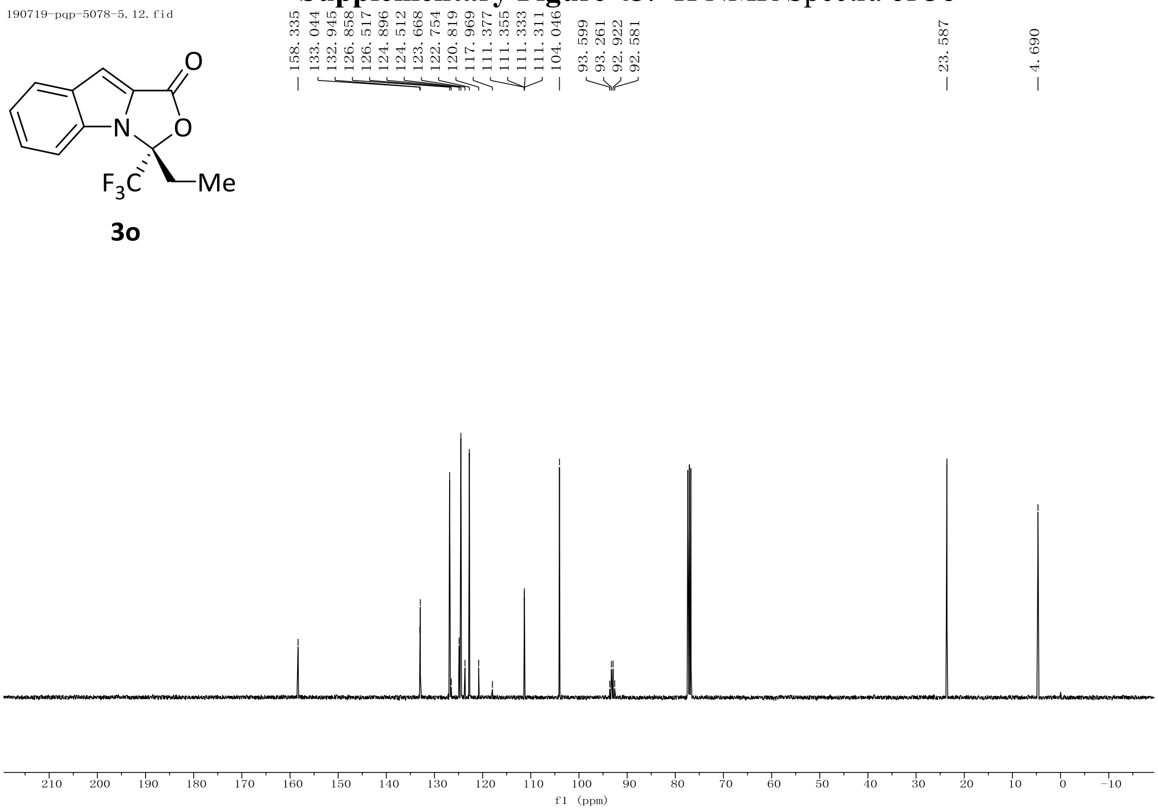

Supplementary Figure 44. ^{13}C NMR Spectra of **3o**

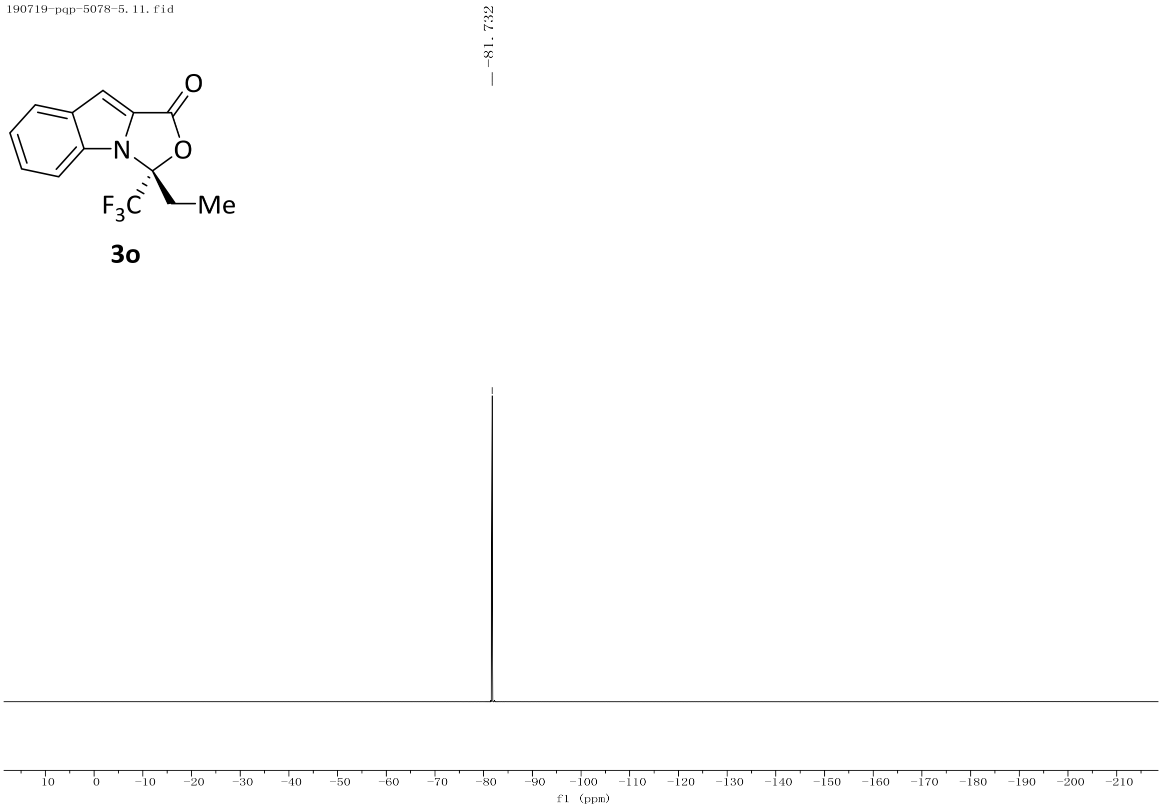

Supplementary Figure 45. ^{19}F NMR Spectra of **3o**

3p (*R*)-3-benzyl-3-(trifluoromethyl)-1*H*,3*H*-oxazolo[3,4-a]indol-1-one

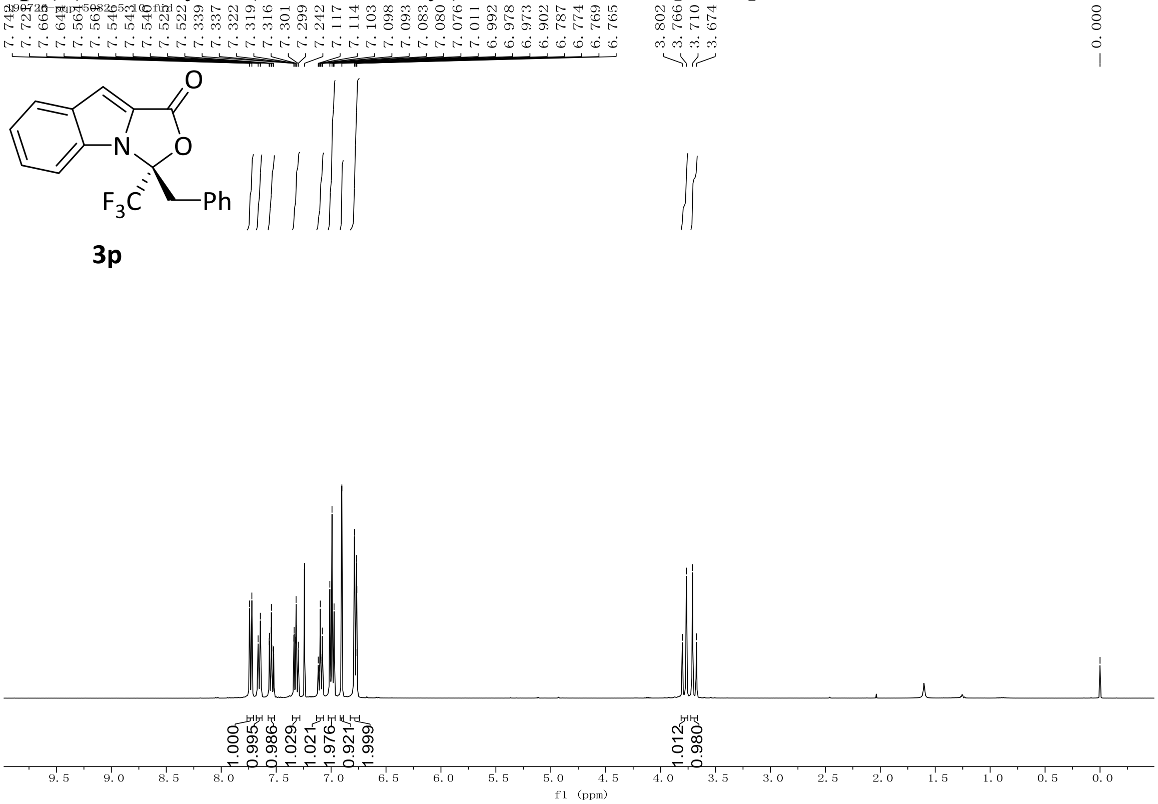

Supplementary Figure 46. ^{1}H NMR Spectra of **3p**

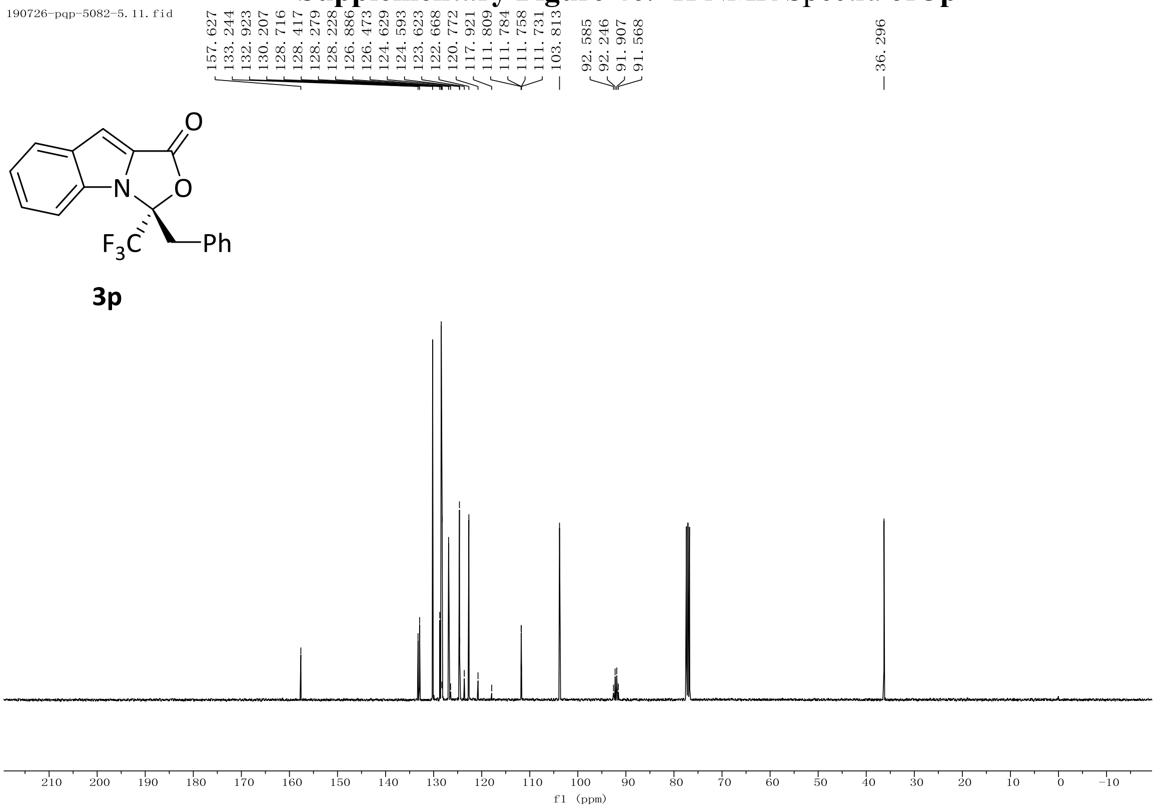

Supplementary Figure 47. ^{13}C NMR Spectra of **3p**

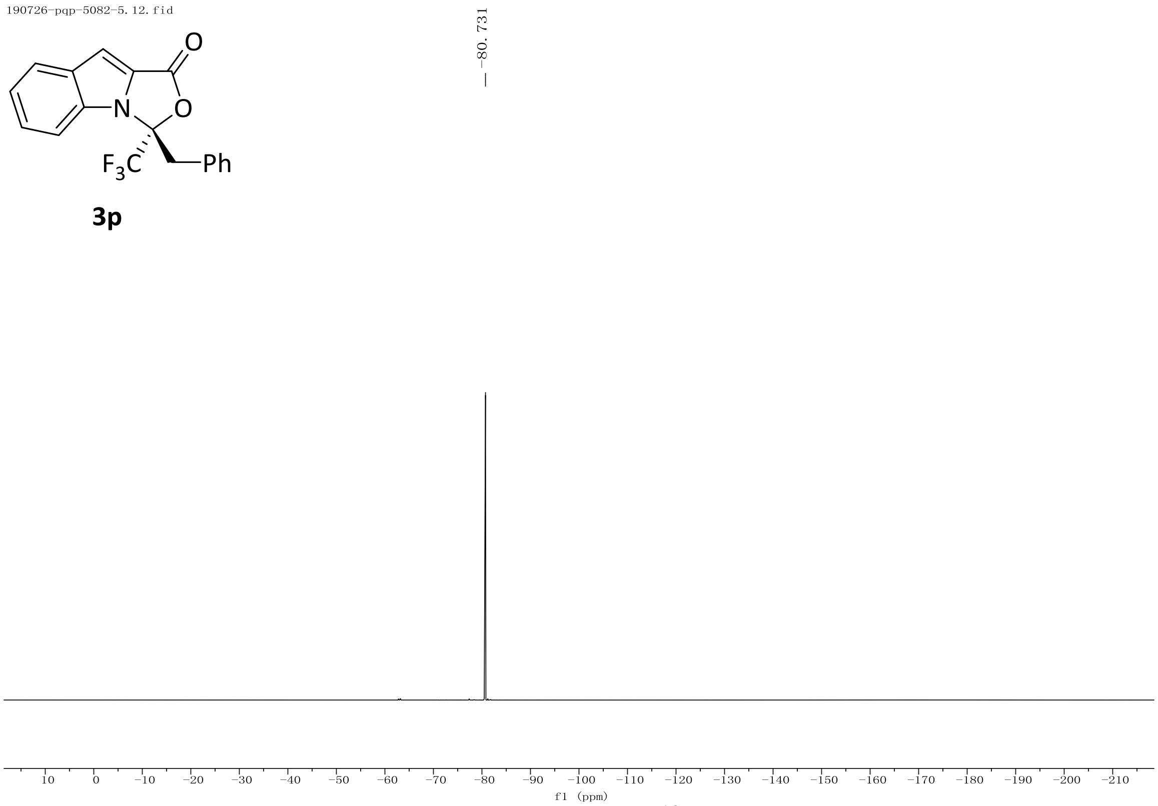

Supplementary Figure 48. ^{19}F NMR Spectra of **3p**

3q (*R,E*)-3-styryl-3-(trifluoromethyl)-1*H*,3*H*-oxazolo[3,4-a]indol-1-one

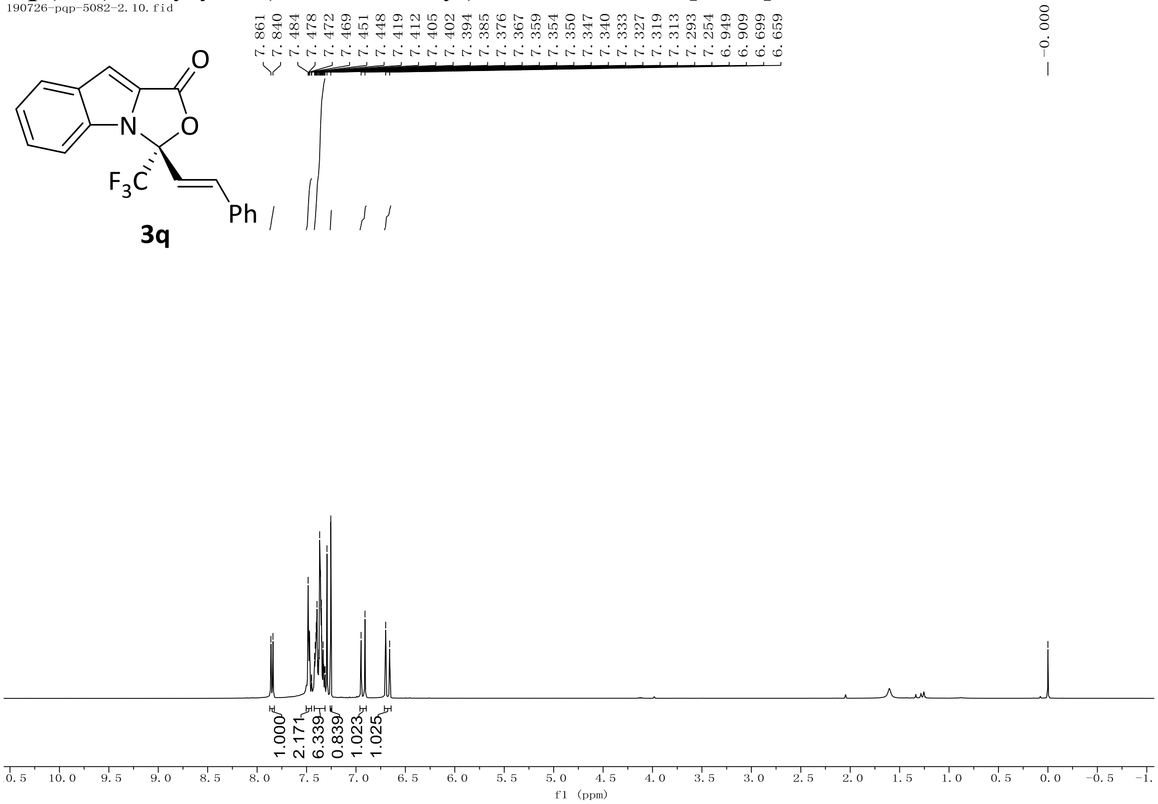

Supplementary Figure 49. ^{1}H NMR Spectra of **3q**

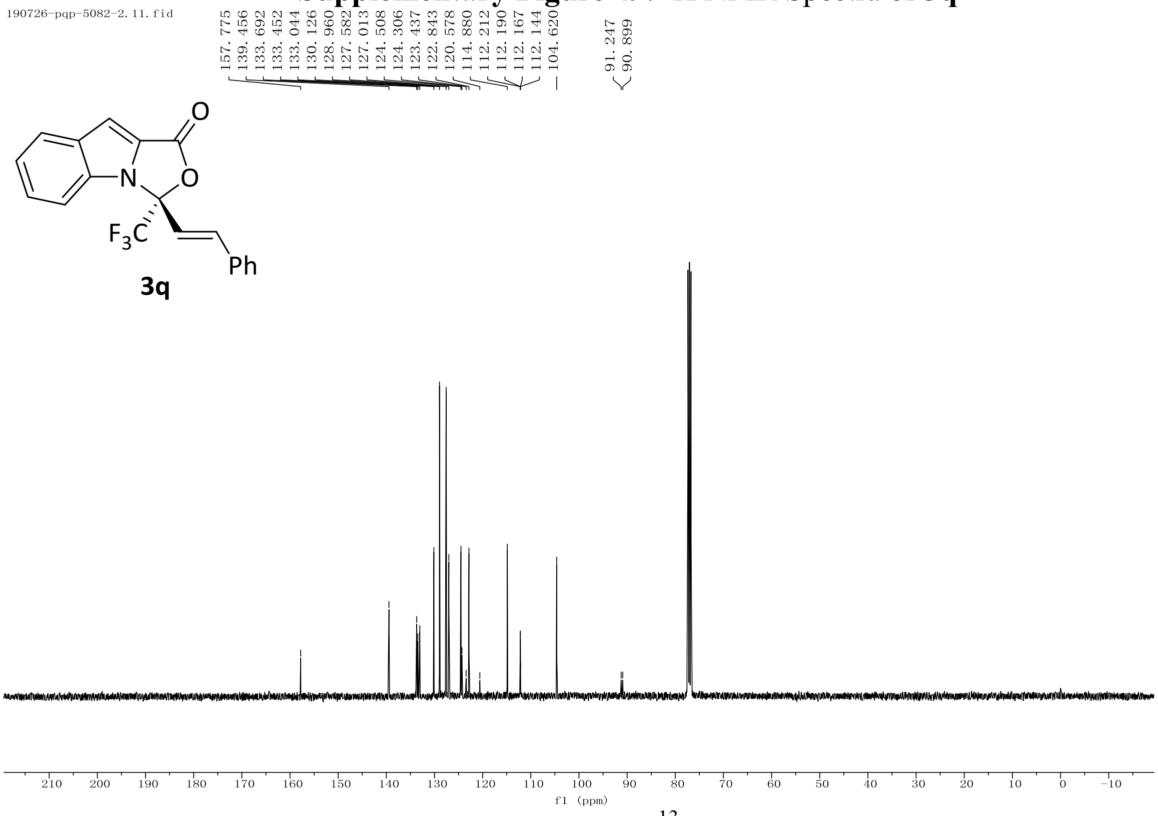

Supplementary Figure 50. ^{13}C NMR Spectra of **3q**

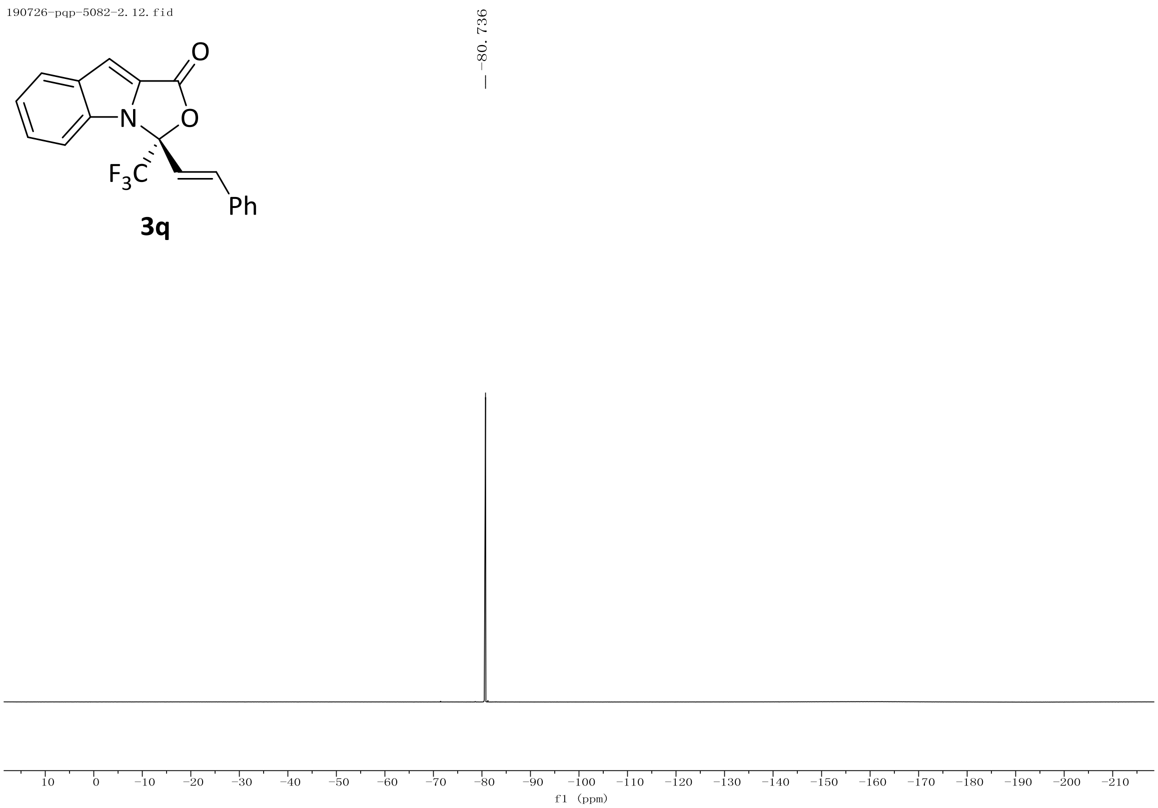

Supplementary Figure 51. ^{19}F NMR Spectra of **3q**

3r (*R*)-3-(difluoromethyl)-3-phenyl-1*H*,3*H*-oxazolo[3,4-a]indol-1-one

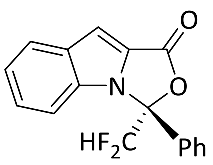

3r

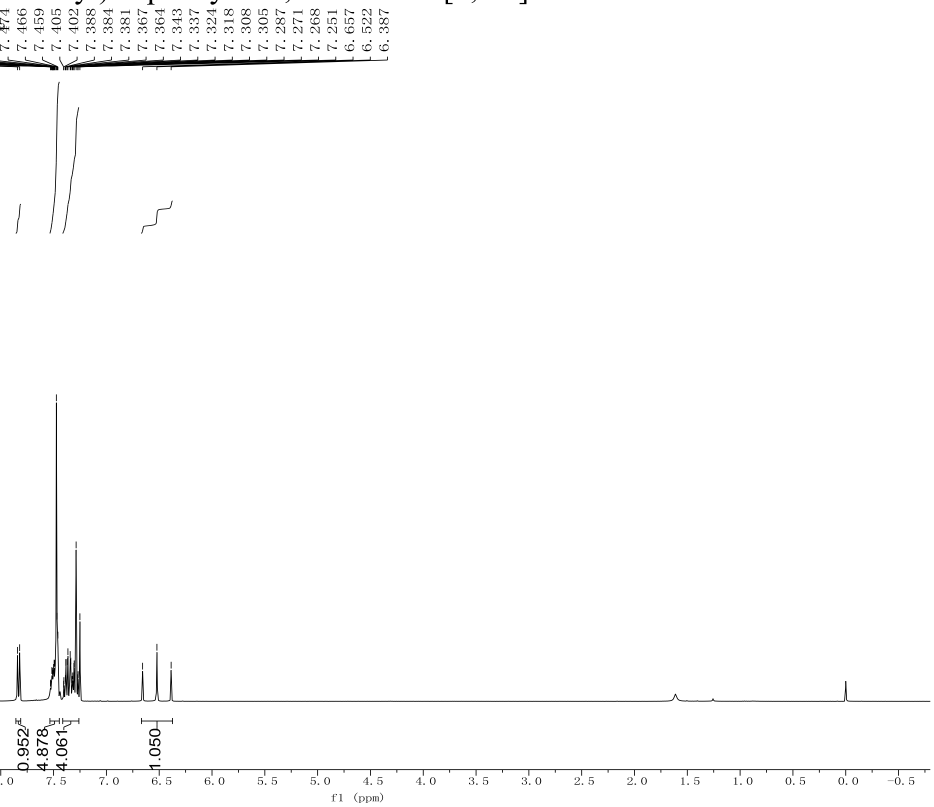

Supplementary Figure 52. ^{1}H NMR Spectra of **3r**

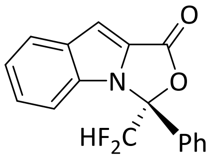

3r

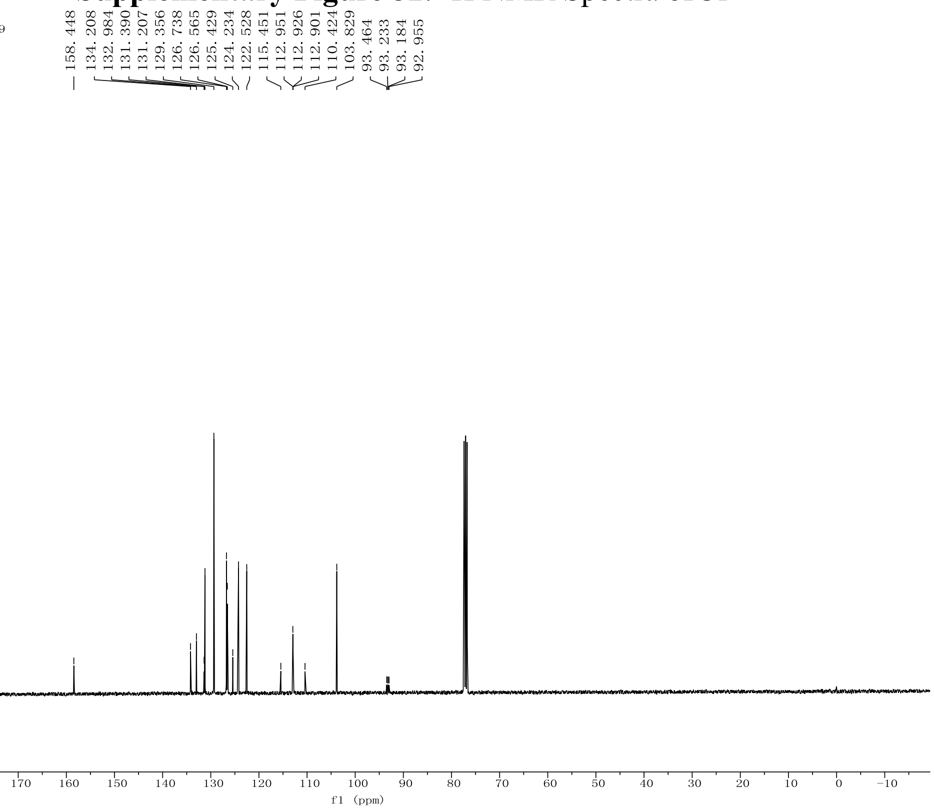

Supplementary Figure 53. ^{13}C NMR Spectra of **3r**

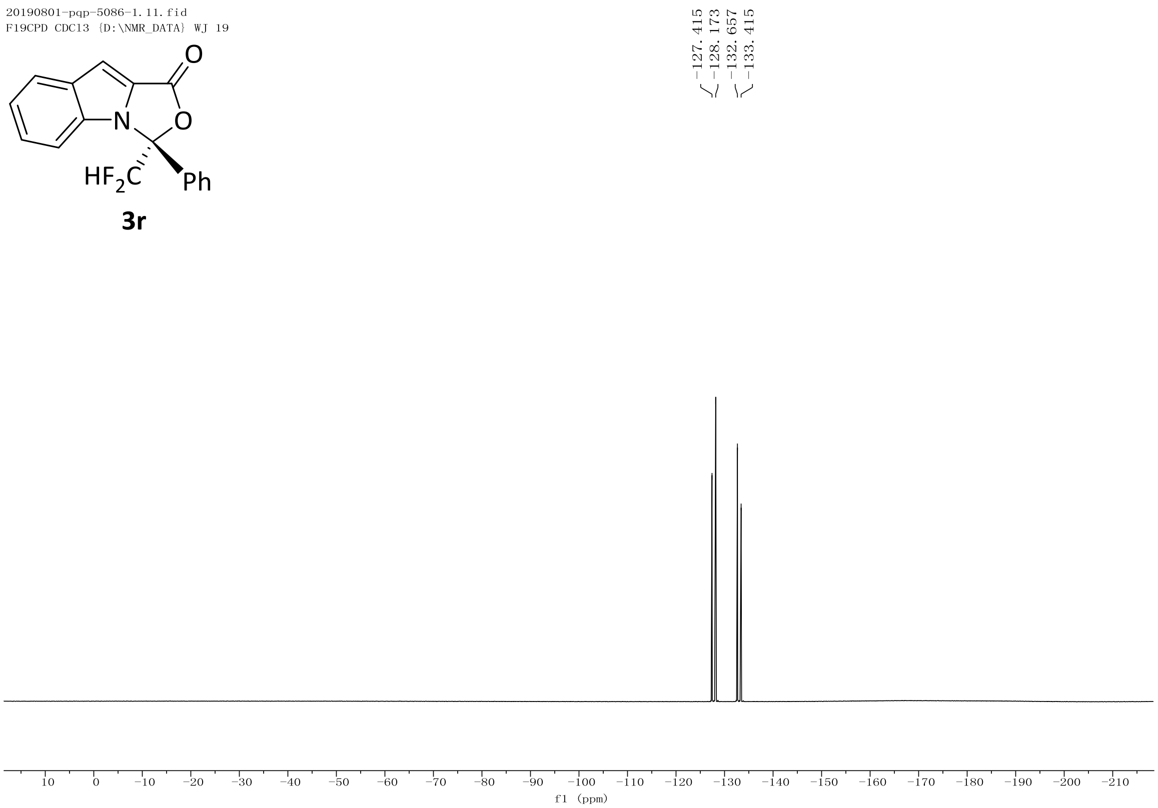

Supplementary Figure 54. ^{19}C NMR Spectra of **3r**

3s (*R*)-3-(chlorodifluoromethyl)-3-phenyl-1*H*,3*H*-oxazolo[3,4-a]indol-1-one

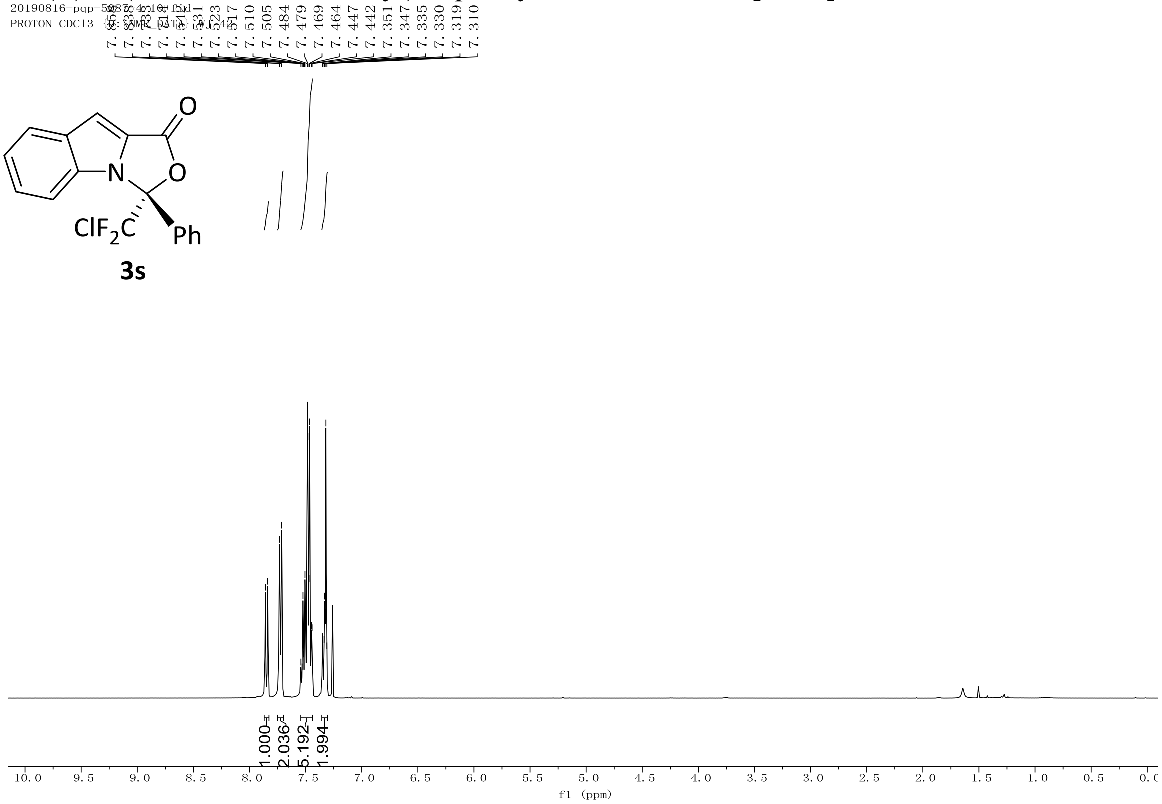

Supplementary Figure 55. ^{1}H NMR Spectra of **3s**

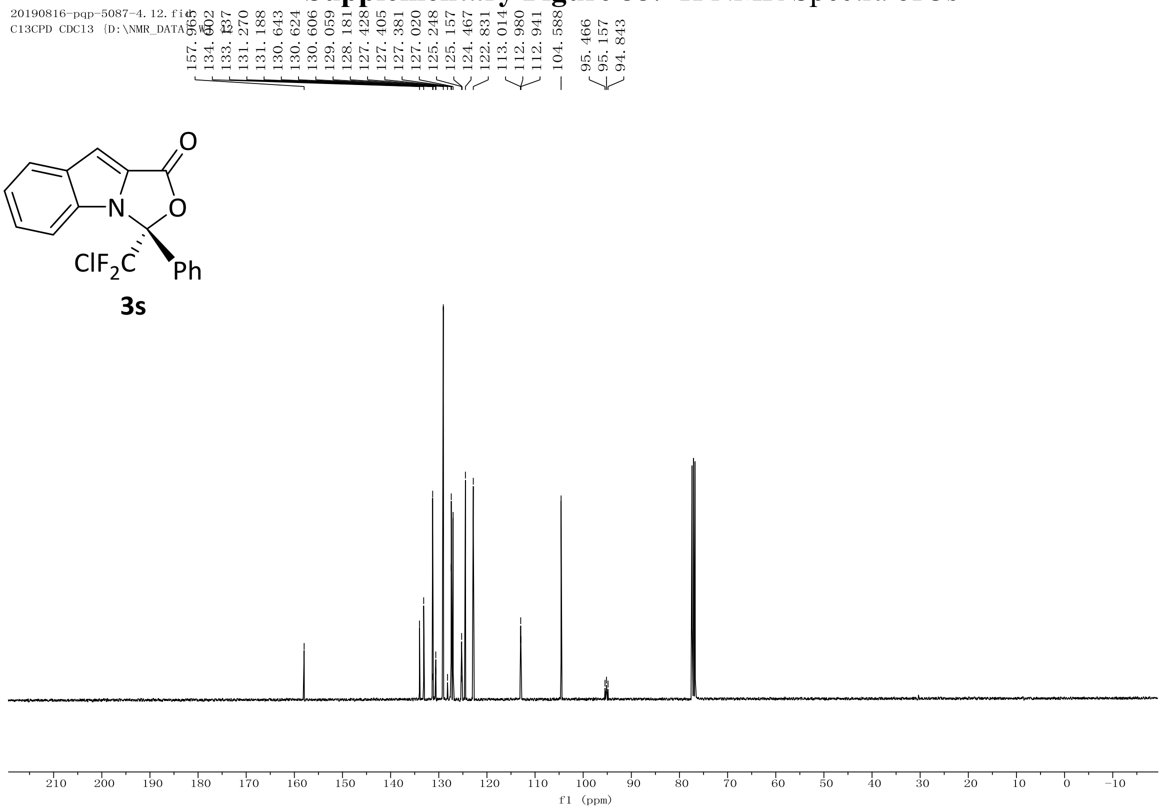

Supplementary Figure 56. ^{13}C NMR Spectra of **3s**

20190816-pqp-5087-4.11.fid
F19CPD CDCl3 {D:\NMR_DATA} WJ 42

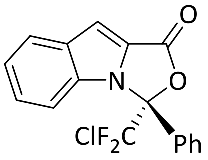

3s

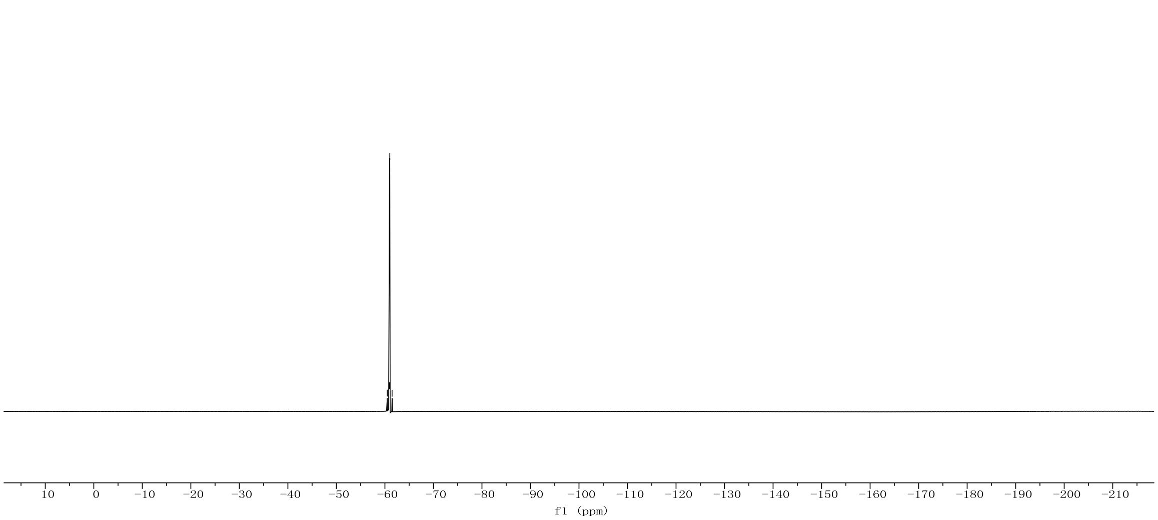

Supplementary Figure 57. ^{13}C NMR Spectra of **3s**

3t (*R*)-3-(perfluoroethyl)-3-phenyl-1*H*,3*H*-oxazolo[3,4-a]indol-1-one

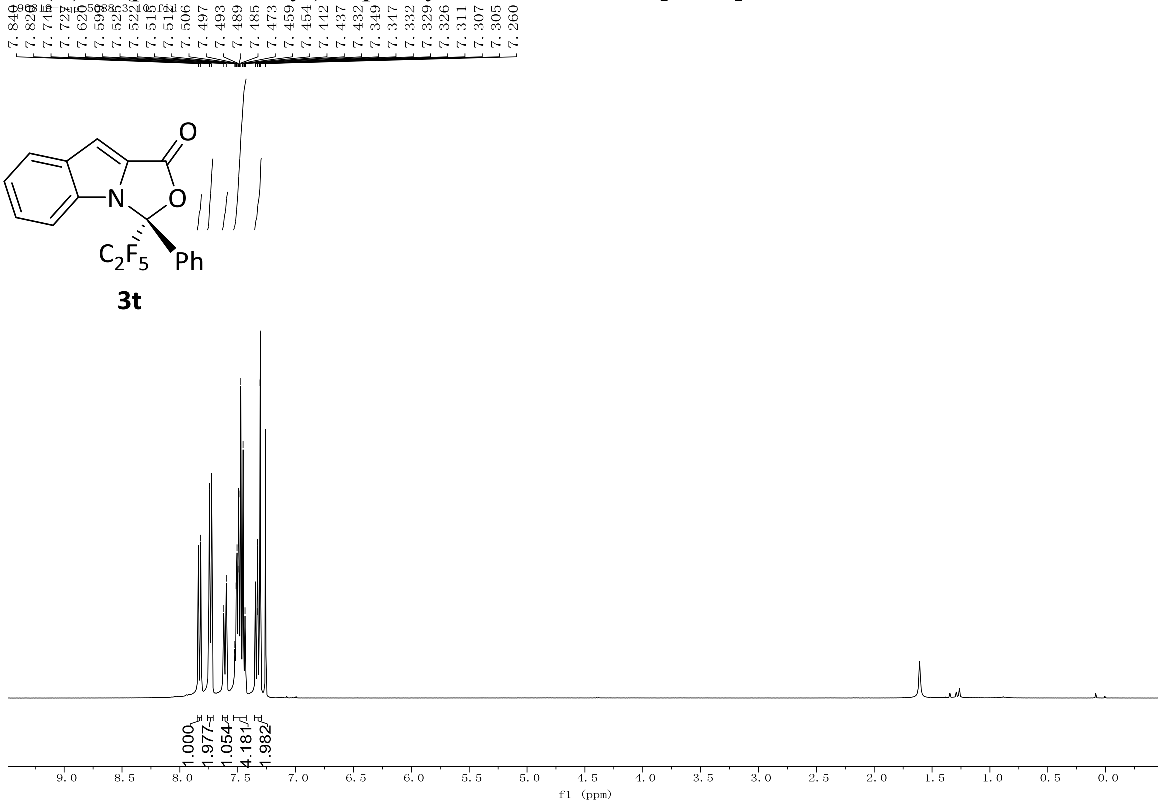

Supplementary Figure 58. ^{1}H NMR Spectra of **3t**

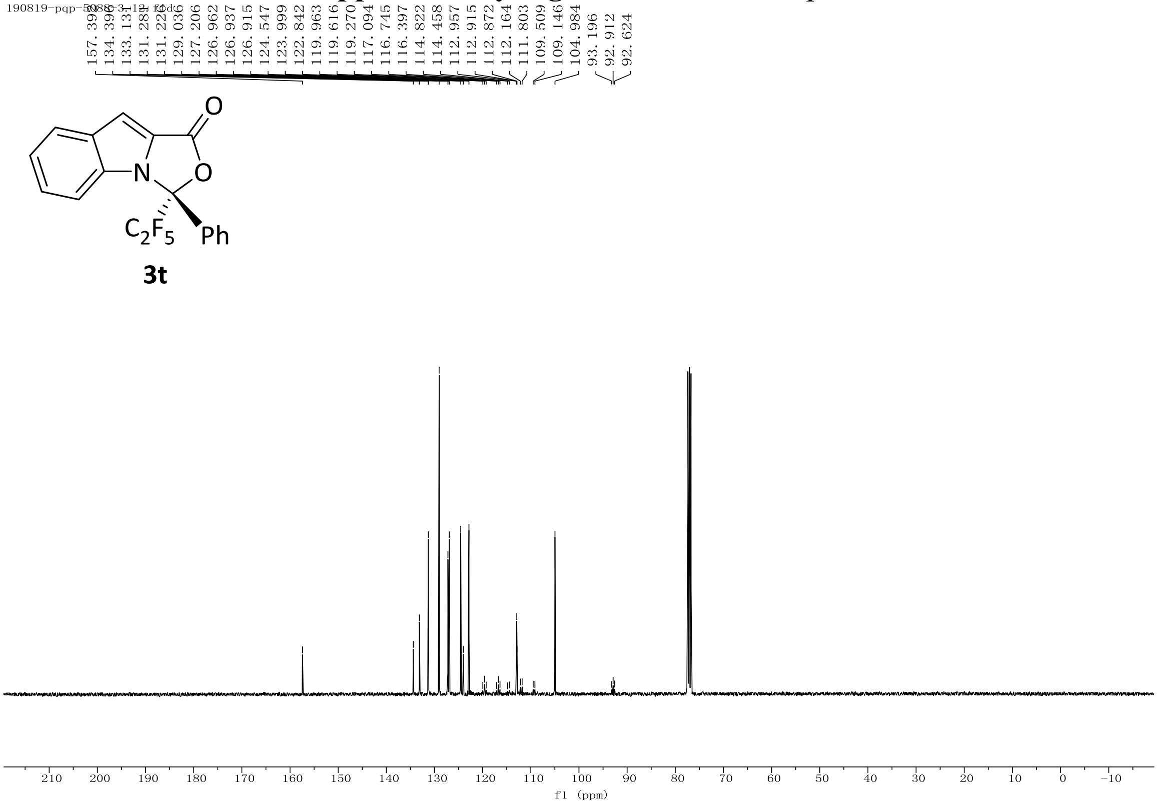

Supplementary Figure 59. ^{13}C NMR Spectra of **3t**

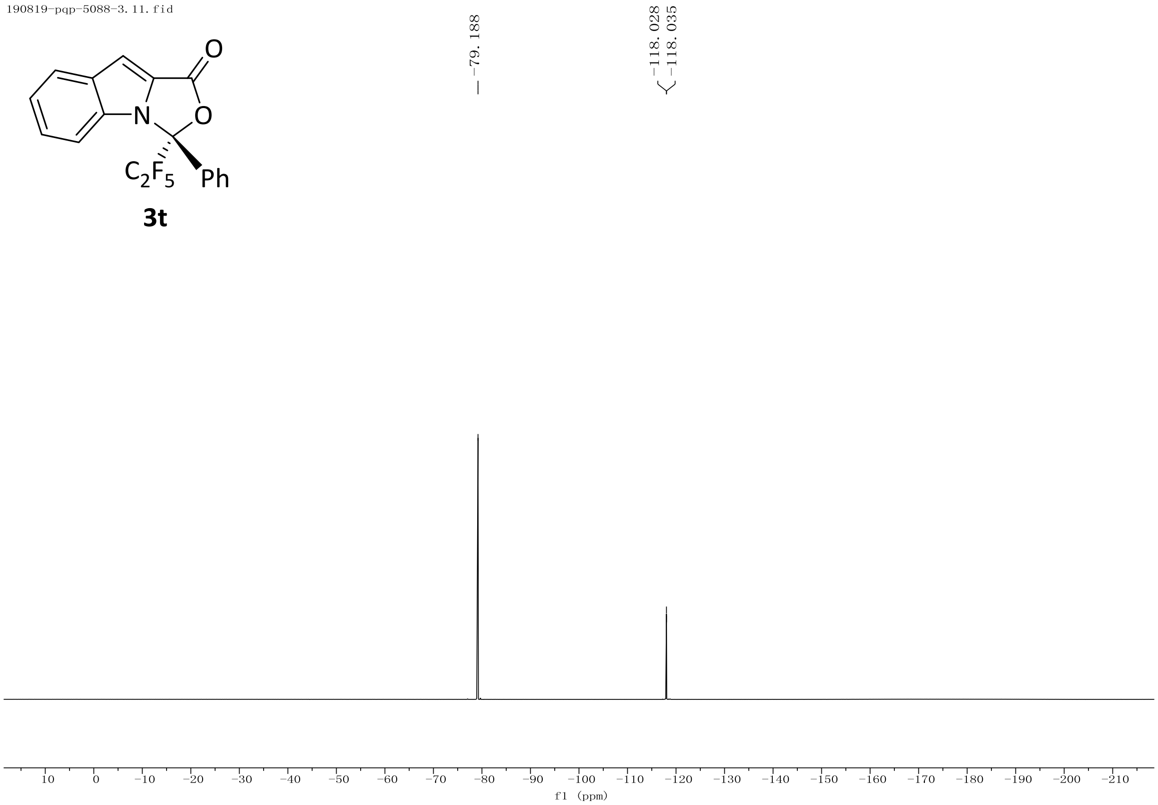

Supplementary Figure 60. ^{19}F NMR Spectra of **3t**

4a (*R*)-7-fluoro-3-phenyl-3-(trifluoromethyl)-1*H*,3*H*-oxazolo[3,4-a]indol-1-one

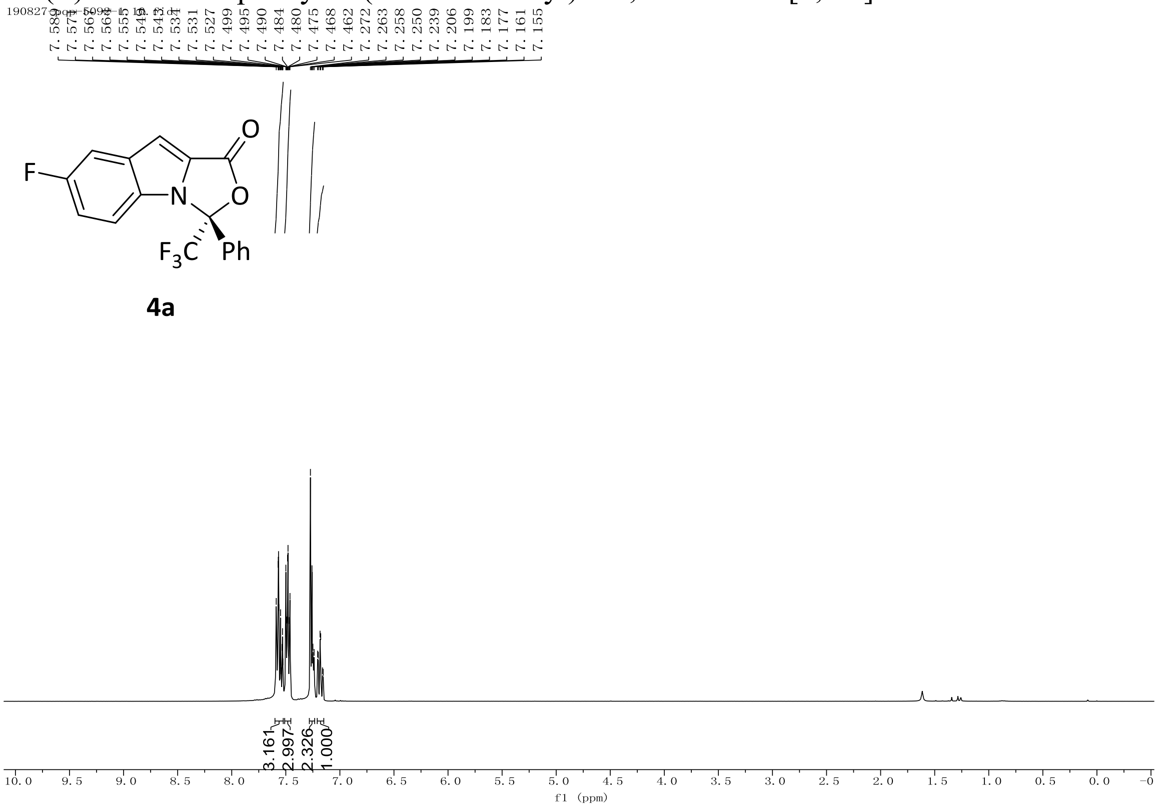

Supplementary Figure 61. ^{1}H NMR Spectra of **4a**

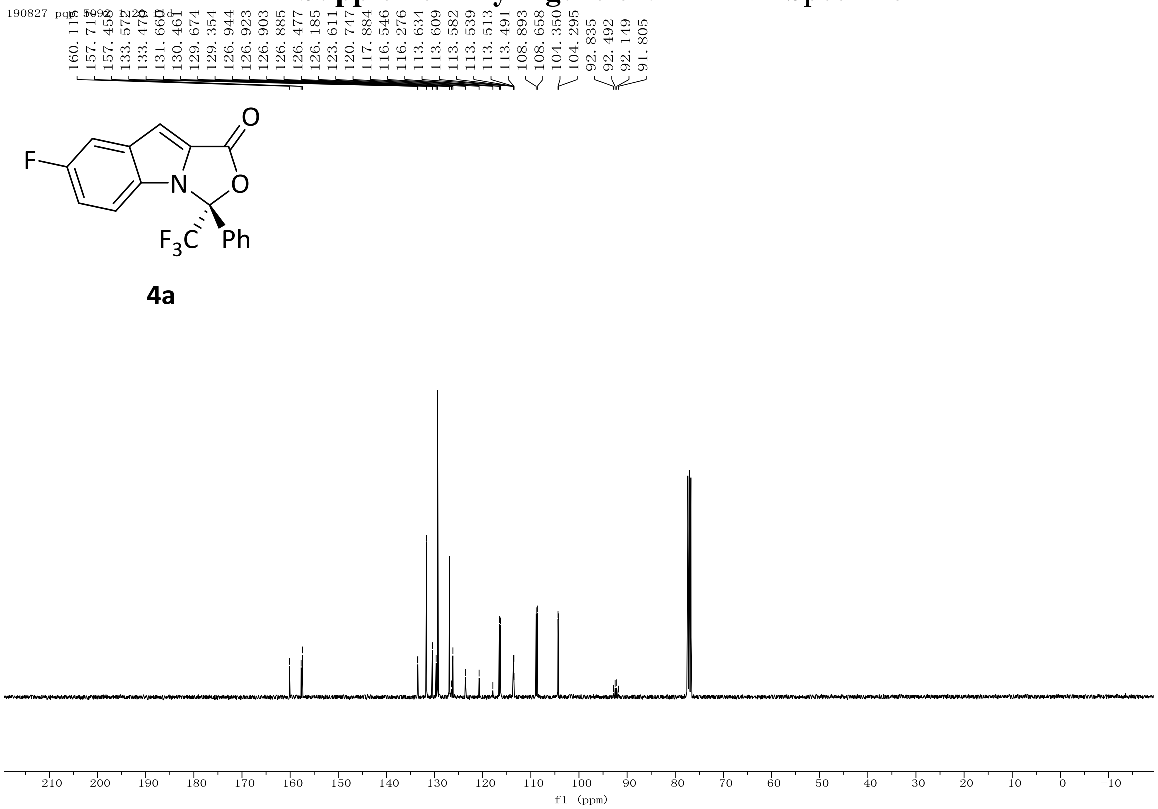

Supplementary Figure 62. ^{13}C NMR Spectra of **4a**

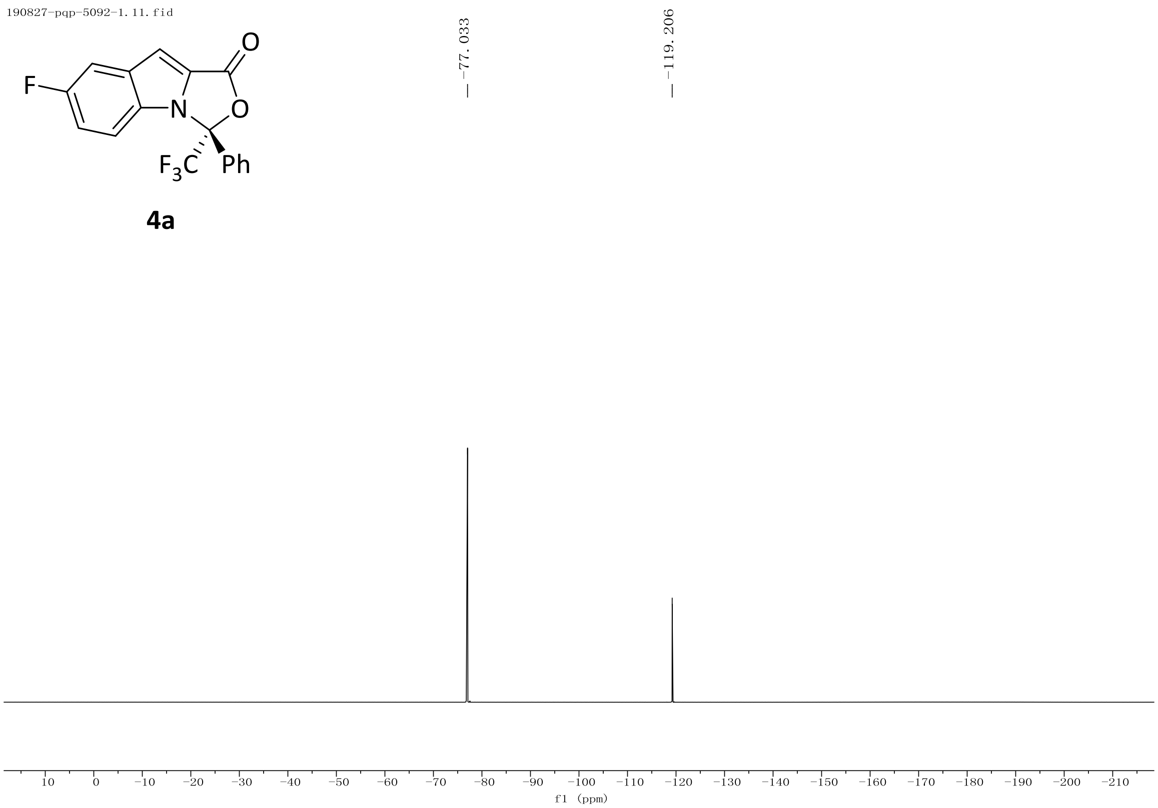

Supplementary Figure 63. ^{19}F NMR Spectra of **4a**

4b (*R*)-7-chloro-3-phenyl-3-(trifluoromethyl)-1*H*,3*H*-oxazolo[3,4-a]indol-1-one

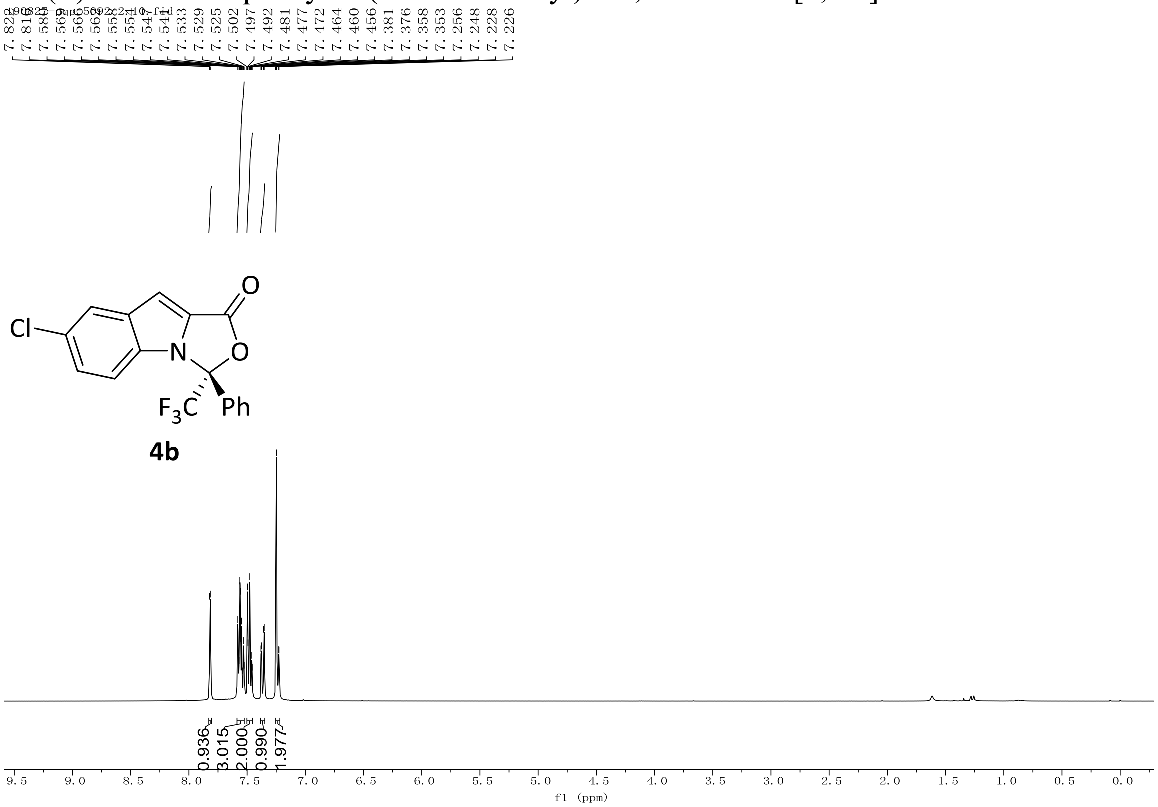

Supplementary Figure 64. ^{1}H NMR Spectra of **4b**

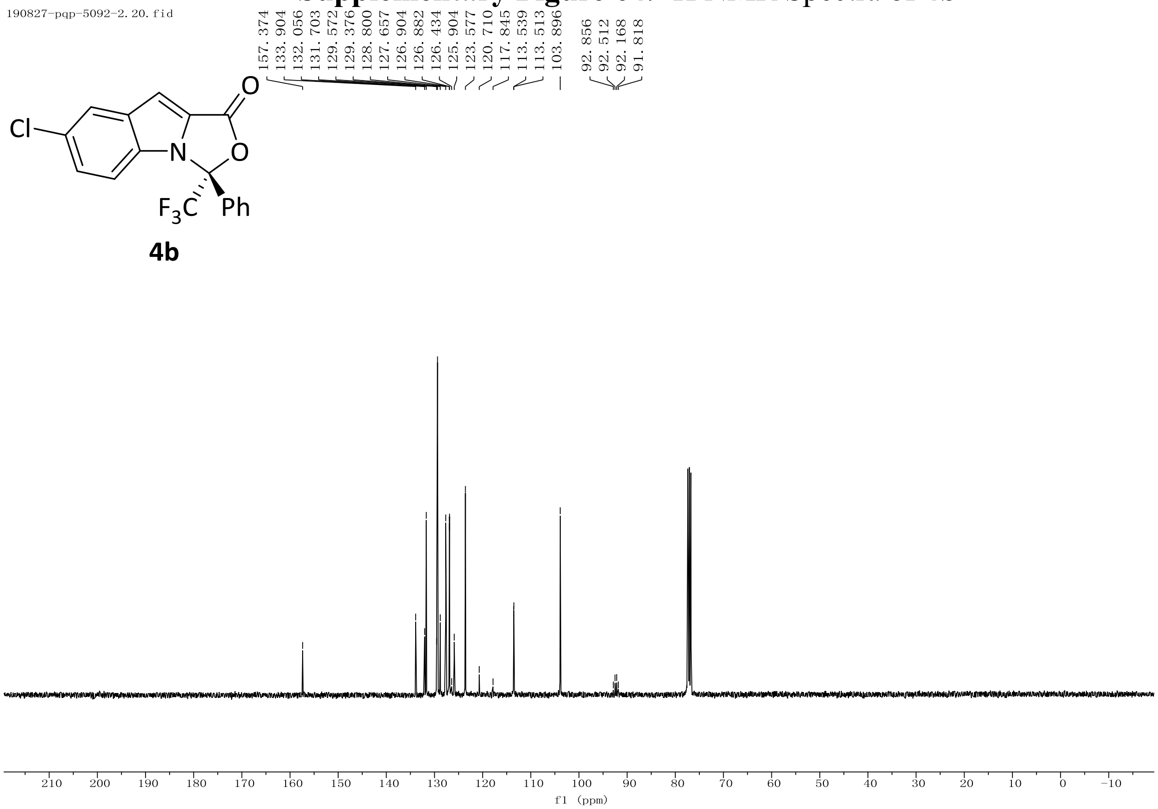

Supplementary Figure 65. ^{13}C NMR Spectra of **4b**

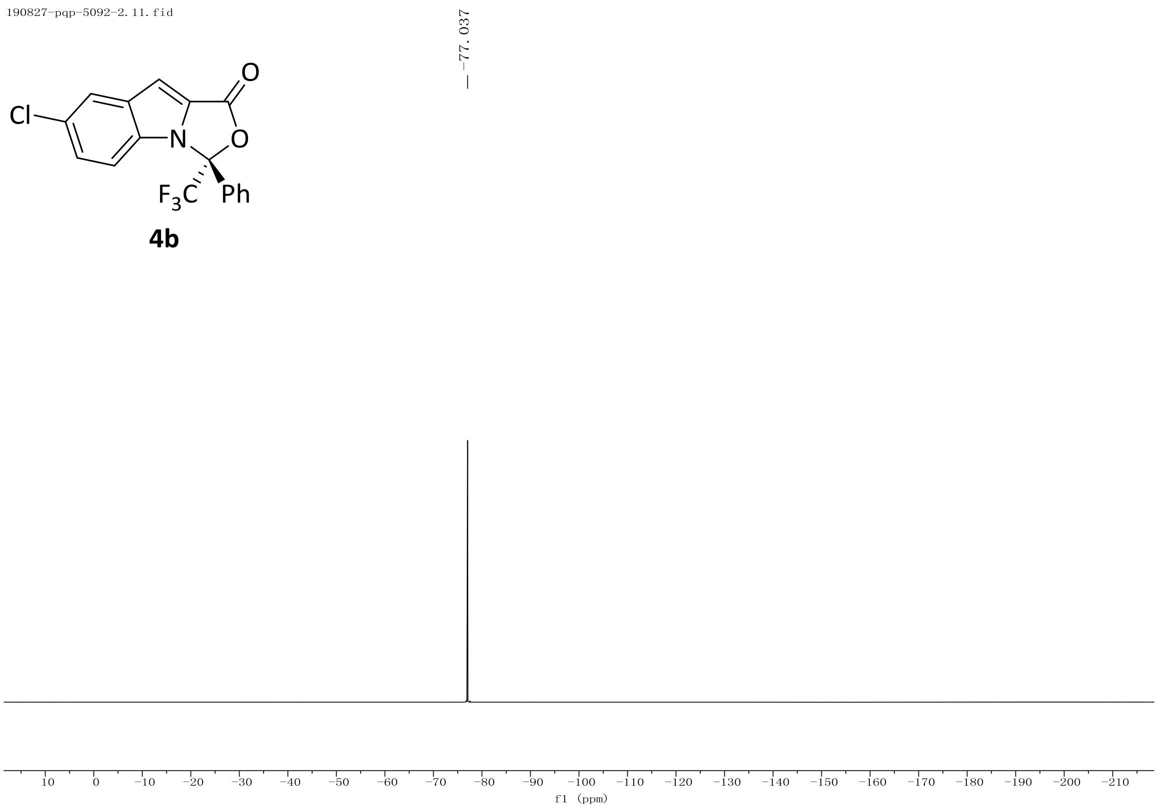

Supplementary Figure 66. ^{19}F NMR Spectra of **4b**

4c (*R*)-7-methyl-3-phenyl-3-(trifluoromethyl)-1*H*,3*H*-oxazolo[3,4-a]indol-1-one

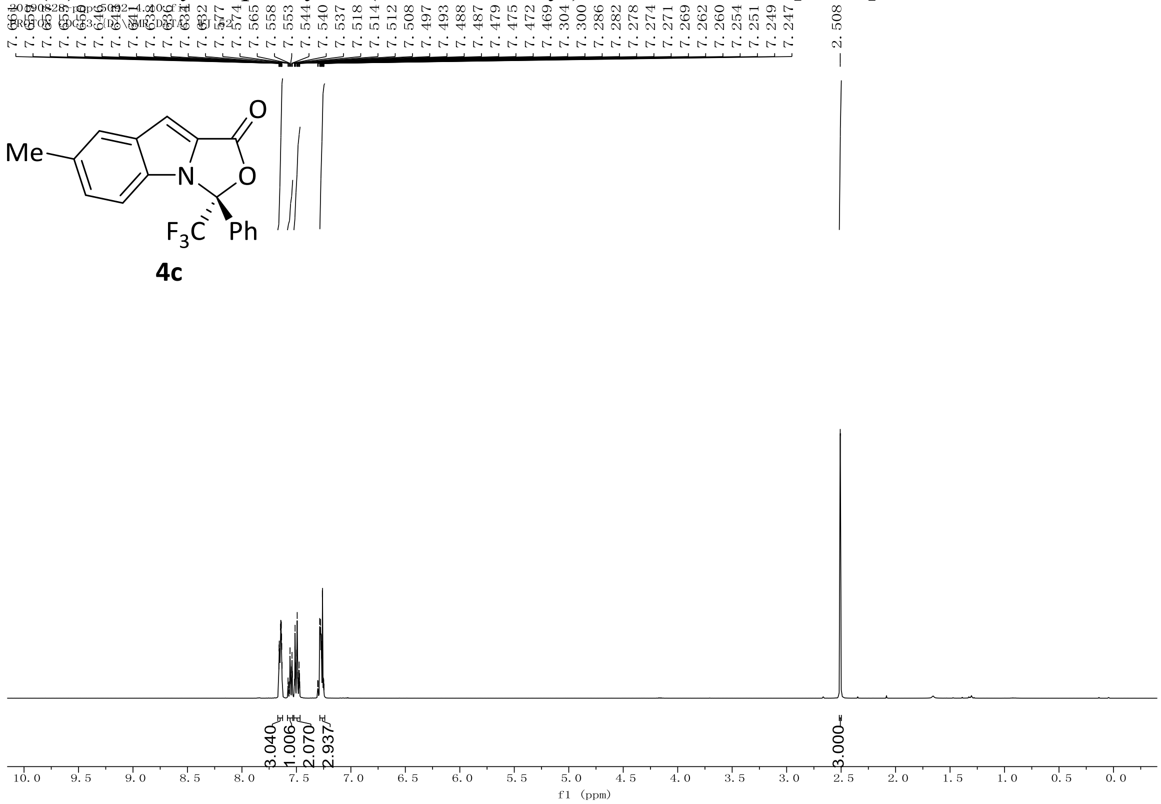

Supplementary Figure 67. ^{1}H NMR Spectra of **4c**

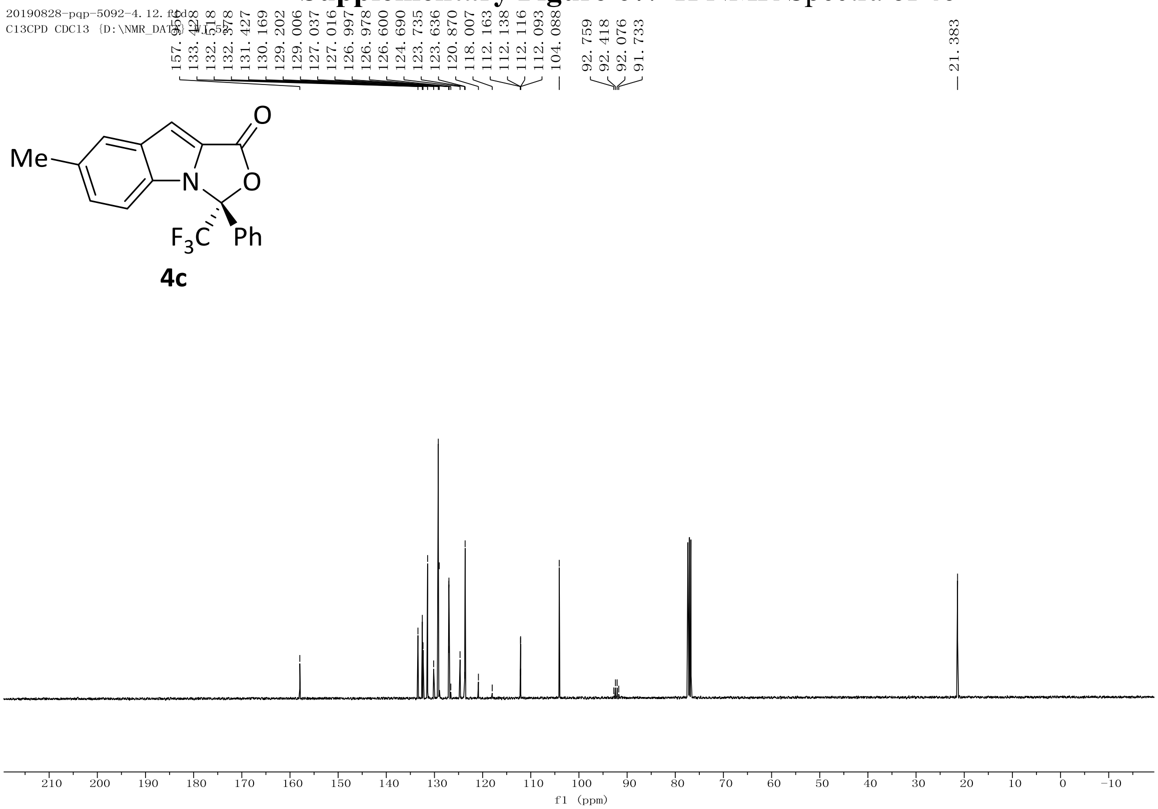

Supplementary Figure 68. ^{13}C NMR Spectra of **4c**

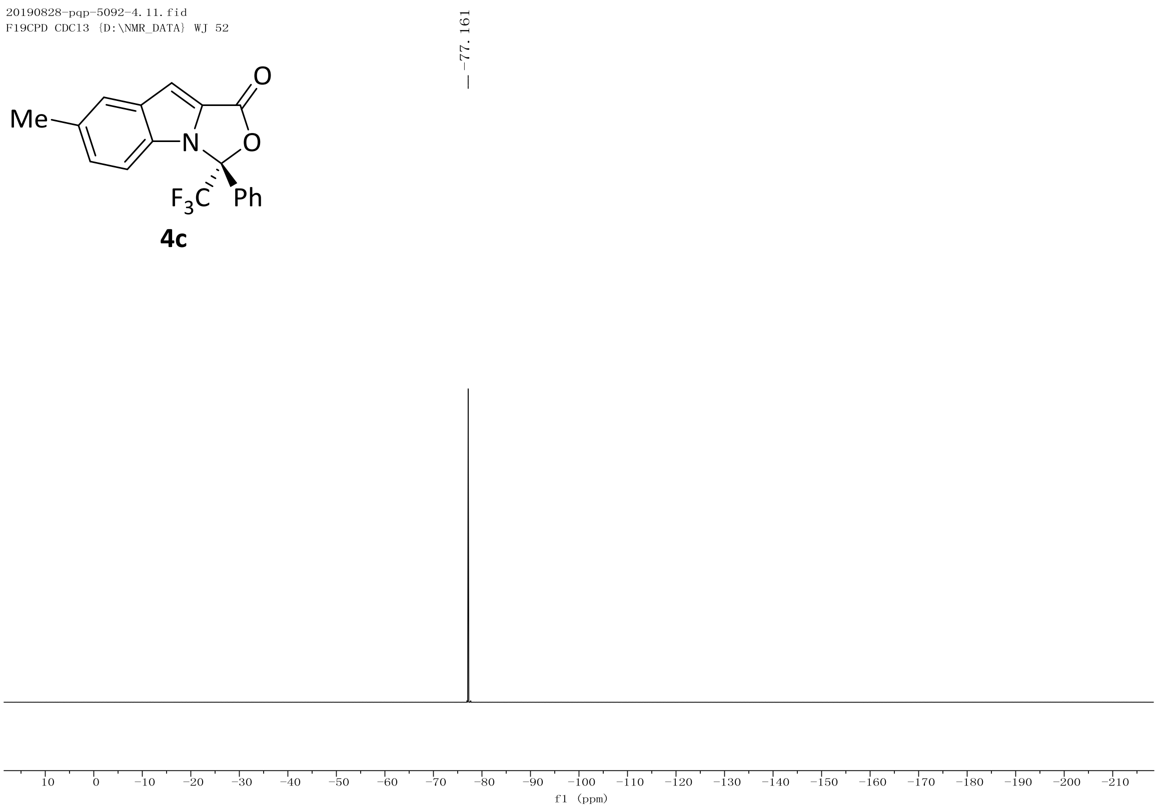

Supplementary Figure 69. ^{19}F NMR Spectra of **4c**

4d (*R*)-7-methoxy-3-phenyl-3-(trifluoromethyl)-1*H*,3*H*-oxazolo[3,4-a]indol-1-one

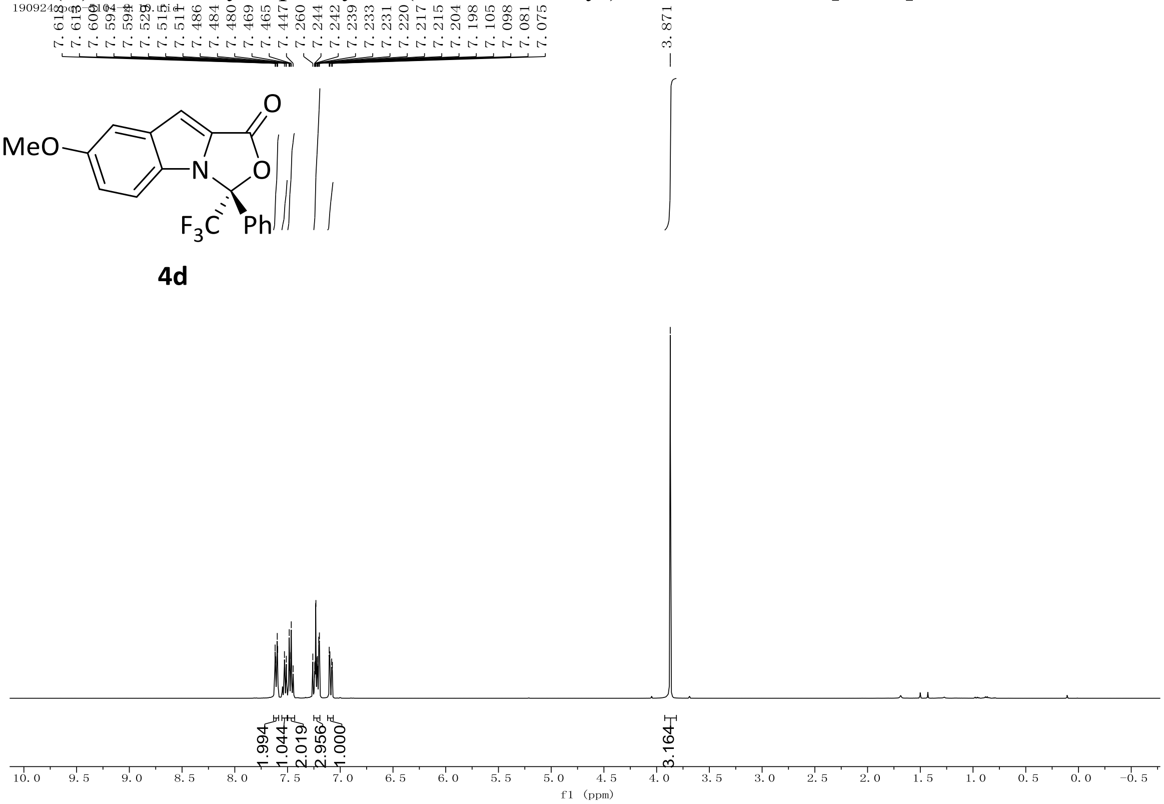

Supplementary Figure 70. ^{1}H NMR Spectra of **4d**

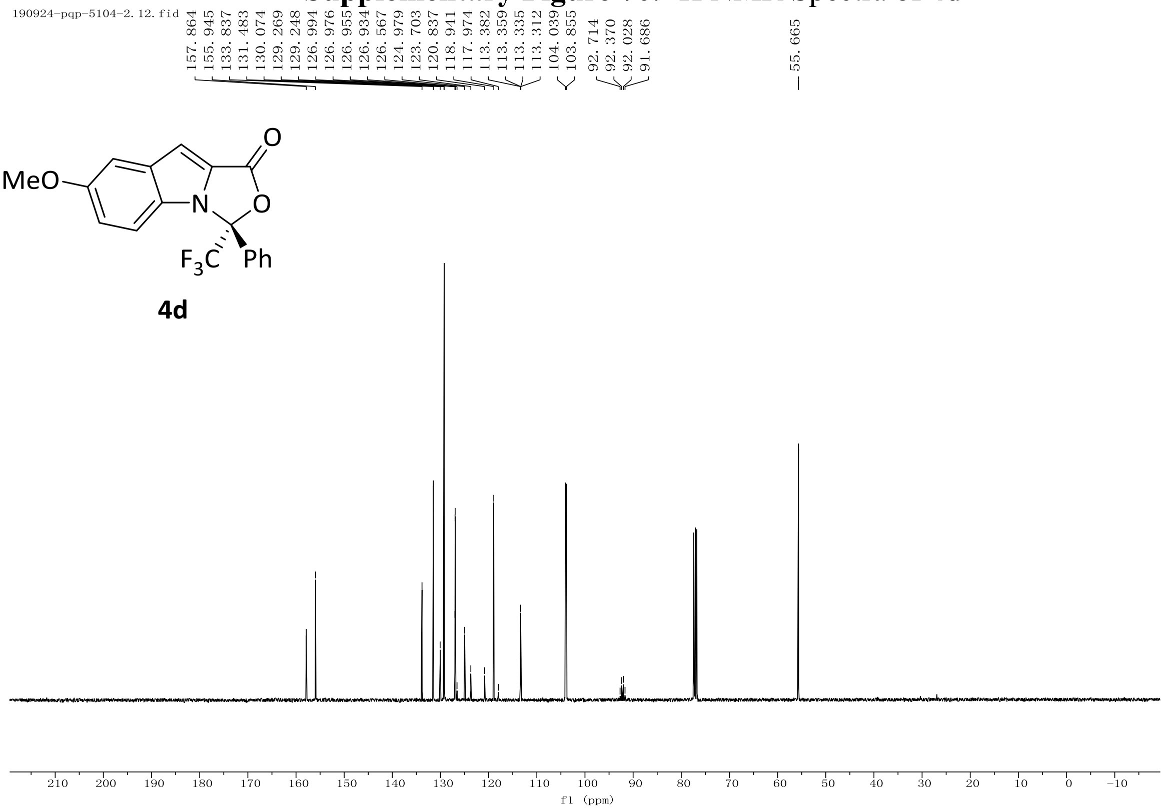

Supplementary Figure 71. ^{13}C NMR Spectra of **4d**

190924-pqp-5104-2.11.fid

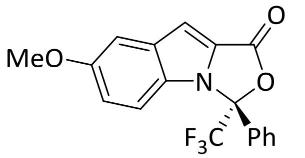

4d

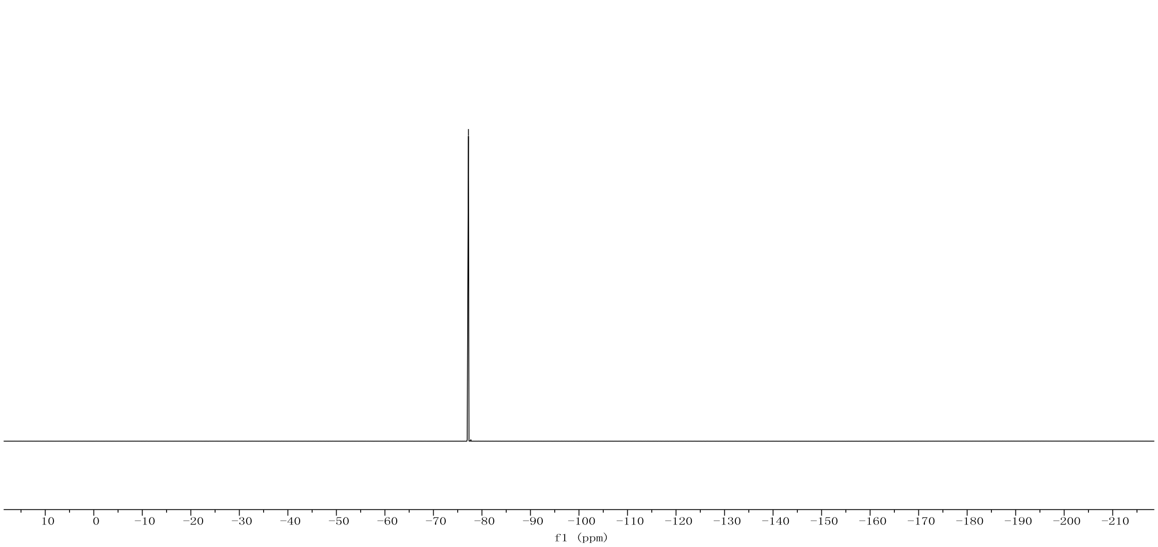

Supplementary Figure 72. ^{19}F NMR Spectra of **4d**

4e (*R*)-9-methyl-3-phenyl-3-(trifluoromethyl)-1*H*,3*H*-oxazolo[3,4-a]indol-1-one

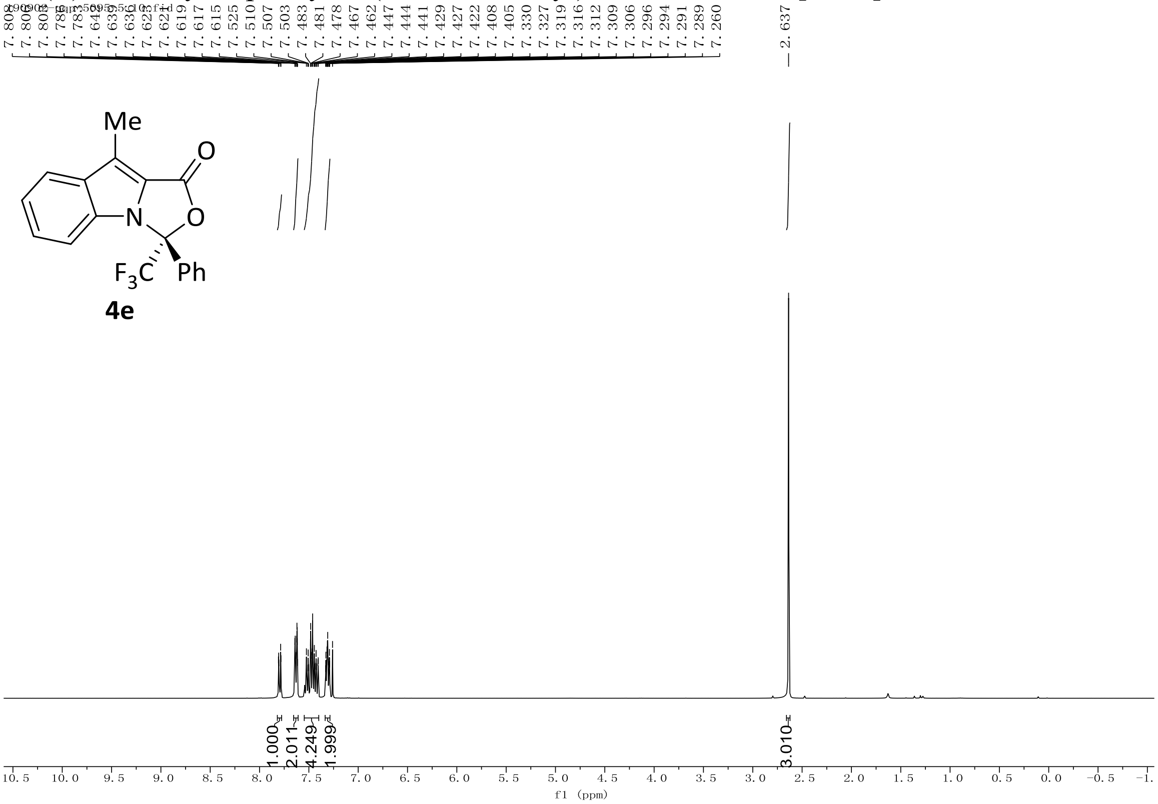

Supplementary Figure 73. ^{1}H NMR Spectra of **4e**

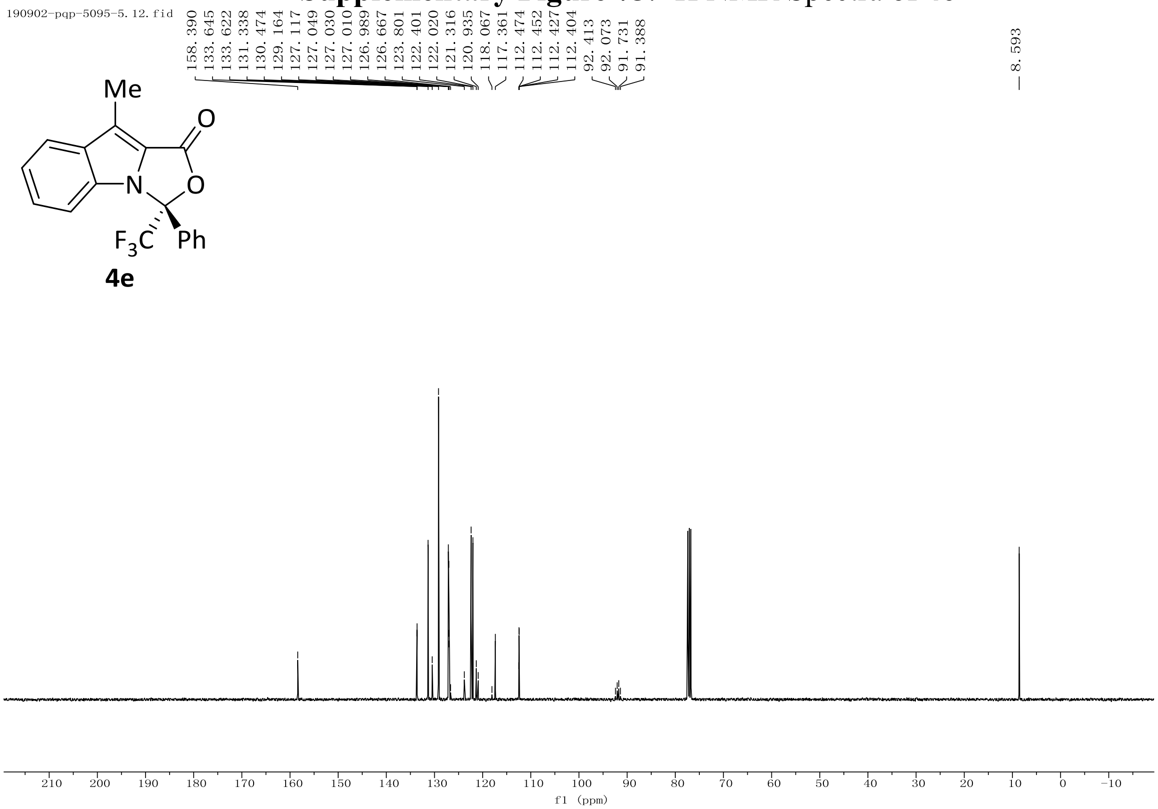

Supplementary Figure 74. ^{13}C NMR Spectra of **4e**

190902-pqp-5095-5.11.fid

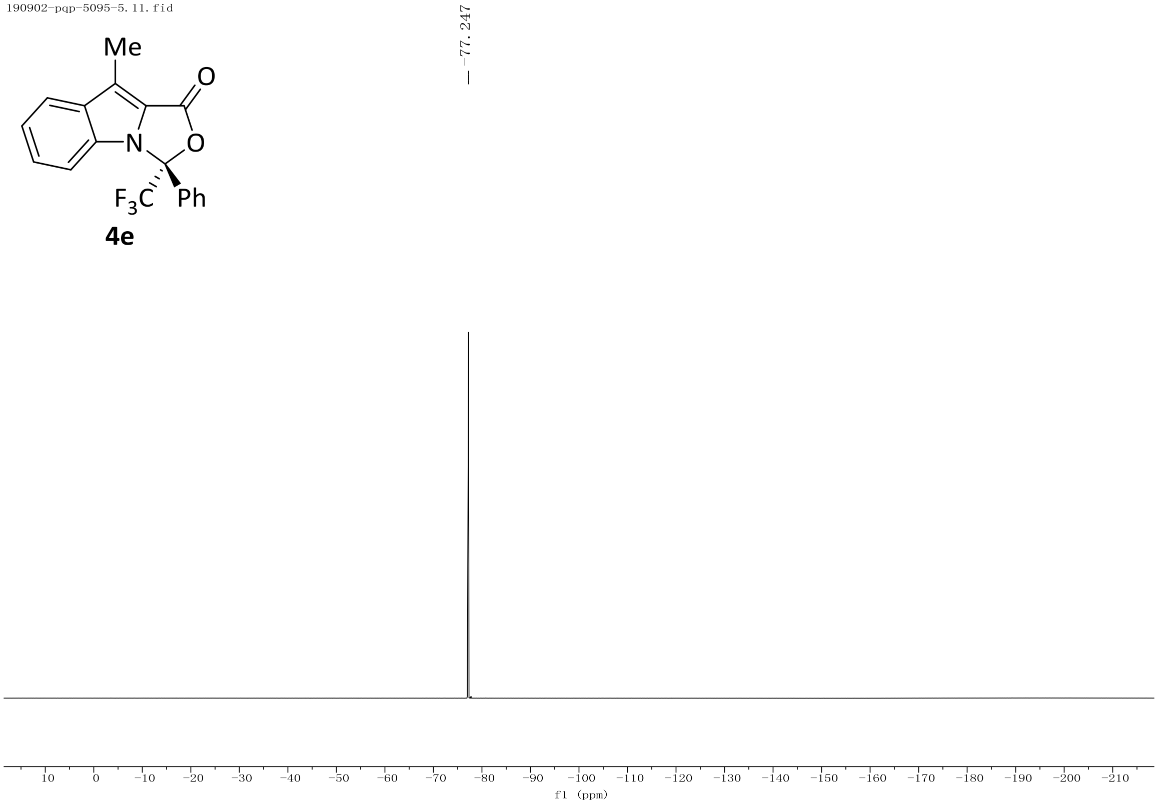

Supplementary Figure 75. ^{19}F NMR Spectra of **4e**

4f (*R*)-8-methyl-3-phenyl-3-(trifluoromethyl)-1*H*,3*H*-oxazolo[3,4-a]indol-1-one

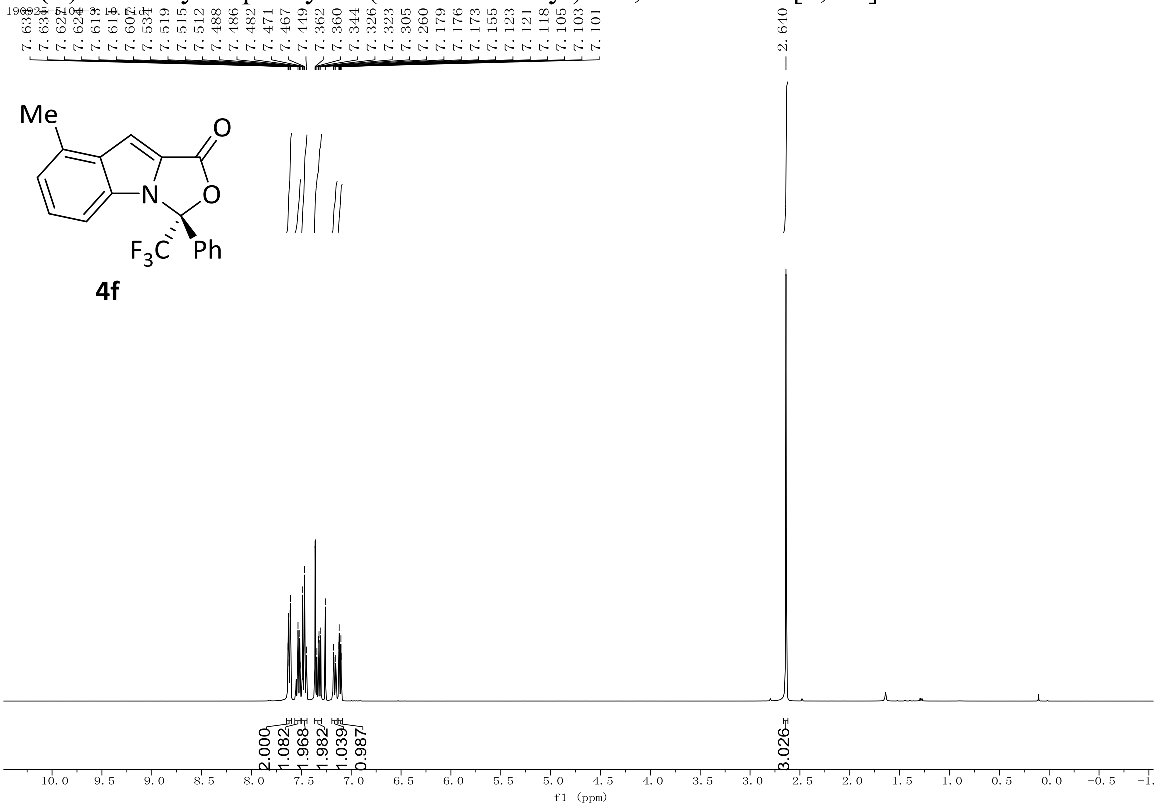

Supplementary Figure 76. ^{1}H NMR Spectra of **4f**

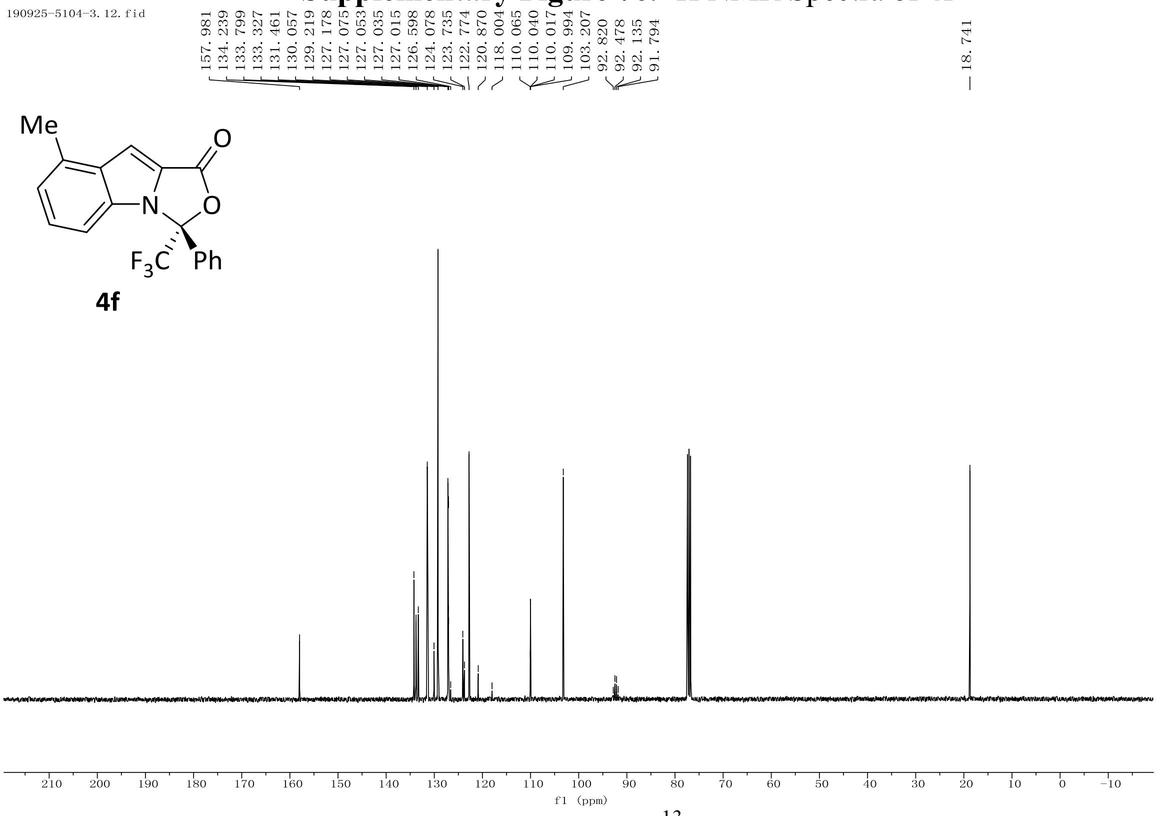

Supplementary Figure 77. ^{13}C NMR Spectra of **4f**

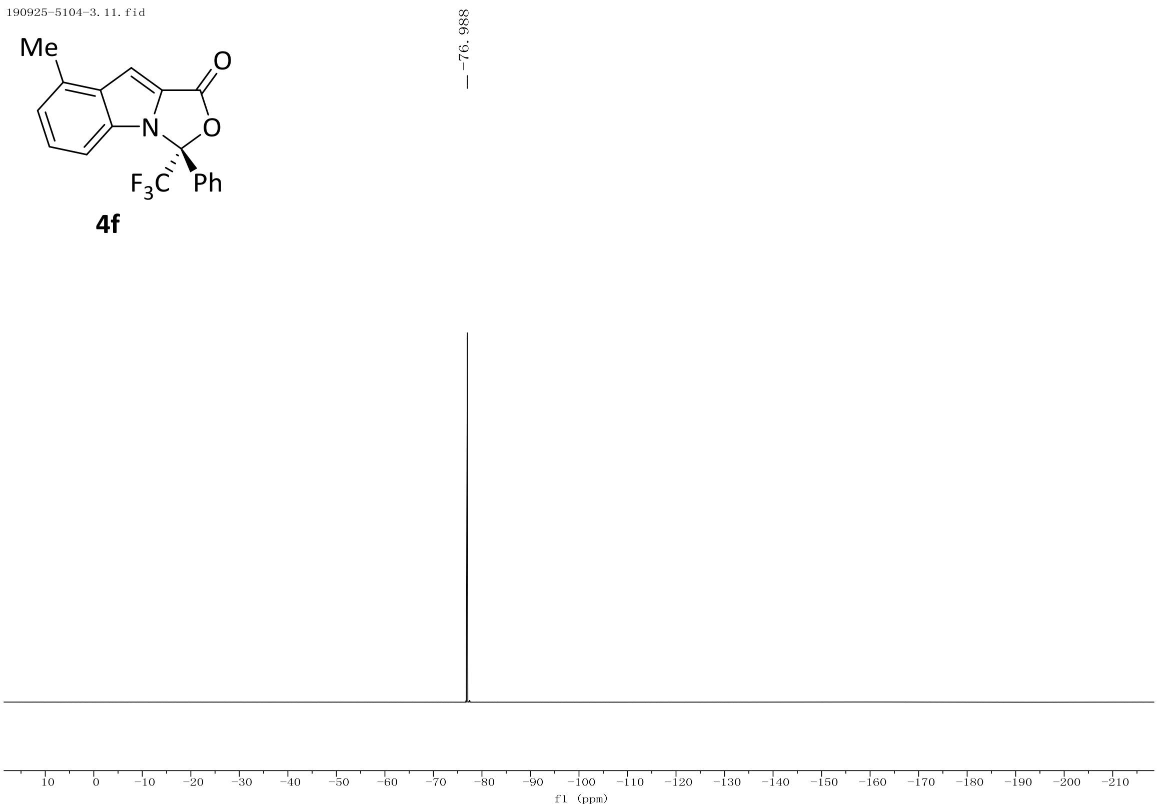

Supplementary Figure 78. ^{19}F NMR Spectra of **4f**

4g (*R*)-6-methyl-3-phenyl-3-(trifluoromethyl)-1*H*,3*H*-oxazolo[3,4-a]indol-1-one

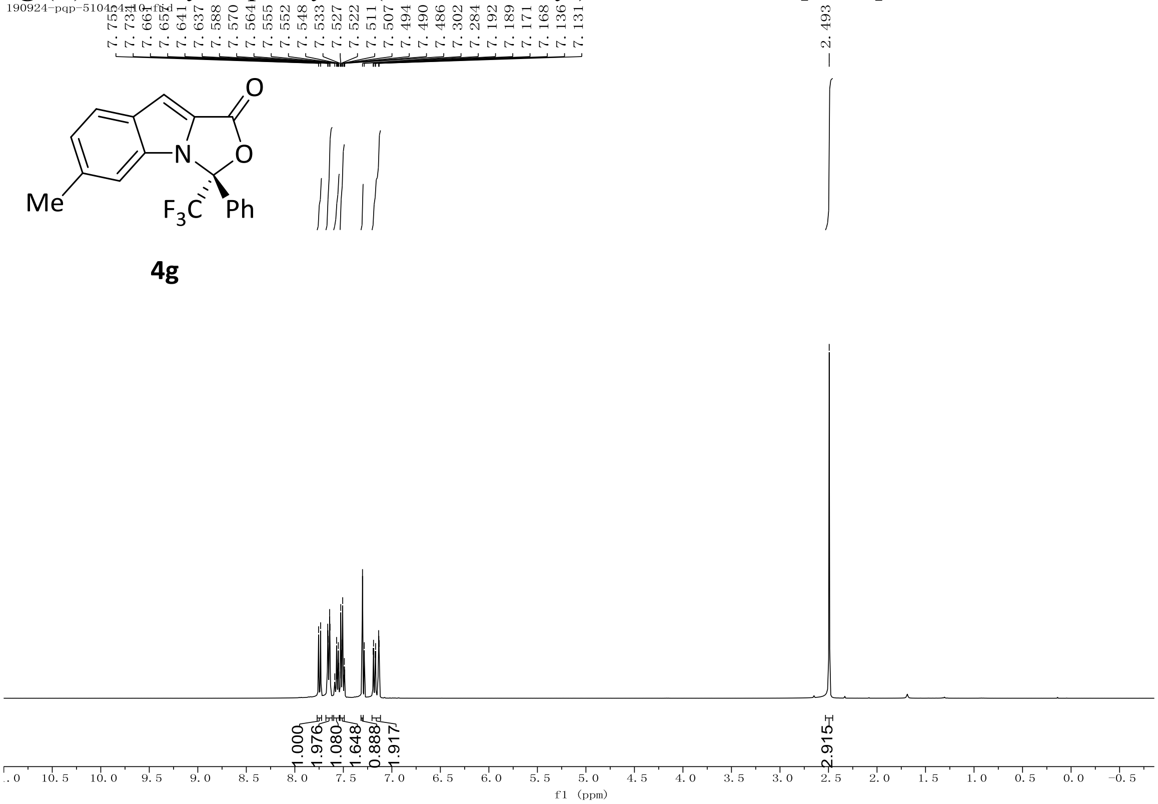

Supplementary Figure 79. ^{1}H NMR Spectra of **4g**

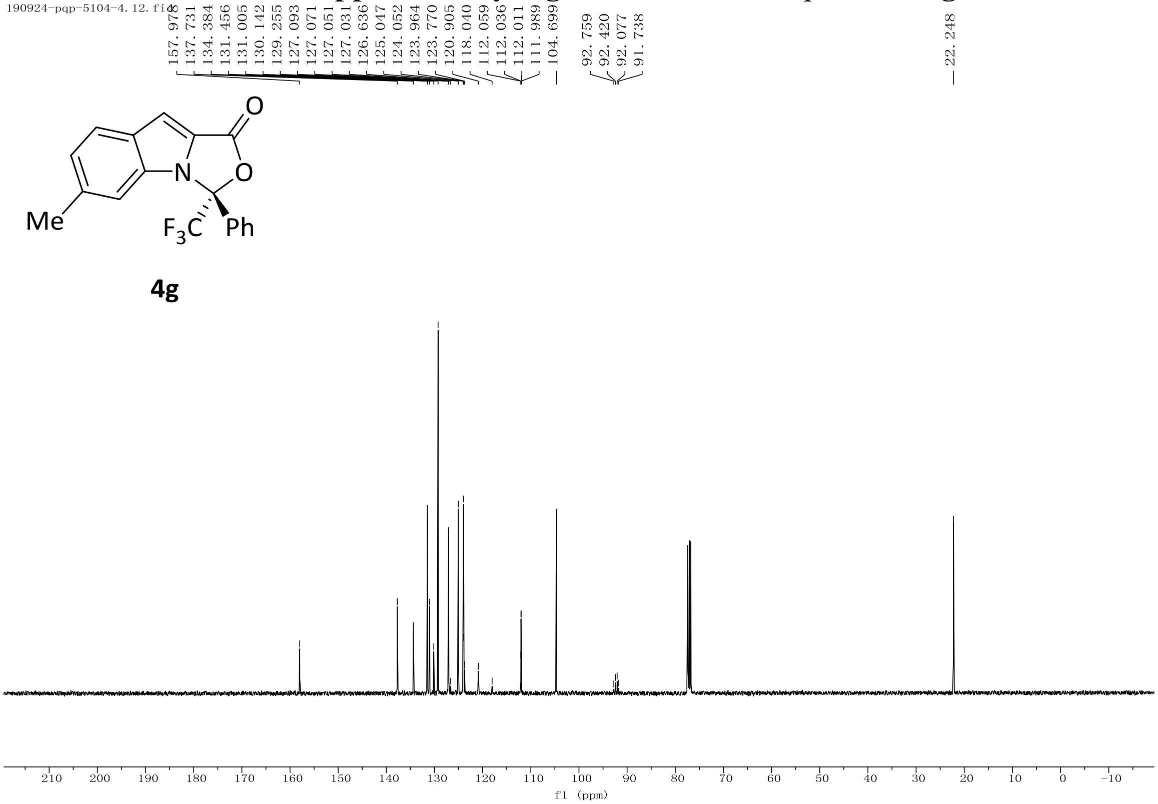

Supplementary Figure 80. ^{13}C NMR Spectra of **4g**

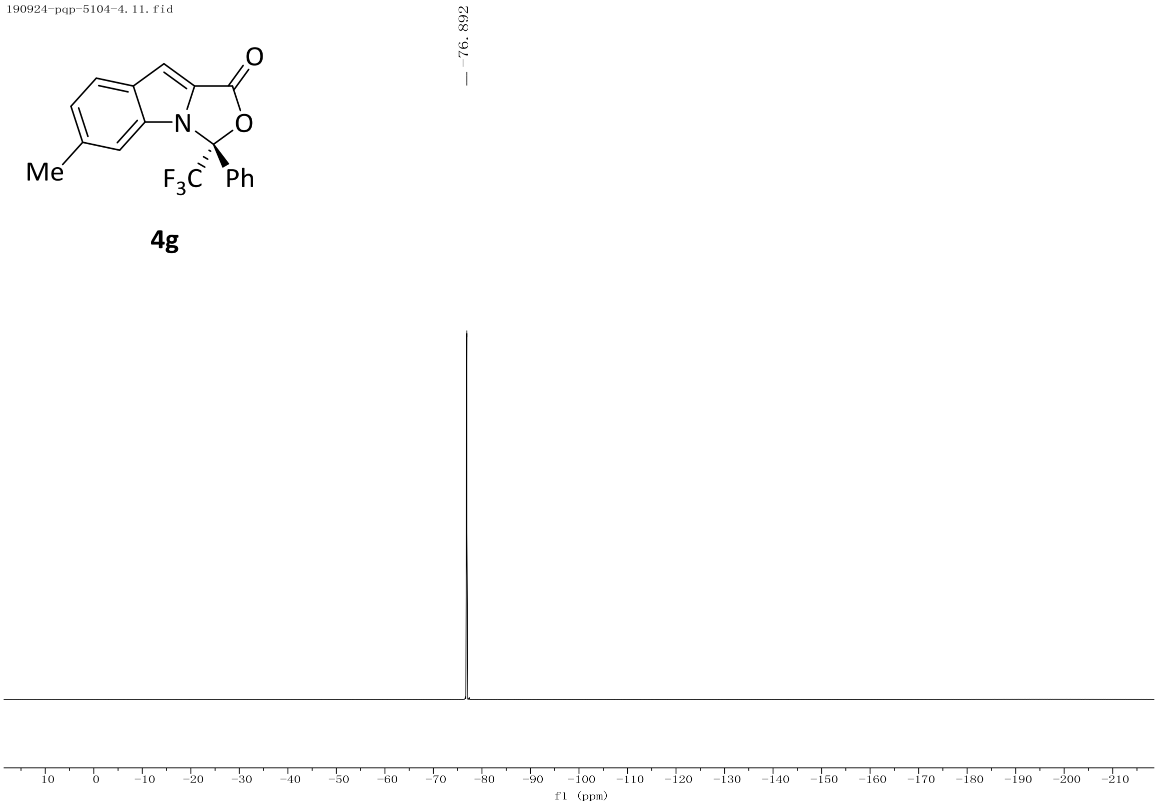

Supplementary Figure 81. ^{19}F NMR Spectra of **4g**

4h (*R*)-8-phenyl-8-(trifluoromethyl)-8*H*,10*H*-benzo[e]oxazolo[3,4-a]indol-10-one

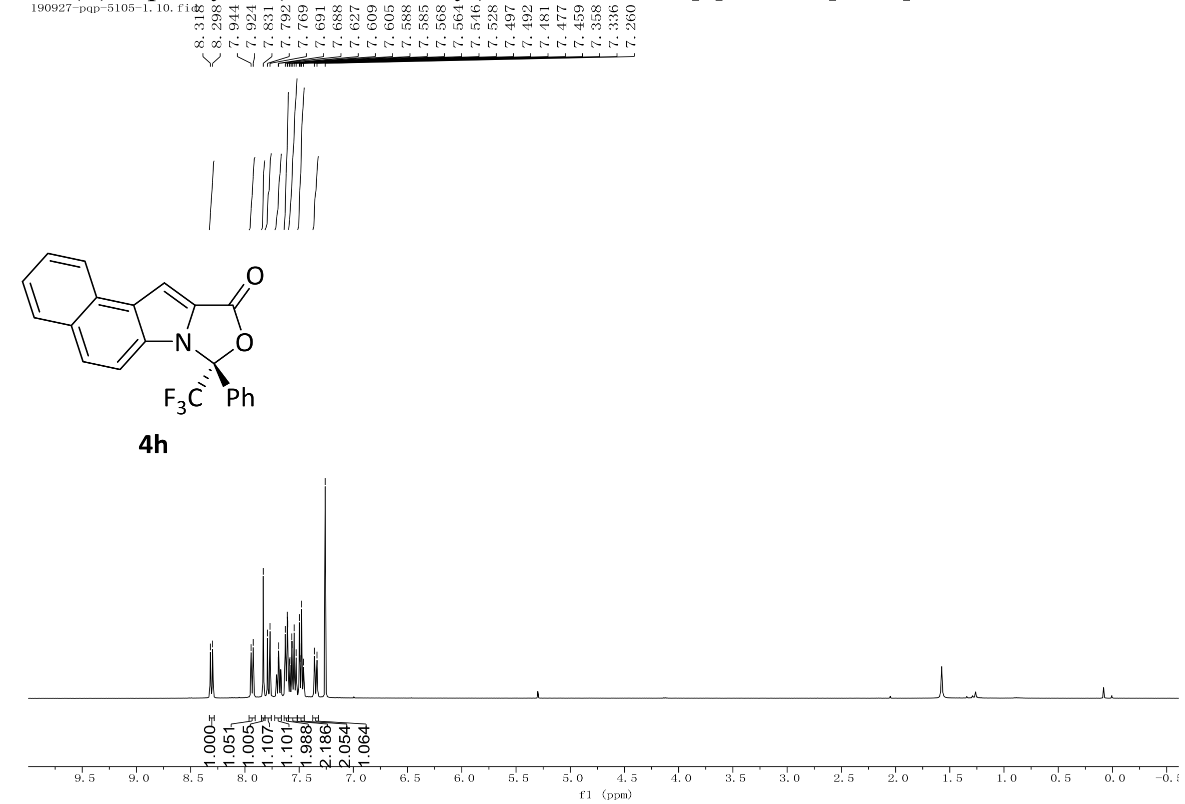

Supplementary Figure 82. ^{1}H NMR Spectra of **4h**

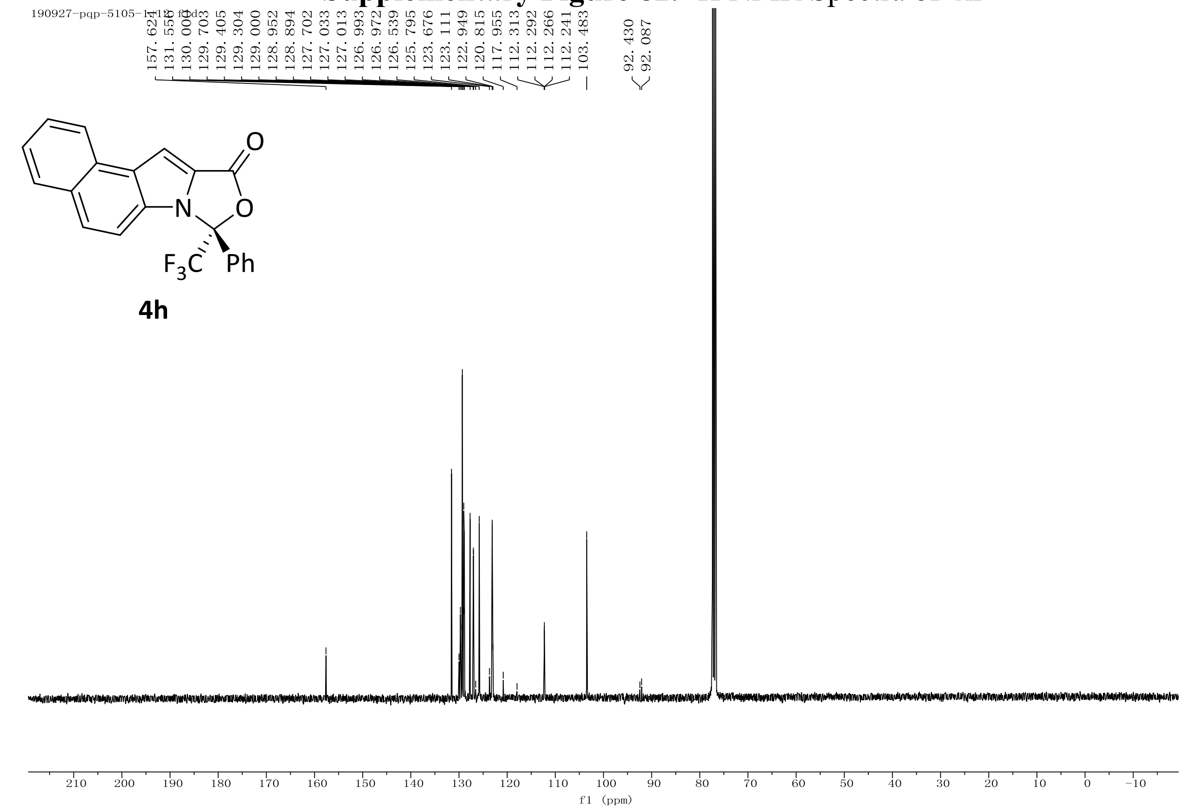

Supplementary Figure 83. ^{13}C NMR Spectra of **4h**

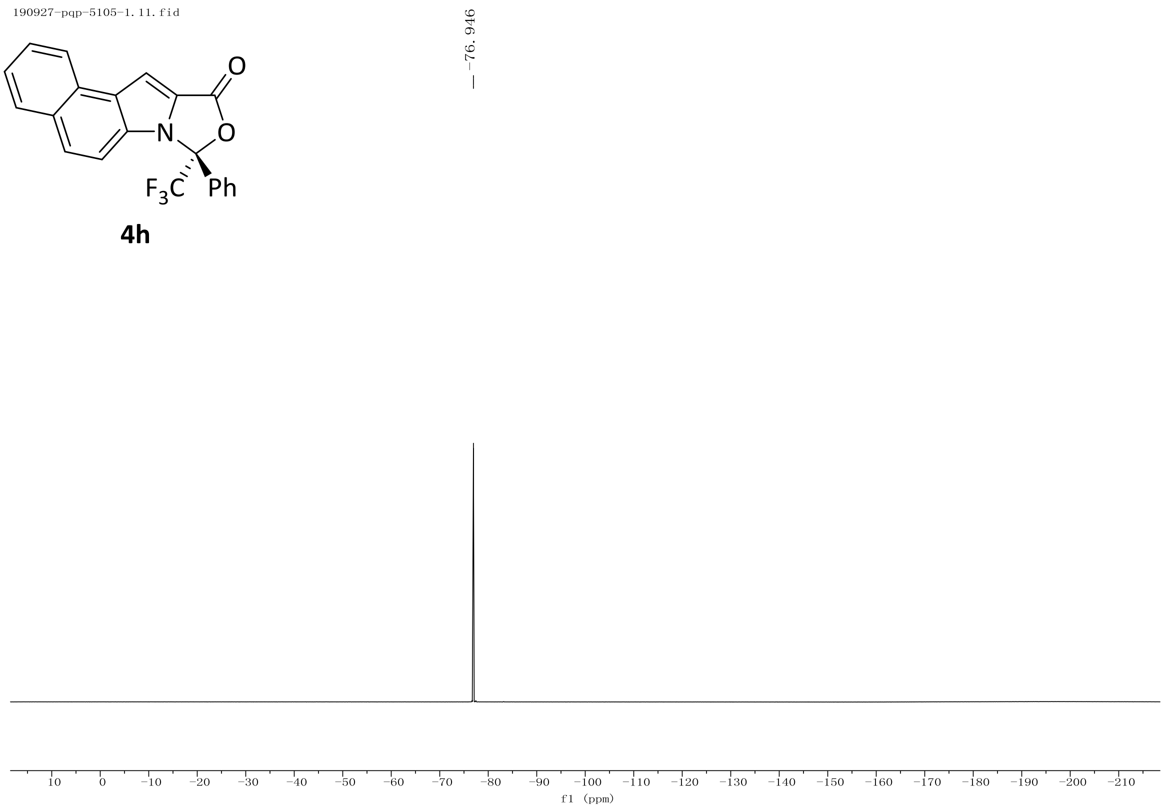

Supplementary Figure 84. ^{19}F NMR Spectra of **4h**

4i (*R*)-3-methyl-7-phenyl-7-(trifluoromethyl)-7*H*-oxazolo[3,4-a]pyrrolo[3,2-e]indol-9(3*H*)-one

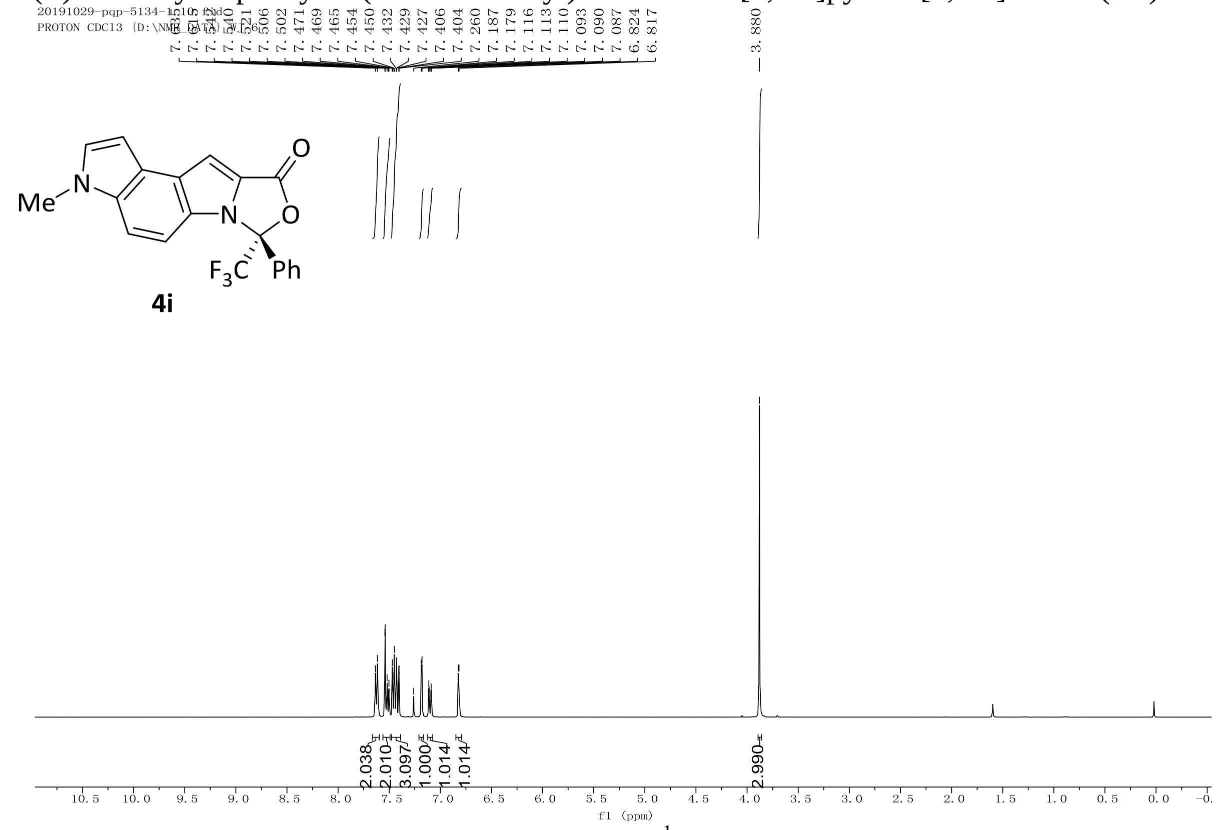

Supplementary Figure 85. ^{1}H NMR Spectra of **4i**

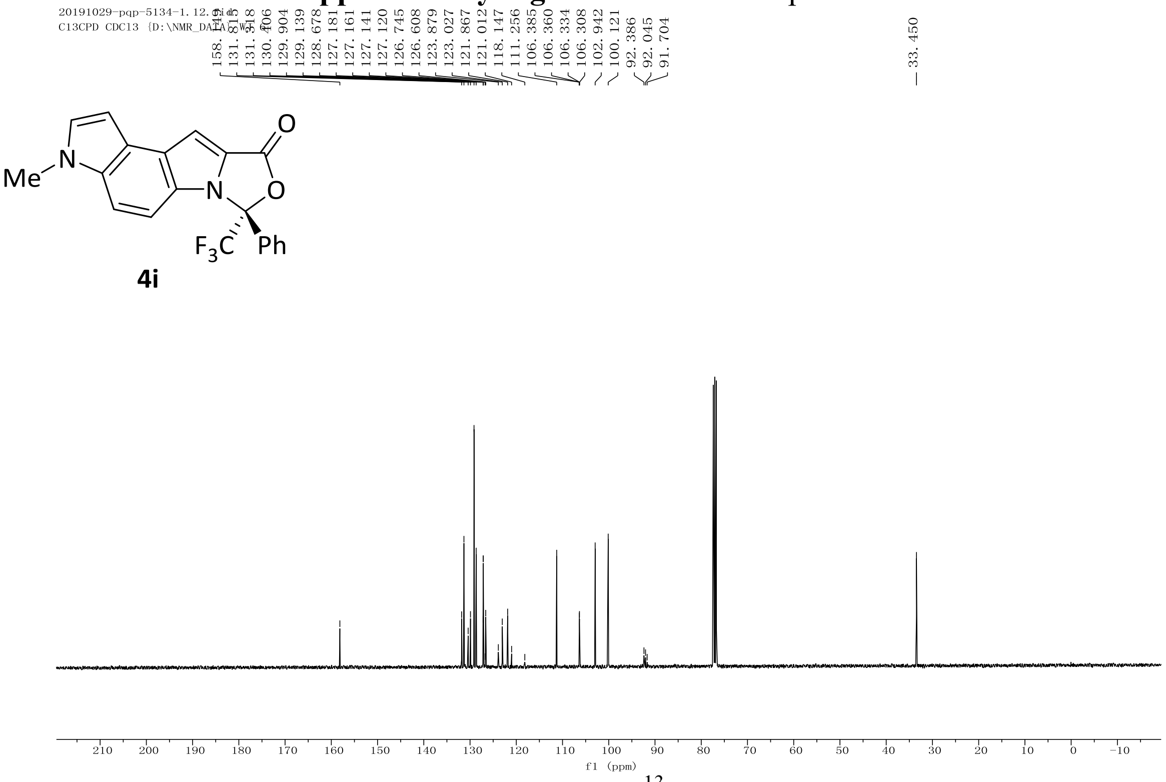

Supplementary Figure 86. ^{13}C NMR Spectra of **4i**

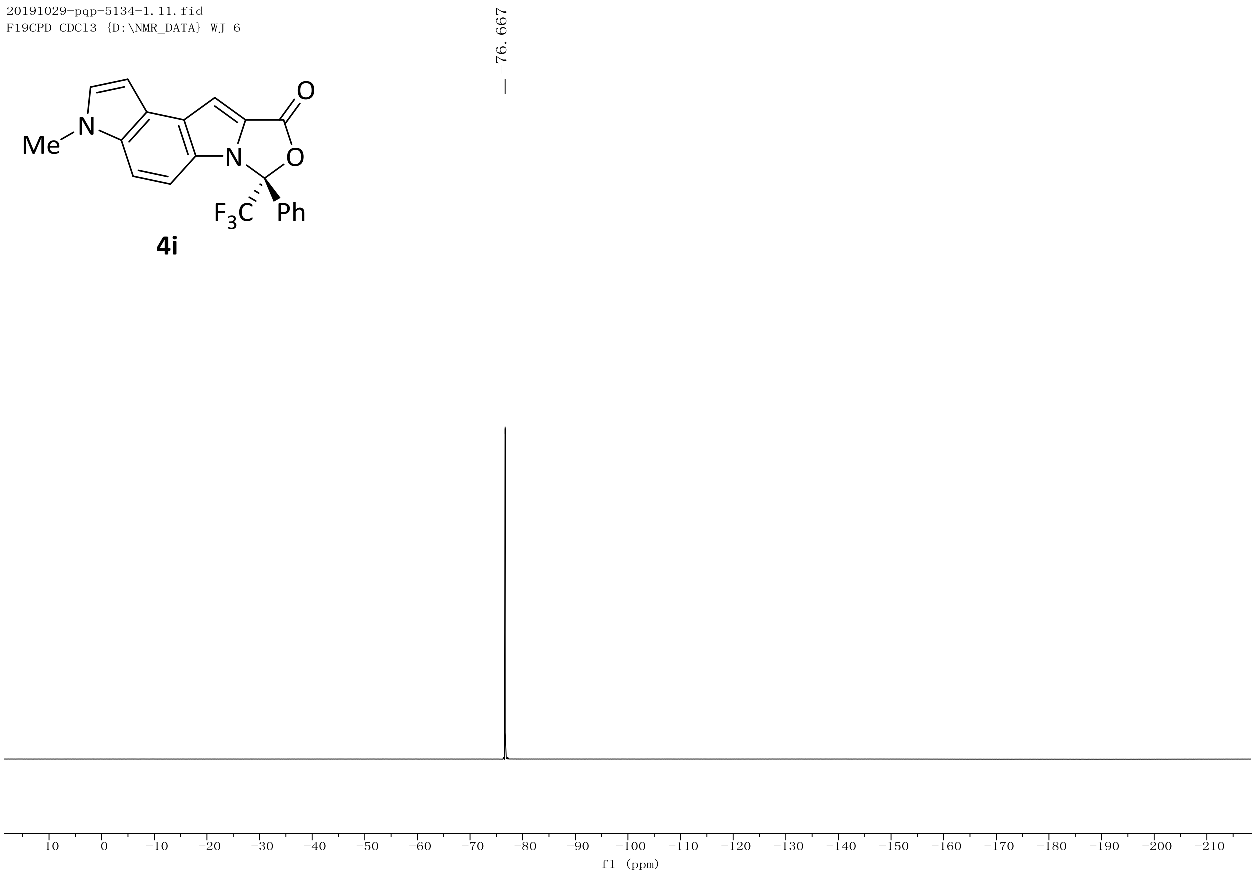

Supplementary Figure 87. ^{19}F NMR Spectra of **4i**

6 (*R*)-3-(4'-methoxy-[1,1'-biphenyl]-4-yl)-3-(trifluoromethyl)-1*H*,3*H*-oxazolo[3,4-a]indol-1-one

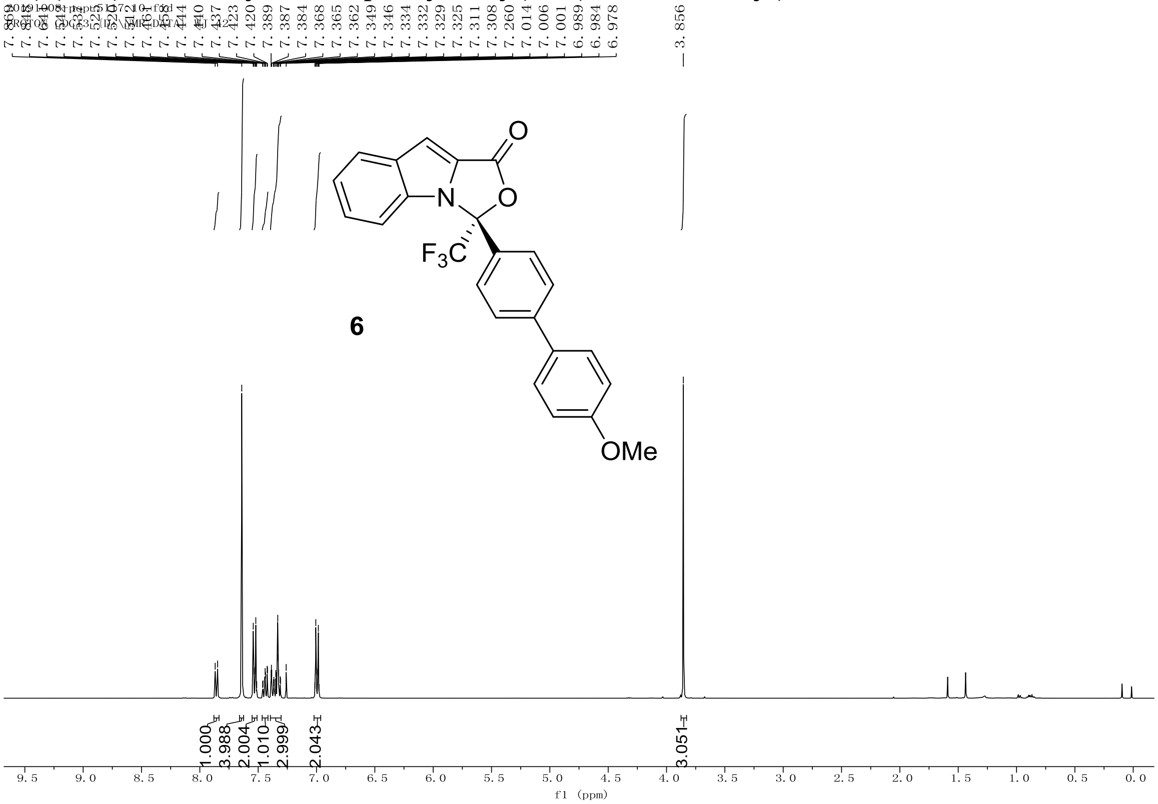

Supplementary Figure 88. ^{1}H NMR Spectra of **6**

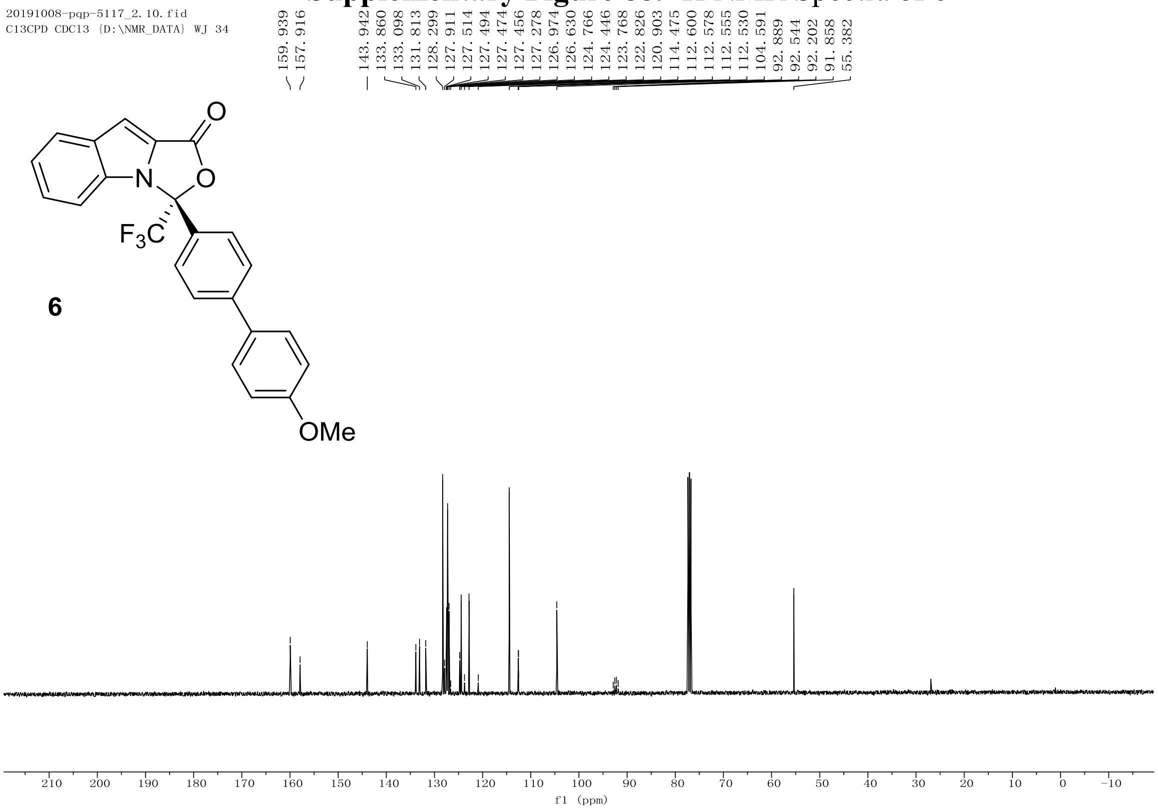

Supplementary Figure 89. ^{13}C NMR Spectra of **6**

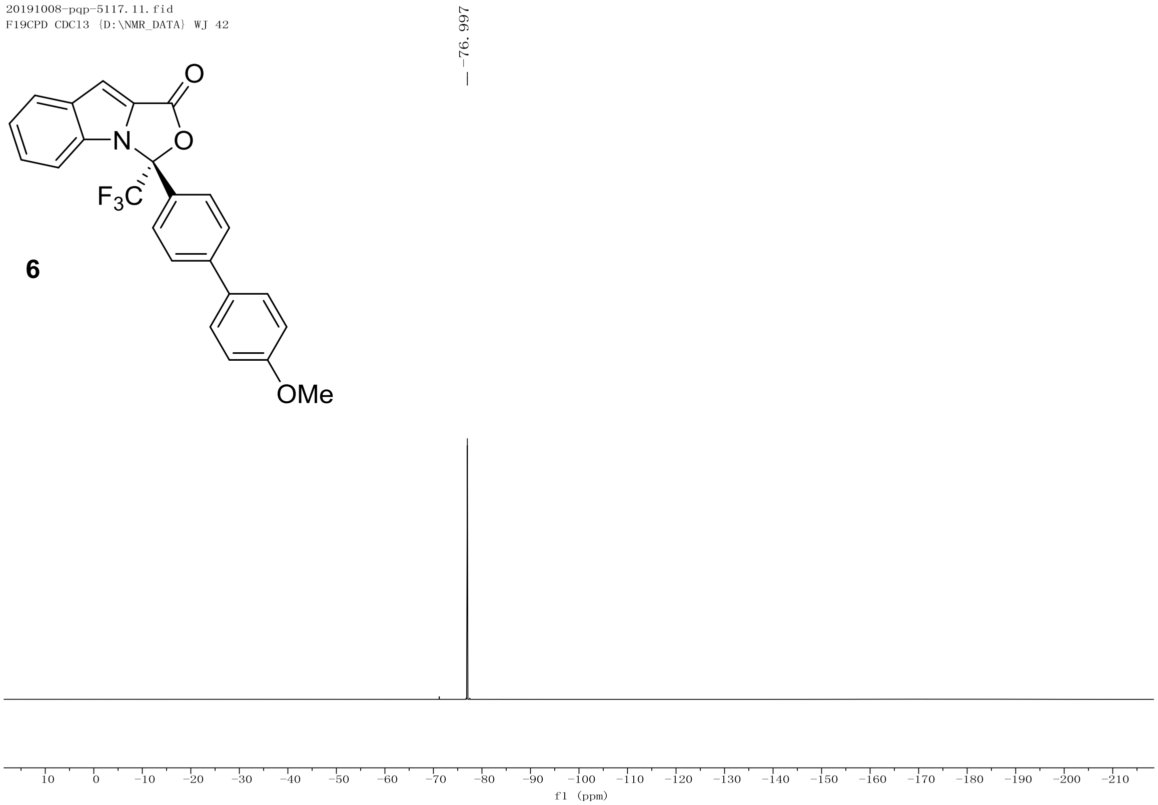

Supplementary Figure 90. ^{19}F NMR Spectra of **6**

F (5a*S*,10b*R*)-9-nitro-2-(perfluorophenyl)-5a,10b-dihydro-4*H*,6*H*-indeno[2,1-*b*][1,2,4]triazolo[4,3-*d*][1,4]oxazin-2-ium tetrafluoroborate

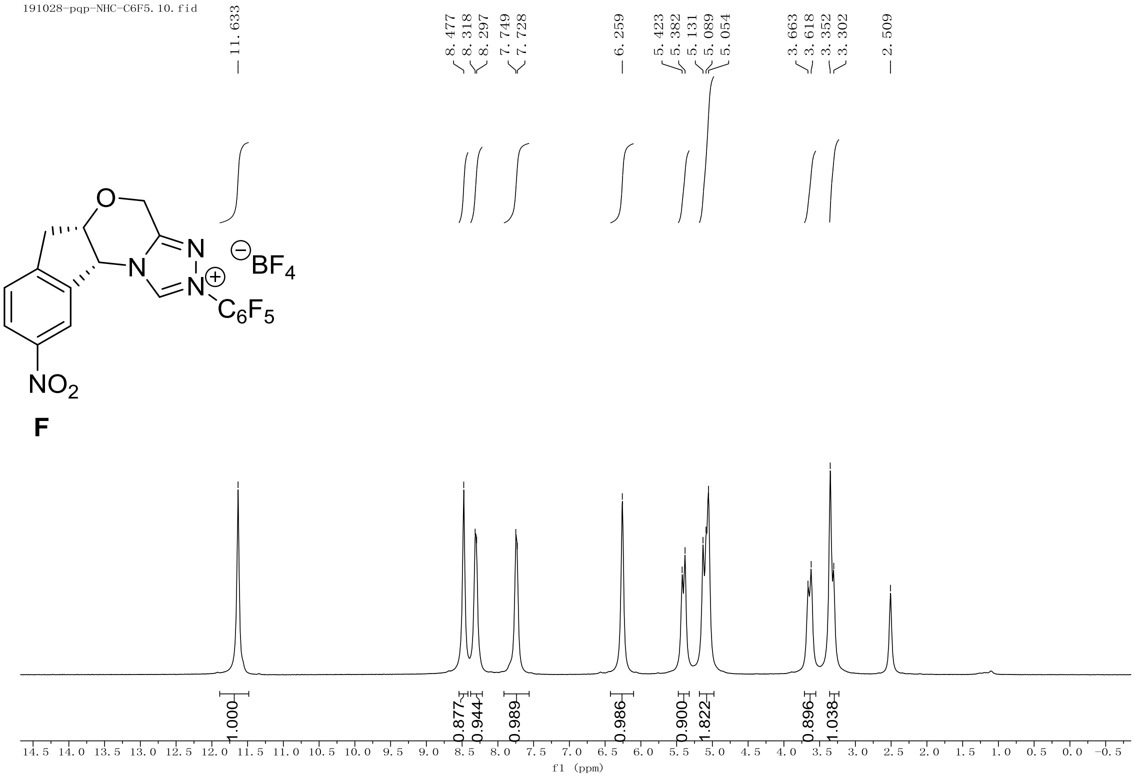

Supplementary Figure 91. ^{1}H NMR Spectra of **F**

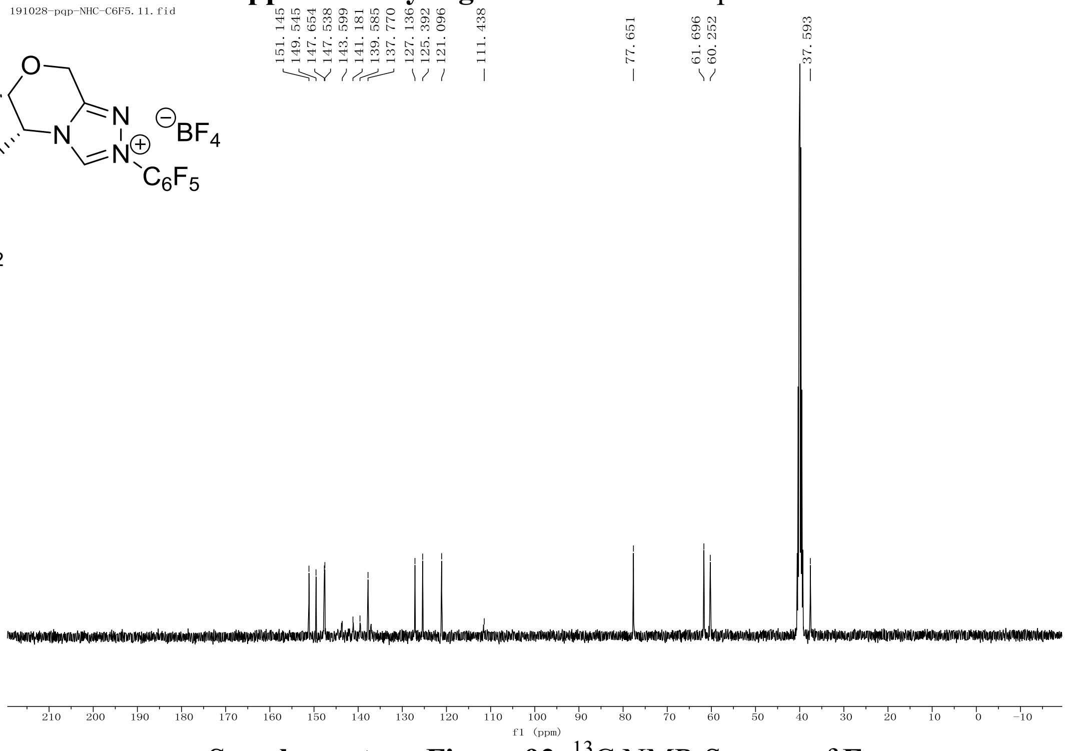

Supplementary Figure 92. ^{13}C NMR Spectra of **F**

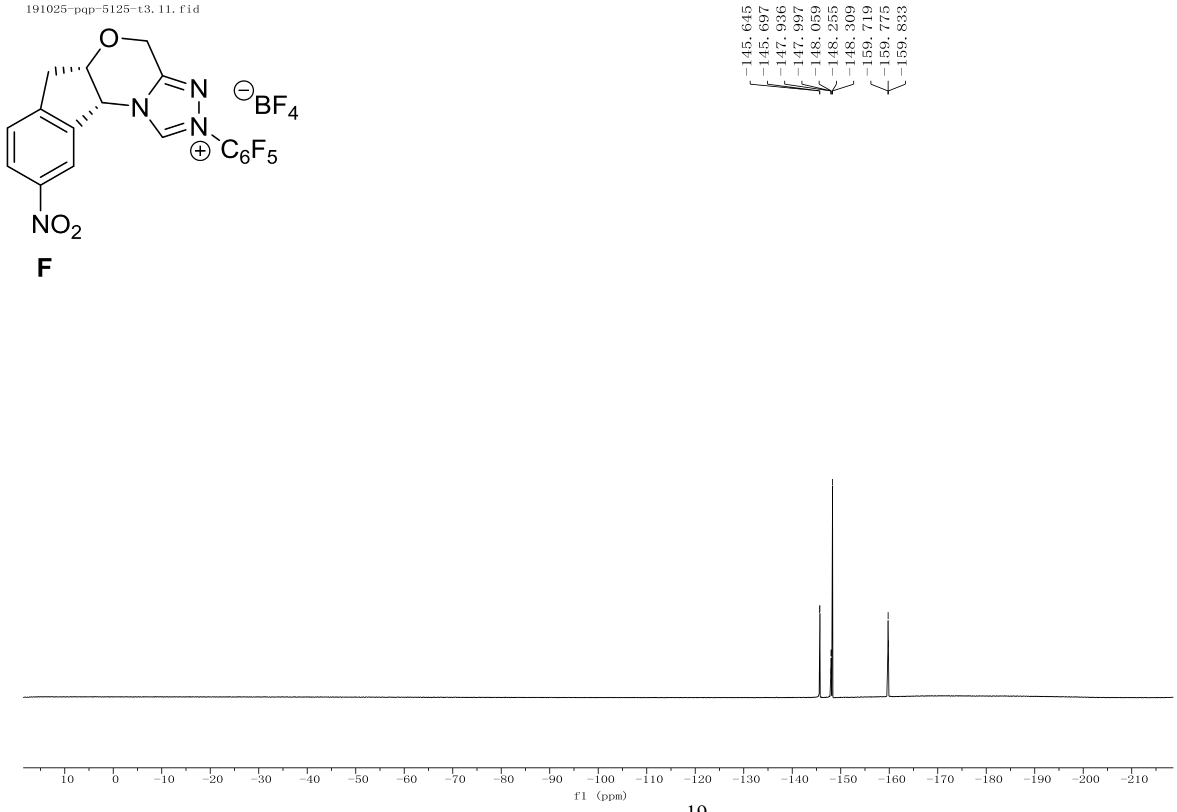

Supplementary Figure 93. ^{19}F NMR Spectra of **F**

HPLC Spectra

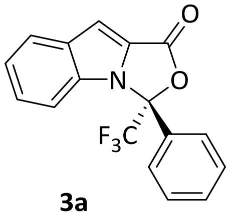

3a (*R*)-3-phenyl-3-(trifluoromethyl)-1*H*,3*H*-oxazolo[3,4-a]indol-1-one

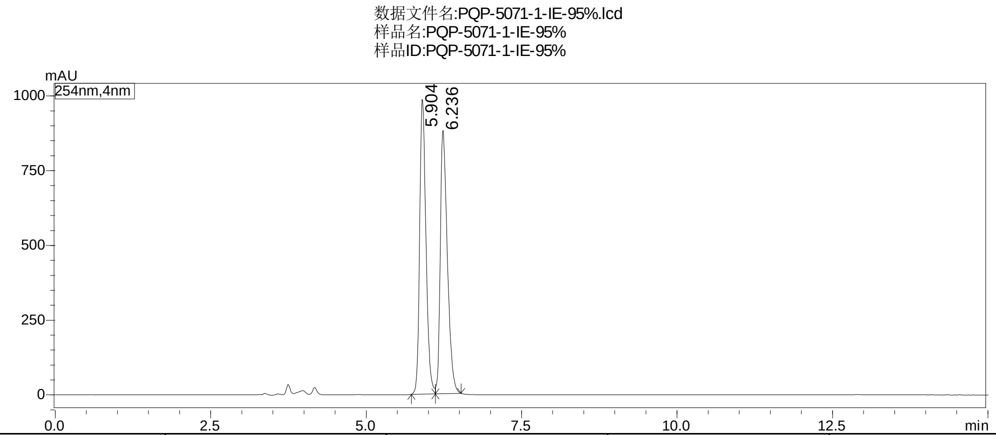

Peak	Ret. Time	Area	Height	Area%
1	5.904	6983485	985666	49.956
2	6.236	6995912	880522	50.044
Total		13979398	1866189	100.000

Supplementary Figure 94. HPLC Spectrum of racemic **3a**

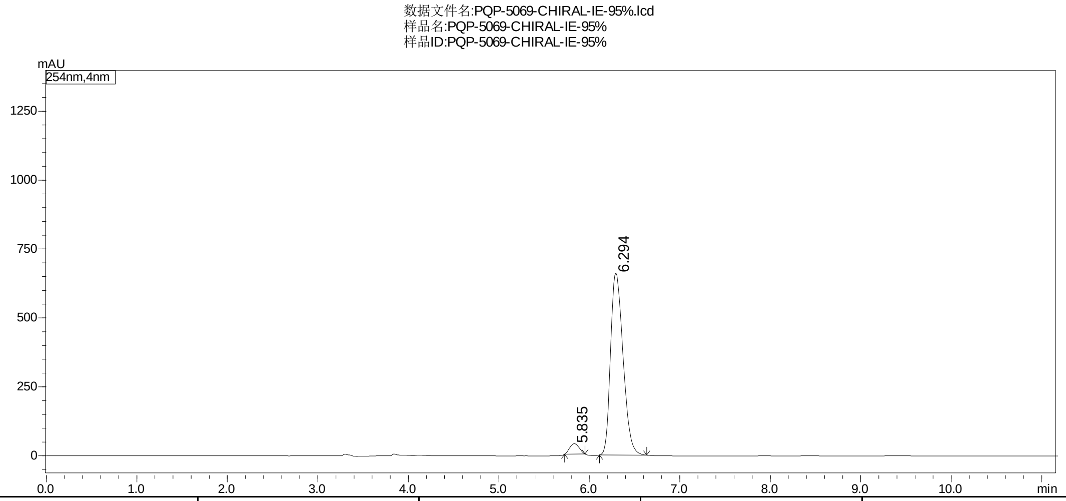

Peak	Ret. Time	Area	Height	Area%
1	5.835	276982	37961	4.179
2	6.294	6350746	661113	95.821
Total		6627728	699074	100.000

Supplementary Figure 95. HPLC Spectrum of **3a**

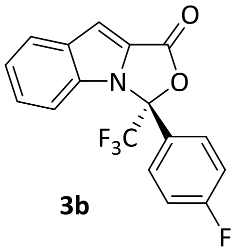

3b

3b (*R*)-3-(4-fluorophenyl)-3-(trifluoromethyl)-1*H*,3*H*-oxazolo[3,4-a]indol-1-one

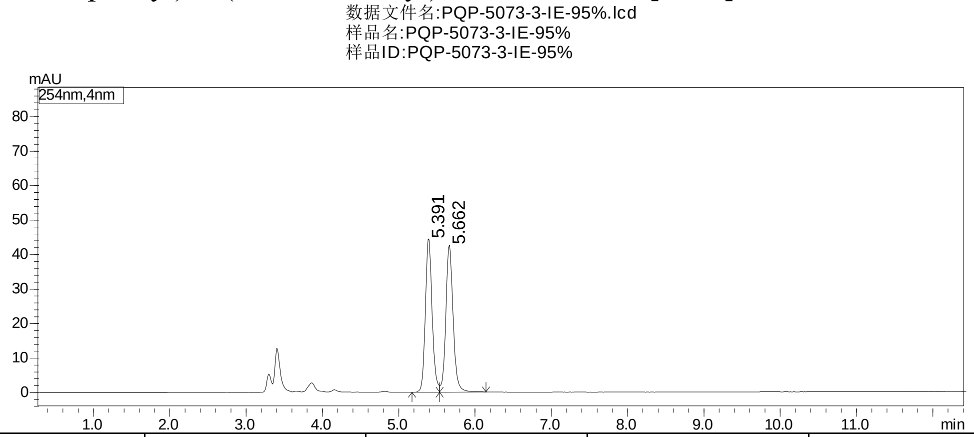

Peak	Ret. Time	Area	Height	Area%
1	5.391	273968	44442	49.331
2	5.662	281396	42701	50.669
Total		555364	87143	100.000

Supplementary Figure 96. HPLC Spectrum of racemic **3b**

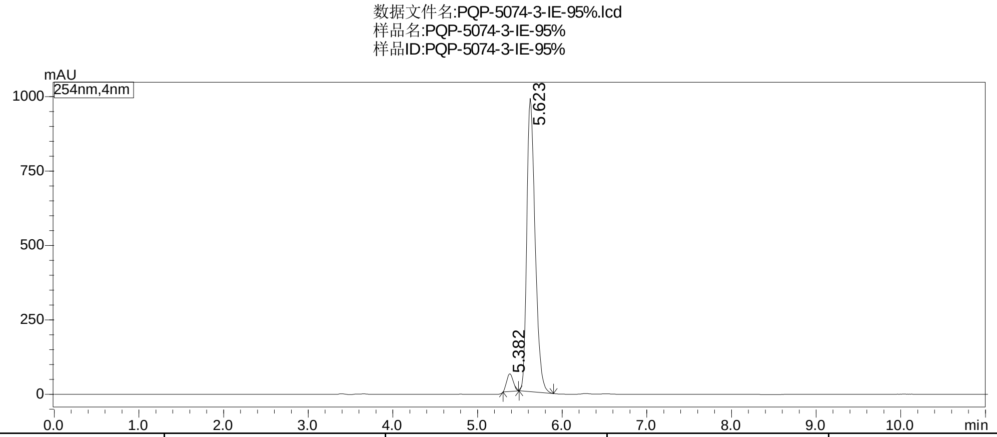

Peak	Ret. Time	Area	Height	Area%
1	5.382	316864	58704	4.476
2	5.623	6762495	985309	95.524
Total		7079359	1044013	100.000

Supplementary Figure 97. HPLC Spectrum of **3b**

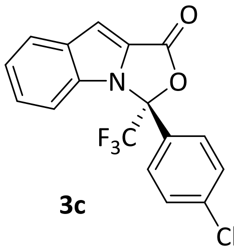

3c

3c (*R*)-3-(4-chlorophenyl)-3-(trifluoromethyl)-1*H*,3*H*-oxazolo[3,4-a]indol-1-one

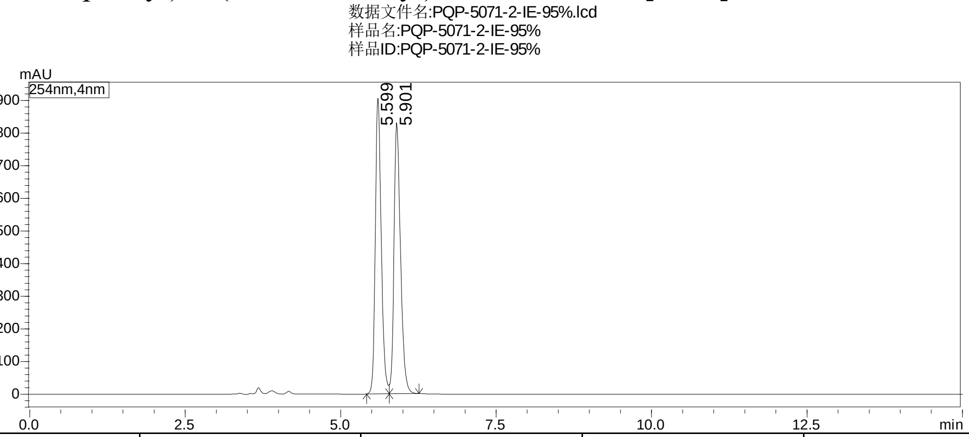

Peak	Ret. Time	Area	Height	Area%
1	5.599	6015839	906077	49.788
2	5.901	6067114	830088	50.212
Total		12082954	1736165	100.000

Supplementary Figure 98. HPLC Spectrum of racemic **3c**

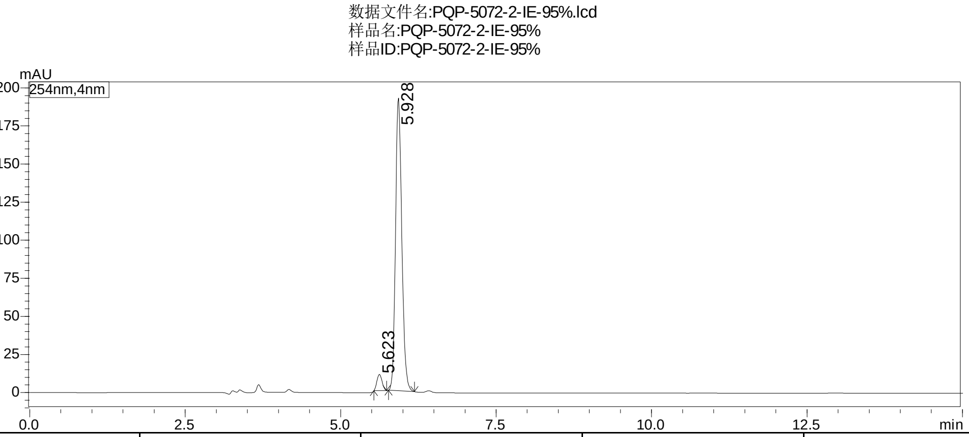

Peak	Ret. Time	Area	Height	Area%
1	5.623	60988	10681	4.310
2	5.928	1353886	192200	95.690
Total		1414873	202881	100.000

Supplementary Figure 99. HPLC Spectrum of **3c**

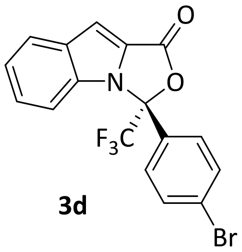

3d (*R*)-3-(4-bromophenyl)-3-(trifluoromethyl)-1*H*,3*H*-oxazolo[3,4-a]indol-1-one

数据文件名:PQP-5071-1-IE-95%.lcd
样品名:PQP-5071-1-IE-95%
样品ID:PQP-5071-1-IE-95%

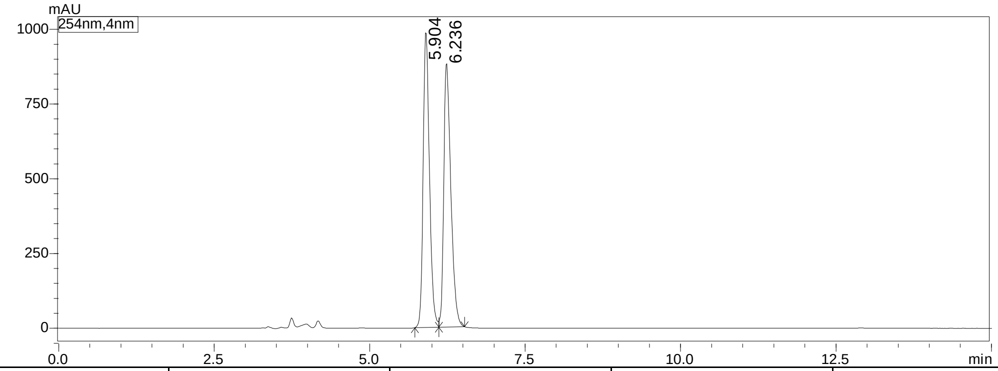

Peak	Ret. Time	Area	Height	Area%
1	5.904	6983485	985666	49.956
2	6.236	6995912	880522	50.044
Total		13979398	1866189	100.000

Supplementary Figure 100. HPLC Spectrum of racemic **3d**

数据文件名:PQP-5072-1-IE-95%.lcd
样品名:PQP-5072-1-IE-95%
样品ID:PQP-5072-1-IE-95%

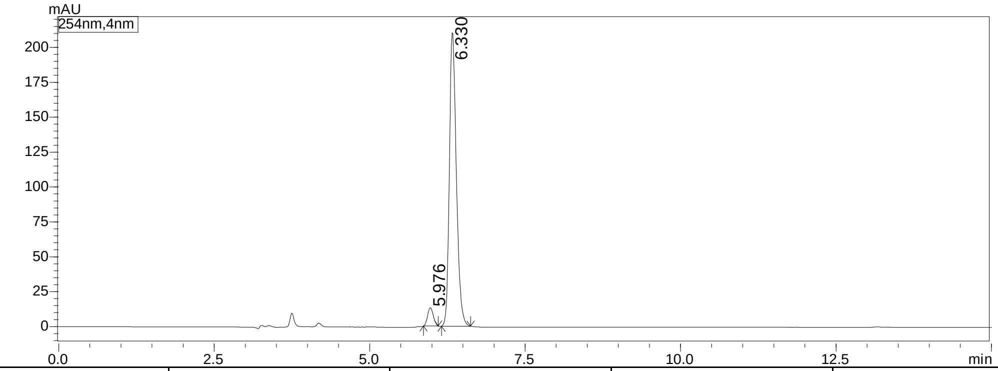

Peak	Ret. Time	Area	Height	Area%
1	5.976	83862	13104	4.867
2	6.330	1639050	210150	95.133
Total		1722912	223253	100.000

Supplementary Figure 101. HPLC Spectrum of **3d**

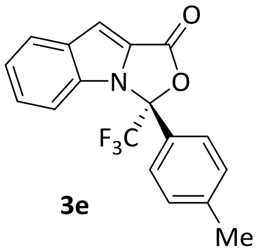

3e

3e (*R*)-3-(p-tolyl)-3-(trifluoromethyl)-1*H*,3*H*-oxazolo[3,4-a]indol-1-one

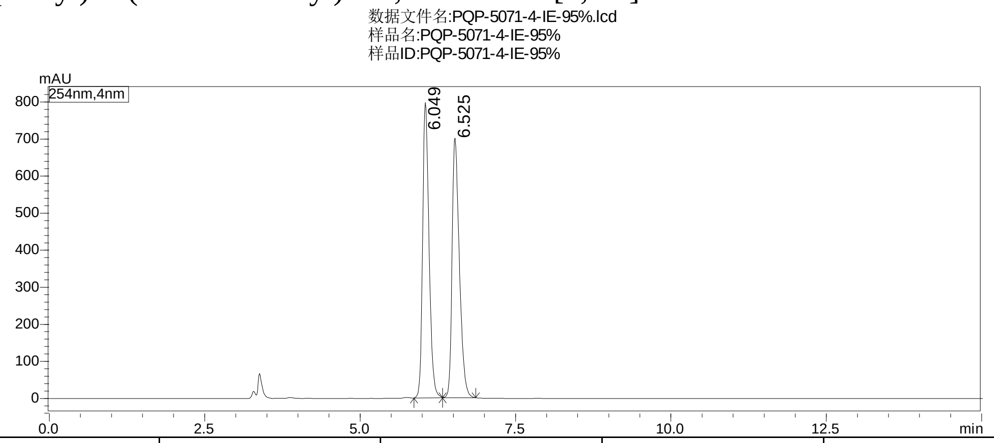

Peak	Ret. Time	Area	Height	Area%
1	6.049	5726063	796923	49.947
2	6.525	5738154	700552	50.053
Total		11464217	1497476	100.000

Supplementary Figure 102. HPLC Spectrum of racemic **3e**

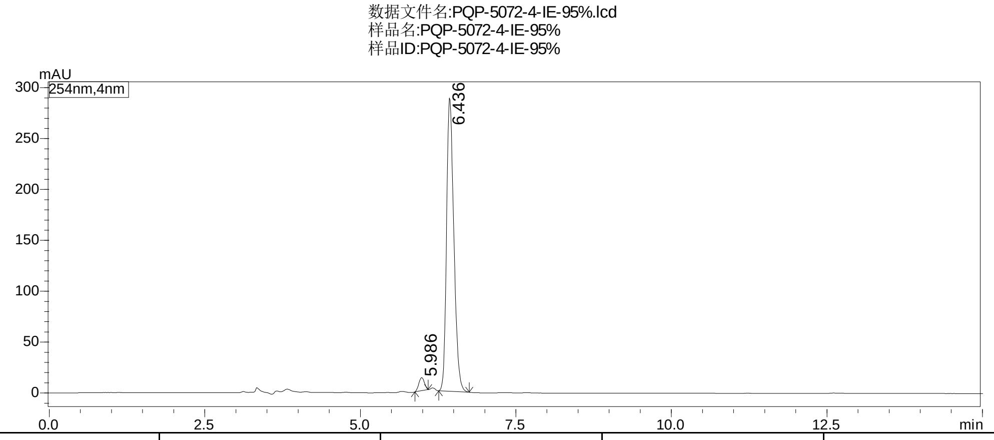

Peak	Ret. Time	Area	Height	Area%
1	5.986	80613	12871	3.314
2	6.436	2352093	288340	96.686
Total		2432706	301211	100.000

Supplementary Figure 103. HPLC Spectrum of **3e**

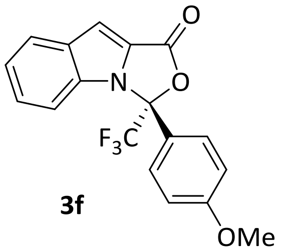

3f

3f (*R*)-3-(4-methoxyphenyl)-3-(trifluoromethyl)-1*H*,3*H*-oxazolo[3,4-a]indol-1-one

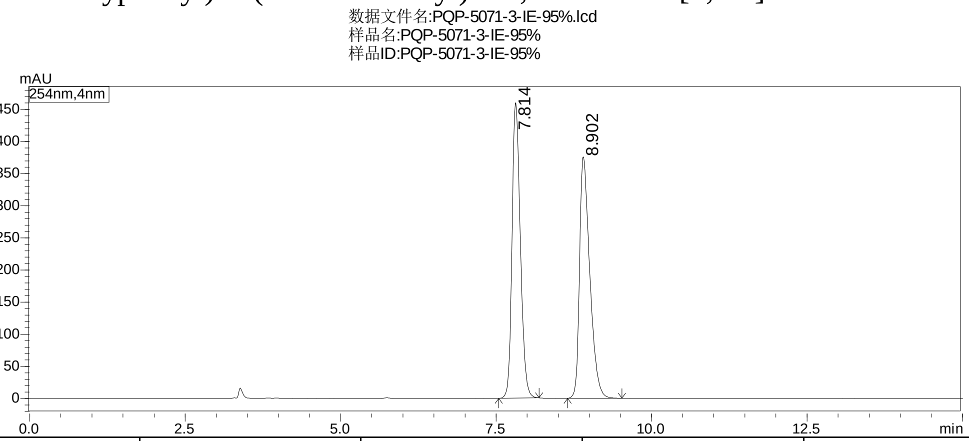

Peak	Ret. Time	Area	Height	Area%
1	7.814	4282337	460142	49.822
2	8.902	4312865	375758	50.178
Total		8595201	835900	100.000

Supplementary Figure 104. HPLC Spectrum of racemic **3f**

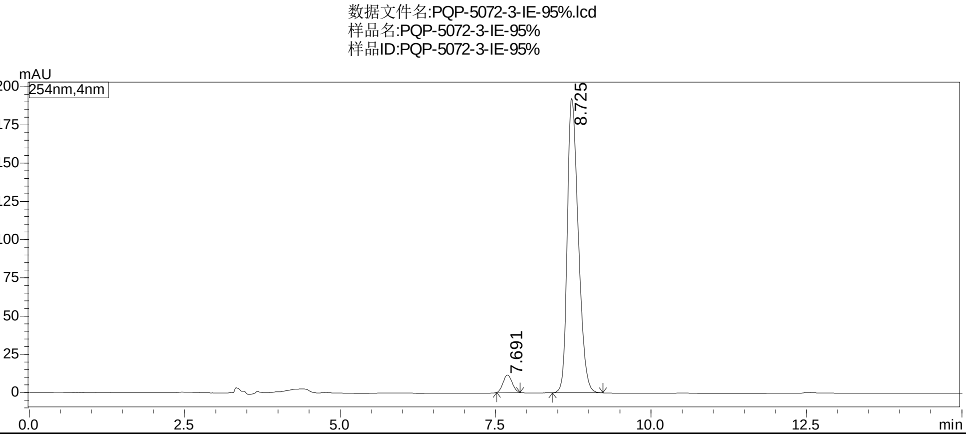

Peak	Ret. Time	Area	Height	Area%
1	7.691	114266	11368	4.586
2	8.725	2377435	192573	95.414
Total		2491701	203942	100.000

Supplementary Figure 105. HPLC Spectrum of **3f**

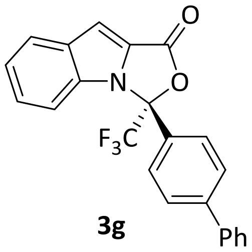

3g

3g (*R*)-3-([1,1'-biphenyl]-4-yl)-3-(trifluoromethyl)-1*H*,3*H*-oxazolo[3,4-a]indol-1-one

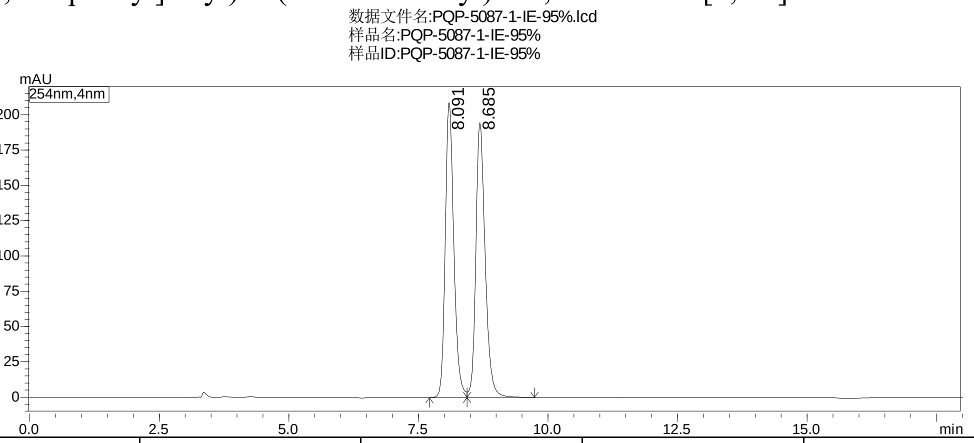

Peak	Ret. Time	Area	Height	Area%
1	8.091	2405959	208797	49.658
2	8.685	2439080	194643	50.342
Total		4845039	403439	100.000

Supplementary Figure 106. HPLC Spectrum of racemic **3g**

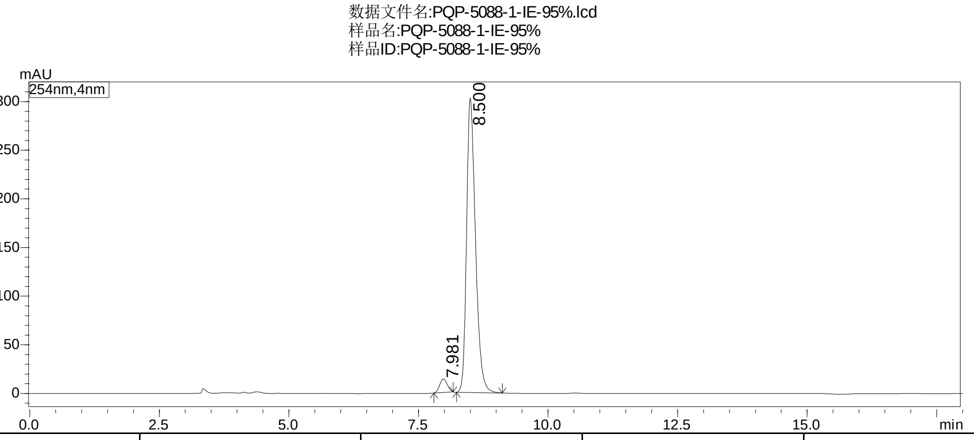

Peak	Ret. Time	Area	Height	Area%
1	7.981	141872	13896	3.778
2	8.500	3613217	302585	96.222
Total		3755090	316481	100.000

Supplementary Figure 107. HPLC Spectrum of **3g**

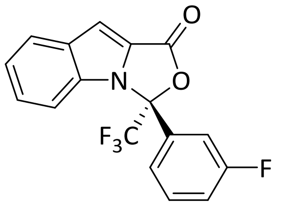

3h

3h (*R*)-3-(3-fluorophenyl)-3-(trifluoromethyl)-1*H*,3*H*-oxazolo[3,4-a]indol-1-one

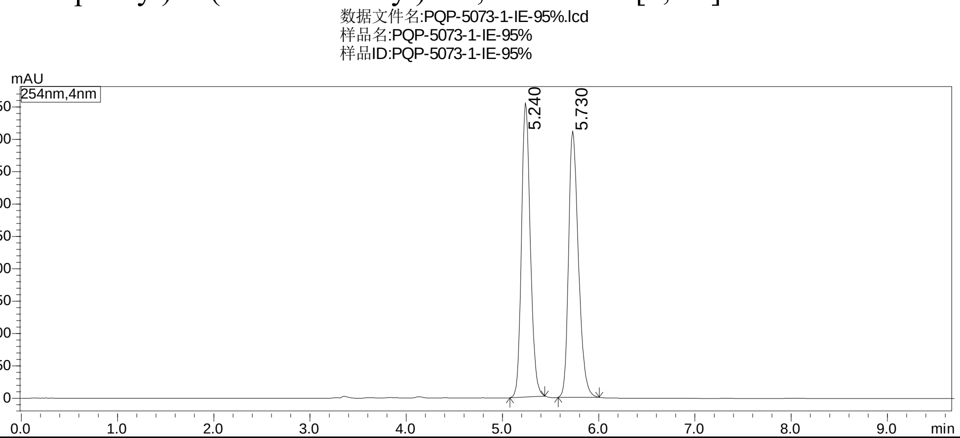

Peak	Ret. Time	Area	Height	Area%
1	5.240	2899376	454977	49.713
2	5.730	2932826	411710	50.287
Total		5832202	866687	100.000

Supplementary Figure 108. HPLC Spectrum of racemic **3h**

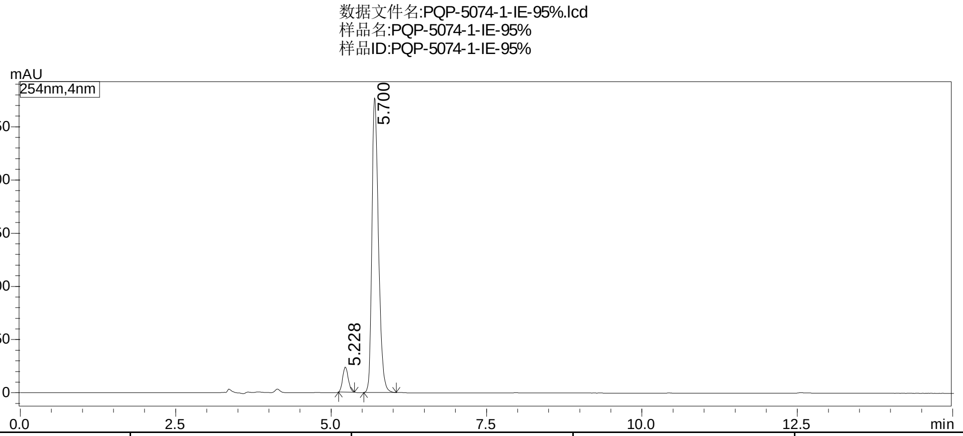

Peak	Ret. Time	Area	Height	Area%
1	5.228	146891	23592	6.719
2	5.700	2039277	277021	93.281
Total		2186169	300613	100.000

Supplementary Figure 109. HPLC Spectrum of **3h**

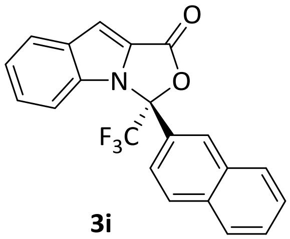

3i

3i (*R*)-3-(naphthalen-2-yl)-3-(trifluoromethyl)-1*H*,3*H*-oxazolo[3,4-a]indol-1-one

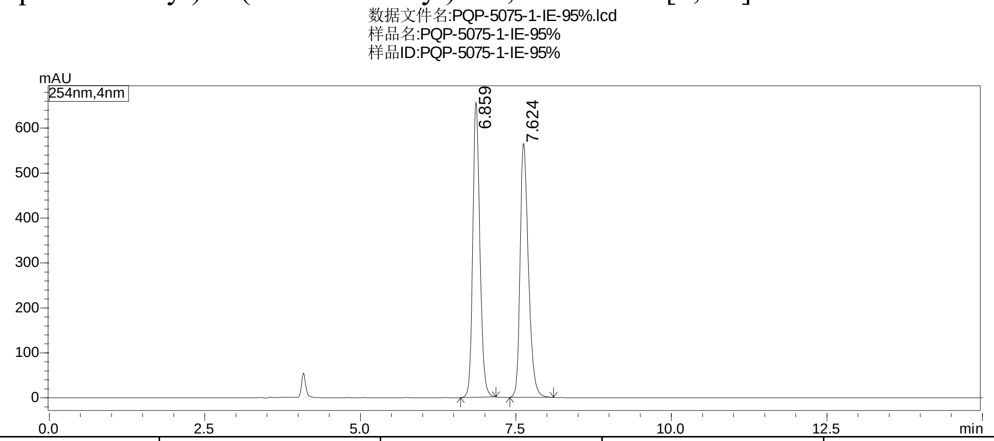

Peak	Ret. Time	Area	Height	Area%
1	6.859	5231341	657273	49.886
2	7.624	5255333	565049	50.114
Total		10486675	1222322	100.000

Supplementary Figure 110. HPLC Spectrum of racemic **3i**

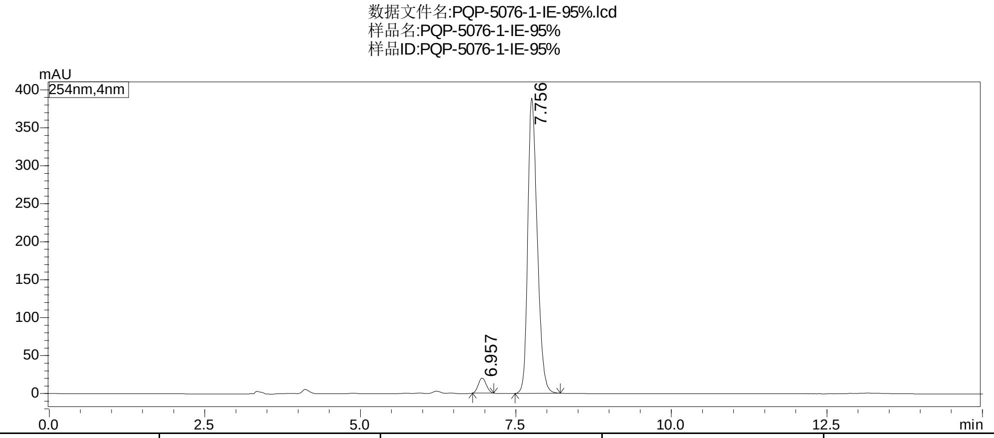

Peak	Ret. Time	Area	Height	Area%
1	6.957	175362	19819	4.075
2	7.756	4127874	388884	95.925
Total		4303237	408704	100.000

Supplementary Figure 111. HPLC Spectrum of **3i**

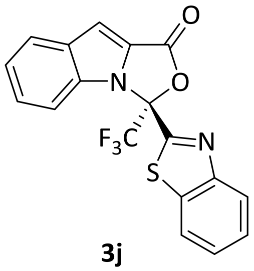

3j

3j (*S*)-3-(benzo[d]thiazol-2-yl)-3-(trifluoromethyl)-1*H*,3*H*-oxazolo[3,4-a]indol-1-one

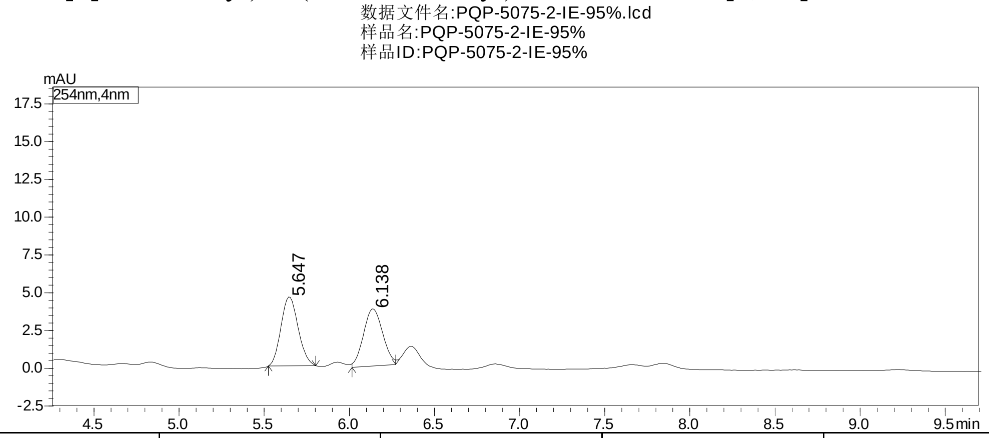

Peak	Ret. Time	Area	Height	Area%
1	5.647	32048	4558	51.787
2	6.138	29836	3786	48.213
Total		61884	8344	100.000

Supplementary Figure 112. HPLC Spectrum of racemic **3j**

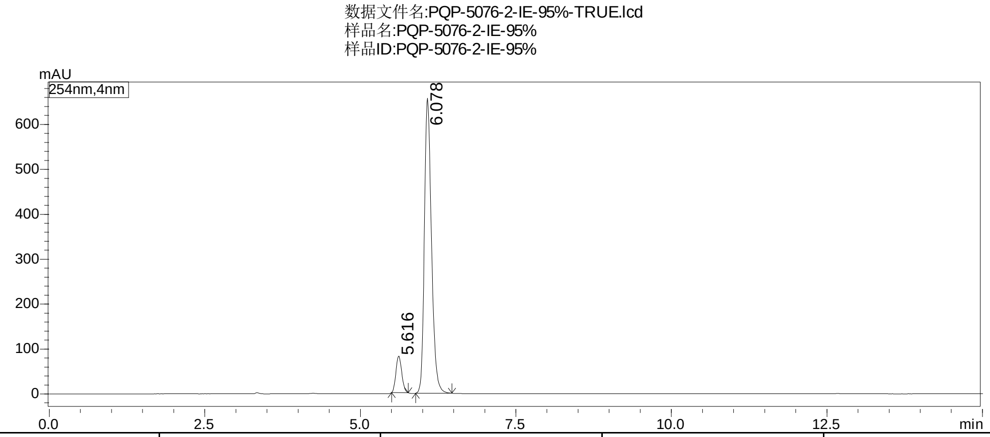

Peak	Ret. Time	Area	Height	Area%
1	5.616	525811	81122	9.188
2	6.078	5196880	657343	90.812
Total		5722691	738465	100.000

Supplementary Figure 113. HPLC Spectrum of **3j**

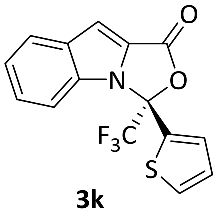

3k

3k (*S*)-3-(thiophen-2-yl)-3-(trifluoromethyl)-1*H*,3*H*-oxazolo[3,4-a]indol-1-one

数据文件名:PQP-5075-4-IE-95%.lcd
样品名:PQP-5075-4-IE-95%
样品ID:PQP-5075-4-IE-95%

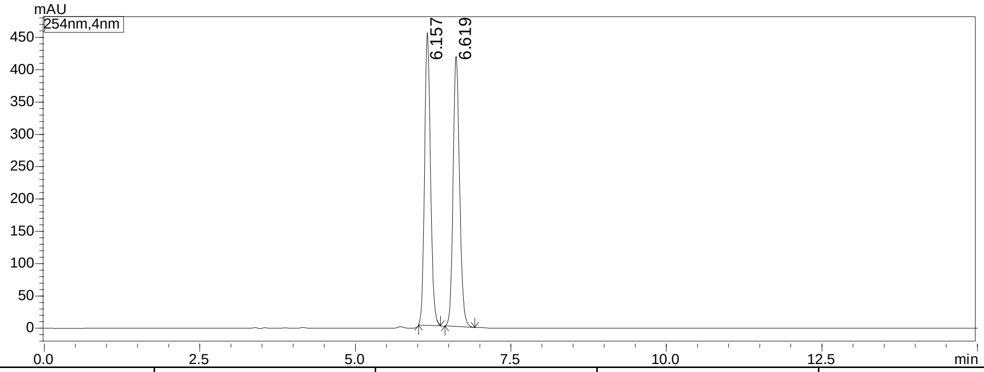

Peak	Ret. Time	Area	Height	Area%
1	6.157	3022535	452680	49.699
2	6.619	3059150	417627	50.301
Total		6081685	870307	100.000

Supplementary Figure 114. HPLC Spectrum of racemic **3k**

数据文件名:PQP-5076-4-IE-95%.lcd
样品名:PQP-5076-4-IE-95%
样品ID:PQP-5076-4-IE-95%

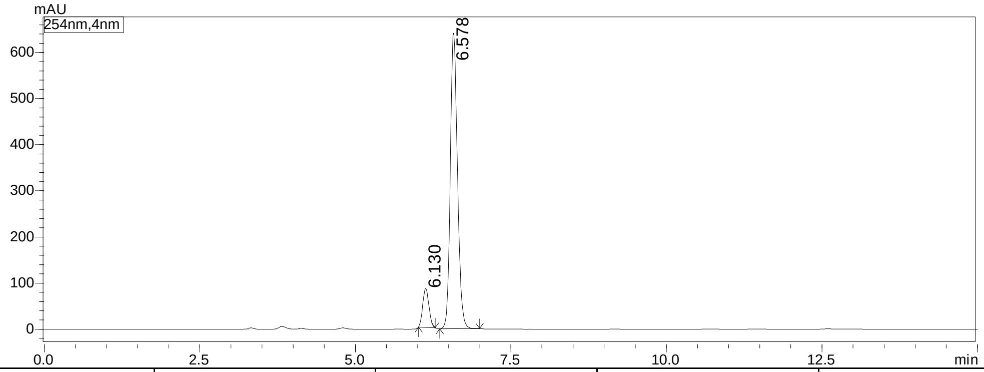

Peak	Ret. Time	Area	Height	Area%
1	6.130	583347	84238	10.204
2	6.578	5133719	640994	89.796
Total		5717066	725232	100.000

Supplementary Figure 115. HPLC Spectrum of **3k**

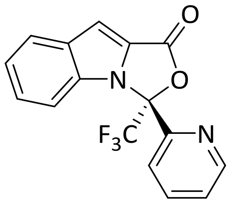

3l

3l (*R*)-3-(pyridin-2-yl)-3-(trifluoromethyl)-1*H*,3*H*-oxazolo[3,4-a]indol-1-one

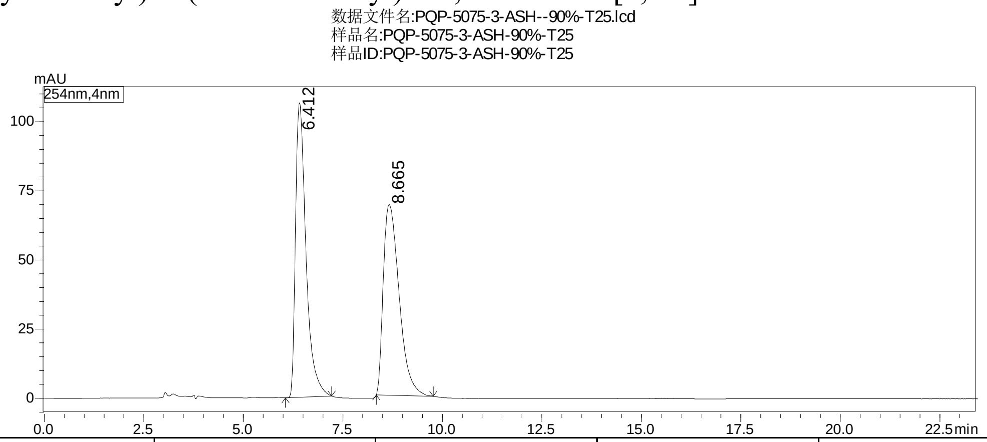

Peak	Ret. Time	Area	Height	Area%
1	6.412	1953667	106504	50.589
2	8.665	1908197	68935	49.411
Total		3861864	175439	100.000

Supplementary Figure 116. HPLC Spectrum of racemic **3l**

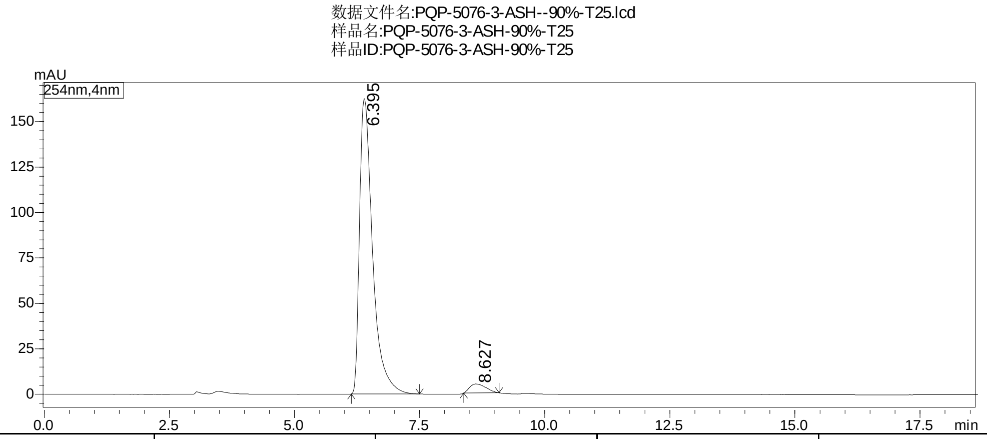

Peak	Ret. Time	Area	Height	Area%
1	6.395	2958604	162459	96.370
2	8.627	111448	4936	3.630
Total		3070052	167394	100.000

Supplementary Figure 117. HPLC Spectrum of **3l**

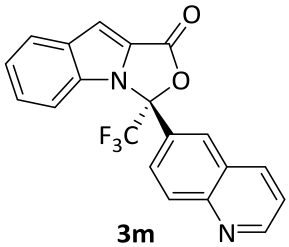

3m

3m (*R*)-3-(quinolin-6-yl)-3-(trifluoromethyl)-1*H*,3*H*-oxazolo[3,4-a]indol-1-one

数据文件名:PQP-5086-3m-rac-IE-90%.lcd
样品名:PQP-5086-3m-rac-IE-90%
样品ID:PQP-5086-3m-rac-IE-90%

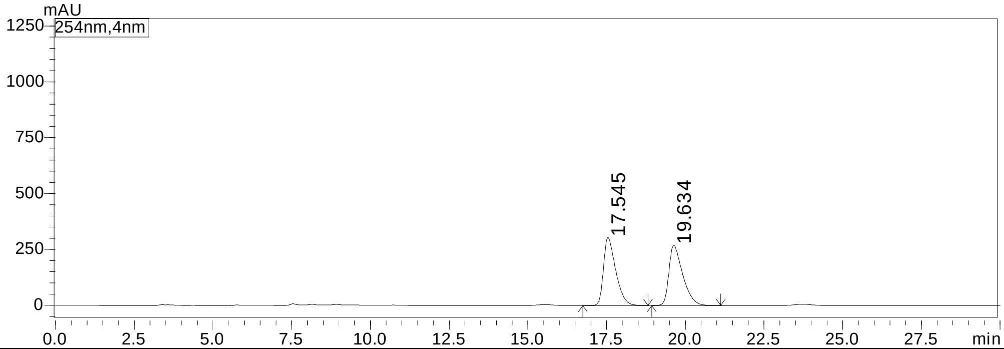

Peak	Ret. Time	Area	Height	Area%
1	17.545	8237867	304910	49.928
2	19.634	8261509	269976	50.072
Total		16499376	574886	100.000

Supplementary Figure 118. HPLC Spectrum of racemic **3m**

数据文件名:PQP-5088-5-IE-90.lcd
样品名:PQP-5088-5-IE-90
样品ID:PQP-5088-5-IE-90

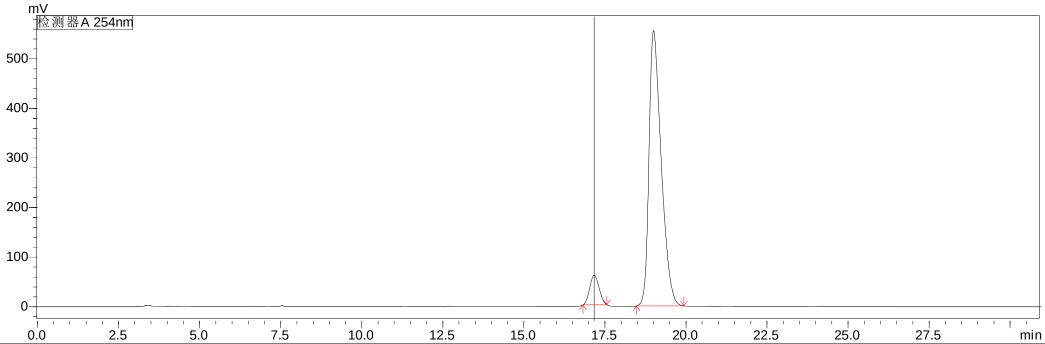

Peak	Ret. Time	Area	Height	Area%
1	17.169	1198377	59667	7.625
2	19.000	14517635	555775	92.375
Total		15716012	615442	100.000

Supplementary Figure 119. HPLC Spectrum of **3m**

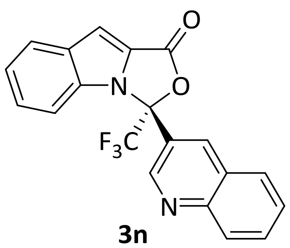

3n

3n (*R*)-3-(quinolin-3-yl)-3-(trifluoromethyl)-1*H*,3*H*-oxazolo[3,4-a]indol-1-one

数据文件名:PQP-5089-2-ASH-90.lcd
样品名:PQP-5089-2-ASH-90
样品ID:PQP-5089-2-ASH-90

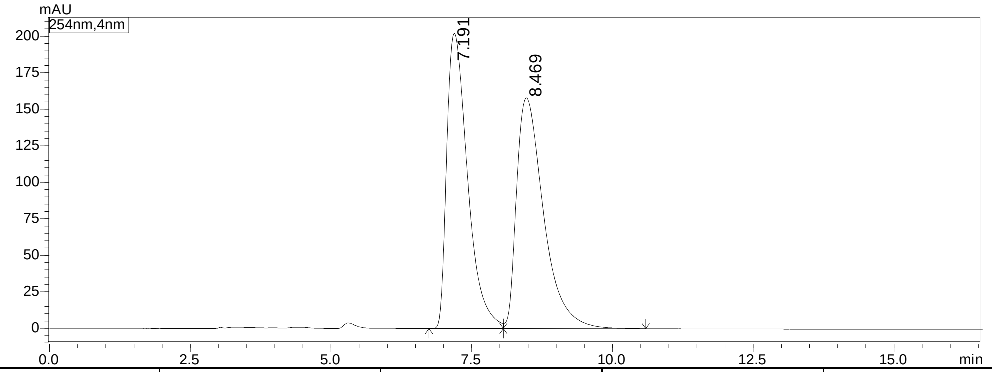

Peak	Ret. Time	Area	Height	Area%
1	7.191	5213857	202132	49.669
2	8.469	5283344	157968	50.331
Total		10497201	360101	100.000

Supplementary Figure 120. HPLC Spectrum of racemic **3n**

数据文件名:PQP-5086-2-ASH-98%.lcd
样品名:PQP-5086-2-ASH-98%
样品ID:PQP-5086-2-ASH-98%

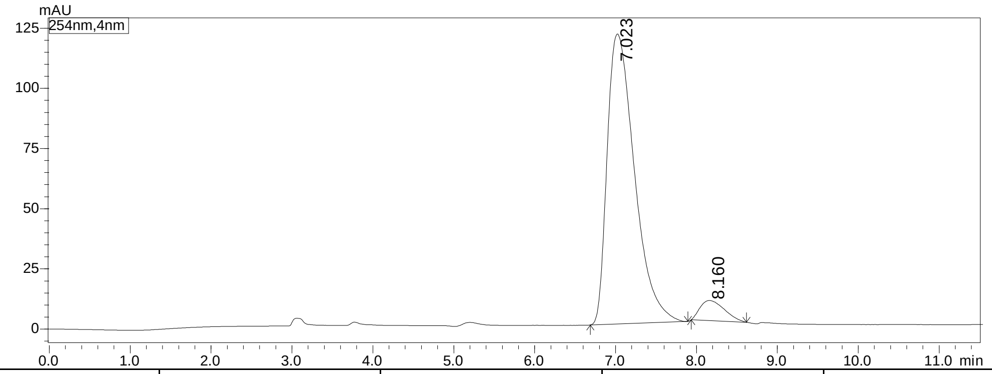

Peak	Ret. Time	Area	Height	Area%
1	7.023	2831583	120486	94.197
2	8.160	174444	8349	5.803
Total		3006027	128835	100.000

Supplementary Figure 121. HPLC Spectrum of **3n**

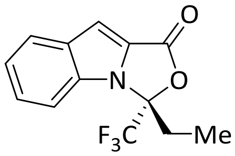

3o

3o (*R*)-3-ethyl-3-(trifluoromethyl)-1*H*,3*H*-oxazolo[3,4-a]indol-1-one

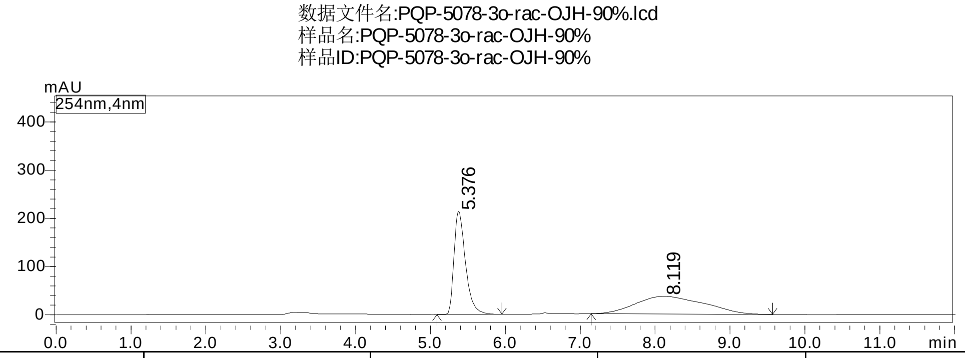

Peak	Ret. Time	Area	Height	Area%
1	5.376	2284067	214189	49.432
2	8.119	2336534	36789	50.568
Total		4620601	250978	100.000

Supplementary Figure 122. HPLC Spectrum of racemic **3o**

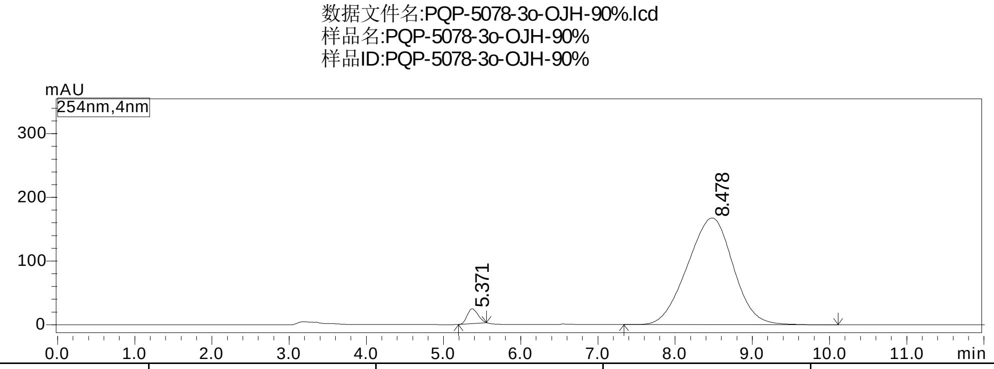

Peak	Ret. Time	Area	Height	Area%
1	5.371	224980	23130	3.149
2	8.478	6918939	167722	96.851
Total		7143919	190853	100.000

Supplementary Figure 123. HPLC Spectrum of **3o**

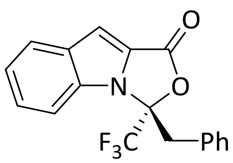

3p

3p (*R*)-3-benzyl-3-(trifluoromethyl)-1*H*,3*H*-oxazolo[3,4-a]indol-1-one

数据文件名:PQP-5077-3p-rac-ADH-98%.lcd
样品名:PQP-5077-3p-rac-ADH-98%
样品ID:PQP-5077-3p-rac-ADH-98%

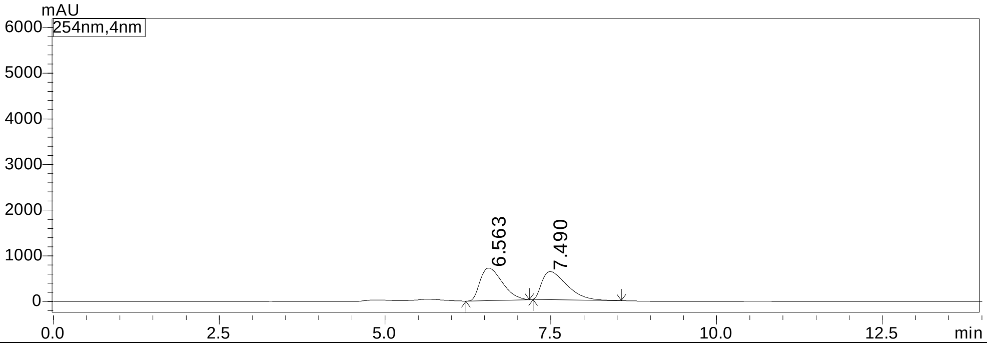

Peak	Ret. Time	Area	Height	Area%
1	6.563	16704603	713387	50.953
2	7.490	16079859	614222	49.047
Total		32784462	1327610	100.000

Supplementary Figure 124. HPLC Spectrum of racemic **3p**

数据文件名:PQP-5077-3p-ADH-98%.lcd
样品名:PQP-5077-3p-ADH-98%
样品ID:PQP-5077-3p-ADH-98%

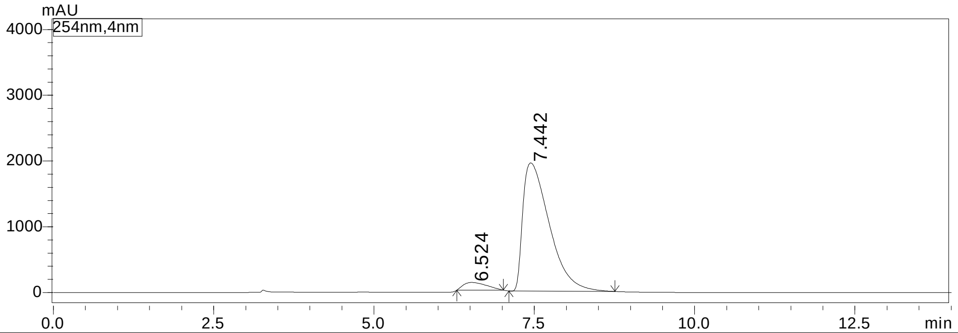

Peak	Ret. Time	Area	Height	Area%
1	6.524	2955364	116253	4.996
2	7.442	56199759	1950276	95.004
Total		59155124	2066529	100.000

Supplementary Figure 125. HPLC Spectrum of **3p**

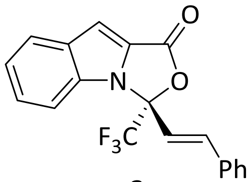

3q

3q (*R,E*)-3-styryl-3-(trifluoromethyl)-1*H*,3*H*-oxazolo[3,4-a]indol-1-one

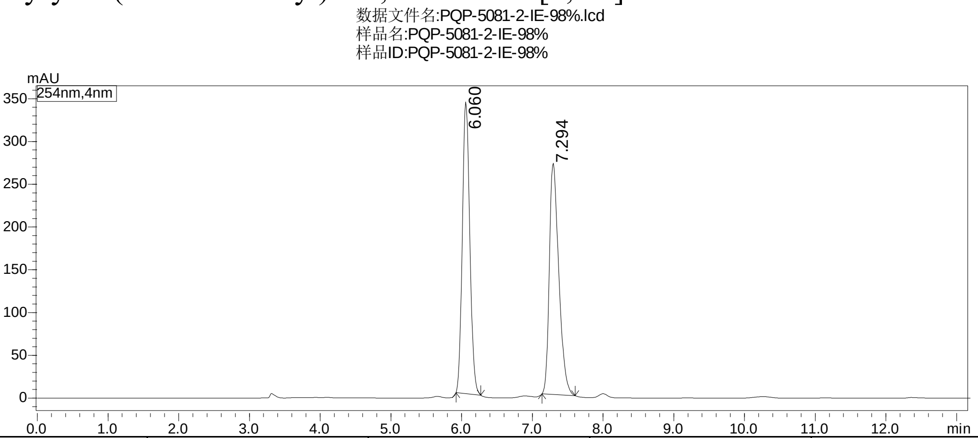

Peak	Ret. Time	Area	Height	Area%
1	6.060	2513781	340991	50.022
2	7.294	2511598	270309	49.978
Total		5025379	611300	100.000

Supplementary Figure 126. HPLC Spectrum of racemic **3q**

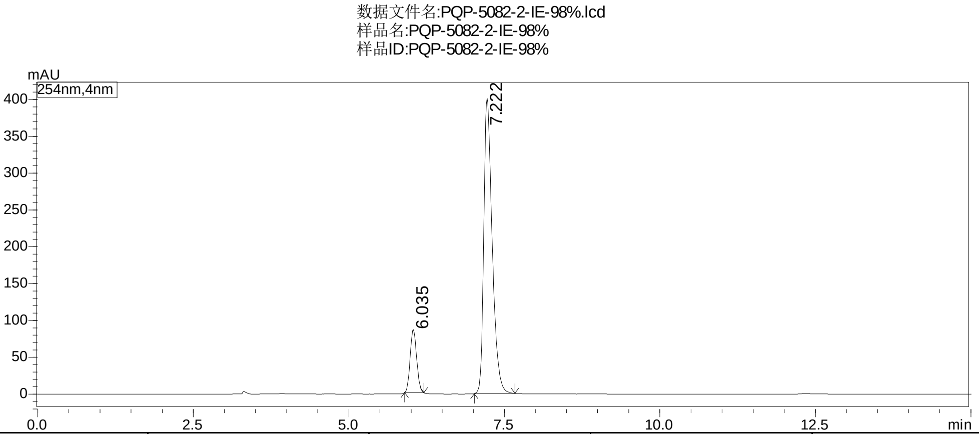

Peak	Ret. Time	Area	Height	Area%
1	6.035	620544	85512	14.127
2	7.222	3771975	400869	85.873
Total		4392519	486381	100.000

Supplementary Figure 127. HPLC Spectrum of **3q**

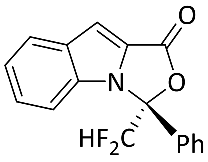

3r

3r (*R*)-3-(difluoromethyl)-3-phenyl-1*H*,3*H*-oxazolo[3,4-a]indol-1-one

数据文件名:PQP-5086-3r-rac-IE-98%-2.lcd
样品名:PQP-5086-3r-rac-IE-98%-2
样品ID:PQP-5086-3r-rac-IE-98%-2

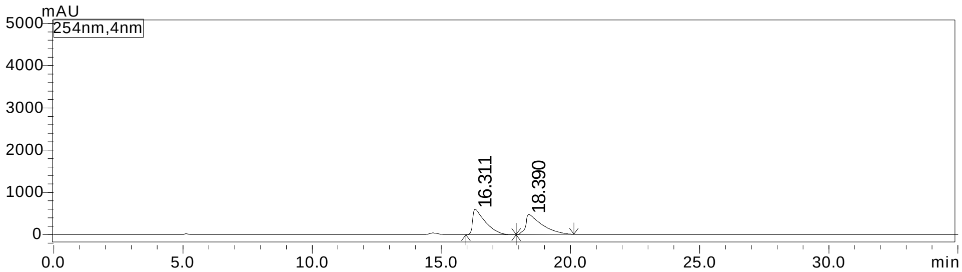

Peak	Ret. Time	Area	Height	Area%
1	16.311	20373602	600415	49.330
2	18.390	20927254	474393	50.670
Total		41300856	1074808	100.000

Supplementary Figure 128. HPLC Spectrum of racemic **3r**

数据文件名:PQP-5086-3r-IE-98%-2.lcd
样品名:PQP-5086-3r-IE-98%-2
样品ID:PQP-5086-3r-IE-98%-2

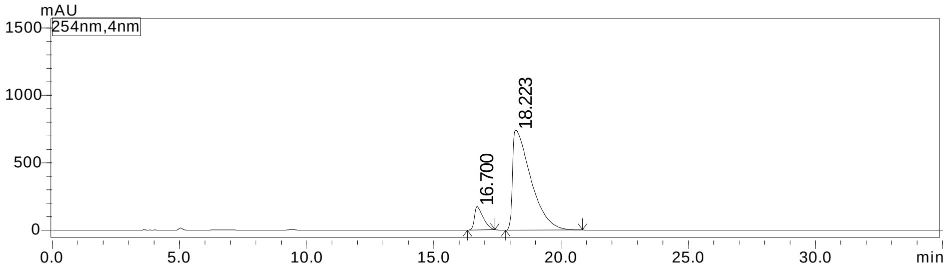

Peak	Ret. Time	Area	Height	Area%
1	16.700	4102384	173350	9.984
2	18.223	36987098	740982	90.016
Total		41089482	914332	100.000

Supplementary Figure 129. HPLC Spectrum of racemic **3r**

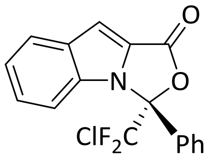

3s

3s (*R*)-3-(chlorodifluoromethyl)-3-phenyl-1*H*,3*H*-oxazolo[3,4-a]indol-1-one

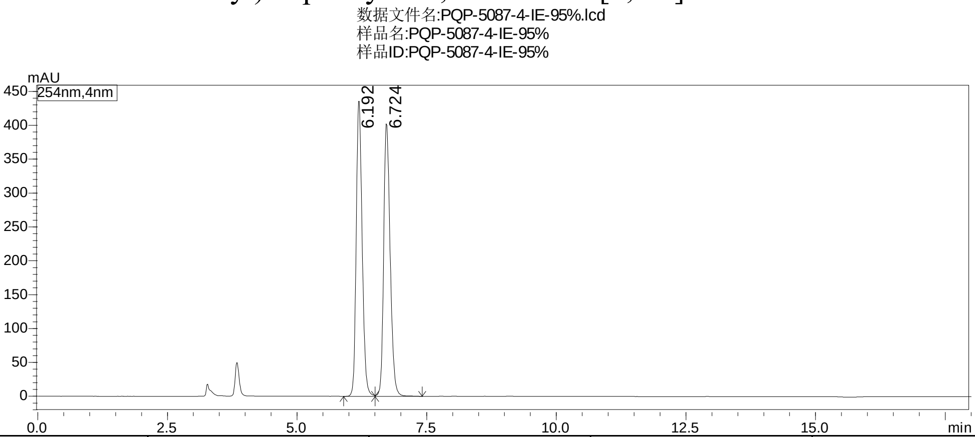

Peak	Ret. Time	Area	Height	Area%
1	6.192	3482123	435569	49.775
2	6.724	3513642	402327	50.225
Total		6995765	837896	100.000

Supplementary Figure 130. HPLC Spectrum of racemic **3s**

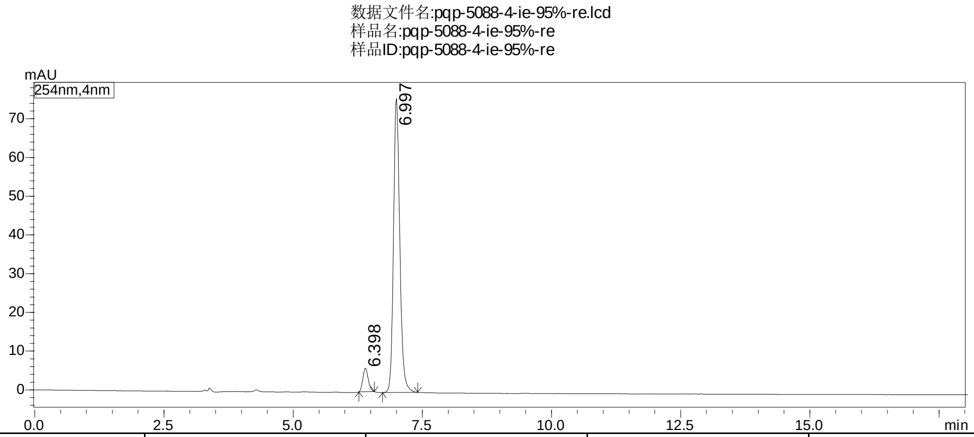

Peak	Ret. Time	Area	Height	Area%
1	6.398	43790	6091	6.384
2	6.997	642189	75939	93.616
Total		685979	82030	100.000

Supplementary Figure 131. HPLC Spectrum of **3s**

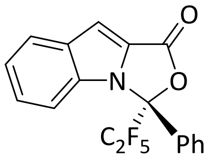

3t

3t (*R*)-3-(perfluoroethyl)-3-phenyl-1*H*,3*H*-oxazolo[3,4-a]indol-1-one

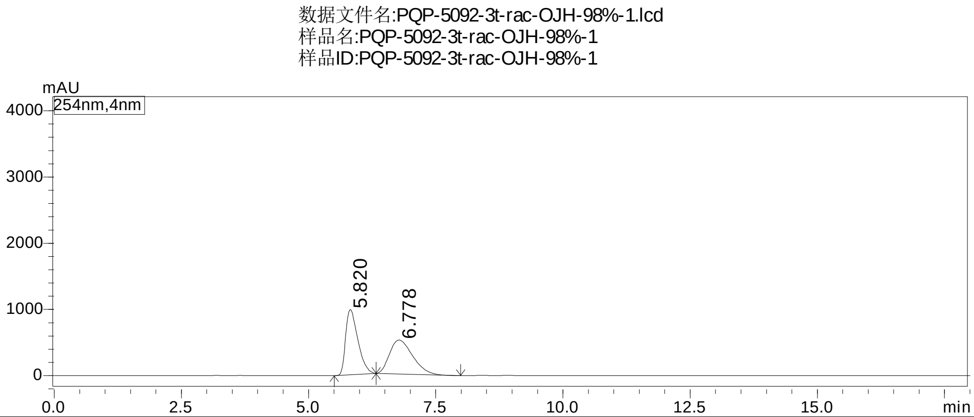

Peak	Ret. Time	Area	Height	Area%
1	5.820	16478135	983813	50.498
2	6.778	16153346	511829	49.502
Total		32631481	1495642	100.000

Supplementary Figure 132. HPLC Spectrum of racemic **3t**

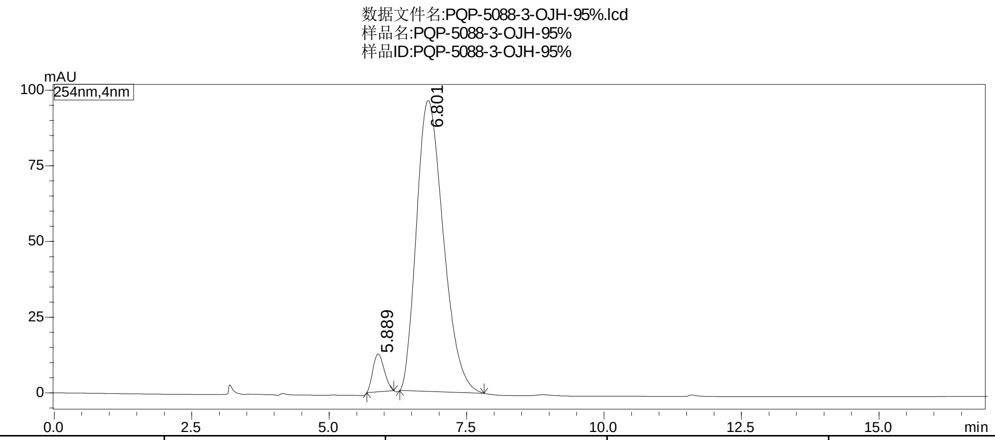

Peak	Ret. Time	Area	Height	Area%
1	5.889	179614	12516	5.206
2	6.801	3270789	96120	94.794
Total		3450403	108636	100.000

Supplementary Figure 133. HPLC Spectrum of **3t**

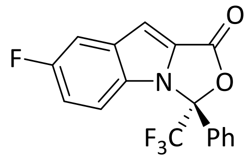

4a

4a (*R*)-7-fluoro-3-phenyl-3-(trifluoromethyl)-1*H*,3*H*-oxazolo[3,4-a]indol-1-one

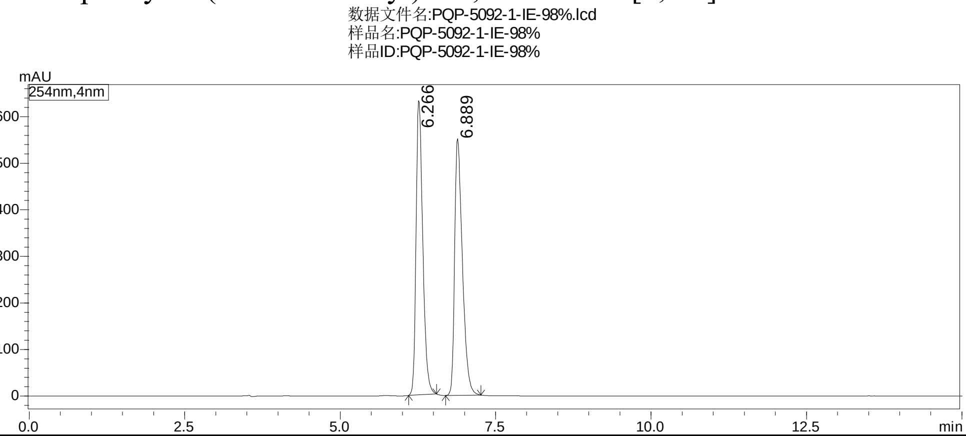

Peak	Ret. Time	Area	Height	Area%
1	6.266	4725127	631845	49.648
2	6.889	4792104	551495	50.352
Total		9517232	1183340	100.000

Supplementary Figure 134. HPLC Spectrum of racemic **4a**

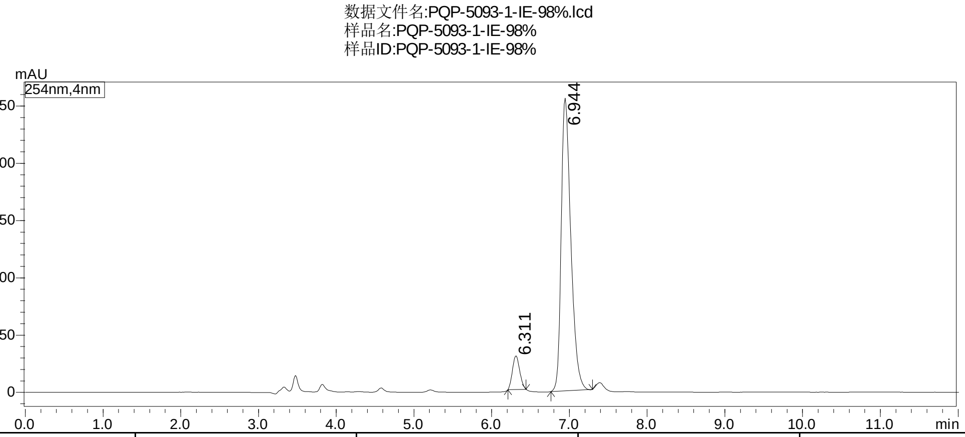

Peak	Ret. Time	Area	Height	Area%
1	6.311	190274	29314	7.993
2	6.944	2190291	255526	92.007
Total		2380565	284840	100.000

Supplementary Figure 135. HPLC Spectrum of **4a**

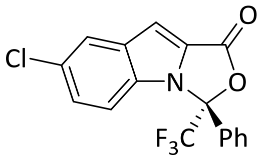

4b

4b (*R*)-7-chloro-3-phenyl-3-(trifluoromethyl)-1*H*,3*H*-oxazolo[3,4-a]indol-1-one

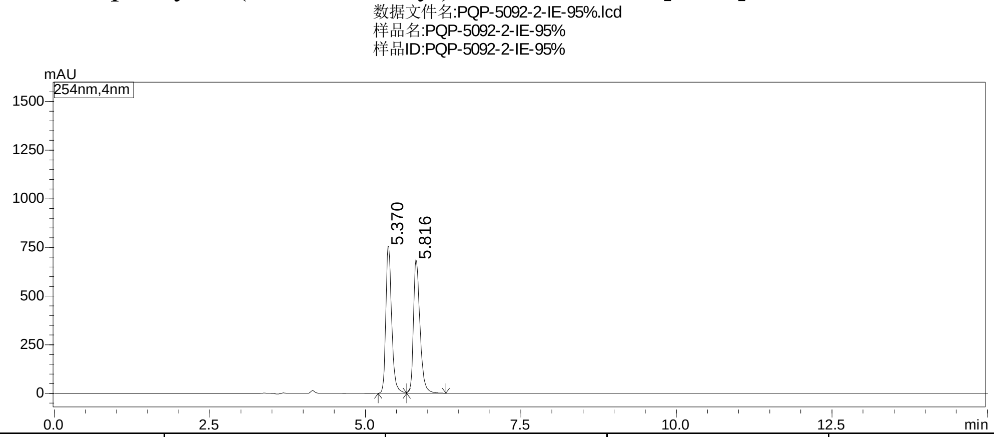

Peak	Ret. Time	Area	Height	Area%
1	5.370	4930687	757504	49.684
2	5.816	4993309	686290	50.316
Total		9923996	1443794	100.000

Supplementary Figure 136. HPLC Spectrum of racemic **4b**

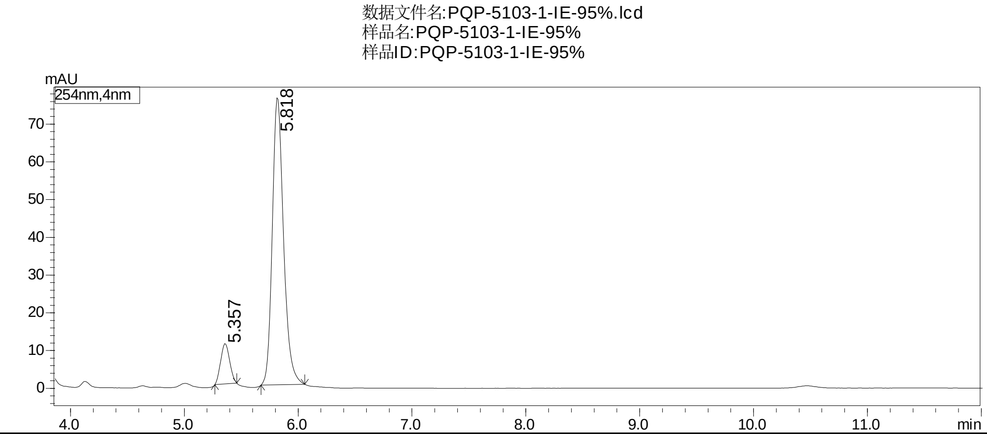

Peak	Ret. Time	Area	Height	Area%
1	5.357	58405	10684	9.993
2	5.818	526066	76022	90.007
Total		584471	86706	100.000

Supplementary Figure 137. HPLC Spectrum of **4b**

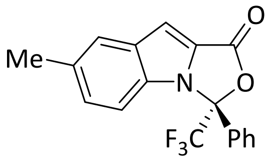

4c

4c (*R*)-7-methyl-3-phenyl-3-(trifluoromethyl)-1*H*,3*H*-oxazolo[3,4-a]indol-1-one

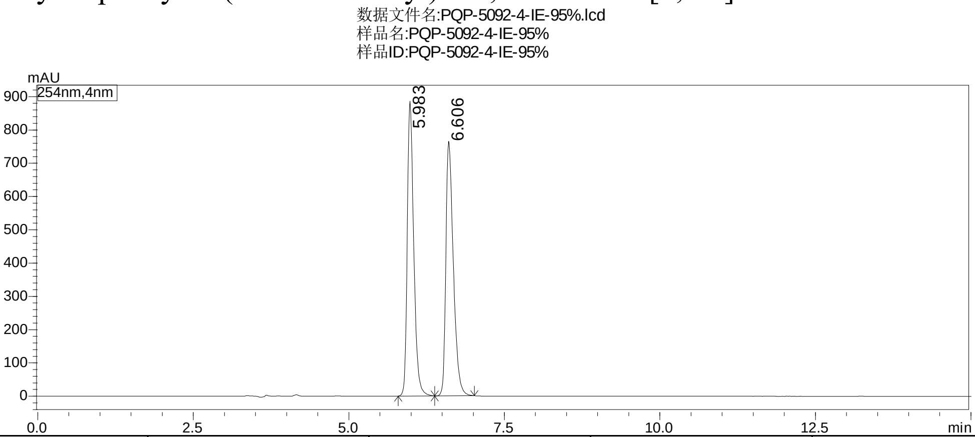

Peak	Ret. Time	Area	Height	Area%
1	5.983	6421004	885762	49.965
2	6.606	6429931	764576	50.035
Total		12850935	1650338	100.000

Supplementary Figure 138. HPLC Spectrum of racemic **4c**

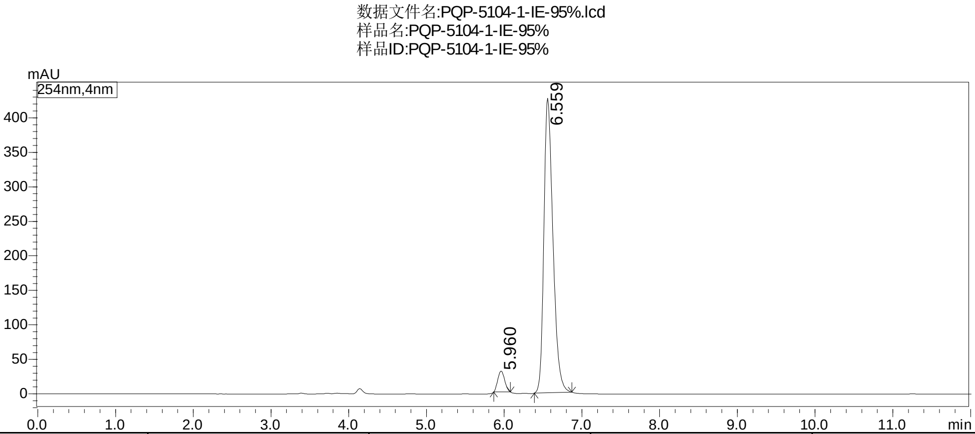

Peak	Ret. Time	Area	Height	Area%
1	5.960	184812	30159	5.066
2	6.559	3463481	426818	94.934
Total		3648293	456977	100.000

Supplementary Figure 139. HPLC Spectrum of **4c**

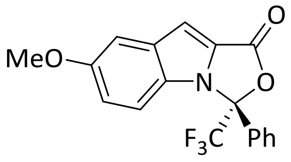

4d

4d (*R*)-7-methoxy-3-phenyl-3-(trifluoromethyl)-1*H*,3*H*-oxazolo[3,4-a]indol-1-one

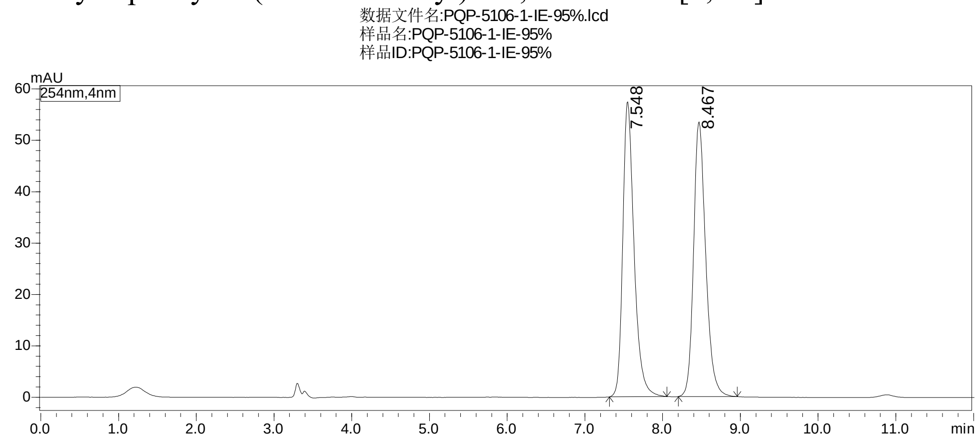

Peak	Ret. Time	Area	Height	Area%
1	7.548	579014	57401	49.945
2	8.467	580286	53481	50.055
Total		1159300	110882	100.000

Supplementary Figure 140. HPLC Spectrum of racemic **4d**

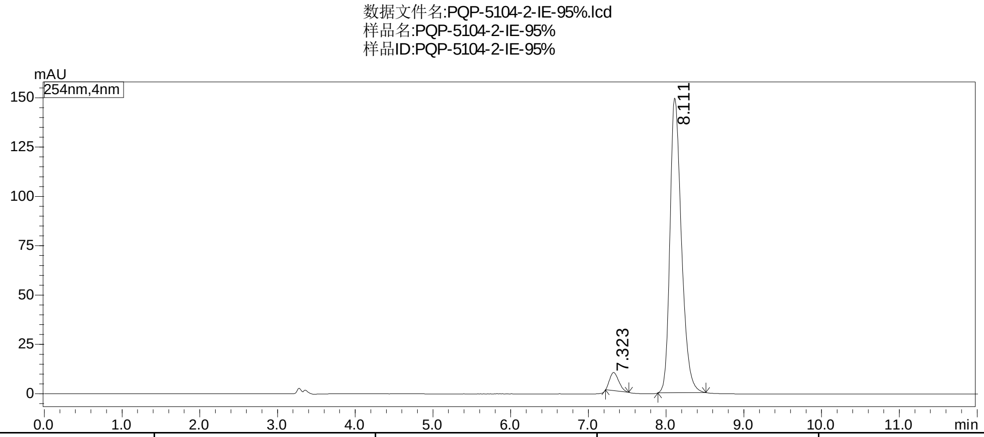

Peak	Ret. Time	Area	Height	Area%
1	7.323	74565	9212	4.633
2	8.111	1534727	149364	95.367
Total		1609292	158576	100.000

Supplementary Figure 141. HPLC Spectrum of **4d**

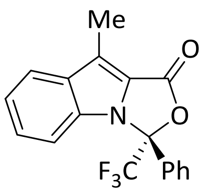

4e

4e (*R*)-9-methyl-3-phenyl-3-(trifluoromethyl)-1*H*,3*H*-oxazolo[3,4-a]indol-1-one

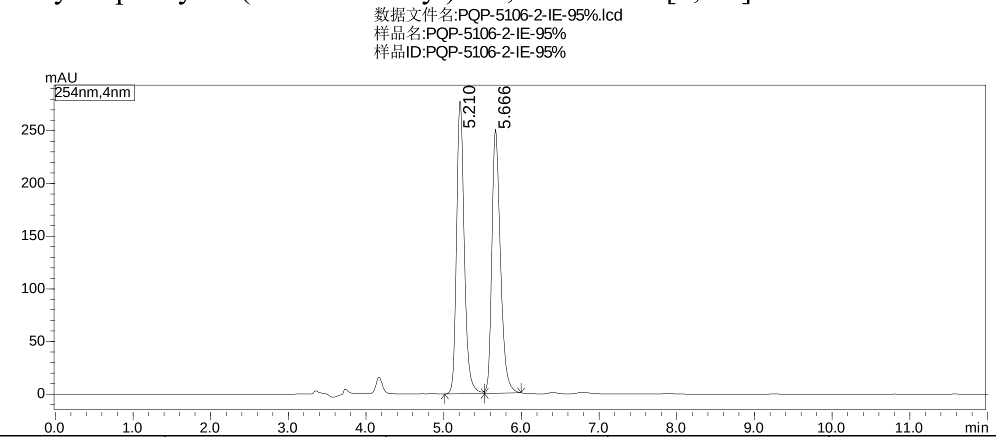

Peak	Ret. Time	Area	Height	Area%
1	5.210	1920332	277746	49.959
2	5.666	1923448	250549	50.041
Total		3843779	528295	100.000

Supplementary Figure 142. HPLC Spectrum of racemic **4e**

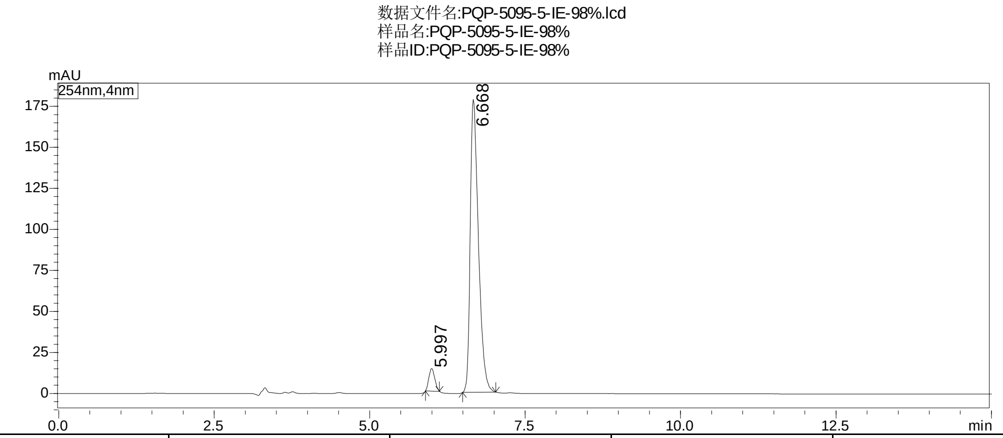

Peak	Ret. Time	Area	Height	Area%
1	5.997	89862	13753	5.206
2	6.668	1636237	178659	94.794
Total		1726100	192412	100.000

Supplementary Figure 143. HPLC Spectrum of **4e**

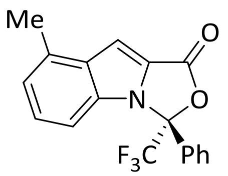

4f

4f (*R*)-8-methyl-3-phenyl-3-(trifluoromethyl)-1*H*,3*H*-oxazolo[3,4-a]indol-1-one

数据文件名:PQP-5106-4f-rac-IE-95%.lcd
样品名:PQP-5106-4f-rac-IE-95%
样品ID:PQP-5106-4f-rac-IE-95%

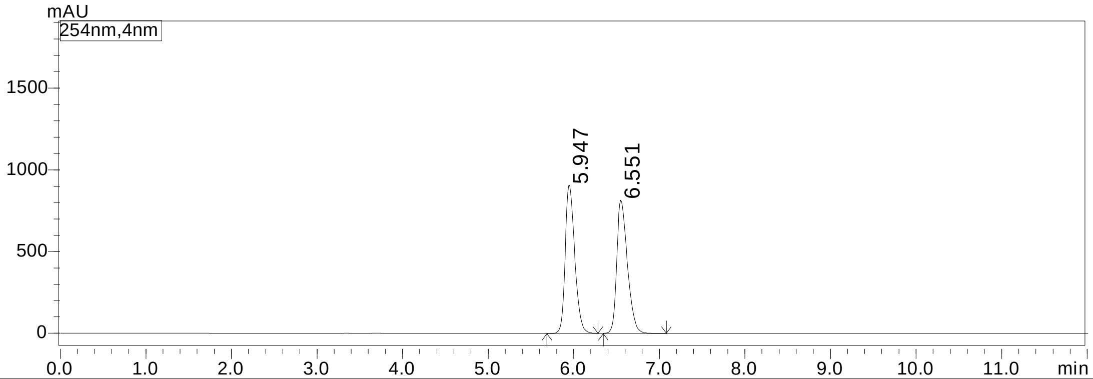

Peak	Ret. Time	Area	Height	Area%
1	5.947	7018629	904721	49.836
2	6.551	7064818	815664	50.164
Total		14083448	1720385	100.000

Supplementary Figure 144. HPLC Spectrum of racemic **4f**

数据文件名:PQP-5106-4f-IE-95%.lcd
样品名:PQP-5106-4f-IE-95%
样品ID:PQP-5106-4f-IE-95%

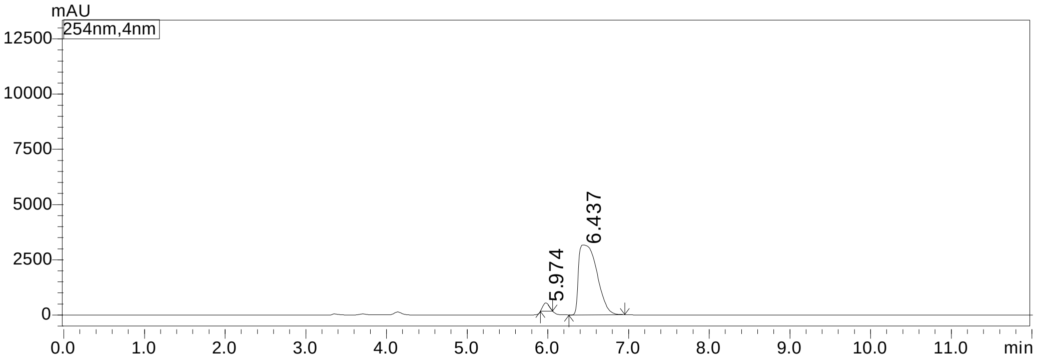

Peak	Ret. Time	Area	Height	Area%
1	5.974	1958034	375832	3.772
2	6.437	49952597	3154895	96.228
Total		51910631	3530727	100.000

Supplementary Figure 145. HPLC Spectrum of **4f**

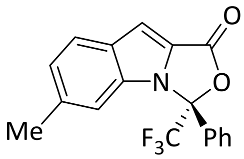

4g

4g (*R*)-6-methyl-3-phenyl-3-(trifluoromethyl)-1*H*,3*H*-oxazolo[3,4-a]indol-1-one

数据文件名:PQP-5106-4-IE-95%.lcd
样品名:PQP-5106-4-IE-95%
样品ID:PQP-5106-4-IE-95%

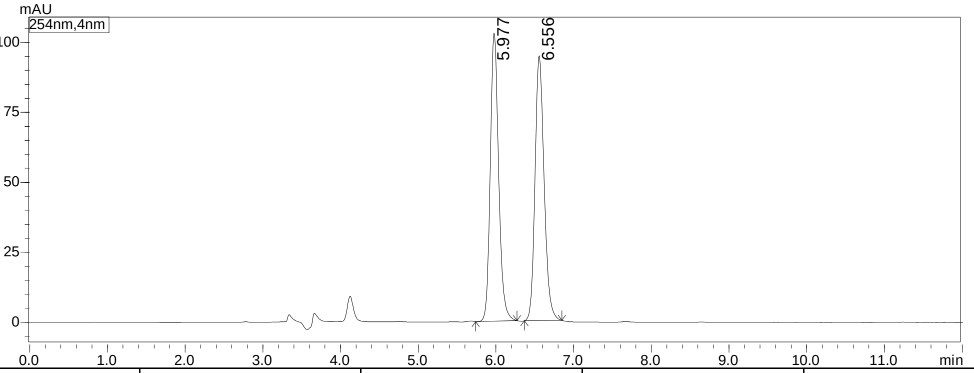

Peak	Ret. Time	Area	Height	Area%
1	5.977	770735	102891	49.993
2	6.556	770936	94578	50.007
Total		1541671	197469	100.000

Supplementary Figure 146. HPLC Spectrum of racemic **4g**

数据文件名:PQP-5104-4-IE-95%.lcd
样品名:PQP-5104-4-IE-95%
样品ID:PQP-5104-4-IE-95%

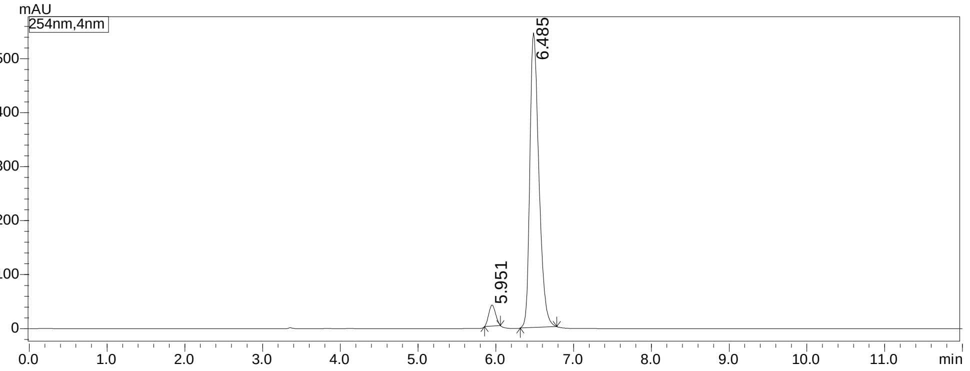

Peak	Ret. Time	Area	Height	Area%
1	5.951	237347	39037	5.103
2	6.485	4414174	546091	94.897
Total		4651520	585128	100.000

Supplementary Figure 147. HPLC Spectrum of **4g**

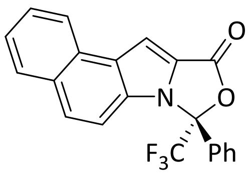

4h

4h (*R*)-8-phenyl-8-(trifluoromethyl)-8*H*,10*H*-benzo[e]oxazolo[3,4-a]indol-10-one

数据文件名:PQP-5134-2-rac-IE-95%.lcd
样品名:PQP-5134-2-rac-IE-95%
样品ID:PQP-5134-2-rac-IE-95%

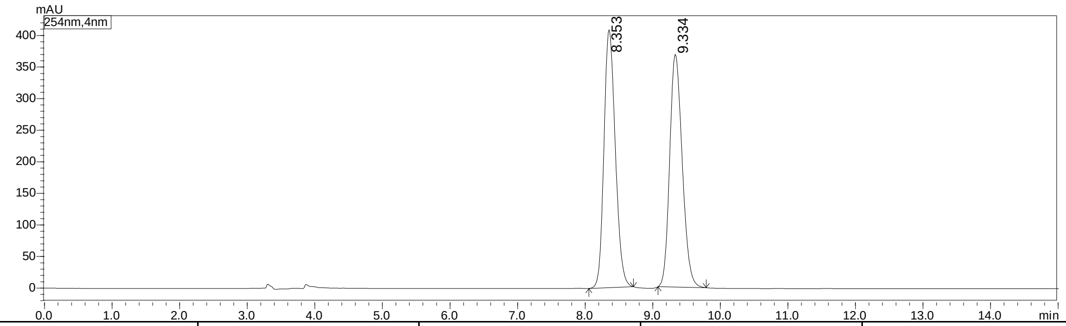

Peak	Ret. Time	Area	Height	Area%
1	8.353	4776086	408420	50.114
2	9.334	4754266	368561	49.886
Total		9530351	776982	100.000

Supplementary Figure 148. HPLC Spectrum of racemic **4h**

数据文件名:PQP-5101-4-IE-95%.lcd
样品名:PQP-5101-4-IE-95%
样品ID:PQP-5101-4-IE-95%

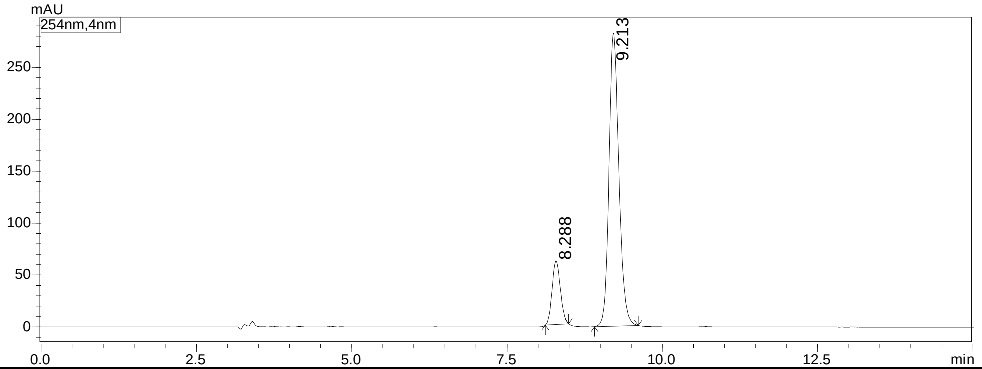

Peak	Ret. Time	Area	Height	Area%
1	8.288	566900	61312	15.331
2	9.213	3130822	282030	84.669
Total		3697722	343341	100.000

Supplementary Figure 149. HPLC Spectrum of **4h**

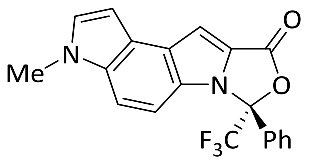

4i

4i (*R*)-3-methyl-7-phenyl-7-(trifluoromethyl)-7*H*-oxazolo[3,4-a]pyrrolo[3,2-e]indol-9(3*H*)-one

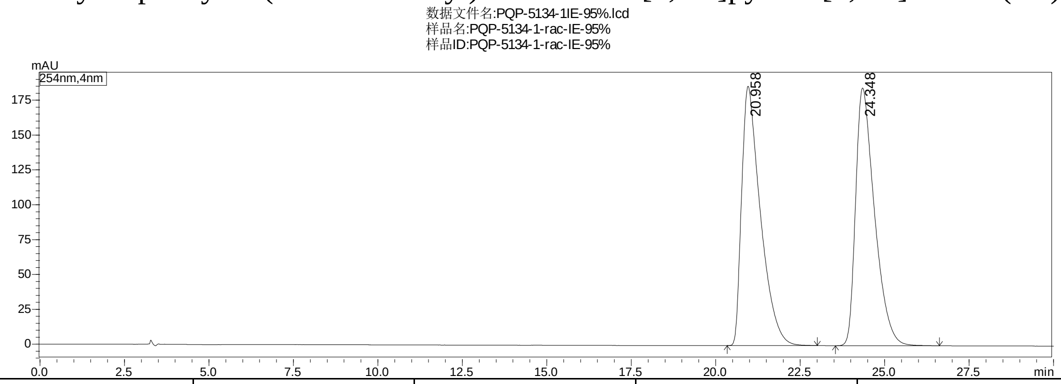

Peak	Ret. Time	Area	Height	Area%
1	20.958	7197432	186035	49.994
2	24.348	7199195	184879	50.006
Total		14396627	370913	100.000

Supplementary Figure 150. HPLC Spectrum of racemic **4i**

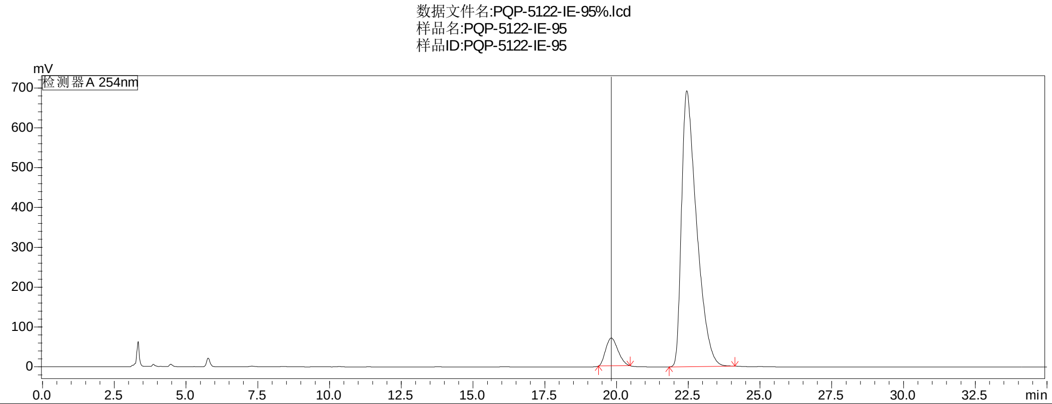

Peak	Ret. Time	Area	Height	Area%
1	19.820	2081907	69956	7.599
2	22.449	25314236	692725	92.401
Total		27396143	762682	100.000

Supplementary Figure 151. HPLC Spectrum of **4i**

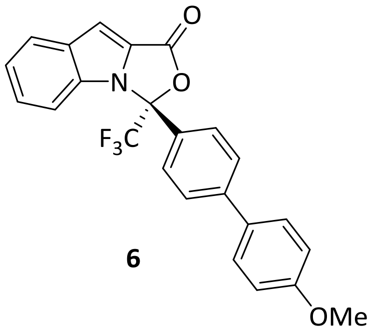

6 (R)-3-(4'-methoxy-[1,1'-biphenyl]-4-yl)-3-(trifluoromethyl)-1H,3H-oxazolo[3,4-a]indol-1-one

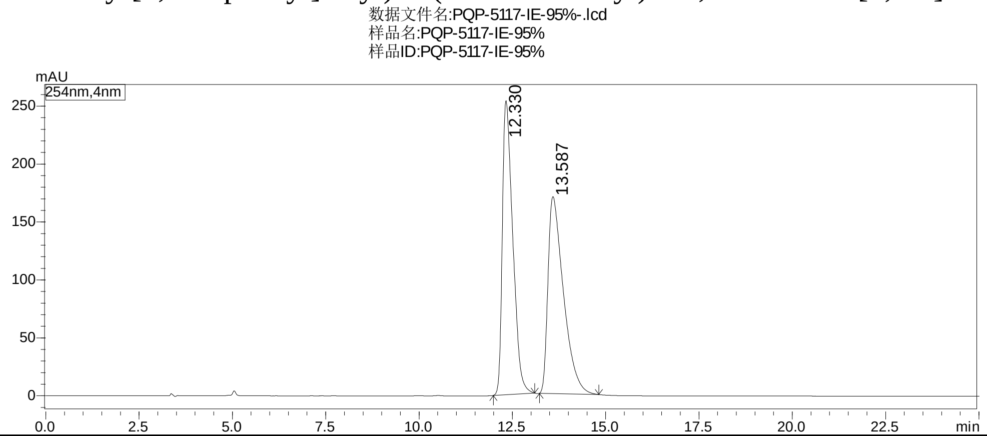

Peak	Ret. Time	Area	Height	Area%
1	12.330	4924151	253883	50.079
2	13.587	4908525	170010	49.921
Total		9832676	423893	100.000

Supplementary Figure 152. HPLC Spectrum of racemic **6**

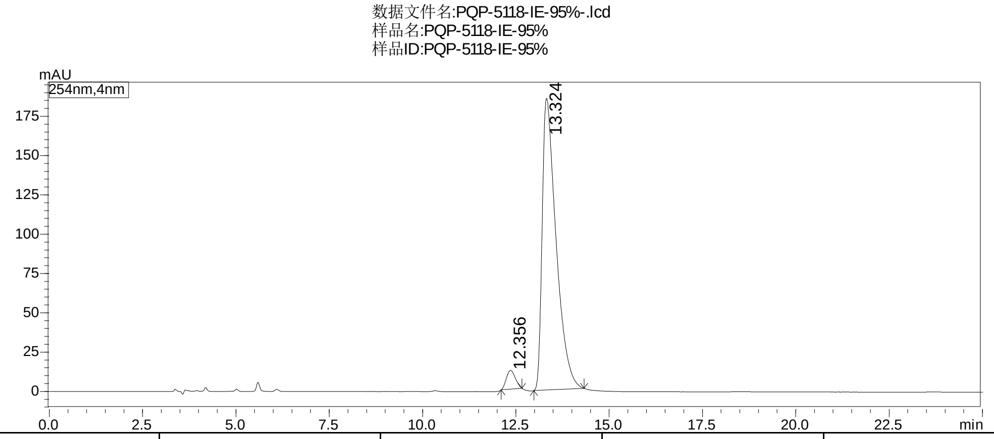

Peak	Ret. Time	Area	Height	Area%
1	12.356	199902	11937	3.989
2	13.324	4811413	185593	96.011
Total		5011315	197530	100.000

Supplementary Figure 153. HPLC Spectrum of **6**

Supplementary Methods
General Information

Chemicals were purchased from commercial suppliers and used as received. Solvents were dried on alumina columns using a solvent dispensing system. Thin-layer chromatography (TLC) was conducted on plates (GF254) supplied by Yantai Chemicals (China) and visualized using a combination of UV, anisaldehyde, iodine, and potassium permanganate staining. ^{1}H NMR, ^{13}C NMR, ^{19}F NMR, spectra were recorded on a Bruker ACF400 (400 MHz) spectrometer. Chemical shifts were reported in parts per million (ppm), and the residual solvent peak was used as an internal reference: proton (chloroform δ 7.26, DMSO-d_6 δ 2.50), carbon (chloroform δ 77.16, DMSO-d_6 δ 39.52) or tetramethylsilane (TMS δ 0.00) was used as a reference. Multiplicity was indicated as follows: s (singlet), d (doublet), t (triplet), q (quartet), m (multiplet), dd (doublet of doublet), bs (broad singlet). Coupling constants were reported in Hertz (Hz). All high resolution mass spectra were obtained from the Tsinghua University Mass Spectrometry Facility. Flash chromatography separations were performed on Silica gel (300-400 mesh) supplied by Tsingdao Haiyang Chemicals (China). The enantiomeric excesses of products were determined on a Shimadzu LC-20AT Chiral HPLC. Infrared spectra were recorded on a Perkin Elmer Spectrum two equipped with MIRacle™ single reflection with ZnSe ATR unit. Fluorinated ketones and indole-2-carbaldehydes were prepared by literatures[1-5] or commercial available.

Procedure for Synthesis of Catalyst F

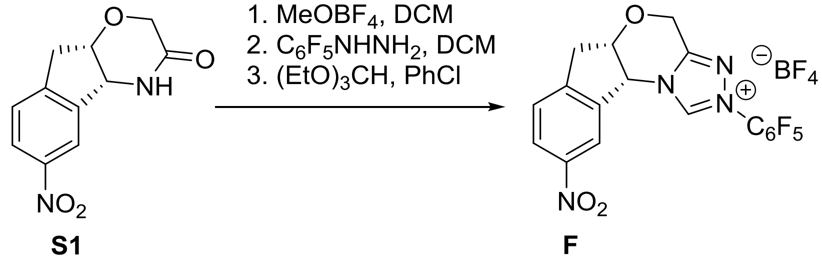

To a solution of **S1**[6] (6.0 g, 25.6 mmol) in CH_2Cl_2 (135 mL) was added Me_3OBF_4 (4.17 g, 28.2 mmol), and the mixture was stirred at rt for 24 h. Then, $C_6F_5NHNH_2$ (5.58 g, 28.2 mmol) was added, and the mixture was stirred for another 36 h. The mixture was concentrated in vacuo, and the residue was dissolved in PhCl (60 mL) followed by the addition of $(EtO)_3CH$ (60 mL). The mixture was heated to 130 °C for 60 h. The solvent was removed on a rotary evaporator, the crude mixture was purified by column chromatography (DCM: MeOH = 100:1 to 40:1) to give

(5a*S*,10b*R*)-9-nitro-2-(perfluorophenyl)-5a,10b-dihydro-4*H*,6*H*-indeno[2,1-*b*][1,2,4]triazolo[4,3-*d*][1,4]oxazin-2-ium tetrafluoroborate **F** as a white solid, 5.56 g, 42% yield. **^{1}H NMR** (400 MHz, DMSO-d_6) δ 11.63 (s, 1H), 8.48 (s, 1H), 8.31 (d, *J* = 8.5 Hz, 1H), 7.74 (d, *J* = 8.4 Hz, 1H), 6.26 (s, 1H), 5.40 (d, *J* = 16.3 Hz, 1H), 5.07 (d, *J* = 15.2 Hz, 2H), 3.64 (d, *J* = 17.9 Hz, 1H), 3.33 (d, *J* = 19.8 Hz, 1H). **^{13}C NMR** (100 MHz, DMSO-d_6) δ 151.1, 149.5, 147.7, 147.5, 143.7 (d, *J* = 13.1 Hz), 141.1 (d, *J* = 11.7 Hz), 140.0 – 139.2 (m), 137.8, 127.1, 125.4, 121.1, 111.5, 77.7, 61.7, 60.3, 37.6. **^{19}F NMR** (376 MHz, DMSO-d_6) δ -145.67 (d, *J* = 19.7 Hz), -148.00 (t, *J* = 23.2 Hz), -148.25, -148.31, -159.78 (t, *J* = 21.5 Hz). ; **HRMS** (ESI) exact mass calcd. for $C_{18}H_{10}F_5N_4O_3$ ($[M - BF_4]^+$) requires m/z 425.0668, found m/z 425.0674; $[\alpha]^{25}_D$ = -96.2 (*c* = 1.0, DCM).

General Procedure for Catalytic Reaction of Indole-2-carbaldehydes with Ketones

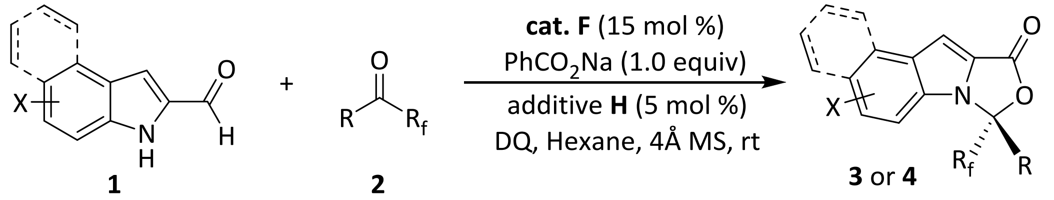

To a flame-dried Schlenk reaction tube equipped with a magnetic stir bar, was added the precatalyst **F** (15.4 mg, 0.03 mmol), DQ (90.0 mg, 0.22 mmol), additive **H** (5.0 mg, 0.01 mmol), $PhCO_2Na$ (28.8 mg, 0.20 mmol), **1** (0.20 mmol) and 4Å MS (60 mg). The Schlenk tube was closed with a septum, evacuated and refilled with argon atmosphere. Hexane (2.0 mL) and **2** (0.24 mmol) was added. The mixture was then stirred at 25 ℃ and monitored by TLC until **1** was consumed. The mixture was concentrated under reduced pressure and purified by column chromatography on silica gel (hexane/EtOAc = 100:1) to afford the desired product **3** or **4**.

Characterization Data

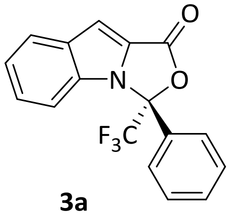

3a

3a (*R*)-3-phenyl-3-(trifluoromethyl)-1*H,*3*H*-oxazolo[3,4-a]indol-1-one

Pale yellow oil, 53.9 mg, 85% yield, 48 h; **^{1}H NMR** (400 MHz, Chloroform-*d*) δ 7.85 (dd, *J* = 8.0, 1.3 Hz, 1H), 7.62 (d, *J* = 7.4 Hz, 2H), 7.58 – 7.50 (m, 1H), 7.52 – 7.38 (m, 3H), 7.37 – 7.28 (m, 3H). **^{13}C NMR** (100 MHz, Chloroform-*d*) δ 157.9, 133.9, 133.1, 131.5, 130.0, 129.3, 127.0, 127.0, 124.7, 124.5, 122.8, 122.3 (q, *J* = 288.1 Hz), 112.5 (q, *J* = 2.4 Hz), 104.7, 92.3 (q, *J* = 34.6 Hz). **^{19}F NMR** (376 MHz, Chloroform-*d*) δ -77.04; **IR** (neat, ATR) 1804, 1787, 1186, 1140, 949, 898, 722, 692 cm^{-1}; **HRMS** (ESI) exact mass calcd. for $C_{17}H_{11}F_3NO_2$ ([M + H]$^{+}$) requires m/z 318.0736, found m/z 318.0733; **HPLC** (Chiralpak IE, *i*-propanol/hexane = 5/95, flow rate 1.0 mL/min, 25 ^{o}C, λ = 254 nm): t_R (major) = 6.3 min, t_R (minor) = 5.8 min, *ee* = 91%; $[\alpha]^{25}_{D}$ = -42.7 (*c* = 1.0, DCM).

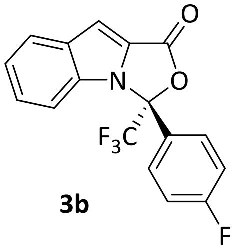

3b

3b (*R*)-3-(4-fluorophenyl)-3-(trifluoromethyl)-1*H*,3*H*-oxazolo[3,4-a]indol-1-one

Pale yellow oil, 55.2 mg, 82% yield, 48 h; **^{1}H NMR** (400 MHz, Chloroform-*d*) δ 7.85 (d, *J* = 8.1 Hz, 1H), 7.61 (dd, *J* = 8.8, 4.9 Hz, 2H), 7.47 – 7.40 (m, 1H), 7.35 – 7.29 (m, 3H), 7.20 – 7.11 (m, 2H). **^{13}C NMR** (100 MHz, Chloroform-*d*) δ 164.3 (d, *J* = 253.1 Hz), 157.6, 133.8, 133.1, 129.5 (dq, *J* = 9.0, 2.1 Hz), 127.2, 126.0 (d, *J* = 3.6 Hz), 124.6, 124.6, 123.0, 122.2 (q, *J* = 288.2 Hz), 116.5 (d, *J* = 22.2 Hz), 112.3 (q, *J* = 2.5 Hz), 104.9, 91.8 (q, *J* = 34.7 Hz). **^{19}F NMR** (376 MHz, Chloroform-*d*) δ -77.23, -107.71; **IR** (neat, ATR) 1807, 1257, 1200, 1185, 897, 797,725 cm^{-1}; **HRMS** (ESI) exact mass calcd. for $C_{17}H_{10}F_4NO_2$ ([M + H]$^{+}$) requires m/z 336.0642, found m/z 336.0638; **HPLC** (Chiralpak IE, *i*-propanol/hexane = 5/95, flow rate 1.0 mL/min, 25 ^{o}C, λ = 254 nm): t_R (major) = 5.6 min, t_R (minor) = 5.4 min, *ee* = 91%; $[\alpha]^{25}_{D}$ = -89.4 (*c* = 1.0, DCM).

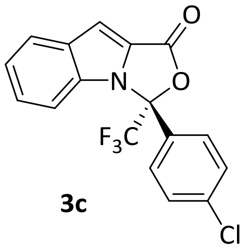

3c

3c (*R*)-3-(4-chlorophenyl)-3-(trifluoromethyl)-1*H*,3*H*-oxazolo[3,4-a]indol-1-one

Yellow oil, 56.1 mg, 80% yield, 48 h; ^{1}**H NMR** (400 MHz, Chloroform-*d*) δ 7.85 (d, *J* = 8.2 Hz, 1H), 7.55 (d, *J* = 8.5 Hz, 2H), 7.47 – 7.42 (m, 3H), 7.38 – 7.28 (m, 3H). ^{13}**C NMR** (100 MHz, Chloroform-*d*) δ 157.5, 138.0, 133.8, 133.1, 129.6, 128.5 (q, *J* = 2.1 Hz), 127.2, 126.4, 124.6, 124.5, 123.0, 122.1 (q, *J* = 288.2 Hz), 112.3 (q, *J* = 2.5 Hz), 105.0, 91.8 (q, *J* = 34.6 Hz). ^{19}**F NMR** (376 MHz, Chloroform-*d*) δ -77.19; **IR** (neat, ATR) 1803, 1785, 1740, 1373, 1188, 1097, 1008, 941, 837, 739 cm^{-1}; **HRMS** (ESI) exact mass calcd. for $C_{17}H_{10}ClF_3NO_2$ ([M + H]$^+$) requires m/z 352.0347, found m/z 352.0344; **HPLC** (Chiralpak IE, *i*-propanol/hexane = 5/95, flow rate 1.0 mL/min, 25 ^{o}C, λ = 254 nm): t_R (major) = 5.9 min, t_R (minor) = 5.6 min, *ee* = 91%; $[\alpha]^{25}_{D}$ = -67.1 (*c* = 1.0, DCM).

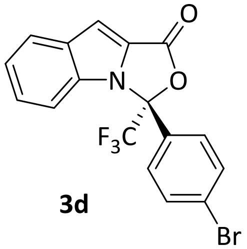

3d

3d (*R*)-3-(4-bromophenyl)-3-(trifluoromethyl)-1*H*,3*H*-oxazolo[3,4-a]indol-1-one

Pale yellow oil, 68.9 mg, 87% yield, 48 h; ^{1}**H NMR** (400 MHz, Chloroform-*d*) δ 7.85 (dd, *J* = 8.2, 1.3 Hz, 1H), 7.64 – 7.57 (m, 2H), 7.54 – 7.41 (m, 3H), 7.39 – 7.28 (m, 3H). ^{13}**C NMR** (100 MHz, Chloroform-*d*) δ 157.5, 133.8, 133.1, 132.6, 129.1, 128.7 (q, *J* = 2.1 Hz), 127.2, 126.3, 124.6, 124.5, 123.0, 122.1 (q, *J* = 288.2 Hz), 112.3 (q, *J* = 2.4 Hz), 105.0, 91.9 (q, *J* = 34.5 Hz). ^{19}**F NMR** (376 MHz, Chloroform-*d*) δ -77.18; **IR** (neat, ATR) 1808, 1739, 1363, 1185, 1165, 1183, 1003, 941, 814, 739 cm^{-1}; **HRMS** (ESI) exact mass calcd. for $C_{17}H_{10}BrF_3NO_2$ ([M + H]$^+$) requires m/z 395.9842, found m/z 395.9842; **HPLC** (Chiralpak IE, *i*-propanol/hexane = 5/95, flow rate 1.0 mL/min, 25 ^{o}C, λ = 254 nm): t_R (major) = 6.3 min, t_R (minor) = 6.0 min, *ee* = 91%; $[\alpha]^{25}_{D}$ = -64.8 (*c* = 1.0, DCM).

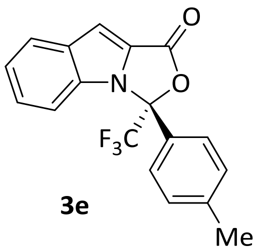

3e (*R*)-3-(p-tolyl)-3-(trifluoromethyl)-1*H*,3*H*-oxazolo[3,4-a]indol-1-one

white solid, 45.7 mg, 69% yield, 72 h; **^{1}H NMR** (400 MHz, Chloroform-*d*) δ 7.83 (d, *J* = 7.6 Hz, 1H), 7.47 (d, *J* = 8.1 Hz, 2H), 7.43 – 7.36 (m, 1H), 7.33 – 7.22 (m, 5H), 2.38 (s, 3H). **^{13}C NMR** (100 MHz, Chloroform-*d*) δ 158.0, 141.9, 133.8, 133.1, 129.9, 126.9, 126.9, 126.9, 124.8, 124.4, 122.8, 122.3 (q, *J* = 288.3 Hz), 112.5 (q, *J* = 2.3 Hz), 104.4, 92.4 (q, *J* = 34.4 Hz), 21.3. **^{19}F NMR** (376 MHz, Chloroform-*d*) δ -77.03; **IR** (neat, ATR) 1801, 1785, 1376, 1187, 1088, 1007, 941, 897, 731 cm^{-1}; **HRMS** (ESI) exact mass calcd. for $C_{18}H_{13}F_3NO_2$ ($[M + H]^+$) requires m/z 332.0893, found m/z 332.0895; **HPLC** (Chiralpak IE, *i*-propanol/hexane = 5/95, flow rate 1.0 mL/min, 25 ^{o}C, λ = 254 nm): t_R (major) = 6.4 min, t_R (minor) = 6.0 min, *ee* = 93%; $[\alpha]^{25}_{D}$ = -136.2 (*c* = 1.0, DCM).

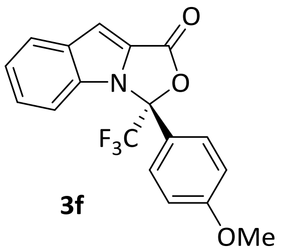

3f (*R*)-3-(4-methoxyphenyl)-3-(trifluoromethyl)-1*H*,3*H*-oxazolo[3,4-a]indol-1-one

Yellow wax, 53.4 mg, 77% yield, 96 h; **^{1}H NMR** (400 MHz, Chloroform-*d*) δ 7.83 (d, *J* = 7.7 Hz, 1H), 7.50 (d, *J* = 8.7 Hz, 2H), 7.45 – 7.36 (m, 1H), 7.34 – 7.27 (m, 3H), 6.97 – 6.92 (m, 2H), 3.82 (s, 3H). **^{13}C NMR** (100 MHz, Chloroform-*d*) δ 161.8, 158.0, 133.8, 133.0, 128.7 (q, *J* = 2.1 Hz), 126.9, 124.9, 124.4, 122.8, 122.4 (q, *J* = 288.3 Hz), 121.6, 114.5, 112.5 (q, *J* = 2.3 Hz), 104.4, 92.4 (q, *J* = 34.5 Hz), 55.4. **^{19}F NMR** (376 MHz, Chloroform-*d*) δ -77.08; **IR** (neat, ATR) 1805, 1612, 1517, 1441, 1177, 1087, 952, 833, 740 cm^{-1}; **HRMS** (ESI) exact mass calcd. for $C_{18}H_{13}F_3NO_3$ ($[M + H]^+$) requires m/z 348.0842, found m/z 348.0840
; **HPLC** (Chiralpak IE, *i*-propanol/hexane = 5/95, flow rate 1.0 mL/min, 25 ^{o}C, λ = 254 nm): t_R (major) = 8.7 min, t_R (minor) = 7.7 min, *ee* = 91%; $[\alpha]^{25}_{D}$ = -83.1 (*c* = 1.0, DCM).

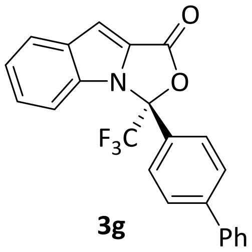

3g

3g (*R*)-3-([1,1'-biphenyl]-4-yl)-3-(trifluoromethyl)-1*H*,3*H*-oxazolo[3,4-a]indol-1-one

Colorless wax, 70.4 mg, 90% yield, 48 h; **^{1}H NMR** (400 MHz, Chloroform-*d*) δ 7.88 (d, *J* = 8.2 Hz, 1H), 7.73 – 7.66 (m, 4H), 7.60 (d, *J* = 7.4 Hz, 2H), 7.51 – 7.39 (m, 5H), 7.38 – 7.32 (m, 2H). **^{13}C NMR** (100 MHz, Chloroform-*d*) δ 157.9, 144.4, 139.4, 133.9, 133.1, 129.1, 128.7, 128.3, 127.9, 127.5 (q, *J* = 2.2 Hz), 127.2, 127.1, 124.8, 124.5, 122.9, 122.4 (q, *J* = 288.2 Hz), 112.6 (d, *J* = 2.4 Hz), 104.7, 92.4 (q, *J* = 34.6 Hz). **^{19}F NMR** (376 MHz, Chloroform-*d*) δ -76.96; **IR** (neat, ATR) 1807, 1440, 1343, 1109, 952, 935, 726 cm^{-1}; **HRMS** (ESI) exact mass calcd. for $C_{23}H_{15}F_{3}NO_{2}$ ([M + H]$^{+}$) requires m/z 394.1049, found m/z 394.1044; **HPLC** (Chiralpak IE, *i*-propanol/hexane = 5/95, flow rate 1.0 mL/min, 25 ^{o}C, λ = 254 nm): t_R (major) = 8.5 min, t_R (minor) = 8.0 min, *ee* = 93%; $[\alpha]^{25}_{D}$ = -116.0 (*c* = 1.0, DCM).

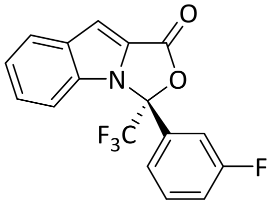

3h

3h (*R*)-3-(3-fluorophenyl)-3-(trifluoromethyl)-1*H*,3*H*-oxazolo[3,4-a]indol-1-one

Pale yellow wax, 55.2 mg, 82% yield, 48 h; **^{1}H NMR** (400 MHz, Chloroform-*d*) δ 7.85 (d, *J* = 8.1 Hz, 1H), 7.51 – 7.40 (m, 3H), 7.39 – 7.29 (m, 4H), 7.29 – 7.19 (m, 1H). **^{13}C NMR** (100 MHz, Chloroform-*d*) δ 162.8 (d, *J* = 248.9 Hz), 157.5, 133.8, 133.1, 132.3 (d, *J* = 7.2 Hz), 131.1 (d, *J* = 8.1 Hz), 127.3, 124.6, 124.4, 123.0, 122.8 (d, *J* = 1.7 Hz), 122.1 (q, *J* = 288.2 Hz), 118.7 (d, *J* = 20.9 Hz), 114.7 (dq, *J* = 24.6, 2.1 Hz), 112.3 (q, *J* = 2.5 Hz), 105.1, 91.6 (q, *J* = 34.8 Hz). **^{19}F NMR** (376 MHz, Chloroform-*d*) δ -77.20, -109.69; **IR** (neat, ATR) 1801, 1381, 1233, 1183, 1091, 999, 948, 846, 728 cm^{-1}; **HRMS** (ESI) exact mass calcd. for $C_{17}H_{10}F_{4}NO_{2}$ ([M + H]$^{+}$) requires m/z 336.0642, found m/z 336.0638; **HPLC** (Chiralpak IE, *i*-propanol/hexane = 5/95, flow rate 1.0 mL/min, 25 ^{o}C, λ = 254 nm): t_R (major) = 5.7 min, t_R (minor) = 5.2 min, *ee* = 87%; $[\alpha]^{25}_{D}$ = -87.0 (*c* = 1.0, DCM).

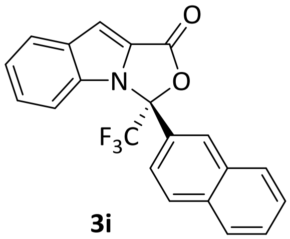

3i

3i (*R*)-3-(naphthalen-2-yl)-3-(trifluoromethyl)-1*H*,3*H*-oxazolo[3,4-a]indol-1-one

Colorless wax, 63.5 mg, 86% yield, 36 h; **^{1}H NMR** (400 MHz, Chloroform-*d*) δ 8.21 (d, *J* = 2.0 Hz, 1H), 7.92 (d, *J* = 8.8 Hz, 1H), 7.90 – 7.85 (m, 3H), 7.65 – 7.53 (m, 3H), 7.46 – 7.31 (m, 4H). **^{13}C NMR** (100 MHz, Chloroform-*d*) δ 158.0, 134.2, 133.9, 133.1, 132.5, 129.5, 129.0, 128.3, 127.7, 127.7 (q, *J* = 2.0 Hz), 127.3, 127.1, 127.1, 124.8, 124.5, 123.0 (q, *J* = 1.9 Hz), 122.9, 122.4 (q, *J* = 288.4 Hz), 112.5 (q, *J* = 2.3 Hz), 104.8, 92.5 (q, *J* = 34.4 Hz). **^{19}F NMR** (376 MHz, Chloroform-*d*) δ -76.57; **IR** (neat, ATR) 1805, 1788, 1375, 1247, 1188, 1010, 955, 900, 844, 733 cm^{-1}; **HRMS** (ESI) exact mass calcd. for $C_{21}H_{13}F_3NO_2$ ([M + H]$^+$) requires m/z 368.0893, found m/z 368.0898; **HPLC** (Chiralpak IE, *i*-propanol/hexane = 5/95, flow rate 1.0 mL/min, 25 ^{o}C, λ = 254 nm): t_R (major) = 7.8 min, t_R (minor) = 7.0 min, *ee* = 92%; $[\alpha]^{25}_{D}$ = -68.6 (*c* = 1.0, DCM).

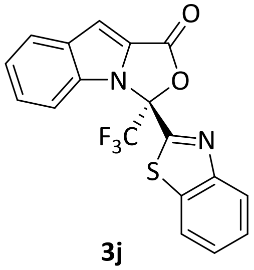

3j

3j (*S*)-3-(benzo[d]thiazol-2-yl)-3-(trifluoromethyl)-1*H*,3*H*-oxazolo[3,4-a]indol-1-one

Pale yellow wax, 60.4 mg, 81% yield, 48 h; **^{1}H NMR** (400 MHz, Chloroform-*d*) δ 8.21 (d, *J* = 8.2 Hz, 1H), 8.14 (d, *J* = 8.5 Hz, 1H), 7.94 (d, *J* = 8.0 Hz, 1H), 7.80 (d, *J* = 8.3 Hz, 1H), 7.62 – 7.47 (m, 3H), 7.39 – 7.29 (m, 2H). **^{13}C NMR** (100 MHz, Chloroform-*d*) δ 159.1, 156.7, 153.1, 134.9, 134.1, 133.1, 127.3, 127.1, 127.1, 124.7, 124.1, 123.2, 122.5, 122.0, 121.4 (q, *J* = 289.0 Hz), 114.2 (d, *J* = 1.9 Hz), 105.9, 89.6 (q, *J* = 36.0 Hz). **^{19}F NMR** (376 MHz, Chloroform-*d*) δ -78.60; **IR** (neat, ATR) 1804, 1343, 1238, 1179, 1141, 969, 814, 727 cm^{-1}; **HRMS** (ESI) exact mass calcd. for $C_{18}H_{10}F_3N_2O_2S$ ([M + H]$^+$) requires m/z 375.0410, found m/z 375.0412; **HPLC** (Chiralpak IE, *i*-propanol/hexane = 5/95, flow rate 1.0 mL/min, 25 ^{o}C, λ = 254 nm): t_R (major) = 6.1 min, t_R (minor) = 5.6 min, *ee* = 82%; $[\alpha]^{25}_{D}$ = -65.7 (*c* = 1.0, DCM).

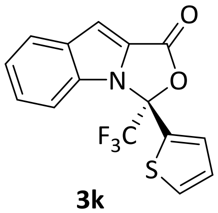

3k

3k (*S*)-3-(thiophen-2-yl)-3-(trifluoromethyl)-1*H*,3*H*-oxazolo[3,4-a]indol-1-one

Pale yellow wax, 44.3 mg, 69% yield, 48 h; **^{1}H NMR** (400 MHz, Chloroform-*d*) δ 7.83 (d, *J* = 8.2 Hz, 1H), 7.53 (dd, *J* = 5.1, 1.2 Hz, 1H), 7.47 – 7.38 (m, 2H), 7.39 – 7.27 (m, 3H), 7.10 (dd, *J* = 5.1, 3.8 Hz, 1H). **^{13}C NMR** (100 MHz, Chloroform-*d*) δ 157.4, 133.5, 133.1, 131.6, 130.1 (q, *J* = 1.9 Hz), 130.0, 127.6, 127.0, 124.4, 124.3, 123.0, 121.9 (q, *J* = 288.0 Hz), 112.6 (q, *J* = 2.2 Hz), 105.0, 90.0 (q, *J* = 36.1 Hz). **^{19}F NMR** (376 MHz, Chloroform-*d*) δ -78.70; **IR** (neat, ATR) 1800, 1739, 1366, 1259, 1192, 1145, 996, 897, 720 cm^{-1}; **HRMS** (ESI) exact mass calcd. for $C_{15}H_9F_3NO_2S$ ($[M + H]^+$) requires m/z 324.0301, found m/z 324.0300; **HPLC** (Chiralpak IE, *i*-propanol/hexane = 5/95, flow rate 1.0 mL/min, 25 ^{o}C, λ = 254 nm): t_R (major) = 6.6 min, t_R (minor) = 6.1 min, *ee* = 80%; $[\alpha]^{25}_{D}$ = -120.3 (*c* = 1.0, DCM).

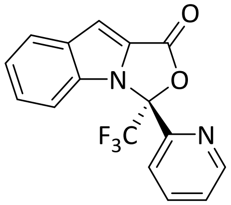

3l

3l (*R*)-3-(pyridin-2-yl)-3-(trifluoromethyl)-1*H*,3*H*-oxazolo[3,4-a]indol-1-one

Yellow wax, 57.2 mg, 90% yield, 36 h; **^{1}H NMR** (400 MHz, Chloroform-*d*) δ 8.76 (d, *J* = 4.8 Hz, 1H), 8.19 (d, *J* = 8.6 Hz, 1H), 7.96 – 7.76 (m, 3H), 7.58 – 7.40 (m, 2H), 7.37 – 7.29 (m, 2H). **^{13}C NMR** (100 MHz, Chloroform-*d*) δ 157.8, 150.3, 149.4, 137.6, 134.8, 133.0, 126.7, 125.6, 123.8, 123.5, 122.8, 122.1 (q, *J* = 1.5 Hz), 122.0 (q, *J* = 288.7 Hz), 115.2 (q, *J* = 1.9 Hz), 104.8, 91.6 (q, *J* = 34.1 Hz). **^{19}F NMR** (376 MHz, Chloroform-*d*) δ -78.42; **IR** (neat, ATR) 1808, 1574, 1558, 1439, 1373, 1269, 1103, 953, 895, 743 cm^{-1}; **HRMS** (ESI) exact mass calcd. for $C_{16}H_{10}F_3N_2O_2$ ($[M + H]^+$) requires m/z 319.0689, found m/z 319.0693; **HPLC** (Chiralpak ASH, *i*-propanol/hexane = 5/95, flow rate 1.0 mL/min, 25 ^{o}C, λ = 254 nm): t_R (major) = 6.4 min, t_R (minor) = 8.6 min, *ee* = 93%; $[\alpha]^{25}_{D}$ = -115.9 (*c* = 1.0, DCM).

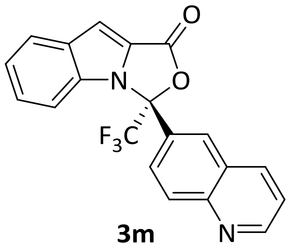
3m

3m (*R*)-3-(quinolin-6-yl)-3-(trifluoromethyl)-1*H*,3*H*-oxazolo[3,4-a]indol-1-one

Yellow oil, 61.1 mg, 84% yield, 96 h; **^{1}H NMR** (400 MHz, Chloroform-*d*) δ 9.02 (dd, *J* = 4.3, 1.7 Hz, 1H), 8.25 – 8.16 (m, 3H), 7.86 (d, *J* = 8.2 Hz, 1H), 7.82 (dd, *J* = 9.0, 2.3 Hz, 1H), 7.50 (dd, *J* = 8.3, 4.3 Hz, 1H), 7.45 – 7.39 (m, 1H), 7.37 – 7.29 (m, 3H). **^{13}C NMR** (100 MHz, Chloroform-*d*) δ 157.6, 152.5, 148.5, 137.3, 133.9, 133.2, 131.0, 128.2, 127.8 (q, *J* = 2.1 Hz), 127.5, 127.3, 126.8 (q, *J* = 2.0 Hz), 124.6, 124.5, 123.0, 122.4, 122.3 (q, *J* = 288.4 Hz), 112.3 (q, *J* = 2.4 Hz), 105.1, 92.1 (q, *J* = 34.8 Hz). **^{19}F NMR** (376 MHz, Chloroform-*d*) δ -76.73; **IR** (neat, ATR) 1805, 1439, 1261, 1238, 1021, 935, 902, 834, 728 cm^{-1}; **HRMS** (ESI) exact mass calcd. for $C_{20}H_{12}F_3N_2O_2$ ([M + H]$^{+}$) requires m/z 369.0845, found m/z 369.0844; **HPLC** (Chiralpak IE, *i*-propanol/hexane = 10/90, flow rate 1.0 mL/min, 25 ^{o}C, λ = 254 nm): t_R (major) = 19.0 min, t_R (minor) = 17.2 min, *ee* = 85%; $[\alpha]^{25}_{D}$ = -88.2 (*c* = 1.0, DCM).

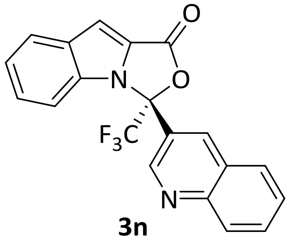
3n

3n (*R*)-3-(quinolin-3-yl)-3-(trifluoromethyl)-1*H*,3*H*-oxazolo[3,4-a]indol-1-one

Yellow wax, 46.4 mg, 63% yield, 48 h; **^{1}H NMR** (400 MHz, Chloroform-*d*) δ 9.05 (d, *J* = 2.4 Hz, 1H), 8.51 (d, *J* = 2.5 Hz, 1H), 8.18 (d, *J* = 8.4 Hz, 1H), 7.96 – 7.81 (m, 3H), 7.69 – 7.61 (m, 1H), 7.47 – 7.42 (m, 1H), 7.40 – 7.31 (m, 3H). **^{13}C NMR** (100 MHz, Chloroform-*d*) δ 157.3, 148.7, 147.2 (q, *J* = 2.4 Hz), 136.0 (d, *J* = 1.9 Hz), 133.8, 133.2, 132.1, 129.4, 128.7, 128.2, 127.5, 126.5, 124.7, 124.2, 123.3, 123.2, 122.1 (q, *J* = 288.4 Hz), 112.2 (q, *J* = 2.5 Hz), 105.6, 91.4 (q, *J* = 35.3 Hz). **^{19}F NMR** (376 MHz, Chloroform-*d*) δ -77.24; **IR** (neat, ATR) 1806, 1573, 1495, 1342, 1275, 1181, 1031, 996, 938, 839, 727 cm^{-1}; **HRMS** (ESI) exact mass calcd. for $C_{20}H_{12}F_3N_2O_2$ ([M + H]$^{+}$) requires m/z 369.0845, found m/z 369.0845; **HPLC**

(Chiralpak IE, *i*-propanol/hexane = 5/95, flow rate 1.0 mL/min, 25 °C, λ = 254 nm): t_R (major) = 7.0 min, t_R (minor) = 8.2 min, *ee* = 88%; $[\alpha]^{25}_D$ = -147.0 (*c* = 1.0, DCM).

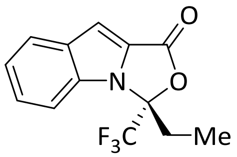

3o

3o (*R*)-3-ethyl-3-(trifluoromethyl)-1*H*,3*H*-oxazolo[3,4-a]indol-1-one

Pale yellow wax, 45.0 mg, 83% yield, 48 h; **^{1}H NMR** (400 MHz, Chloroform-*d*) δ 7.82 (d, *J* = 8.3 Hz, 1H), 7.51 – 7.42 (m, 2H), 7.35 – 7.29 (m, 1H), 7.22 (s, 1H), 2.72 – 2.40 (m, 2H), 0.66 (t, *J* = 7.3 Hz, 3H). **^{13}C NMR** (100 MHz, Chloroform-*d*) δ 158.3, 133.0, 132.9, 126.9, 124.9, 124.5, 122.8, 122.2 (q, *J* = 286.7 Hz), 111.3 (q, *J* = 2.2 Hz), 104.0, 93.1 (q, *J* = 34.1 Hz), 23.6, 4.7; **^{19}F NMR** (376 MHz, Chloroform-*d*) δ -81.73. **IR** (neat, ATR) 1790, 1572, 1558, 1366, 1226, 1162, 1078, 987, 805, 727 cm^{-1}; **HRMS** (ESI) exact mass calcd. for $C_{13}H_{11}F_3NO_2$ ($[M + H]^+$) requires m/z 270.0736, found m/z 270.0734; **HPLC** (Chiralpak OJH, *i*-propanol/hexane = 10/90, flow rate 1.0 mL/min, 25 °C, λ = 254 nm): t_R (major) = 8.5 min, t_R (minor) = 5.4 min, *ee* = 94%; $[\alpha]^{25}_D$ = -82.0 (*c* = 1.0, DCM).

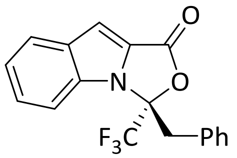

3p

3p (*R*)-3-benzyl-3-(trifluoromethyl)-1*H*,3*H*-oxazolo[3,4-a]indol-1-one

Pale yellow wax, 50.6 mg, 76% yield, 48 h; **^{1}H NMR** (400 MHz, Chloroform-*d*) δ 7.73 (d, *J* = 8.2 Hz, 1H), 7.65 (d, *J* = 8.3 Hz, 1H), 7.59 – 7.52 (m, 1H), 7.40 – 7.29 (m, 1H), 7.13 – 7.07 (m, 1H), 6.99 (t, *J* = 7.6 Hz, 2H), 6.90 (s, 1H), 6.80 – 6.75 (m, 2H), 3.78 (d, *J* = 14.5 Hz, 1H), 3.69 (d, *J* = 14.5 Hz, 1H). **^{13}C NMR** (100 MHz, Chloroform-*d*) δ 157.6, 133.2, 132.9, 130.2, 128.7, 128.4, 128.2, 126.9, 124.6, 124.6, 122.7, 122.2 (q, *J* = 286.8 Hz), 111.8 (q, *J* = 2.6 Hz), 103.8, 92.1 (q, *J* = 34.1 Hz), 36.3. **^{19}F NMR** (376 MHz, Chloroform-*d*) δ -80.73; **IR** (neat, ATR) 2971, 1788, 1379, 1287, 1218, 1069, 999, 950, 817, 728 cm^{-1}; **HRMS** (ESI) exact mass calcd. for $C_{18}H_{13}F_3NO_2$ ($[M + H]^+$) requires m/z 332.0893, found m/z 332.0891; **HPLC** (Chiralpak ADH, *i*-propanol/hexane = 2/98, flow rate 1.0 mL/min, 25 °C, λ = 254 nm): t_R (major)

= 7.4 min, t_R (minor) = 6.5 min, *ee* = 90%; $[\alpha]^{25}_D$ = -103.5 (*c* = 1.0, DCM).

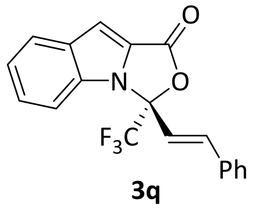

3q

3q (*R,E*)-3-styryl-3-(trifluoromethyl)-1*H*,3*H*-oxazolo[3,4-a]indol-1-one

Pale yellow oil, 50.3 mg, 73% yield, 48 h; **^{1}H NMR** (400 MHz, Chloroform-*d*) δ 7.85 (d, *J* = 8.2 Hz, 1H), 7.50 – 7.45 (m, 2H), 7.43 – 7.31 (m, 6H), 7.25 (s, 1H), 6.93 (d, *J* = 15.9 Hz, 1H), 6.68 (d, *J* = 15.9 Hz, 1H). **^{13}C NMR** (100 MHz, Chloroform-*d*) δ 157.8, 139.5, 133.7, 133.5, 133.0, 130.1, 129.0, 127.6, 127.0, 124.5, 124.3, 122.8, 122.0 (d, *J* = 287.7 Hz), 114.9, 112.2 (q, *J* = 2.2 Hz), 104.6, 91.1 (d, *J* = 35.0 Hz). **^{19}F NMR** (376 MHz, Chloroform-*d*) δ -80.74; **IR** (neat, ATR) 1797, 1558, 1441, 1342, 1292, 1175, 1125, 1070, 993, 937, 813, 741 cm^{-1}; **HRMS** (ESI) exact mass calcd. for $C_{19}H_{13}F_3NO_2$ ($[M + H]^+$) requires m/z 344.0893, found m/z 344.0899; **HPLC** (Chiralpak IE, *i*-propanol/hexane = 2/98, flow rate 1.0 mL/min, 25 ^{o}C, λ = 254 nm): t_R (major) = 7.2 min, t_R (minor) = 6.0 min, *ee* = 72%; $[\alpha]^{25}_D$ = -103.5 (*c* = 1.0, DCM).

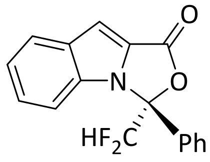

3r

3r (*R*)-3-(difluoromethyl)-3-phenyl-1*H*,3*H*-oxazolo[3,4-a]indol-1-one

Pale yellow wax, 41.3 mg, 69% yield, 48 h; **^{1}H NMR** (400 MHz, Chloroform-*d*) δ 7.83 (d, *J* = 8.2 Hz, 1H), 7.55 – 7.44 (m, 5H), 7.43 – 7.25 (m, 4H), 6.52 (t, *J* = 54.0 Hz, 1H). **^{13}C NMR** (100 MHz, Chloroform-*d*) δ 158.4, 134.2, 133.0, 131.4, 131.2, 129.4, 126.7, 126.6, 125.4, 124.2, 122.5, 112.9 (t, *J* = 252.9 Hz), 112.9 (d, *J* = 2.5 Hz), 103.8, 93.2 (dd, *J* = 28.1, 23.1 Hz). **^{19}F NMR** (376 MHz, Chloroform-*d*) δ -127.79 (d, *J* = 285.4 Hz), -133.04 (d, *J* = 285.4 Hz). **IR** (neat, ATR) 1795, 1725, 1366, 1286, 1075, 983, 911, 738 cm^{-1}; **HRMS** (ESI) exact mass calcd. for $C_{17}H_{12}F_2NO_2$ ($[M + H]^+$) requires m/z 300.0831, found m/z 300.0830; **HPLC** (Chiralpak IE, *i*-propanol/hexane = 2/98, flow rate 1.0 mL/min, 25 ^{o}C, λ = 254 nm): t_R (major) = 18.2 min, t_R (minor) = 16.7 min, *ee* = 81%; $[\alpha]^{25}_D$ = -55.7 (*c* = 1.0, DCM).

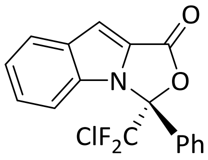

3s

3s (*R*)-3-(chlorodifluoromethyl)-3-phenyl-1*H*,3*H*-oxazolo[3,4-a]indol-1-one

Pale yellow solid, 54.5 mg, 82% yield, 96 h; ^{1}**H NMR** (400 MHz, Chloroform-*d*) δ 7.85 (d, *J* = 8.2 Hz, 1H), 7.72 (d, *J* = 7.7 Hz, 2H), 7.55 – 7.43 (m, 5H), 7.36 – 7.30 (m, 2H). ^{13}**C NMR** (100 MHz, Chloroform-*d*) δ 158.0, 134.0, 133.1, 131.3, 130.6 (t, *J* = 1.9 Hz), 129.1, 127.4 (t, *J* = 2.4 Hz), 127.0, 126.7 (t, *J* = 305.2 Hz), 125.2, 124.5, 122.8, 113.0 (t, *J* = 3.5 Hz), 104.6, 95.2 (t, *J* = 31.4 Hz). ^{19}**F NMR** (376 MHz, Chloroform-*d*) δ -60.79 (d, *J* = 176.1 Hz), -61.24 (d, *J* = 177.1 Hz); **IR** (neat, ATR) 1807, 1790, 1739, 1556, 1440, 1371, 1140, 1038, 837, 733 cm^{-1}; **HRMS** (ESI) exact mass calcd. for $C_{17}H_{11}ClF_2NO_2$ ($[M + H]^+$) requires m/z 334.0441, found m/z 334.0447; **HPLC** (Chiralpak IE, *i*-propanol/hexane = 5/95, flow rate 1.0 mL/min, 25 ^{o}C, λ = 254 nm): t_R (major) = 7.0 min, t_R (minor) = 6.4 min, *ee* = 87%; $[\alpha]^{25}_{D}$ = -85.7 (*c* = 1.0, DCM).

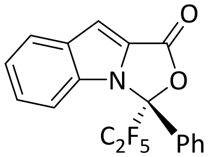

3t

3t (*R*)-3-(perfluoroethyl)-3-phenyl-1*H*,3*H*-oxazolo[3,4-a]indol-1-one

Pale yellow wax, 36.6 mg, 50% yield, 108 h; ^{1}**H NMR** (400 MHz, Chloroform-*d*) δ 7.83 (d, *J* = 8.2 Hz, 1H), 7.74 (d, *J* = 7.3 Hz, 2H), 7.61 (d, *J* = 8.5 Hz, 1H), 7.53 – 7.42 (m, 4H), 7.36 – 7.29 (m, 2H). ^{13}**C NMR** (100 MHz, Chloroform-*d*) δ 157.4, 134.4, 133.1, 131.3, 131.2, 129.0, 127.2, 126.9 (t, *J* = 2.4 Hz), 124.5, 124.0, 122.8, 118.2 (dt, *J* = 289.1, 35.0 Hz), 112.9 (t, *J* = 4.3 Hz), 112.0 (td, *J* = 267.4, 36.6 Hz), 105.0, 92.9 (t, *J* = 28.8 Hz). ^{19}**F NMR** (376 MHz, Chloroform-*d*) δ -79.19, -118.03 (d, *J* = 2.6 Hz). **IR** (neat, ATR) 1806, 1336, 1315, 1220, 1163, 1002, 919, 866, 727 cm^{-1}; **HRMS** (ESI) exact mass calcd. for $C_{18}H_{11}F_5NO_2$ ($[M + H]^+$) requires m/z 368.0704, found m/z 368.0700; **HPLC** (Chiralpak OJH, *i*-propanol/hexane = 5/95, flow rate 1.0 mL/min, 25 ^{o}C, λ = 254 nm): t_R (major) = 6.8 min, t_R (minor) = 5.9 min, *ee* = 90%; $[\alpha]^{25}_{D}$ = -61.4 (*c* = 1.0, DCM).

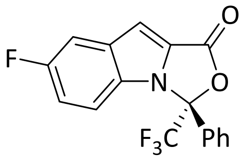

4a

4a (*R*)-7-fluoro-3-phenyl-3-(trifluoromethyl)-1*H*,3*H*-oxazolo[3,4-a]indol-1-one

Pale yellow wax, 45.6 mg, 68% yield, 48 h; ^{1}**H NMR** (400 MHz, Chloroform-*d*) δ 7.60 – 7.53 (m, 3H), 7.51 – 7.45 (m, 3H), 7.28 – 7.23 (m, 2H), 7.18 (td, *J* = 9.0, 2.5 Hz, 1H). ^{13}**C NMR** (100 MHz, Chloroform-*d*) δ 158.9 (d, *J* = 241.3 Hz), 157.5, 133.5 (d, *J* = 10.3 Hz), 131.7, 130.5, 129.7, 129.4, 126.9 (d, *J* = 2.0 Hz), 126.2, 122.2 (q, *J* = 288.2 Hz), 116.4 (d, *J* = 27.1 Hz), 113.6 (dd, *J* = 9.6, 2.5 Hz), 108.8 (d, *J* = 23.7 Hz), 104.3 (d, *J* = 5.6 Hz), 92.3 (q, *J* = 34.6 Hz); ^{19}**F NMR** (376 MHz, Chloroform-*d*) δ -77.03, -119.21; **IR** (neat, ATR) 1811, 1558, 1443, 1379, 1181, 1141, 1010, 949, 863, 808, 722 cm^{-1}; **HRMS** (ESI) exact mass calcd. for $C_{17}H_{10}F_4NO_2$ ($[M + H]^+$) requires m/z 336.0642, found m/z 336.0643; **HPLC** (Chiralpak IE, *i*-propanol/hexane = 2/98, flow rate 1.0 mL/min, 25 ^{o}C, λ = 254 nm): t_R (major) = 6.9 min, t_R (minor) = 6.3 min, *ee* = 84%; $[\alpha]^{25}_{D}$ = -100.9 (*c* = 1.0, DCM).

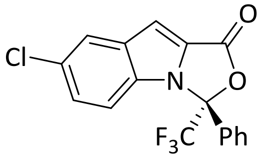

4b

4b (*R*)-7-chloro-3-phenyl-3-(trifluoromethyl)-1*H*,3*H*-oxazolo[3,4-a]indol-1-one

Pale yellow wax, 47.8 mg, 68% yield, 48 h; ^{1}**H NMR** (400 MHz, Chloroform-*d*) δ 7.82 (d, *J* = 2.1 Hz, 1H), 7.60 – 7.50 (m, 3H), 7.52 – 7.43 (m, 2H), 7.37 (dd, *J* = 9.0, 2.0 Hz, 1H), 7.24 (d, *J* = 8.1 Hz, 2H). ^{13}**C NMR** (100 MHz, Chloroform-*d*) δ 157.4, 133.9, 132.1, 131.7, 129.6, 129.4, 128.8, 127.7, 126.9 (d, *J* = 2.2 Hz), 125.9, 123.6, 121.1 (q, *J* = 288.2 Hz), 113.5 (d, *J* = 2.6 Hz), 103.9, 92.3 (q, *J* = 34.9 Hz). ^{19}**F NMR** (376 MHz, Chloroform-*d*) δ -77.04; **IR** (neat, ATR) 1808, 1552, 1454, 1433, 1377, 1273, 1181, 1141, 1065, 1011, 950, 873, 177, 723 cm^{-1}; **HRMS** (ESI) exact mass calcd. for $C_{17}H_{10}ClF_3NO_2$ ($[M + H]^+$) requires m/z 352.0347, found m/z 352.0345; **HPLC** (Chiralpak IE, *i*-propanol/hexane = 5/95, flow rate 1.0 mL/min, 25 ^{o}C, λ = 254 nm): t_R (major) = 5.8 min, t_R (minor) = 5.4 min, *ee* = 80%; $[\alpha]^{25}_{D}$ = -50.7 (*c* = 1.0, DCM).

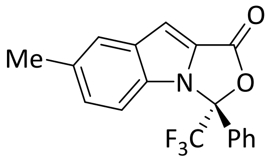

4c

4c (*R*)-7-methyl-3-phenyl-3-(trifluoromethyl)-1*H*,3*H*-oxazolo[3,4-a]indol-1-one

Pale yellow wax, 55.4 mg, 84% yield, 48 h; **^{1}H NMR** (400 MHz, Chloroform-*d*) δ 7.67 – 7.63 (m, 3H), 7.59 – 7.53 (m, 1H), 7.52 – 7.47 (m, 2H), 7.28 – 7.24 (m, 3H), 2.51 (s, 3H). **^{13}C NMR** (100 MHz, Chloroform-*d*) δ 158.0, 133.4, 132.5, 132.4, 131.4, 130.2, 129.2, 129.0, 127.0 (q, *J* = 2.1 Hz), 124.7, 123.6, 122.3 (q, *J* = 288.2 Hz), 112.1 (d, *J* = 2.3 Hz), 104.1, 92.2 (q, *J* = 34.4 Hz), 21.4. **^{19}F NMR** (376 MHz, Chloroform-*d*) δ -77.16; **IR** (neat, ATR) 1805, 1739, 1557, 1455, 1363, 1190, 1083, 997, 948, 883, 797, 727 cm^{-1}; **HRMS** (ESI) exact mass calcd. for $C_{18}H_{13}F_3NO_2$ ([M + H]$^+$) requires m/z 332.0893, found m/z 332.0895; **HPLC** (Chiralpak IE, *i*-propanol/hexane = 5/95, flow rate 1.0 mL/min, 25 ^{o}C, λ = 254 nm): t_R (major) = 6.6 min, t_R (minor) = 6.0 min, *ee* = 90%; $[\alpha]^{25}_{D}$ = -52.9 (*c* = 1.0, DCM).

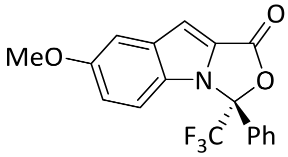

4d

4d (*R*)-7-methoxy-3-phenyl-3-(trifluoromethyl)-1*H*,3*H*-oxazolo[3,4-a]indol-1-one

Pale yellow wax, 62.4 mg, 90% yield, 36 h; **^{1}H NMR** (400 MHz, Chloroform-*d*) δ 7.61 (d, *J* = 8.3 Hz, 2H), 7.56 – 7.49 (m, 1H), 7.51 – 7.42 (m, 2H), 7.27 – 7.18 (m, 3H), 7.09 (dd, *J* = 9.3, 2.5 Hz, 1H), 3.87 (s, 3H). **^{13}C NMR** (100 MHz, Chloroform-*d*) δ 157.9, 155.9, 133.8, 131.5, 130.1, 129.3, 129.2, 127.0 (q, *J* = 2.0 Hz), 125.0, 122.3 (q, *J* = 288.2 Hz), 118.9, 113.3 (q, *J* = 2.3 Hz), 104.0, 103.9, 92.2 (q, *J* = 34.5 Hz), 55.7. **^{19}F NMR** (376 MHz, Chloroform-*d*) δ -77.22; **IR** (neat, ATR) 1790, 1556, 1540, 1376, 1344, 1259, 1178, 1015, 951, 842, 796, 721 cm^{-1}; **HRMS** (ESI) exact mass calcd. for $C_{18}H_{13}F_3NO_3$ ([M + H]$^+$) requires m/z 348.0842, found m/z 348.0845; **HPLC** (Chiralpak IE, *i*-propanol/hexane = 5/95, flow rate 1.0 mL/min, 25 ^{o}C, λ = 254 nm): t_R (major) = 8.1 min, t_R (minor) = 7.3 min, *ee* = 90%; $[\alpha]^{25}_{D}$ = -86.7 (*c* = 1.0, DCM).

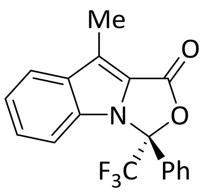

4e

4e (R)-9-methyl-3-phenyl-3-(trifluoromethyl)-1H,3H-oxazolo[3,4-a]indol-1-one

Colorless wax, 48.9 mg, 74% yield, 72 h; ^{1}**H NMR** (400 MHz, Chloroform-d) δ 7.83 – 7.76 (m, 1H), 7.63 (d, J = 7.7 Hz, 2H), 7.55 – 7.38 (m, 4H), 7.35 – 7.27 (m, 2H), 2.64 (s, 3H). ^{13}**C NMR** (100 MHz, Chloroform-d) δ 158.4, 133.6, 133.6, 131.3, 130.5, 129.2, 127.1, 127.0 (q, J = 2.0 Hz), 122.4, 122.4 (q, J = 288.4 Hz), 122.0, 121.3, 117.4, 112.4 (q, J = 2.3 Hz), 91.9 (q, J = 34.4 Hz), 8.6. ^{19}**F NMR** (376 MHz, Chloroform-d) δ -77.25; **IR** (neat, ATR) 1789, 1602, 1442, 1329, 1178, 1121, 1054, 981, 948, 898, 720 cm^{-1}; **HRMS** (ESI) exact mass calcd. for $C_{18}H_{13}F_{3}NO_{2}$ ([M + H]$^{+}$) requires m/z 332.0893, found m/z 332.0898; **HPLC** (Chiralpak IE, i-propanol/hexane = 2/98, flow rate 1.0 mL/min, 25 °C, λ = 254 nm): t_R (major) = 6.7 min, t_R (minor) = 6.0 min, ee = 90%; $[\alpha]^{25}_{D}$ = -102.0 (c = 1.0, DCM).

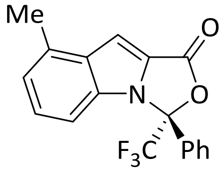

4f

4f (R)-8-methyl-3-phenyl-3-(trifluoromethyl)-1H,3H-oxazolo[3,4-a]indol-1-one

Colorless wax, 49.7 mg, 75% yield, 48 h; ^{1}**H NMR** (400 MHz, Chloroform-d) δ 7.62 (d, J = 8.0 Hz, 2H), 7.57 – 7.49 (m, 1H), 7.51 – 7.43 (m, 2H), 7.38 – 7.28 (m, 2H), 7.16 (d, J = 8.6 Hz, 1H), 7.11 (dt, J = 7.1, 1.0 Hz, 1H), 2.64 (s, 3H). ^{13}**C NMR** (100 MHz, Chloroform-d) δ 158.0, 134.2, 133.8, 133.3, 131.5, 130.1, 129.2, 127.2, 127.0 (d, J = 1.9 Hz), 124.1, 122.8, 122.3 (q, J = 288.2 Hz), 110.0 (q, J = 2.4 Hz), 103.2, 92.3 (q, J = 34.4 Hz), 18.7. ^{19}**F NMR** (376 MHz, Chloroform-d) δ -76.99; **IR** (neat, ATR) 1790, 1558, 1453, 1381, 1345, 1241, 1171, 1137, 1050, 947, 901, 777, 722 cm^{-1}; **HRMS** (ESI) exact mass calcd. for $C_{18}H_{13}F_{3}NO_{2}$ ([M + H]$^{+}$) requires m/z 332.0893, found m/z 332.0894; **HPLC** (Chiralpak IE, i-propanol/hexane = 5/95, flow rate 1.0 mL/min, 25 °C, λ = 254 nm): t_R (major) = 6.4 min, t_R (minor) = 6.0 min, ee = 92%; $[\alpha]^{25}_{D}$ = -46.1 (c = 1.0, DCM).

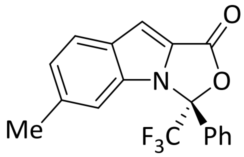

4g

4g (*R*)-6-methyl-3-phenyl-3-(trifluoromethyl)-1*H*,3*H*-oxazolo[3,4-a]indol-1-one

Colorless wax, 52.5 mg, 79% yield, 36 h; **^{1}H NMR** (400 MHz, Chloroform-*d*) δ 7.74 (d, *J* = 8.4 Hz, 1H), 7.65 (dd, *J* = 7.8, 1.6 Hz, 2H), 7.61 – 7.54 (m, 1H), 7.51 (dd, *J* = 8.3, 6.5 Hz, 2H), 7.30 (s, 1H), 7.21 – 7.11 (m, 2H), 2.49 (s, 3H). **^{13}C NMR** (100 MHz, Chloroform-*d*) δ 158.0, 137.7, 134.4, 131.5, 131.0, 130.1, 129.3, 127.1 (q, *J* = 2.1 Hz), 125.0, 124.1, 124.0, 122.3 (q, *J* = 288.3 Hz), 112.0 (q, *J* = 2.3 Hz), 104.7, 92.2 (q, *J* = 34.1 Hz), 22.2. **^{19}F NMR** (376 MHz, Chloroform-*d*) δ -76.89; **IR** (neat, ATR) 1808, 1622, 1553, 1453, 1373, 1336, 1180, 1073, 1012, 948, 892, 826, 722 cm^{-1}; **HRMS** (ESI) exact mass calcd. for $C_{18}H_{13}F_3NO_2$ ($[M + H]^+$) requires m/z 332.0893, found m/z 332.0896; **HPLC** (Chiralpak IE, *i*-propanol/hexane = 5/95, flow rate 1.0 mL/min, 25 °C, λ = 254 nm): t_R (major) = 6.5 min, t_R (minor) = 6.0 min, *ee* = 90%; $[\alpha]^{25}_D$ = -162.1 (*c* = 1.0, DCM).

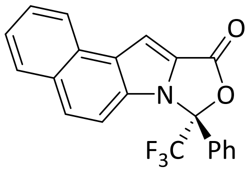

4h

4h (*R*)-8-phenyl-8-(trifluoromethyl)-8*H*,10*H*-benzo[e]oxazolo[3,4-a]indol-10-one

Pale yellow wax, 30.8 mg, 42% yield, 48 h; **^{1}H NMR** (400 MHz, Chloroform-*d*) δ 8.31 (d, *J* = 8.1 Hz, 1H), 7.93 (d, *J* = 8.1 Hz, 1H), 7.83 (s, 1H), 7.78 (d, *J* = 9.2 Hz, 1H), 7.74 – 7.65 (m, 1H), 7.65 – 7.58 (m, 2H), 7.61 – 7.51 (m, 2H), 7.48 (dd, *J* = 8.4, 6.6 Hz, 2H), 7.35 (d, *J* = 9.1 Hz, 1H). **^{13}C NMR** (100 MHz, Chloroform-*d*) δ 157.6, 131.6, 130.0, 129.7, 129.4, 129.3, 129.0, 129.0, 128.9, 127.7, 127.0 (q, *J* = 2.0 Hz), 125.8, 123.1, 122.9, 122.2 (q, *J* = 287.8 Hz), 112.3 (q, *J* = 2.3 Hz), 103.5, 92.3 (d, *J* = 34.5 Hz), miss one carbon. **^{19}F NMR** (376 MHz, Chloroform-*d*) δ -76.95; **IR** (neat, ATR) 2964, 1739, 1542, 1445, 1365, 1260, 1016, 949, 798, 720 cm^{-1}; **HRMS** (ESI) exact mass calcd. for $C_{21}H_{13}F_3NO_2$ ($[M + H]^+$) requires m/z 368.0893, found m/z 368.0893; **HPLC** (Chiralpak IE, *i*-propanol/hexane = 5/95, flow rate 1.0 mL/min, 25 °C, λ = 254 nm): t_R (major) = 9.2 min, t_R (minor) = 8.3 min, *ee* = 69%; $[\alpha]^{25}_D$ = -92.1 (*c* = 1.0, DCM).

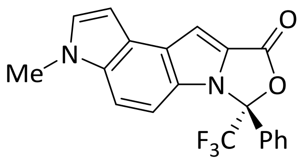

4i

4i (*R*)-3-methyl-7-phenyl-7-(trifluoromethyl)-7*H*-oxazolo[3,4-a]pyrrolo[3,2-e]indol-9(3*H*)-one White solid, 32.4 mg, 42% yield, 48 h; ^{1}**H NMR** (400 MHz, Chloroform-*d*) δ 7.62 (d, *J* = 8.0 Hz, 2H), 7.57 – 7.49 (m, 2H), 7.48 – 7.40 (m, 3H), 7.18 (d, *J* = 3.0 Hz, 1H), 7.12 – 7.06 (m, 1H), 6.82 (d, *J* = 2.5 Hz, 1H), 3.88 (s, 3H). ^{13}**C NMR** (100 MHz, Chloroform-*d*) δ 158.1, 131.8, 131.3, 130.4, 129.9, 129.1, 128.7, 127.2 (q, *J* = 2.0 Hz), 126.6, 123.0, 122.4 (d, *J* = 288.4 Hz), 121.9, 111.3, 106.3 (q, *J* = 2.6 Hz), 102.9, 100.1, 92.2 (d, *J* = 34.3 Hz), 33.4. ^{19}**F NMR** (376 MHz, Chloroform-*d*) δ -76.67; **IR** (neat, ATR) 1789, 1556, 1496, 1369, 1241, 1187, 1059, 1021, 952, 823, 738 cm^{-1}; **HRMS** (ESI) exact mass calcd. for $C_{20}H_{14}F_3N_2O_2$ ($[M + H]^+$) requires m/z 371.1002, found m/z 371.1000; **HPLC** (Chiralpak IE, *i*-propanol/hexane = 5/95, flow rate 1.0 mL/min, 25 ^{o}C, λ = 254 nm): t_R (major) = 22.5 min, t_R (minor) = 19.8 min, *ee* = 85%; $[\alpha]^{25}_D$ = -78.1 (*c* = 1.0, DCM).

Gram Scale Synthesis Procedure

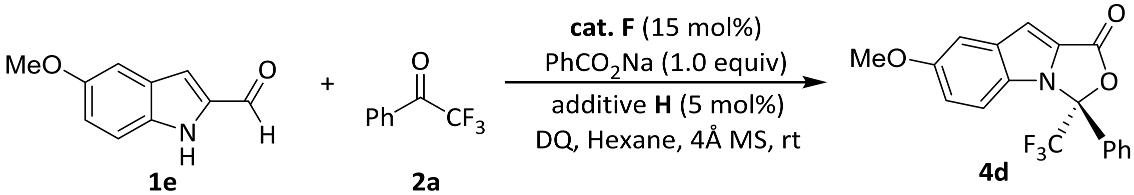

To a flame-dried Schlenk reaction tube equipped with a magnetic stir bar, was added the precatalyst **F** (308.4 mg, 0.60 mmol), DQ (1.80 g, 4.4 mmol), additive **H** (100.0 mg, 0.20 mmol), $PhCO_2Na$ (576.0 mg, 4.0 mmol), **1e** (700.8 mg, 4.0 mmol) and 4Å M.S. (600 mg). The Schlenk tube was closed with a septum, evacuated and refilled with argon atmosphere. Hexane (33.4 mL), DCM (6.6 mL) and **2a** (835.2 mg, 4.8 mmol) was added. The mixture was then stirred at 25 ℃ and monitored by TLC until **1e** was consumed. The mixture was concentrated under reduced pressure and purified by column chromatography on silica gel (hexane/EtOAc = 100:1) to afford the desired product **4d** as a pale yellow wax, 1.29 g, 92% yield, 90% ee.

Synthetic Transformation

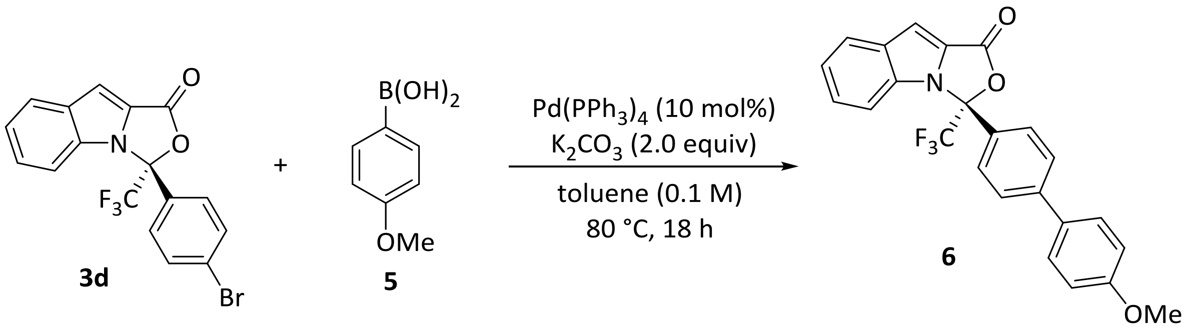

To a flame-dried Schlenk reaction tube equipped with a magnetic stir bar, was added **3d** (19.8 mg, 0.05 mmol), (4-methoxyphenyl)boronic acid **5** (15.2 mg, 0.10 mmol), K_2CO_3 (13.8 mg, 0.10 mmol), and $Pd(PPh_3)_4$ (5.7 mg, 0.005 mmol). The Schlenk tube was closed with a septum, evacuated and refilled with argon atmosphere. Toluene (0.5 mL) was added then the mixture was then stirred at 80 ℃ for 18 h. The mixture was concentrated under reduced pressure and purified by column chromatography on silica gel (hexane/EtOAc = 100:1) to afford the desired product (*R*)-3-(4'-methoxy-[1,1'-biphenyl]-4-yl)-3-(trifluoromethyl)-1*H*,3*H*-oxazolo[3,4-a]indol-1-one **6** as a yellow wax, 15.1 mg, 71% yield; **^{1}H NMR** (400 MHz, Chloroform-*d*) δ 7.86 (d, *J* = 8.3 Hz, 1H), 7.64 (s, 4H), 7.56 – 7.50 (m, 2H), 7.48 – 7.41 (m, 1H), 7.40 – 7.30 (m, 3H), 7.03 – 6.95 (m, 2H), 3.86 (s, 3H). **^{13}C NMR** (100 MHz, Chloroform-*d*) δ 159.9, 157.9, 143.9, 133.9, 133.1, 131.8, 128.3, 127.9, 127.5 (q, *J* = 1.9 Hz), 127.3, 127.0, 124.8, 124.4, 122.8, 122.3 (d, *J* = 288.2 Hz), 114.5, 112.6 (q, *J* = 2.4 Hz), 104.6, 92.4 (q, *J* = 34.7 Hz), 55.4.**^{19}F NMR** (376 MHz, Chloroform-*d*) δ -77.00; **HRMS** (ESI) exact mass calcd. for $C_{24}H_{17}F_3NO_3$ ($[M + H]^+$) requires m/z 424.1155, found m/z 424.1157; **HPLC** (Chiralpak IE, *i*-propanol/hexane = 5/95, flow rate 1.0 mL/min, 25 °C, λ = 254 nm): t_R (major) = 13.3 min, t_R (minor) = 12.4 min, *ee* = 92%; $[\alpha]^{25}_D$ = -94.5 (*c* = 1.0, DCM).

HRMS of II

Supplementary Table 1 HRMS of **II**

Mass	Calc. Mass	mDa	PPM	DBE	i-FIT	Norm	Conf(%)	Formula
568.1049	568.1044	0.5	0.9	20.5	678.1	2.027	13.18	$C_{27}H_{15}N_5O_4F_5$

Kinetic Experiment

Determination of the reaction order

To simply the reaction conditions and improve the reaction robustness, we didn't add additive and change solvent to DCM-d_2. A Young style NMR tube with a rubber valve was added precatalyst **F**, DQ (22.4 mg, 0.06 mmol), $PhCO_2Na$ (7.3 mg, 0.05 mmol), **1a** (0.05 mmol) and 4Å MS (20 mg) under argon. DCM-d_2 (0.5 mL) and **2a** (0.06 mmol) was added by injection and the NMR tube was subjected to a 400 MHz NMR spectrometer at 25 ℃. ^{1}H-NMR and ^{19}F-NMR spectrum were then taken alternately about every 120s in initial 0.5 h. The rate order of precatalyst **F** was evaluated by varying the starting concentration of precatalyst **F** between 5 and 25 mol%. Using the differential rate laws demonstrated first-order dependence with the catalyst precatalyst **F**. And with the same method varying the equiv. of substrates **1a**, **2a** and DQ respectively, a zero-order dependence on substrates could be deduced.

Determination of precatalyst **F** rate order

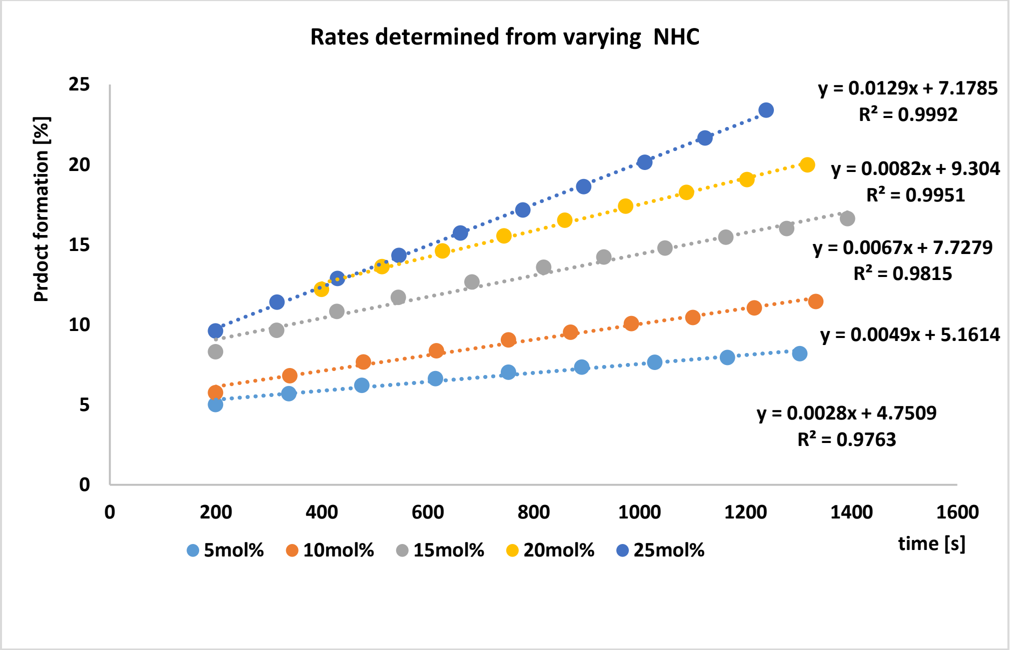

Supplementary Figure 154. Rates determined from varying [precatalyst **F**]

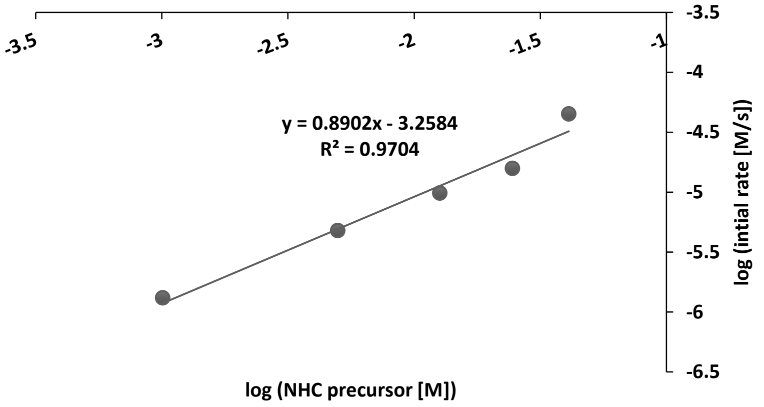

NHC-precursor **F** [M]	Log(NHC-precursor **F**)	Initial rate [M/s]	Log(Initial rate [M/s])
5.0 x 10^{-2}	-3.00	2.8 x 10^{-3}	-5.88
10.0 x 10^{-2}	-2.30	4.9 x 10^{-3}	-5.32
15.0 x 10^{-2}	-1.90	6.7 x 10^{-3}	-5.01
20.0 x 10^{-2}	-1.61	8.2 x 10^{-3}	-4.80
25.0 x 10^{-2}	-1.39	12.9 x 10^{-3}	-4.35

Supplementary Figure 155. Plot log(initial rate) vs. log(concentration [precatalyst **F**])

Determination of **1a** rate order

The rate order of **1a** was evaluated following the above procedure, varying the starting concentration of **1a** between 0.5 and 3.0 equiv. The quantitative results and graphs of these experiments are provided below. Using the differential rate laws demonstrated zero-order dependence with **1a**.

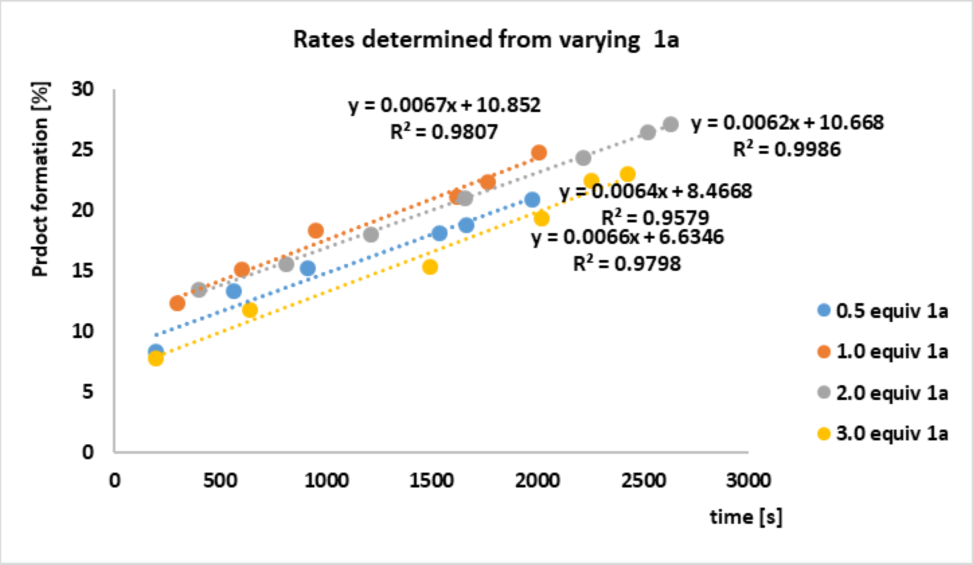

Supplementary Figure 156. Rates determined from varying [**1a**]

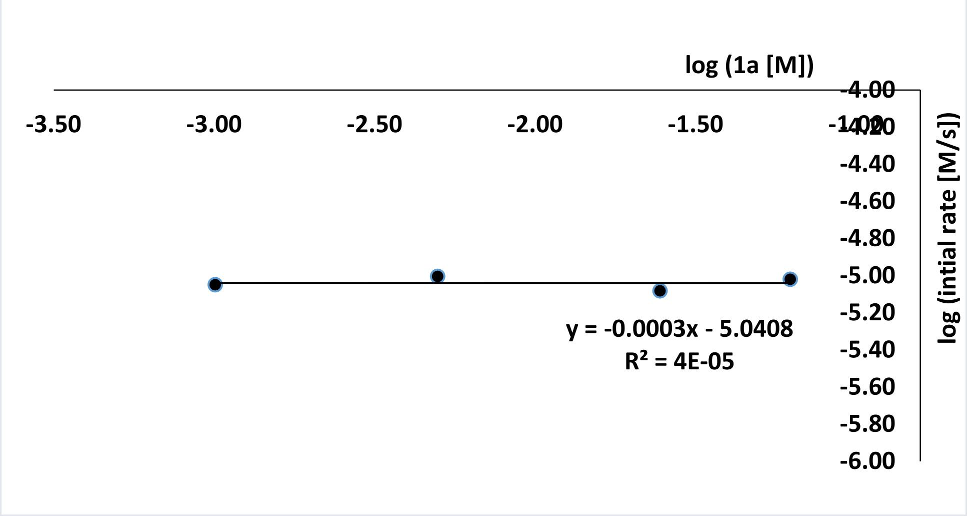

**1a** [M]	Log(**1a**)	Initial rate [M/s]	Log(Initial rate [M/s])
0.05	-3.00	6.4 x 10^{-3}	-5.05
0.1	-2.30	6.7 x 10^{-3}	-5.01
0.2	-1.61	6.2 x 10^{-3}	-5.08
0.3	-1.20	6.6 x 10^{-3}	-5.02

Supplementary Figure 157. Plot log(initial rate) vs. log(concentration [**1a**])

Determination of **2a** rate order

The rate order of **2a** was evaluated following the above procedure, varying the starting concentration of **2a** between 0.6 and 3.6 equiv. The quantitative results and graphs of these experiments are provided below. Using the differential rate laws demonstrated zero-order dependence with **2a**.

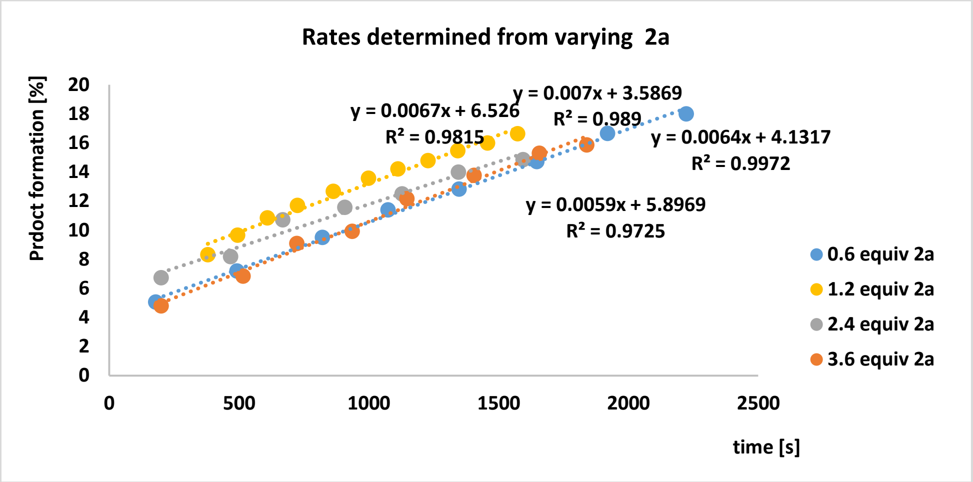

Supplementary Figure 158. Rates determined from varying [**2a**]

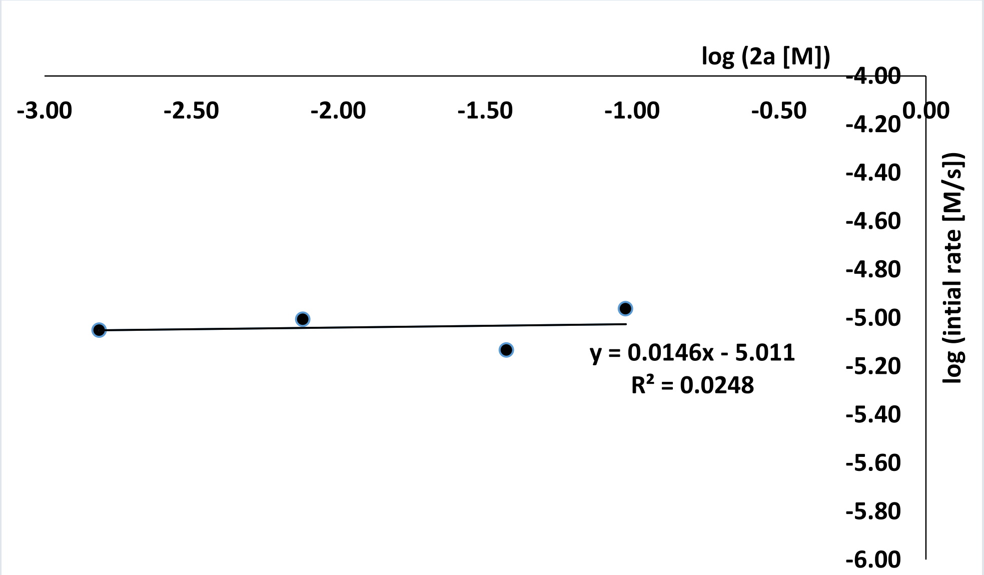

**2a** [M]	Log(**2a**)	Initial rate [M/s]	Log(Initial rate [M/s])
0.06	-2.81	6.4×10^{-3}	-5.05
0.12	-2.12	6.7×10^{-3}	-5.01
0.24	-1.43	5.9×10^{-3}	-5.13
0.36	-1.02	7.0×10^{-3}	-4.96

Supplementary Figure 159. Plot log(initial rate) vs. log(concentration [**2a**])

Determination of DQ rate order

The rate order of DQ was evaluated following the above procedure, varying the starting concentration of DQ between 0.55 and 3.3 equiv. The quantitative results and graphs of these experiments are provided below. Using the differential rate laws demonstrated zero-order dependence with DQ.

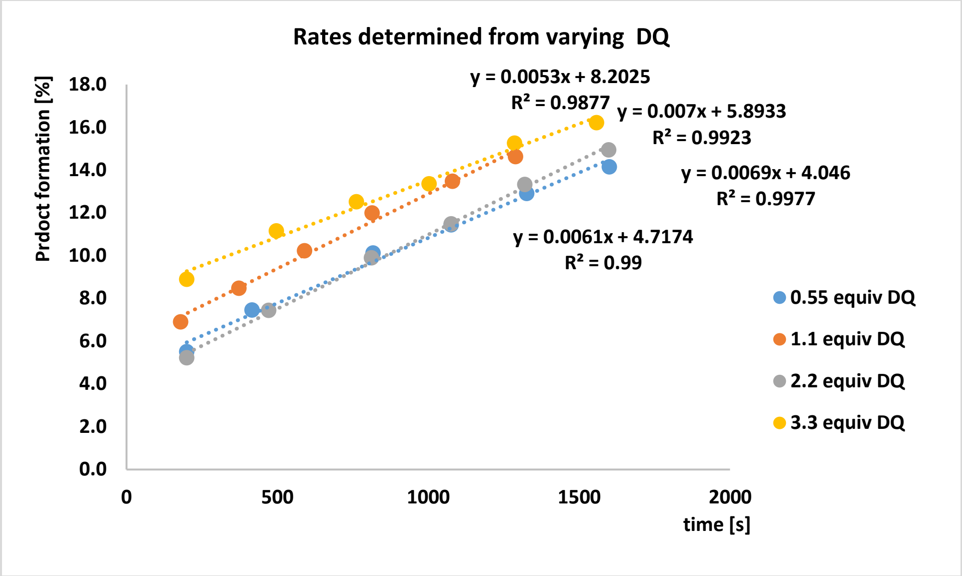

Supplementary Figure 160. Rates determined from varying [DQ]

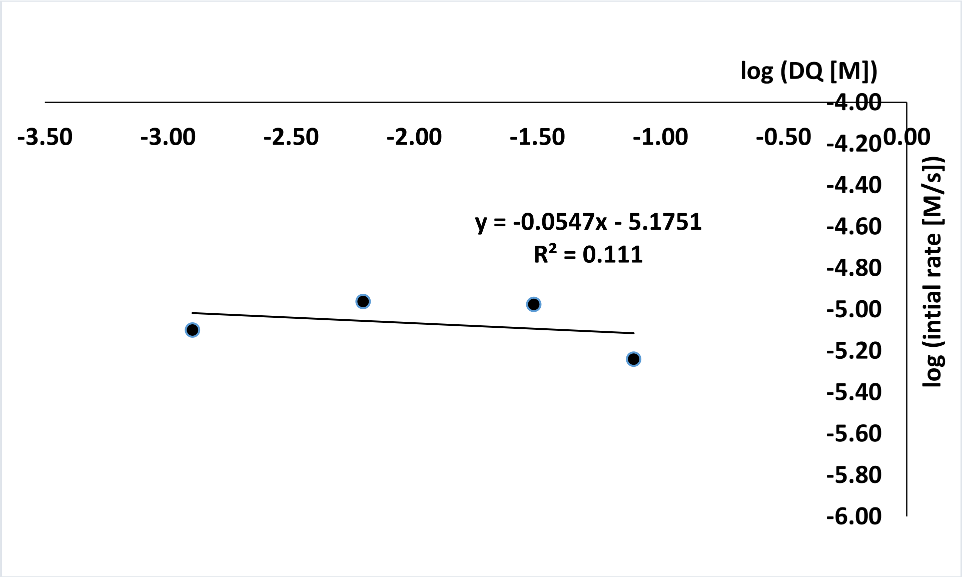

DQ [M]	Log(DQ)	Initial rate [M/s]	Log(Initial rate [M/s])
0.055	-2.90	6.1×10^{-3}	-5.10
0.11	-2.21	7.0×10^{-3}	-4.96
0.22	-1.51	6.9×10^{-3}	-4.98
0.33	-1.11	5.3×10^{-3}	-5.24

Supplementary Figure 161. Plot log(initial rate) vs. log(concentration [DQ])

Determination of the reaction rate related the electron property of indole and aryl ketone

To a flame-dried Schlenk reaction tube equipped with a magnetic stir bar, was added the precatalyst **F** (15.4 mg, 0.03 mmol), DQ (90.0 mg, 0.22 mmol), additive **H** (5.0 mg, 0.01 mmol),

$PhCO_2Na$ (28.8 mg, 0.20 mmol), **1** (0.20 mmol) and 4Å MS (60 mg). The Schlenk tube was closed with a septum, evacuated and refilled with argon atmosphere. Hexane (2.0 mL) and **2** (0.24 mmol) was added. The mixture was then stirred at 25 ℃. Yield was determined by ^{19}F NMR based on the use of benzotrifluoride as internal standard or ^{1}H NMR based on the use of TMS as internal standard. And the result showed that the electron-rich indole gave more favorable outcomes (a and b) and the electronic-deficient aryl ketone reacted faster (b, c and d).

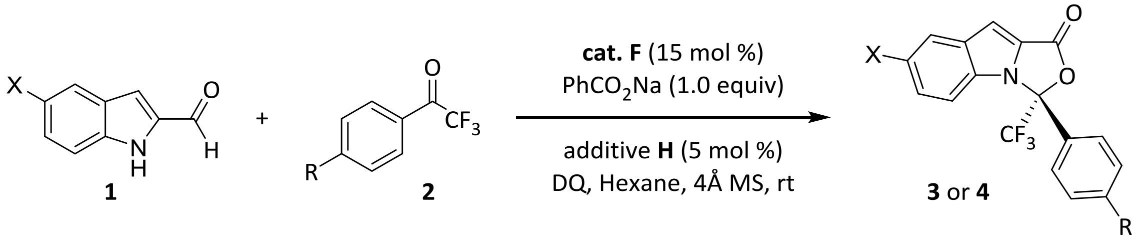

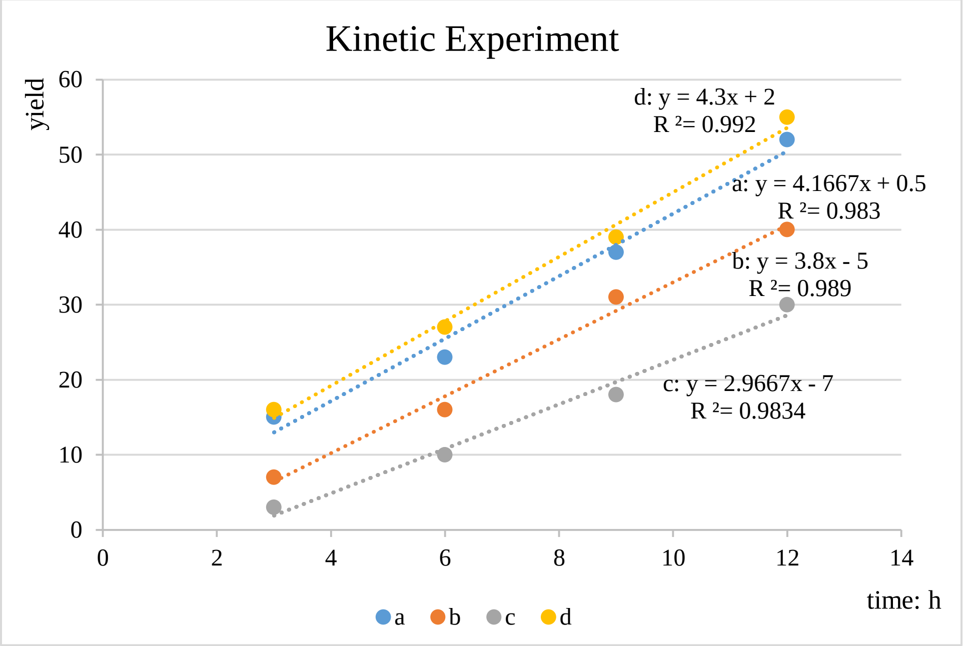

a: X = Me, R = H; b: X = H, R = H; c: X = H, R = Me; d: X = H, R = Cl.

Supplementary Figure 162. Determination of the reaction rate of the electron property of indole and aryl ketone

Control Experiment

When the reaction of **1a** and **2a** (Supplementary Figure 163a) was performed under the standard conditions without the pre-catalyst **F**, the desired product **3a** or acetal was not formed neither indicating the role of NHC in this [10+2] annulation and the direct N-H addition of initially formed acylazolium then cyclization in a [10+2] pathway is impossible in this case. Further, when the reaction was performed without DQ (Supplementary Figure 163b), the product **3u** was not formed signifying the conjugated pi-electron is indispensable. Additionally, when used N-Me protected aldehyde **1k** (Supplementary Figure 163c), the target product **3v** cannot be detected implying the aza-benzofulvene intermediate's activated site *Nitrogen* cannot be displaced by *Carbon*.

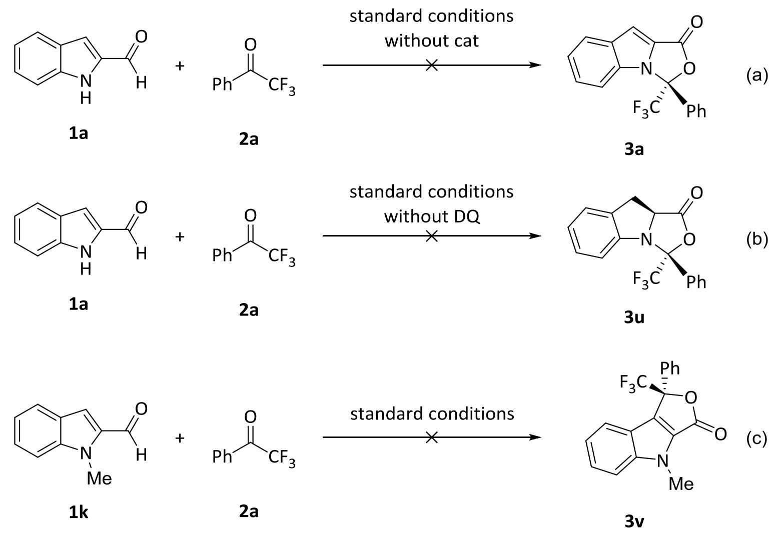

Supplementary Figure 163 Control Experiment

Crystal Structure of 3e

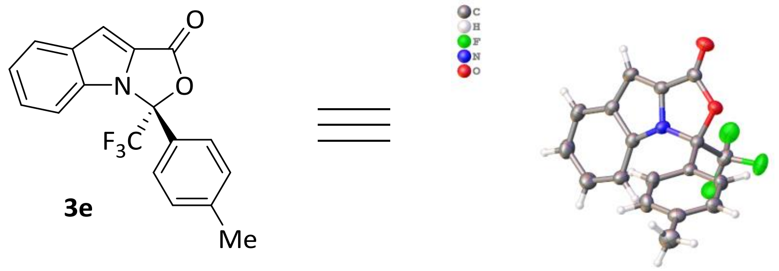

Supplementary Figure 164. Crystal Structure of **3e**

Supplementary Table 2 Crystal Structure of **3e**

Identification code	20190718pengqiupeng-2
Empirical formula	$C_{18}H_{12}F_3NO_2$
Formula weight	331.29
Temperature/K	173.00(10)
Crystal system	orthorhombic
Space group	$P2_12_12_1$
a/Å	10.0743(3)
b/Å	11.6391(2)
c/Å	12.9583(3)
α/°	90
β/°	90
γ/°	90
Volume/Å^3	1519.44(6)
Z	4
ρ_{calc}g/cm^3	1.448
μ/mm^{-1}	0.119
F(000)	680.0
Crystal size/mm^3	0.4 × 0.35 × 0.3
Radiation	MoKα (λ = 0.71073)
2Θ range for data collection/°	7.002 to 60.87
Index ranges	$-13 \leq h \leq 14$, $-16 \leq k \leq 16$, $-17 \leq l \leq 18$
Reflections collected	28355
Independent reflections	4274 [R_{int} = 0.0315, R_{sigma} = 0.0214]
Data/restraints/parameters	4274/0/218
Goodness-of-fit on F^2	1.034
Final R indexes [I>=2σ (I)]	R_1 = 0.0358, wR_2 = 0.0799
Final R indexes [all data]	R_1 = 0.0449, wR_2 = 0.0846
Largest diff. peak/hole / e Å^{-3}	0.22/-0.24
Flack parameter	0.02(17)

Computational Details

All the theoretical calculations in the study were performed using Gaussian16 program package[7]. All the geometries were optimized at the M062X[8]/6-31G(d, p) level, and the solvent effect was utilized the polarizable continuum model using integral equation formalism model (IEFPCM) in hexane solvent.[9] And the harmonic vibrational frequency calculations were performed at the same level to confirm the local minima and transition state. In addition, intrinsic reaction coordinate (IRC) computations with Hessian-based predictor−corrector integrator (HPC)[10-12] have been used to test some reaction steps to confirm the located transition states as residing on the correct reaction coordinates.

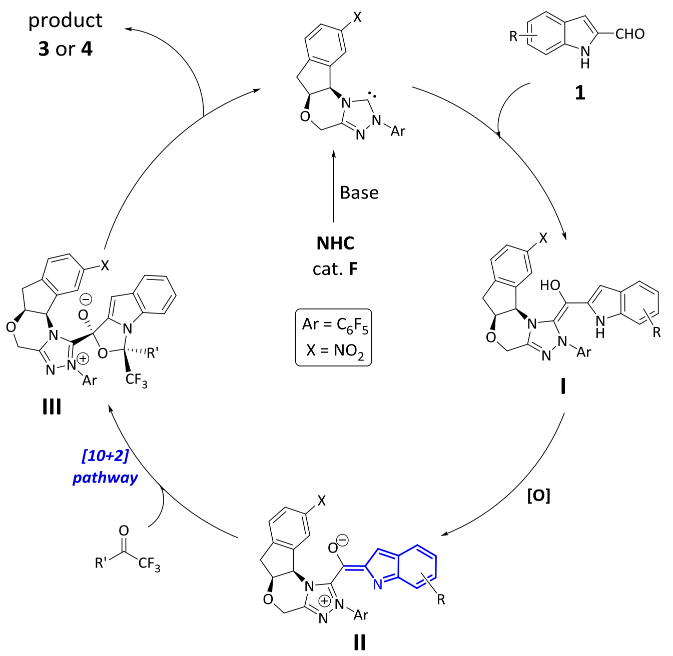

Supplementary Figure 165. The Gibbs free energy barriers and distortion energy in the process. (kcal/mol)

Supplementary Table 3 The free-energy for the corresponding structures

Species	E(au)	Species	E(au)
2	-476.849491	II	-2111.045501
TS(II-III)R	-2792.1352	TS(II-III)S	-2792.127130

Supplementary Table 4 The distortion energy for the TS(II-III)

Species	E(au)	Species	E(au)
2	-682.3617644	II	-2110.191031
TS(II-III)R	-2792.567189	TS(II-III)S	-2792.558481
2 in TS(II-III)R	-682.3453045	II in TS(II-III)R	-2110.16726
2 in TS(II-III)R	-682.3377994	II in TS(II-III)R	-2110.171377

Supplementary Table 5 The free-energy for the corresponding transition states by using different methods and basis sets.

Method/Basis set	TS(II-III)R(au)	TS(II-III)R(au)	ΔΔG‡ (kcal/mol)
**B3LYP-D3/6-31g(d,p)**	-2793.243032	-2793.237577	3.4
**M11L/6-31 g(d,p)**	-2792.32462	-2792.319474	3.2
**Wb97xd**	-2792.315445	-2792.311206	2.7
**M062X/def2-TZVP[a]**	-2793.653391	-2793.661387	4.5
**M062X/6-311G(d,p)[a]**	-2793.351839	-2793.343334	4.8

a: The single point energy at the level+ ZPVE of M062X/6-31G(d,p).

The IRC of transition state TS(II)R

In order to find out whether the process from II to III would be concerted, the IRC (intrinsic reaction coordinate) calculation of transition state TS(II)R has been performed. **Supplementary Figure 166** clearly shows the C1 of trifluoroacetophenone and N1 of indole would form the covalent bond firstly. Along with the decreasing distance of C1-N1, the bond of oxgen atom O1−C2 gradually formed unitl the intermedaite III generate. Hence, we spectulate that the process tends to be a concerted asynchronous process.

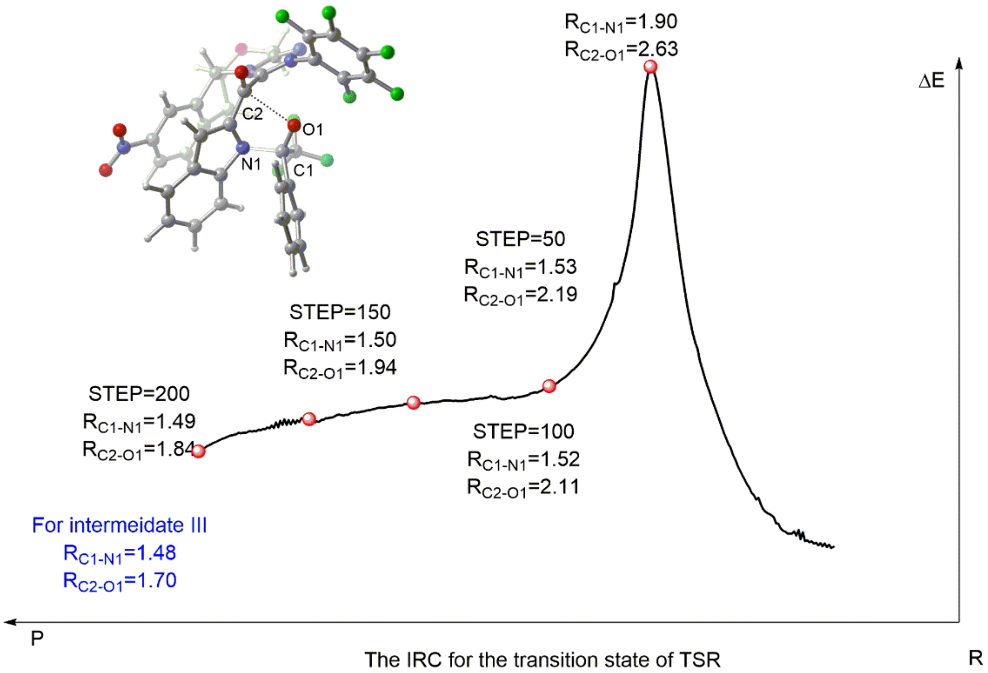

Supplementary Figure 166. IRC calculation of transition state TS(II)R

Supplementary References

1 Khodakovskiy, P. V. *et al.* 2-(Trifluoroacetyl) imidazoles, 2-Trifluoroacetyl-1, 3-thiazoles, and 2-Trifluoroacetyl-1, 3-oxazoles. *Synthesis* **2008**, 948-956 (2008).

2 Bos, M. *et al.* Catalytic Enantioselective Synthesis of Highly Functionalized Difluoromethylated Cyclopropanes. *Angew. Chem. Int. Ed.* **56**, 13319-13323 (2017).

3 Enders, D., Wang, C. & Raabe, G. Enantioselective synthesis of 3H-pyrrolo [1, 2-a] indole-2-carbaldehydes via an organocatalytic domino aza-Michael/aldol condensation reaction. *Synthesis* **2009**, 4119-4124 (2009).

4 Hong, L., Sun, W., Liu, C., Wang, L. & Wang, R. Asymmetric Organocatalytic N□ Alkylation of Indole□2□carbaldehydes with α, β□Unsaturated Aldehydes: One□Pot Synthesis of Chiral Pyrrolo [1, 2□a] indole□2□carbaldehydes. *Chem. Eur. J.* **16**, 440-444 (2010).

5 Bonnamour, J. & Bolm, C. Iron (II) triflate as a catalyst for the synthesis of indoles by intramolecular C– H amination. *Org. Lett.* **13**, 2012-2014 (2011).

6 Kuwano, S. *et al.* Enhanced rate and selectivity by carboxylate salt as a basic cocatalyst in chiral N-Heterocyclic carbene-catalyzed asymmetric acylation of secondary alcohols. *J. Am. Chem. Soc.* **135**, 11485-11488 (2013).

7 Frisch, M. J.; Trucks, G. W.; Schlegel, H. B.; Scuseria, G. E.; Robb, M. A.; Cheeseman, J. R.; Scalmani, G.; Barone, V.; Petersson, G. A.; Nakatsuji, H.; Li, X.; Caricato, M.; Marenich, A. V.; Bloino, J.; Janesko, B. G.; Gomperts, R.; Mennucci, B.; Hratchian, H. P.; Ortiz, J. V.; Izmaylov, A. F.; Sonnenberg, J. L.; Williams-Young, D.; Ding, F.; Lipparini, F.; Egidi, F.; Goings, J.; Peng, B.; Petrone, A.; Henderson, T.; Ranasinghe, D.; Zakrzewski, V. G.; Gao, J.; Rega, N.; Zheng, G.; Liang, W.; Hada, M.; Ehara, M.; Toyota, K.; Fukuda, R.; Hasegawa, J.; Ishida, M.; Nakajima, T.; Honda, Y.; Kitao, O.; Nakai, H.; Vreven, T.; Throssell, K.; Montgomery Jr., J. A.; Peralta, J. E.; Ogliaro, F.; Bearpark, M. J.; Heyd, J. J.; Brothers, E. N.; Kudin, K. N.; Staroverov, V. N.; Keith, T. A.; Kobayashi, R.; Normand, J.; Raghavachari, K.; Rendell, A. P.; Burant, J. C.; Iyengar, S. S.; Tomasi, J.; Cossi, M.; Millam, J. M.; Klene, M.; Adamo, C.; Cammi, R.; Ochterski, J. W.; Martin, R. L.; Morokuma, K.; Farkas, O.; Foresman, J. B.; Fox, D. J. *Gaussian16*, Gaussian, Inc., Wallingford, CT, 2016.

8 Zhao, Y. & Truhlar, D. G. The M06 suite of density functionals for main group thermochemistry, thermochemical kinetics, noncovalent interactions, excited states, and transition elements: two new functionals and systematic testing of four M06-class functionals and 12 other functionals. *Theoretical Chemistry Accounts* **120**, 215-241 (2008).

9 Furche, F. & Ahlrichs, R. Adiabatic time-dependent density functional methods for excited state properties. *J. Chem. Phys.,***117**, 7433-7447 (2002).

10 Fukui, K. The path of chemical reactions-the IRC approach. *Acc. Chem. Res.* **14**, 363-368 (1981).

11 Hratchian, H. P. & Schlegel, H. B. Accurate reaction paths using a Hessian based predictor–corrector integrator. *J. Chem. Phys.,***120**, 9918-9924 (2004).

12 Hratchian, H. & Schlegel, H. Using Hessian updating to increase the efficiency of a Hessian based predictor-corrector reaction path following method. *J. Chem. Phys.,***1**, 61-69 (2005).
