# Supplementary material for: N-Heterocyclic carbene-catalyzed enantioselective hetero-[10 + 2] annulation
Source: Commun Chem. 2020 Nov 27;3:177. doi: 10.1038/s42004-020-00425-7 (PMC9814252; doi:10.1038/s42004-020-00425-7)
Supplement: Supplementary file 2 — Description of Additional Supplementary Files [file 42004_2020_425_MOESM2_ESM.docx]

**Description of Additional Supplementary Files**

File Name: Supplementary Data1

Description: crystallographic cif data of **3e**

File Name: Supplementary Data2

Description: The coordinates for the corresponding structures and IRC of transition state TS(II)R.
