# Supplementary material for: N-Heterocyclic carbene-catalyzed enantioselective hetero-[10 + 2] annulation
Source: Commun Chem. 2020 Nov 27;3:177. doi: 10.1038/s42004-020-00425-7 (PMC9814252; doi:10.1038/s42004-020-00425-7)
Supplement: Supplementary file 4 — Supplementary Data 2 [file 42004_2020_425_MOESM4_ESM.docx]

**Supplementary data 2**

The coordinates for the corresponding structures

| II  6 -0.216759000 -1.430455000 -0.624863000  6 -0.826181000 -3.248802000 0.415520000  7 -1.278062000 -2.196550000 -0.341765000  7 0.457856000 -3.171144000 0.609136000  6 2.153287000 -1.557046000 -0.020429000  6 2.667424000 -1.052908000 1.168863000  6 2.883117000 -1.460070000 -1.195347000  6 3.924482000 -0.475788000 1.187194000  6 4.144590000 -0.881822000 -1.184633000  6 4.659562000 -0.391640000 0.008495000  7 0.819428000 -2.032393000 -0.049011000  6 -1.768487000 -4.298030000 0.922101000  1 -1.895871000 -4.185497000 2.006093000  1 -1.350078000 -5.287151000 0.729029000  8 -2.995202000 -4.220489000 0.233483000  6 -2.697639000 -1.953140000 -0.713189000  6 -3.547455000 -2.926613000 0.142110000  1 -4.500306000 -3.055944000 -0.376040000  1 -2.779887000 -2.111081000 -1.788809000  6 -3.797171000 -2.175492000 1.469205000  1 -4.732711000 -2.480887000 1.942156000  1 -2.992615000 -2.344831000 2.194839000  6 -3.790245000 -0.736558000 1.022779000  6 -4.303042000 0.375249000 1.682839000  6 -3.180099000 -0.603465000 -0.230045000  6 -4.200807000 1.623711000 1.078623000  1 -4.776844000 0.275747000 2.653550000  6 -3.104217000 0.627967000 -0.869247000  6 -3.614895000 1.718339000 -0.178884000  1 -4.573280000 2.521606000 1.555754000  1 -2.668401000 0.755858000 -1.853008000  7 -3.536972000 3.041322000 -0.818452000  8 -3.090047000 3.095277000 -1.948556000  8 -3.932674000 3.999490000 -0.179951000  6 0.621016000 3.084803000 -0.428561000  6 0.996452000 2.161976000 0.623365000  6 1.618677000 2.643859000 1.810164000  6 1.833893000 3.989405000 1.931161000  6 1.447052000 4.907868000 0.901369000  6 0.855504000 4.477830000 -0.255511000  6 0.079361000 2.298809000 -1.447997000  6 0.157935000 0.978715000 -0.949608000  1 1.917100000 1.939995000 2.582887000  1 2.309212000 4.382089000 2.825260000  1 1.635422000 5.966476000 1.051326000  1 0.569702000 5.182493000 -1.031184000  1 -0.329096000 2.608807000 -2.399721000  7 0.703254000 0.895481000 0.314170000  6 -0.302838000 -0.217611000 -1.548641000  8 -0.862287000 -0.436665000 -2.620896000  9 2.359254000 -1.892324000 -2.336022000  9 4.854616000 -0.797803000 -2.302436000  9 5.862103000 0.161015000 0.024448000  9 4.416022000 0.027625000 2.312955000  9 1.940861000 -1.087419000 2.271686000 |
| --- |
| 1  6 -3.325084000 -0.467486000 0.000091000  6 -2.319425000 -1.430920000 -0.000160000  6 -0.983136000 -1.046536000 -0.000253000  6 -0.656226000 0.314405000 -0.000105000  6 -1.669702000 1.279397000 0.000105000  6 -3.000719000 0.889343000 0.000219000  1 -4.366059000 -0.774662000 0.000174000  1 -2.575671000 -2.484831000 -0.000291000  1 -0.207503000 -1.803658000 -0.000516000  1 -1.388393000 2.327162000 0.000198000  1 -3.786288000 1.637444000 0.000415000  6 0.742660000 0.808336000 -0.000137000  8 1.050836000 1.975770000 -0.000378000  6 1.879540000 -0.238857000 0.000131000  9 1.794272000 -1.025906000 1.082449000  9 3.067693000 0.345753000 0.000354000  9 1.794676000 -1.025815000 -1.082392000 |
| TS3(II-III)R  6 -0.957354000 -1.518492000 0.452820000  6 -1.171819000 -3.034095000 -1.101084000  7 -0.270687000 -2.455350000 -0.230739000  7 -2.361346000 -2.542063000 -0.953816000  6 -3.343685000 -0.841849000 0.400382000  6 -3.925850000 -0.999343000 1.651931000  6 -3.910255000 0.018608000 -0.537761000  6 -5.032724000 -0.236142000 2.000099000  6 -5.019093000 0.773376000 -0.193955000  6 -5.572121000 0.648063000 1.076596000  7 -2.216837000 -1.617649000 0.028363000  6 -0.763129000 -4.005010000 -2.160436000  1 -0.600733000 -3.444108000 -3.091762000  1 -1.562406000 -4.728215000 -2.324364000  8 0.388709000 -4.708997000 -1.771609000  6 1.064993000 -3.102981000 0.013077000  6 1.415378000 -3.893103000 -1.276833000  1 2.220551000 -4.579430000 -1.003986000  1 0.930585000 -3.744401000 0.886581000  6 2.000501000 -2.794847000 -2.196866000  1 2.571932000 -3.203720000 -3.031577000  1 1.212420000 -2.139634000 -2.589586000  6 2.836836000 -2.054698000 -1.183951000  6 4.064775000 -1.428150000 -1.345638000  6 2.282261000 -2.217750000 0.095078000  6 4.756295000 -0.996192000 -0.215022000  1 4.499204000 -1.301267000 -2.331402000  6 2.962726000 -1.821770000 1.231335000  6 4.198133000 -1.209594000 1.037827000  1 5.718869000 -0.505026000 -0.286740000  1 2.583034000 -1.977128000 2.233667000  7 4.943017000 -0.776296000 2.224482000  8 4.364269000 -0.821424000 3.297166000  8 6.089114000 -0.397105000 2.072646000  6 2.591131000 1.366567000 1.500357000  6 2.223047000 1.281722000 0.125208000  6 3.050825000 1.838971000 -0.864592000  6 4.246382000 2.406161000 -0.468310000  6 4.637374000 2.452216000 0.889825000  6 3.815932000 1.948965000 1.877160000  6 1.551019000 0.720765000 2.223486000  6 0.652842000 0.283895000 1.266227000  1 2.770173000 1.797882000 -1.909415000  1 4.910895000 2.822161000 -1.219810000  1 5.594703000 2.889496000 1.153777000  1 4.117170000 1.969064000 2.920763000  1 1.502602000 0.516788000 3.284715000  7 1.026757000 0.614315000 -0.018953000  6 -0.488538000 -0.563543000 1.559093000  8 -1.065462000 -0.658184000 2.622872000  6 -0.449780000 1.427811000 -0.890846000  8 -1.464865000 1.085020000 -0.242696000  6 -0.293588000 0.728723000 -2.256080000  6 -0.029066000 2.879624000 -0.855206000  6 -0.217749000 3.543797000 0.358904000  6 0.508678000 3.568364000 -1.941595000  6 0.151833000 4.874981000 0.493585000  1 -0.649706000 2.991035000 1.187590000  6 0.862307000 4.910023000 -1.808722000  1 0.653927000 3.073018000 -2.895679000  6 0.692356000 5.562870000 -0.592859000  1 0.015819000 5.380047000 1.444596000  1 1.275169000 5.442660000 -2.659672000  1 0.977782000 6.605066000 -0.490332000  9 -1.088373000 1.316319000 -3.161871000  9 -0.673248000 -0.562213000 -2.188392000  9 0.950076000 0.729382000 -2.764927000  9 -3.461771000 -1.898479000 2.504799000  9 -5.596172000 -0.378297000 3.194451000  9 -6.639243000 1.363880000 1.398889000  9 -5.567603000 1.603779000 -1.072483000  9 -3.415629000 0.100392000 -1.758152000 |
| TS3(II-III)S  6 -1.032999000 0.474526000 -1.124489000  6 -1.258009000 2.646483000 -1.148601000  7 -0.360565000 1.617371000 -1.358729000  7 -2.435270000 2.196930000 -0.841497000  6 -3.421708000 0.019019000 -0.645617000  6 -3.979773000 -0.643059000 -1.738096000  6 -4.056724000 -0.040550000 0.592326000  6 -5.093226000 -1.452113000 -1.572639000  6 -5.175717000 -0.843691000 0.758650000  6 -5.686582000 -1.552859000 -0.320879000  7 -2.287294000 0.846602000 -0.856085000  6 -0.860981000 4.084265000 -1.236137000  1 -0.569274000 4.429752000 -0.234267000  1 -1.711994000 4.673923000 -1.578275000  8 0.180618000 4.243002000 -2.168170000  6 0.898567000 1.855294000 -2.151973000  6 1.251745000 3.355714000 -1.979496000  1 1.968858000 3.594330000 -2.768612000  1 0.655999000 1.581523000 -3.180797000  6 1.972838000 3.375262000 -0.615614000  1 2.578475000 4.271137000 -0.465342000  1 1.253521000 3.302647000 0.209257000  6 2.773338000 2.104714000 -0.723658000  6 4.009149000 1.792173000 -0.168251000  6 2.161094000 1.228610000 -1.626914000  6 4.648250000 0.623522000 -0.567718000  1 4.486526000 2.464384000 0.537366000  6 2.797591000 0.081500000 -2.072333000  6 4.042151000 -0.189325000 -1.520384000  1 5.614910000 0.335820000 -0.173489000  1 2.373404000 -0.589390000 -2.809223000  7 4.773883000 -1.371913000 -1.998237000  8 4.248441000 -2.048586000 -2.863734000  8 5.867983000 -1.591280000 -1.513663000  6 2.377550000 -2.780814000 -0.283740000  6 2.343018000 -1.706493000 0.666804000  6 3.408706000 -1.583415000 1.594500000  6 4.446971000 -2.481958000 1.536358000  6 4.477852000 -3.542602000 0.590260000  6 3.455806000 -3.698369000 -0.311100000  6 1.224213000 -2.628504000 -1.071350000  6 0.584324000 -1.486802000 -0.579912000  1 3.427412000 -0.793830000 2.334958000  1 5.273122000 -2.379491000 2.233714000  1 5.326348000 -4.218316000 0.578755000  1 3.477435000 -4.490692000 -1.053403000  1 0.907805000 -3.214268000 -1.923217000  7 1.248006000 -0.910251000 0.480166000  6 -0.593382000 -0.988372000 -1.253736000  8 -1.294619000 -1.640570000 -2.011753000  6 -0.230069000 -0.212536000 1.904804000  8 -1.295568000 -0.457731000 1.347368000  6 0.174357000 -1.238487000 2.979031000  6 0.234529000 1.208615000 2.113983000  6 1.552715000 1.570221000 2.405393000  6 -0.757634000 2.192366000 2.112013000  6 1.863947000 2.886915000 2.727313000  1 2.333849000 0.824004000 2.355006000  6 -0.442628000 3.515808000 2.421468000  1 -1.780137000 1.901648000 1.895765000  6 0.867187000 3.862994000 2.744640000  1 2.890185000 3.153835000 2.961139000  1 -1.227082000 4.266947000 2.438148000  1 1.112118000 4.888046000 3.004766000  9 -0.675660000 -1.078584000 4.011038000  9 1.407818000 -1.071783000 3.471503000  9 0.054262000 -2.486079000 2.544167000  9 -3.480932000 -0.471978000 -2.950002000  9 -3.645926000 0.688086000 1.615422000  9 -5.778098000 -0.908159000 1.938880000  9 -6.761692000 -2.307373000 -0.161444000  9 -5.617154000 -2.090815000 -2.610831000 |

The data of IRC of transition state TS(II)R

|  | E(a.u.) |  | E(a.u.) |
| --- | --- | --- | --- |
| 0 | -2792.567189 | 0 | -2792.567189 |
| -0.32676 | -2792.567253 | 0.32676 | -2792.567241 |
| -0.65311 | -2792.567459 | 0.6531 | -2792.567381 |
| -0.97951 | -2792.567802 | 0.97931 | -2792.567584 |
| -1.30598 | -2792.568238 | 1.30581 | -2792.567829 |
| -1.63241 | -2792.568692 | 1.63236 | -2792.568099 |
| -1.95866 | -2792.569092 | 1.95896 | -2792.568383 |
| -2.28441 | -2792.569404 | 2.28558 | -2792.568675 |
| -2.6098 | -2792.569646 | 2.61222 | -2792.568978 |
| -2.93564 | -2792.569849 | 2.93885 | -2792.569287 |
| -3.26194 | -2792.570029 | 3.26548 | -2792.569594 |
| -3.58843 | -2792.570192 | 3.5921 | -2792.569891 |
| -3.9149 | -2792.570341 | 3.9187 | -2792.570169 |
| -4.24072 | -2792.570463 | 4.24527 | -2792.570427 |
| -4.55921 | -2792.570503 | 4.57184 | -2792.570663 |
| -4.8645 | -2792.570487 | 4.89833 | -2792.57088 |
| -5.13646 | -2792.570733 | 5.22472 | -2792.571077 |
| -5.45222 | -2792.570843 | 5.55012 | -2792.571246 |
| -5.77214 | -2792.57093 | 5.87106 | -2792.571392 |
| -6.08551 | -2792.571029 | 6.18803 | -2792.571494 |
| -6.40763 | -2792.571114 | 6.49182 | -2792.571661 |
| -6.72724 | -2792.571198 | 6.80836 | -2792.571796 |
| -7.05105 | -2792.571274 | 7.1258 | -2792.571919 |
| -7.37304 | -2792.571345 | 7.44134 | -2792.572035 |
| -7.69499 | -2792.571408 | 7.7566 | -2792.57213 |
| -8.01308 | -2792.571467 | 8.06553 | -2792.572245 |
| -8.32927 | -2792.57152 | 8.37993 | -2792.57234 |
| -8.64271 | -2792.571572 | 8.68988 | -2792.57244 |
| -8.95615 | -2792.571623 | 9.00135 | -2792.57254 |
| -9.27186 | -2792.57167 | 9.31391 | -2792.572643 |
| -9.58833 | -2792.571715 | 9.62925 | -2792.572746 |
| -9.90683 | -2792.571754 | 9.94792 | -2792.572832 |
| -0.22207 | -2792.571789 | 10.2604 | -2792.572923 |
| -0.53432 | -2792.571815 | 10.57354 | -2792.572985 |
| -0.83887 | -2792.571854 | 10.87629 | -2792.573092 |
| -1.14832 | -2792.571887 | 11.19022 | -2792.573163 |
| -1.45816 | -2792.571915 | 11.49695 | -2792.573258 |
| -1.76667 | -2792.57194 | 11.8115 | -2792.573328 |
| -2.07313 | -2792.571964 | 12.12034 | -2792.573403 |
| -2.37794 | -2792.571992 | 12.43059 | -2792.573469 |
| -2.68925 | -2792.572015 | 12.73729 | -2792.57354 |
| -2.99903 | -2792.572036 | 13.04529 | -2792.573595 |
| -3.30811 | -2792.572054 | 13.34926 | -2792.573665 |
| -3.61352 | -2792.572053 | 13.65653 | -2792.573701 |
| -3.90909 | -2792.572067 | 13.95302 | -2792.573773 |
| -4.20287 | -2792.572082 | 14.25751 | -2792.573745 |
| -4.49496 | -2792.572116 | 14.54122 | -2792.573835 |
| -4.80392 | -2792.572129 | 14.83742 | -2792.573908 |
| -5.10547 | -2792.572145 | 15.14234 | -2792.573951 |
| -5.41327 | -2792.572143 | 15.44491 | -2792.573979 |
| -5.70536 | -2792.572152 | 15.73977 | -2792.573992 |
| -6.00093 | -2792.572157 | 16.03039 | -2792.574014 |
| -6.2936 | -2792.572181 | 16.3178 | -2792.574043 |
| -6.59647 | -2792.57219 | 16.59968 | -2792.574133 |
| -6.88986 | -2792.572204 | 16.91145 | -2792.574137 |
| -7.19411 | -2792.572207 | 17.20036 | -2792.574208 |
| -7.48862 | -2792.572213 | 17.52089 | -2792.574242 |
| -7.7813 | -2792.572218 | 17.8386 | -2792.574262 |
| -8.075 | -2792.572229 | 18.14355 | -2792.574289 |
| -8.37757 | -2792.57222 | 18.4454 | -2792.574288 |
| -8.65471 | -2792.572225 | 18.7376 | -2792.57429 |
| -8.9437 | -2792.572231 | 19.02445 | -2792.574255 |
| -9.23989 | -2792.572241 | 19.29545 | -2792.57432 |
| -9.53156 | -2792.572245 | 19.60238 | -2792.574233 |
| -9.82359 | -2792.572246 | 19.85866 | -2792.574375 |
| -0.11729 | -2792.572235 | 20.17519 | -2792.574268 |
| -0.40472 | -2792.572229 | 20.42359 | -2792.574402 |
| -0.69499 | -2792.572206 | 20.73864 | -2792.574391 |
| -0.97022 | -2792.572215 | 21.02434 | -2792.574421 |
| -1.25476 | -2792.572196 | 21.33221 | -2792.574406 |
| -1.54612 | -2792.572214 | 21.609 | -2792.574443 |
| -1.85239 | -2792.572227 | 21.91721 | -2792.574428 |
| -2.15142 | -2792.572226 | 22.19091 | -2792.574468 |
| -2.4383 | -2792.572237 | 22.50541 | -2792.574435 |
| -2.7307 | -2792.572235 | 22.77219 | -2792.574481 |
| -3.0169 | -2792.572243 | 23.08627 | -2792.574438 |
| -3.30738 | -2792.572243 | 23.33924 | -2792.574493 |
| -3.59138 | -2792.57225 | 23.65727 | -2792.574459 |
| -3.89003 | -2792.572249 | 23.9175 | -2792.574505 |
| -4.18202 | -2792.572258 | 24.23634 | -2792.574488 |
| -4.47875 | -2792.572267 |  |  |
| -4.77441 | -2792.572272 |  |  |
| -5.06971 | -2792.572275 |  |  |
| -5.36855 | -2792.572274 |  |  |
| -5.6649 | -2792.572275 |  |  |
| -5.96491 | -2792.572272 |  |  |
| -6.259 | -2792.572272 |  |  |
| -6.55216 | -2792.572269 |  |  |
| -6.84264 | -2792.572265 |  |  |
| -7.12567 | -2792.572266 |  |  |
| -7.41812 | -2792.572267 |  |  |
| -7.71812 | -2792.572272 |  |  |
| -8.01628 | -2792.572281 |  |  |
| -8.316 | -2792.572286 |  |  |
| -8.61347 | -2792.57229 |  |  |
| -8.9105 | -2792.57229 |  |  |
| -9.2043 | -2792.572293 |  |  |
| -9.50043 | -2792.5723 |  |  |
| -9.8053 | -2792.572305 |  |  |
| -0.10397 | -2792.572312 |  |  |
| -0.40339 | -2792.572311 |  |  |
| -0.69309 | -2792.572312 |  |  |
| -0.9882 | -2792.572313 |  |  |
| -1.28591 | -2792.572319 |  |  |
| -1.59128 | -2792.572323 |  |  |
| -1.89341 | -2792.572324 |  |  |
| -2.19119 | -2792.572319 |  |  |
| -2.48007 | -2792.572323 |  |  |
| -2.77049 | -2792.572322 |  |  |
| -3.05981 | -2792.57234 |  |  |
| -3.37594 | -2792.572343 |  |  |
| -3.67379 | -2792.572351 |  |  |
| -3.97481 | -2792.572353 |  |  |
| -4.27292 | -2792.572356 |  |  |
| -4.56915 | -2792.572358 |  |  |
| -4.86416 | -2792.572362 |  |  |
| -5.16307 | -2792.572367 |  |  |
| -5.45964 | -2792.572375 |  |  |
| -5.75789 | -2792.572378 |  |  |
| -6.05133 | -2792.572378 |  |  |
| -6.33894 | -2792.572382 |  |  |
| -6.6332 | -2792.572392 |  |  |
| -6.94245 | -2792.572401 |  |  |
| -7.2462 | -2792.572407 |  |  |
| -7.54847 | -2792.572416 |  |  |
| -7.85407 | -2792.572422 |  |  |
| -8.15621 | -2792.572431 |  |  |
| -8.46029 | -2792.572438 |  |  |
| -8.76412 | -2792.572442 |  |  |
| -9.06125 | -2792.57245 |  |  |
| -9.36013 | -2792.572445 |  |  |
| -9.6486 | -2792.572457 |  |  |
| -9.94022 | -2792.572459 |  |  |
| -0.23093 | -2792.572484 |  |  |
| -0.54781 | -2792.572485 |  |  |
| -0.84671 | -2792.572494 |  |  |
| -1.14658 | -2792.572487 |  |  |
| -1.4403 | -2792.572478 |  |  |
| -1.72701 | -2792.572487 |  |  |
| -2.00411 | -2792.572507 |  |  |
| -2.3019 | -2792.572522 |  |  |
| -2.6041 | -2792.572526 |  |  |
| -2.90092 | -2792.572536 |  |  |
| -3.19855 | -2792.572537 |  |  |
| -3.48847 | -2792.57256 |  |  |
| -3.79179 | -2792.572565 |  |  |
| -4.08537 | -2792.57259 |  |  |
| -4.39758 | -2792.572582 |  |  |
| -4.68598 | -2792.572603 |  |  |
| -4.99122 | -2792.572591 |  |  |
| -5.27459 | -2792.572588 |  |  |
| -5.56943 | -2792.572575 |  |  |
| -5.85931 | -2792.572512 |  |  |
| -6.11882 | -2792.57256 |  |  |
| -6.41113 | -2792.57253 |  |  |
| -6.69364 | -2792.572595 |  |  |
| -6.99178 | -2792.572566 |  |  |
| -7.25873 | -2792.57262 |  |  |
| -7.56373 | -2792.572562 |  |  |
| -7.8322 | -2792.572631 |  |  |
| -8.13844 | -2792.572564 |  |  |
| -8.39004 | -2792.572639 |  |  |
| -8.6965 | -2792.572551 |  |  |
| -8.95398 | -2792.572638 |  |  |
| -9.25569 | -2792.572575 |  |  |
| -9.5128 | -2792.572675 |  |  |
| -9.82412 | -2792.572599 |  |  |
| -0.08225 | -2792.572685 |  |  |
| -0.389 | -2792.572646 |  |  |
| -0.64831 | -2792.572689 |  |  |
| -0.95302 | -2792.572672 |  |  |
| -1.23342 | -2792.572701 |  |  |
| -1.53249 | -2792.572696 |  |  |
| -1.81253 | -2792.572719 |  |  |
| -2.12283 | -2792.572709 |  |  |
| -2.40272 | -2792.572733 |  |  |
| -2.70777 | -2792.572728 |  |  |
| -2.99166 | -2792.572737 |  |  |
| -3.28752 | -2792.57273 |  |  |
| -3.57182 | -2792.572747 |  |  |
| -3.87243 | -2792.572757 |  |  |
| -4.17009 | -2792.572758 |  |  |
| -4.46278 | -2792.572765 |  |  |
| -4.75783 | -2792.572759 |  |  |
| -5.04328 | -2792.572778 |  |  |
| -5.34643 | -2792.572786 |  |  |
| -5.64146 | -2792.572796 |  |  |
| -5.93545 | -2792.572803 |  |  |
| -6.23027 | -2792.572818 |  |  |
| -6.53276 | -2792.572831 |  |  |
| -6.83652 | -2792.572847 |  |  |
| -7.14051 | -2792.57286 |  |  |
| -7.44535 | -2792.572875 |  |  |
| -7.74826 | -2792.57289 |  |  |
| -8.04999 | -2792.572908 |  |  |
| -8.35142 | -2792.572923 |  |  |
| -8.64881 | -2792.572943 |  |  |
| -8.94866 | -2792.57296 |  |  |
| -9.2492 | -2792.572985 |  |  |
| -9.55439 | -2792.573001 |  |  |
| -9.85427 | -2792.573017 |  |  |
